# Measuring universal health coverage based on an index of effective coverage of health services in 204 countries and territories, 1990–2019: a systematic analysis for the Global Burden of Disease Study 2019

**DOI:** 10.1016/S0140-6736(20)30750-9

**Published:** 2020-10-17

**Authors:** GBD 2019 Universal Health Coverage Collaborators, Rafael Lozano, Nancy Fullman, John Everett Mumford, Megan Knight, Celine M Barthelemy, Cristiana Abbafati, Hedayat Abbastabar, Foad Abd-Allah, Mohammad Abdollahi, Aidin Abedi, Hassan Abolhassani, Akine Eshete Abosetugn, Lucas Guimarães Abreu, Michael R M Abrigo, Abdulaziz Khalid Abu Haimed, Abdelrahman I Abushouk, Maryam Adabi, Oladimeji M Adebayo, Victor Adekanmbi, Jaimie Adelson, Olatunji O Adetokunboh, Davoud Adham, Shailesh M Advani, Ashkan Afshin, Gina Agarwal, Pradyumna Agasthi, Seyed Mohammad Kazem Aghamir, Anurag Agrawal, Tauseef Ahmad, Rufus Olusola Akinyemi, Fares Alahdab, Ziyad Al-Aly, Khurshid Alam, Samuel B Albertson, Yihun Mulugeta Alemu, Robert Kaba Alhassan, Muhammad Ali, Saqib Ali, Vahid Alipour, Syed Mohamed Aljunid, François Alla, Majid Abdulrahman Hamad Almadi, Ali Almasi, Amir Almasi-Hashiani, Nihad A Almasri, Hesham M Al-Mekhlafi, Abdulaziz M Almulhim, Jordi Alonso, Rajaa M Al-Raddadi, Khalid A Altirkawi, Nelson Alvis-Guzman, Nelson J Alvis-Zakzuk, Saeed Amini, Mostafa Amini-Rarani, Fatemeh Amiri, Arianna Maever L Amit, Dickson A Amugsi, Robert Ancuceanu, Deanna Anderlini, Catalina Liliana Andrei, Sofia Androudi, Fereshteh Ansari, Alireza Ansari-Moghaddam, Carl Abelardo T Antonio, Catherine M Antony, Ernoiz Antriyandarti, Davood Anvari, Razique Anwer, Jalal Arabloo, Morteza Arab-Zozani, Aleksandr Y Aravkin, Olatunde Aremu, Johan Ärnlöv, Malke Asaad, Mehran Asadi-Aliabadi, Ali A Asadi-Pooya, Charlie Ashbaugh, Seyyed Shamsadin Athari, Maha Moh'd Wahbi Atout, Marcel Ausloos, Leticia Avila-Burgos, Beatriz Paulina Ayala Quintanilla, Getinet Ayano, Martin Amogre Ayanore, Yared Asmare Aynalem, Getie Lake Aynalem, Muluken Altaye Ayza, Samad Azari, Peter S Azzopardi, Darshan B B, Ebrahim Babaee, Ashish D Badiye, Mohammad Amin Bahrami, Atif Amin Baig, Mohammad Hossein Bakhshaei, Ahad Bakhtiari, Shankar M Bakkannavar, Arun Balachandran, Shelly Balassyano, Maciej Banach, Srikanta K Banerjee, Palash Chandra Banik, Agegnehu Bante Bante, Simachew Animen Bante, Suzanne Lyn Barker-Collo, Till Winfried Bärnighausen, Lope H Barrero, Quique Bassat, Sanjay Basu, Bernhard T Baune, Mohsen Bayati, Bayisa Abdissa Baye, Neeraj Bedi, Ettore Beghi, Masoud Behzadifar, Tariku Tesfaye Tesfaye Bekuma, Michelle L Bell, Isabela M Bensenor, Adam E Berman, Eduardo Bernabe, Robert S Bernstein, Akshaya Srikanth Bhagavathula, Dinesh Bhandari, Pankaj Bhardwaj, Anusha Ganapati Bhat, Krittika Bhattacharyya, Suraj Bhattarai, Zulfiqar A Bhutta, Ali Bijani, Boris Bikbov, Ver Bilano, Antonio Biondi, Binyam Minuye Birihane, Moses John Bockarie, Somayeh Bohlouli, Hunduma Amensisa Bojia, Srinivasa Rao Rao Bolla, Archith Boloor, Oliver J Brady, Dejana Braithwaite, Paul Svitil Briant, Andrew M Briggs, Nikolay Ivanovich Briko, Sharath Burugina Nagaraja, Reinhard Busse, Zahid A Butt, Florentino Luciano Caetano dos Santos, Lucero Cahuana-Hurtado, Luis Alberto Cámera, Rosario Cárdenas, Giulia Carreras, Juan J Carrero, Felix Carvalho, Joao Mauricio Castaldelli-Maia, Carlos A Castañeda-Orjuela, Giulio Castelpietra, Franz Castro, Ferrán Catalá-López, Kate Causey, Christopher R Cederroth, Kelly M Cercy, Ester Cerin, Joht Singh Chandan, Angela Y Chang, Jaykaran Charan, Vijay Kumar Chattu, Sarika Chaturvedi, Ken Lee Chin, Daniel Youngwhan Cho, Jee-Young Jasmine Choi, Hanne Christensen, Dinh-Toi Chu, Michael T Chung, Liliana G Ciobanu, Massimo Cirillo, Haley Comfort, Kelly Compton, Paolo Angelo Cortesi, Vera Marisa Costa, Ewerton Cousin, Saad M A Dahlawi, Giovanni Damiani, Lalit Dandona, Rakhi Dandona, Jiregna Darega Gela, Aso Mohammad Darwesh, Ahmad Daryani, Aditya Prasad Dash, Gail Davey, Claudio Alberto Dávila-Cervantes, Kairat Davletov, Jan-Walter De Neve, Edgar Denova-Gutiérrez, Kebede Deribe, Nikolaos Dervenis, Rupak Desai, Samath Dhamminda Dharmaratne, Govinda Prasad Dhungana, Mostafa Dianatinasab, Diana Dias da Silva, Daniel Diaz, Ilse N Dippenaar, Hoa Thi Do, Fariba Dorostkar, Leila Doshmangir, Bruce B Duncan, Andre Rodrigues Duraes, Arielle Wilder Eagan, David Edvardsson, Iman El Sayed, Maha El Tantawi, Islam Y Elgendy, Iqbal RF Elyazar, Khalil Eskandari, Sharareh Eskandarieh, Saman Esmaeilnejad, Alireza Esteghamati, Oluchi Ezekannagha, Tamer Farag, Mohammad Farahmand, Emerito Jose A Faraon, Carla Sofia e Sá Farinha, Andrea Farioli, Pawan Sirwan Faris, Andre Faro, Mehdi Fazlzadeh, Valery L Feigin, Eduarda Fernandes, Pietro Ferrara, Garumma Tolu Feyissa, Irina Filip, Florian Fischer, James L Fisher, Luisa Sorio Flor, Nataliya A Foigt, Morenike Oluwatoyin Folayan, Artem Alekseevich Fomenkov, Masoud Foroutan, Joel Msafiri Francis, Weijia Fu, Takeshi Fukumoto, João M Furtado, Mohamed M Gad, Abhay Motiramji Gaidhane, Emmanuela Gakidou, Natalie C Galles, Silvano Gallus, William M Gardner, Biniyam Sahiledengle Geberemariyam, Abiyu Mekonnen Gebrehiwot, Leake G Gebremeskel, Gebreamlak Gebremedhn Gebremeskel, Hailay Abrha Gesesew, Keyghobad Ghadiri, Mansour Ghafourifard, Ahmad Ghashghaee, Nermin Ghith, Asadollah Gholamian, Syed Amir Gilani, Paramjit Singh Gill, Tiffany K Gill, Themba G Ginindza, Mojgan Gitimoghaddam, Giorgia Giussani, Mustefa Glagn, Elena V Gnedovskaya, Myron Anthony Godinho, Salime Goharinezhad, Sameer Vali Gopalani, Amir Hossein Goudarzian, Bárbara Niegia Garcia Goulart, Mohammed Ibrahim Mohialdeen Gubari, Rafael Alves Guimarães, Rashid Abdi Guled, Teklemariam Gultie, Yuming Guo, Rajeev Gupta, Rahul Gupta, Nima Hafezi-Nejad, Abdul Hafiz, Teklehaimanot Gereziher Haile, Randah R Hamadeh, Sajid Hameed, Samer Hamidi, Chieh Han, Hannah Han, Demelash Woldeyohannes Handiso, Asif Hanif, Graeme J Hankey, Josep Maria Haro, Ahmed I Hasaballah, Md Mehedi Hasan, Abdiwahab Hashi, Shoaib Hassan, Amr Hassan, Soheil Hassanipour, Hadi Hassankhani, Rasmus J Havmoeller, Simon I Hay, Khezar Hayat, Golnaz Heidari, Reza Heidari-Soureshjani, Delia Hendrie, Claudiu Herteliu, Thomas R Hird, Hung Chak Ho, Michael K Hole, Ramesh Holla, Bruce Hollingsworth, Praveen Hoogar, Kathleen Pillsbury Hopf, Nobuyuki Horita, Naznin Hossain, Mostafa Hosseini, Mehdi Hosseinzadeh, Mihaela Hostiuc, Sorin Hostiuc, Mowafa Househ, Vivian Chia-rong Hsieh, Guoqing Hu, Tanvir M Huda, Ayesha Humayun, Bing-Fang Hwang, Ivo Iavicoli, Segun Emmanuel Ibitoye, Nayu Ikeda, Olayinka Stephen Ilesanmi, Irena M Ilic, Milena D Ilic, Leeberk Raja Inbaraj, Usman Iqbal, Seyed Sina Naghibi Irvani, Caleb Mackay Salpeter Irvine, M Mofizul Islam, Sheikh Mohammed Shariful Islam, Farhad Islami, Hiroyasu Iso, Chinwe Juliana Iwu, Chidozie C D Iwu, Jalil Jaafari, Farhad Jadidi-Niaragh, Morteza Jafarinia, Deepa Jahagirdar, Mohammad Ali Jahani, Nader Jahanmehr, Mihajlo Jakovljevic, Hosna Janjani, Tahereh Javaheri, Achala Upendra Jayatilleke, Ensiyeh Jenabi, Ravi Prakash Jha, Vivekanand Jha, John S Ji, Peng Jia, Yetunde O John-Akinola, Jost B Jonas, Farahnaz Joukar, Jacek Jerzy Jozwiak, Mikk Jürisson, Zubair Kabir, Leila R Kalankesh, Rohollah Kalhor, Aruna M Kamath, Tanuj Kanchan, Neeti Kapoor, Behzad Karami Matin, Marina Karanikolos, Seyed M Karimi, Nicholas J Kassebaum, Srinivasa Vittal Katikireddi, Gbenga A Kayode, Peter Njenga Keiyoro, Yousef Saleh Khader, Mohammad Khammarnia, Maseer Khan, Ejaz Ahmad Khan, Young-Ho Khang, Khaled Khatab, Amir M Khater, Mona M Khater, Mahalaqua Nazli Khatib, Maryam Khayamzadeh, Jagdish Khubchandani, Neda Kianipour, Young-Eun Kim, Yun Jin Kim, Ruth W Kimokoti, Yohannes Kinfu, Adnan Kisa, Katarzyna Kissimova-Skarbek, Mika Kivimäki, Cameron J Kneib, Jonathan M Kocarnik, Sonali Kochhar, Stefan Kohler, Jacek A Kopec, Anna V Korotkova, Vladimir Andreevich Korshunov, Soewarta Kosen, Anirudh Kotlo, Parvaiz A Koul, Ai Koyanagi, Kewal Krishan, Kris J Krohn, Nuworza Kugbey, Vaman Kulkarni, G Anil Kumar, Nithin Kumar, Manasi Kumar, Om P Kurmi, Dian Kusuma, Hmwe Hmwe Kyu, Carlo La Vecchia, Ben Lacey, Dharmesh Kumar Lal, Ratilal Lalloo, Iván Landires, Van Charles Lansingh, Anders O Larsson, Savita Lasrado, Kathryn Mei-Ming Lau, Paolo Lauriola, Jeffrey V Lazarus, Jorge R Ledesma, Paul H Lee, Shaun Wen Huey Lee, Andrew T Leever, Kate E LeGrand, James Leigh, Matilde Leonardi, Shanshan Li, Stephen S Lim, Lee-Ling Lim, Xuefeng Liu, Giancarlo Logroscino, Alan D Lopez, Platon D Lopukhov, Paulo A Lotufo, Alton Lu, Jianing Ma, Mohammed Madadin, Phetole Walter Mahasha, Morteza Mahmoudi, Azeem Majeed, Jeadran N Malagón-Rojas, Shokofeh Maleki, Deborah Carvalho Malta, Borhan Mansouri, Mohammad Ali Mansournia, Santi Martini, Francisco Rogerlândio Martins-Melo, Ira Martopullo, Benjamin Ballard Massenburg, Claudia I Mastrogiacomo, Manu Raj Mathur, Colm McAlinden, Martin McKee, Carlo Eduardo Medina-Solís, Birhanu Geta Meharie, Man Mohan Mehndiratta, Entezar Mehrabi Nasab, Fereshteh Mehri, Ravi Mehrotra, Teferi Mekonnen, Addisu Melese, Peter T N Memiah, Walter Mendoza, Ritesh G Menezes, George A Mensah, Tuomo J Meretoja, Atte Meretoja, Tomislav Mestrovic, Bartosz Miazgowski, Irmina Maria Michalek, Erkin M Mirrakhimov, Maryam Mirzaei, Mehdi Mirzaei-Alavijeh, Philip B Mitchell, Babak Moazen, Masoud Moghadaszadeh, Efat Mohamadi, Yousef Mohammad, Dara K Mohammad, Naser Mohammad Gholi Mezerji, Abdollah Mohammadian-Hafshejani, Shafiu Mohammed, Jemal Abdu Mohammed, Ali H Mokdad, Lorenzo Monasta, Stefania Mondello, Masoud Moradi, Maziar Moradi-Lakeh, Rahmatollah Moradzadeh, Paula Moraga, Joana Morgado-da-Costa, Shane Douglas Morrison, Abbas Mosapour, Jonathan F Mosser, Amin Mousavi Khaneghah, Moses K Muriithi, Ghulam Mustafa, Ashraf F Nabhan, Mehdi Naderi, Ahamarshan Jayaraman Nagarajan, Mohsen Naghavi, Behshad Naghshtabrizi, Mukhammad David Naimzada, Vinay Nangia, Jobert Richie Nansseu, Vinod C Nayak, Javad Nazari, Rawlance Ndejjo, Ionut Negoi, Ruxandra Irina Negoi, Subas Neupane, Kiirithio N Ngari, Georges Nguefack-Tsague, Josephine W Ngunjiri, Cuong Tat Nguyen, Diep Ngoc Nguyen, Huong Lan Thi Nguyen, Chukwudi A Nnaji, Shuhei Nomura, Ole F Norheim, Jean Jacques Noubiap, Christoph Nowak, Virginia Nunez-Samudio, Adrian Otoiu, Felix Akpojene Ogbo, Onome Bright Oghenetega, In-Hwan Oh, Emmanuel Wandera Okunga, Morteza Oladnabi, Andrew T Olagunju, Jacob Olusegun Olusanya, Bolajoko Olubukunola Olusanya, Mojisola Morenike Oluwasanu, Ahmed Omar Bali, Muktar Omer Omer, Kanyin L Ong, Obinna E Onwujekwe, Doris V V Ortega-Altamirano, Alberto Ortiz, Sergej M Ostojic, Nikita Otstavnov, Stanislav S Otstavnov, Simon Øverland, Mayowa O Owolabi, Jagadish Rao. Padubidri, Smita Pakhale, Raffaele Palladino, Adrian Pana, Songhomitra Panda-Jonas, Helena Ullyartha Pangaribuan, Mona Pathak, George C Patton, Sagun Paudel, Hamidreza Pazoki Toroudi, Spencer A Pease, Amy E Peden, Alyssa Pennini, Emmanuel K Peprah, Jeevan Pereira, David M Pigott, Thomas Pilgrim, Tessa M Pilz, Marina Pinheiro, Michael A Piradov, Meghdad Pirsaheb, Khem Narayan Pokhrel, Maarten J Postma, Hadi Pourjafar, Farshad Pourmalek, Reza Pourmirza Kalhori, Akram Pourshams, Sergio I Prada, Dimas Ria Angga Pribadi, Elisabetta Pupillo, Zahiruddin Quazi Syed, Amir Radfar, Ata Rafiee, Alireza Rafiei, Alberto Raggi, Fakher Rahim, Muhammad Aziz Rahman, Ali Rajabpour-Sanati, Saleem Muhammad Rana, Chhabi Lal Ranabhat, Sowmya J Rao, Davide Rasella, Vahid Rashedi, Goura Kishor Rath, Priya Rathi, Salman Rawaf, David Laith Rawaf, Lal Rawal, Reza Rawassizadeh, Christian Razo, Vishnu Renjith, Andre M N Renzaho, Bhageerathy Reshmi, Nima Rezaei, Seyed Mohammad Riahi, Daniel Cury Ribeiro, Jennifer Rickard, Nicholas L S Roberts, Leonardo Roever, Michele Romoli, Luca Ronfani, Gholamreza Roshandel, Enrico Rubagotti, Godfrey M Rwegerera, Siamak Sabour, Perminder S Sachdev, Basema Saddik, Masoumeh Sadeghi, Ehsan Sadeghi, Yahya Safari, Rajesh Sagar, Amirhossein Sahebkar, Mohammad Ali Sahraian, S. Mohammad Sajadi, Mohammad Reza Salahshoor, Marwa R Rashad Salem, Hosni Salem, Joshua Salomon, Hossein Samadi Kafil, Abdallah M Samy, Juan Sanabria, Milena M Santric-Milicevic, Sivan Yegnanarayana Iyer Saraswathy, Rodrigo Sarmiento-Suárez, Benn Sartorius, Arash Sarveazad, Brijesh Sathian, Thirunavukkarasu Sathish, Davide Sattin, Miloje Savic, Susan M Sawyer, Deepak Saxena, Alyssa N Sbarra, Lauren E Schaeffer, Silvia Schiavolin, Maria Inês Schmidt, Aletta Elisabeth Schutte, David C Schwebel, Falk Schwendicke, Soraya Seedat, Feng Sha, Saeed Shahabi, Amira A Shaheen, Masood Ali Shaikh, Morteza Shamsizadeh, Mohammed Shannawaz, Kiomars Sharafi, Fablina Sharara, Hamid Sharifi, David H Shaw, Aziz Sheikh, Abbas Sheikhtaheri, B Suresh Kumar Shetty, Kenji Shibuya, Wondimeneh Shibabaw Shiferaw, Mika Shigematsu, Jae Il Shin, Rahman Shiri, Reza Shirkoohi, K M Shivakumar, Mark G Shrime, Kerem Shuval, Soraya Siabani, Radoslaw Sierpinski, Inga Dora Sigfusdottir, Rannveig Sigurvinsdottir, Diego Augusto Santos Silva, João Pedro Silva, Biagio Simonetti, Kyle E Simpson, Jasvinder A Singh, Pushpendra Singh, Dhirendra Narain Sinha, Valentin Yurievich Skryabin, Emma U R Smith, Amin Soheili, Shahin Soltani, Moslem Soofi, Reed J.D. Sorensen, Joan B Soriano, Muluken Bekele Sorrie, Ireneous N Soyiri, Emma Elizabeth Spurlock, Chandrashekhar T Sreeramareddy, Jeffrey D Stanaway, Nicholas Steel, Caroline Stein, Mark A Stokes, Mu'awiyyah Babale Sufiyan, Hafiz Ansar Rasul Suleria, Iyad Sultan, Łukasz Szumowski, Rafael Tabarés-Seisdedos, Takahiro Tabuchi, Santosh Kumar Tadakamadla, Biruk Wogayehu Taddele, Degena Bahrey Tadesse, Amir Taherkhani, Animut Tagele Tamiru, Frank C Tanser, Md Ismail Tareque, Ingan Ukur Tarigan, Whitney L Teagle, Fabrizio Tediosi, Yonas Getaye Getaye Tefera, Freweini Gebrearegay Tela, Zemenu Tadesse Tessema, Bhaskar Thakur, Mariya Vladimirovna Titova, Marcello Tonelli, Roman Topor-Madry, Fotis Topouzis, Marcos Roberto Roberto Tovani-Palone, Bach Xuan Tran, Ravensara Travillian, Christopher E Troeger, Lorainne Tudor Car, Riaz Uddin, Irfan Ullah, Chukwuma David Umeokonkwo, Bhaskaran Unnikrishnan, Era Upadhyay, Olalekan A Uthman, Marco Vacante, Pascual R Valdez, Santosh Varughese, Tommi Juhani Vasankari, Yasser Vasseghian, Narayanaswamy Venketasubramanian, Francesco S Violante, Vasily Vlassov, Stein Emil Vollset, Avina Vongpradith, Theo Vos, Yasir Waheed, Magdalene K Walters, Richard G Wamai, Haidong Wang, Yuan-Pang Wang, Robert G Weintraub, Jordan Weiss, Andrea Werdecker, Ronny Westerman, Lauren B Wilner, Gebremariam Woldu, Charles D A Wolfe, Ai-Min Wu, Sarah Wulf Hanson, Yang Xie, Rixing Xu, Seyed Hossein Yahyazadeh Jabbari, Kazumasa Yamagishi, Yuichiro Yano, Sanni Yaya, Vahid Yazdi-Feyzabadi, Jamal A Yearwood, Yordanos Gizachew Yeshitila, Paul Yip, Naohiro Yonemoto, Mustafa Z Younis, Zabihollah Yousefi, Taraneh Yousefinezhadi, Hasan Yusefzadeh, Siddhesh Zadey, Telma Zahirian Moghadam, Syed Saoud Zaidi, Leila Zaki, Sojib Bin Zaman, Mohammad Zamani, Maryam Zamanian, Hamed Zandian, Mikhail Sergeevich Zastrozhin, Kaleab Alemayehu Zewdie, Yunquan Zhang, Xiu-Ju George Zhao, Yingxi Zhao, Peng Zheng, Cong Zhu, Arash Ziapour, Bianca S Zlavog, Sanjay Zodpey, Christopher J L Murray

## Abstract

**Background:**

Achieving universal health coverage (UHC) involves all people receiving the health services they need, of high quality, without experiencing financial hardship. Making progress towards UHC is a policy priority for both countries and global institutions, as highlighted by the agenda of the UN Sustainable Development Goals (SDGs) and WHO's Thirteenth General Programme of Work (GPW13). Measuring effective coverage at the health-system level is important for understanding whether health services are aligned with countries' health profiles and are of sufficient quality to produce health gains for populations of all ages.

**Methods:**

Based on the Global Burden of Diseases, Injuries, and Risk Factors Study (GBD) 2019, we assessed UHC effective coverage for 204 countries and territories from 1990 to 2019. Drawing from a measurement framework developed through WHO's GPW13 consultation, we mapped 23 effective coverage indicators to a matrix representing health service types (eg, promotion, prevention, and treatment) and five population-age groups spanning from reproductive and newborn to older adults (≥65 years). Effective coverage indicators were based on intervention coverage or outcome-based measures such as mortality-to-incidence ratios to approximate access to quality care; outcome-based measures were transformed to values on a scale of 0–100 based on the 2·5th and 97·5th percentile of location-year values. We constructed the UHC effective coverage index by weighting each effective coverage indicator relative to its associated potential health gains, as measured by disability-adjusted life-years for each location-year and population-age group. For three tests of validity (content, known-groups, and convergent), UHC effective coverage index performance was generally better than that of other UHC service coverage indices from WHO (ie, the current metric for SDG indicator 3.8.1 on UHC service coverage), the World Bank, and GBD 2017. We quantified frontiers of UHC effective coverage performance on the basis of pooled health spending per capita, representing UHC effective coverage index levels achieved in 2019 relative to country-level government health spending, prepaid private expenditures, and development assistance for health. To assess current trajectories towards the GPW13 UHC billion target—1 billion more people benefiting from UHC by 2023—we estimated additional population equivalents with UHC effective coverage from 2018 to 2023.

**Findings:**

Globally, performance on the UHC effective coverage index improved from 45·8 (95% uncertainty interval 44·2–47·5) in 1990 to 60·3 (58·7–61·9) in 2019, yet country-level UHC effective coverage in 2019 still spanned from 95 or higher in Japan and Iceland to lower than 25 in Somalia and the Central African Republic. Since 2010, sub-Saharan Africa showed accelerated gains on the UHC effective coverage index (at an average increase of 2·6% [1·9–3·3] per year up to 2019); by contrast, most other GBD super-regions had slowed rates of progress in 2010–2019 relative to 1990–2010. Many countries showed lagging performance on effective coverage indicators for non-communicable diseases relative to those for communicable diseases and maternal and child health, despite non-communicable diseases accounting for a greater proportion of potential health gains in 2019, suggesting that many health systems are not keeping pace with the rising non-communicable disease burden and associated population health needs. In 2019, the UHC effective coverage index was associated with pooled health spending per capita (*r*=0·79), although countries across the development spectrum had much lower UHC effective coverage than is potentially achievable relative to their health spending. Under maximum efficiency of translating health spending into UHC effective coverage performance, countries would need to reach $1398 pooled health spending per capita (US$ adjusted for purchasing power parity) in order to achieve 80 on the UHC effective coverage index. From 2018 to 2023, an estimated 388·9 million (358·6–421·3) more population equivalents would have UHC effective coverage, falling well short of the GPW13 target of 1 billion more people benefiting from UHC during this time. Current projections point to an estimated 3·1 billion (3·0–3·2) population equivalents still lacking UHC effective coverage in 2023, with nearly a third (968·1 million [903·5–1040·3]) residing in south Asia.

**Interpretation:**

The present study demonstrates the utility of measuring effective coverage and its role in supporting improved health outcomes for all people—the ultimate goal of UHC and its achievement. Global ambitions to accelerate progress on UHC service coverage are increasingly unlikely unless concerted action on non-communicable diseases occurs and countries can better translate health spending into improved performance. Focusing on effective coverage and accounting for the world's evolving health needs lays the groundwork for better understanding how close—or how far—all populations are in benefiting from UHC.

**Funding:**

Bill & Melinda Gates Foundation.

**Research in context**

**Evidence before this study**

Various approaches have been proposed for monitoring universal health coverage (UHC) service coverage, including those from WHO (ie, the UHC service coverage index, the official UN measure for the Sustainable Development Goal indicator 3.8.1) and the World Bank. Currently available service coverage metrics are heavily focused on infectious diseases as well as reproductive, neonatal, maternal, and child health, despite the recognition that advances towards UHC also require service provision for non-communicable diseases and delivering interventions to a broader range of population-age groups. Inconsistent trend estimation across indicators, if time series are generated, impedes measurements of progress—a priority emphasised in the member-state-led Political Declaration for the UN High-Level Meeting on Universal Health Coverage in 2019. Although the 2014 WHO and World Bank framework for UHC service coverage is explicitly focused on health-system effective coverage, efforts to date have focused on crude coverage or health-system resource inputs, or a combination of both. Effective coverage at the health-system level, or the fraction of potential health gains delivered by a health system, has yet to be incorporated into UHC monitoring efforts, even though WHO and member states have signalled increasing interest in understanding the impact of UHC beyond service coverage alone.

**Added value of this study**

Drawing from the WHO Thirteenth General Programme of Work (GPW13) Expert Reference Group and Task Force on Metrics recommendations on UHC monitoring and conceptual work on effective coverage of health systems, the present study offers a new measurement framework for UHC effective coverage, representing health needs and corresponding service types across the life course while accounting for potential health gains delivered to populations. The framework mapped 23 effective coverage indicators against five health service domains—promotion, prevention, treatment, rehabilitation, and palliation—and five population-age groups (ie, reproductive and newborn, children <5 years, children and adolescents aged 5–19 years, adults aged 20–64 years, and adults aged ≥65 years). Based on estimates from the Global Burden of Diseases, Injuries, and Risk Factors Study (GBD) 2019, these 23 effective coverage indicators involved either direct measures of intervention coverage (eg, antiretroviral therapy coverage) or outcome-based indicators, such as mortality-to-incidence ratios, to approximate access to quality care. We weighted each effective coverage indicator on the basis of potential health gains deliverable by health systems, as approximated by the disability-adjusted life-years associated with each effective coverage indicator, and aggregated them to produce the UHC effective coverage index. Three types of validity were assessed (content, known groups, and convergent) for the UHC effective coverage index and other multi-country service coverage measures (eg, the UHC service coverage index for 2017, as estimated by WHO, and the GBD 2017 UHC service coverage index for 2017). We also quantified relationships between pooled health spending per capita (ie, government expenditures, prepaid private spending, and development assistance for health) and UHC effective coverage performance to examine how well countries are currently translating resources into improved UHC effective coverage. Last, we estimated the number of population equivalents covered by effective health services from 2018 to 2023—a key component of WHO's GPW13—by assuming a direct translation of the UHC effective coverage index to a fractional metric and multiplying country-level population estimates.

**Implications of all the available evidence**

This study offers another step forward in measuring UHC effective coverage across settings, developing a measurement framework and methods for country and global stakeholders to better track progress in effective health service provision at the population level. Our results highlight the importance of including non-communicable disease indicators alongside interventions for reproductive, neonatal, maternal, and child health and for infectious diseases, as well as capturing potential health gains delivered by health systems at the population level. In combination, we expect these analytical advances to better identify where countries have improved effective health service delivery, and what health needs along the life course increasingly threaten further progress. Focusing on UHC effective coverage, both in terms of its measurement and its capacity for instilling greater accountability for improving health outcomes across the development spectrum, lays a data-driven path towards achieving UHC for all populations.

## Introduction

Universal health coverage (UHC) is viewed as a crucial avenue through which improved health for all can be attained,^1^, ^2^ by ensuring all people can receive quality health services they need, without experiencing financial hardship. Global agendas and actors have amplified calls for UHC in recent years, driven at least in part by the explicit inclusion of UHC achievement in target 3.8 of the UN Sustainable Development Goals (SDGs)^3^, ^4^, ^5^ and heightened emphasis within recent UN resolutions^1^ and WHO programmatic objectives (eg, the target of 1 billion more people benefiting from UHC from 2018 to 2023 as part of WHO's Thirteenth General Programme of Work [GPW13]).^6^, ^7^ Regional and country-driven efforts to elevate UHC on policy agendas have occurred as well, both building upon long established UHC programmes (eg, in Japan,^8^ much of western Europe,^9^, ^10^ and many countries in Latin America^11^, ^12^) and galvanising newer commitments to UHC implementation (eg, in India, Kenya, and South Africa).^13^ To better understand how actions and investments are delivering on the ultimate goal of UHC—improving health outcomes—it is essential to quantify and track trends in effective health service provision, as well as the extent to which advances in service coverage correspond with the potential health gains populations should experience.

In 2014, WHO and the World Bank published a UHC measurement framework in which service coverage was defined as a spectrum of services—promotion, prevention, treatment, rehabilitation, and palliation—across the life cycle.^14^, ^15^ This framework emphasised the importance of providing services for individuals' health needs throughout their lifespans and quantifying effective coverage of interventions delivered by health systems. Conceptually, effective coverage unites intervention need, use, and quality into a single metric, representing the proportion of health gain that could be potentially received from an intervention relative to what is actually experienced.^16^, ^17^ At the health-system level, effective coverage aims to capture the fraction of total potential health gains actually delivered relative to what a health system could have theoretically delivered.^16^ To quantify such population-level health gains, Shengelia and colleagues outlined an approach to measure an aggregate of health-system effective coverage.^16^ Effective coverage is a powerful measure: this metric not only demands accountability of intervention availability and use, but also requires that the services received are of sufficient quality to provide the health gains they are supposed to. Yet in practice, effective coverage has to date been rarely measured, particularly across countries and over time. Minimal uptake of effective coverage as a metric for UHC monitoring is at least partly due to data challenges, as most health data systems are not able to capture all three intervention components together (ie, need, use or receipt, and quality) and few data sources can adequately represent these dimensions for conditions involving more complex care (eg, cancer or stroke). Tracer or proxy indicators of effective coverage exist for certain interventions or cause groups (eg, cancers), and recent health-system research by Kruk and colleagues used mortality-to-incidence rates to garner insights into health-care quality in low-income to middle-income countries.^18^ Nevertheless, to date no multi-country UHC measurement effort to our knowledge has sought to estimate effective coverage across health service domains and population-age groups within a cohesive analytical platform.

Following the 2014 WHO/World Bank UHC monitoring framework and SDG adoption in 2015, several multi-country health service coverage indices have been developed to inform UHC measurement.^19^, ^20^, ^21^, ^22^, ^23^, ^24^, ^25^, ^26^ Although each effort has shown recognition of prevailing data limitations and challenges with operationalising UHC service coverage across myriad settings,^21^, ^24^ they each have limitations in how well they capture country-level trends and health service needs across the life course.^17^, ^27^, ^28^, ^29^, ^30^, ^31^ First, current indices primarily rely solely on household survey point estimates from multi-country survey series, which can lead to various measurement limitations (ie, being primarily focused on low-income to middle-income countries; restricted sets of interventions captured; and lags in data availability for understanding trends). Second, most indices include either risk factor indicators (eg, prevalence of non-smoking and non-raised blood pressure in the UHC service coverage index,^19^, ^20^, ^21^ the SDG indicator 3.8.1^4^) or health-system inputs or process indicators (eg, health workers per capita and hospital beds per capita in the UHC service coverage index; inpatient admission rates for Wagstaff and colleagues' service coverage index^23^, ^24^), or both. The use of such proxy indicators, as well as those influenced by factors outside the health system (eg, tobacco prevalence), for service coverage measurement could misattribute successes in health service provision or misrepresent UHC service coverage. With non-communicable diseases accounting for at least 60% of early death and disability worldwide,^32^ the omission of non-communicable disease indicators beyond risk factor prevalence proxies or cancer screening is at odds with the reality of countries' populations and health systems. Third, approaches used to construct overall indices of UHC service coverage typically involve somewhat arbitrary weighting schemes (eg, a series of geometric means^4^, ^19^, ^20^, ^21^ or weighted geometric means^23^, ^24^), and thus might not capture the alignment of services provided given a country's health and demographic profile. Last, none of these approaches explicitly accounts for the potential health gains delivered through the health system, a limitation that inhibits our collective understanding of whether or how gains in UHC are improving health outcomes for all.

Recent developments from WHO indicate a revived interest in using effective coverage for UHC monitoring; these include the WHO GPW13 Expert Reference Group (ERG) Task Force on Metrics recommendations on effective service coverage measurement^33^ and the WHA72 resolution recommending country pilots on monitoring UHC effective coverage.^7^ The GPW13 ERG also supported initial efforts to map health services against population-age groups within a measurement framework and to identify indicator options across the life course in order to estimate UHC effective coverage across countries.^33^ The present analysis contributes to this endeavour through the Global Burden of Diseases, Injuries, and Risk Factors Study (GBD) 2019, mapping 23 effective coverage indicators across health service types and population-age groups for 204 countries and territories from 1990 to 2019. Based on the construct of health-system effective coverage, we aggregated individual effective coverage indicators to produce an overall index using health gain weights, which were derived from country-specific disease burden estimates relative to theoretical levels of burden avertable given intervention levels and associated effectiveness. We compared the performance of this UHC effective coverage measure against that of previous multi-country UHC service coverage indices^21^, ^24^, ^26^ on a series of validity tests. We then assessed the relationships between pooled health spending per capita and index performance, aiming to capture how close—or how far—countries were in reaching UHC effective coverage frontiers relative to their current spending. Finally, we considered applications of this index for current global and national UHC priorities, such as translating index performance to the number of people covered by effective coverage for the GPW13 UHC billion target.

## Methods

### Overview

Our primary analysis involved three main steps: first, to use intervention coverage or compute proxy measures of effective coverage for 23 indicators; second, to calculate the fraction of potential health gains associated with each effective coverage indicator based on each location's disease burden profile; and third, to construct the overall UHC effective coverage index by weighting each effective coverage indicator relative to its health gains fraction. We then did secondary analyses, assessing UHC effective coverage performance relative to health spending and current trajectories towards the GPW13 UHC billion target. Each step is summarised below and further described in appendix 1 (pp 12–61).

This analysis uses estimates from the broader GBD 2019,^34^, ^35^, ^36^ covering 204 countries and territories from 1990 to 2019. Details of disease-specific, injury-specific, and coverage-specific data inputs and processing, statistical synthesis approaches, and final models are available in the accompanying GBD 2019 capstone publications.^34^, ^35^, ^36^ This study complies with the Guidelines for Accurate and Transparent Health Estimates Reporting (GATHER) statement,^37^ with further information provided in the appendix 1 (pp 69–72).

### Measurement of UHC effective coverage

#### Framework and indicators

**Figure 1:**
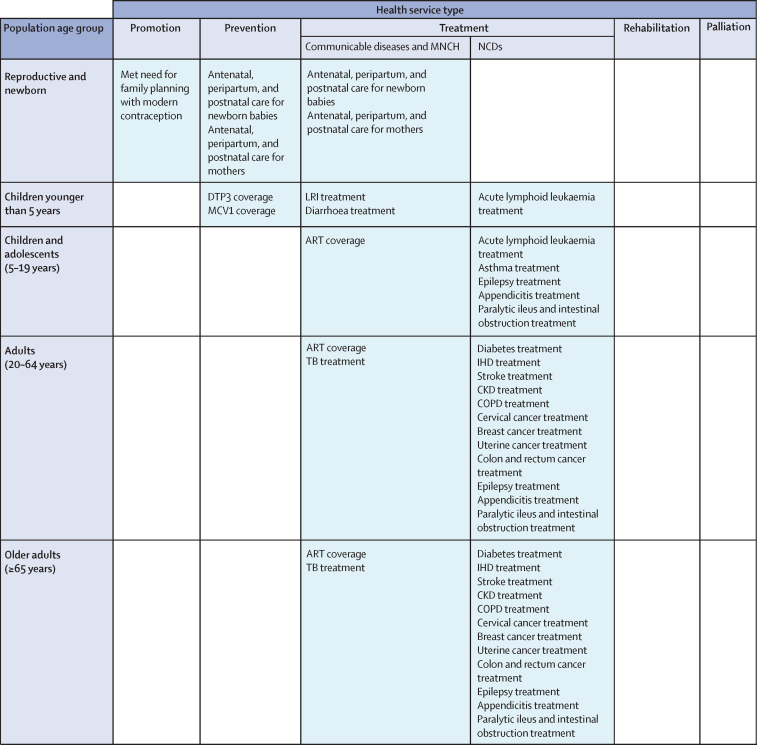
UHC effective coverage measurement framework Additional information about the framework development process and selection of effective coverage indicators can be found in appendix 1 (pp 12–28). ART=antiretroviral therapy. DTP3=diphtheria-tetanus-pertussis vaccine, 3 doses. IHD=ischaemic heart disease. CKD=chronic kidney disease. COPD=chronic obstructive pulmonary disease. LRI=lower respiratory infection. MCV1=measles-containing-vaccine, 1 dose. MNCH=maternal, neonatal, and child health. NCDs=non-communicable diseases. TB=tuberculosis. UHC=universal health coverage.

Development of the UHC effective coverage measurement framework and selection of effective coverage indicators was based on consultation, methods testing, and refinement via the WHO ERG on the GPW13 from 2017 to 2019;^7^, ^33^, ^38^, ^39^ the background and details of this process are provided in the appendix 1 (pp 12–28). The resulting framework (figure 1) and currently included effective coverage indicators (table 1) sought to represent the range of different health services that populations need across their lifespans while recognising present data gaps and appeals for measurement parsimony (appendix 1 pp 18–28).

**Table 1:** Details of the 23 effective coverage indicators included in the UHC effective coverage index, by health service type

	**Effective coverage indicator**	**Metric**	**Effective coverage indicator measurement**		**Health gain weight inputs**	**Effectiveness category**
			Numerator	Denominator		
**Reproductive and newborn**						
Promotion	Met need for family planning with modern contraception	Coverage	Females aged 15–49 years with demand for family planning met with modern contraception	Females aged 15–49 years with demand for family planning	50% of DALYs due to maternal disorders for females aged 10–54 years	5
Prevention; treatment, communicable diseases and MNCH	Antenatal, peripartum, and postnatal care for newborn babies	Early neonatal mortality rate	All-cause deaths during the first 7 days of life	Population of early neonates	Early neonatal deaths multiplied by life expectancy at birth (on the basis of theoretical minimum risk life table)	3
Prevention; treatment, communicable diseases and MNCH	Antenatal, peripartum, and postnatal care for mothers	Maternal mortality ratio	Deaths due to maternal disorders for females aged 10–54 years	Livebirths among females aged 10–54 years	50% of DALYs due to maternal disorders for females aged 10–54 years	1
**Children younger than 5 years**						
Prevention	DTP3 vaccine coverage	Coverage	Receipt of three doses of DTP vaccine among children aged 12–23 months	Children aged 12–23 months	DALYs due to diphtheria, tetanus, and pertussis for children younger than 5 years	1
Prevention	MCV1 coverage	Coverage	Receipt of MCV1 among children aged 12–23 months	Children aged 12–23 months	DALYs due to measles for children younger than 5 years	1
Treatment, communicable diseases and MNCH	LRI treatment	MIR	Mortality from LRIs for children younger than 5 years	Incidence of LRIs for children younger than 5 years	DALYs due to LRIs for children younger than 5 years	1
Treatment, communicable diseases and MNCH	Diarrhoea treatment	MIR	Mortality from diarrhoeal diseases for children younger than 5 years	Incidence of diarrhoeal diseases for children younger than 5 years	DALYs due to diarrhoeal diseases for children younger than 5 years	1
Treatment, NCDs	Acute lymphoid leukaemia treatment	MIR	Mortality from acute lymphoid leukaemia for children aged 1–4 years	Incidence of acute lymphoid leukaemia for children aged 1–4 years	DALYs due to acute lymphoid leukaemia for children aged 1–4 years	1
**Children and adolescents (5–19 years)**						
Treatment, communicable diseases and MNCH	ART coverage	Coverage	Populations aged 5–19 years living with HIV/AIDS and on ART	Populations aged 5–19 years living with HIV/AIDS	DALYs due to HIV for populations aged 5–19 years	1
Treatment, NCDs	Acute lymphoid leukaemia treatment	MIR	Mortality from acute lymphoid leukaemia for populations aged 5–19 years	Incidence of acute lymphoid leukaemia for populations aged 5–19 years	DALYs due to acute lymphoid leukaemia for populations 5–19 years	1
Treatment, NCDs	Asthma treatment	MPR	Mortality from asthma for populations aged 5–19 years	Prevalence of asthma for populations aged 5–19 years	DALYs due to asthma for populations aged 5–19 years	1
Treatment, NCDs	Epilepsy treatment	MPR	Mortality from epilepsy for populations aged 5–19 years	Prevalence of epilepsy for populations aged 5–19	DALYs due to epilepsy for populations aged 5–19 years	3
Treatment, NCDs	Appendicitis treatment	MIR	Mortality from appendicitis for populations aged 5–19 years	Incidence of appendicitis for populations aged 5–19 years	DALYs due to appendicitis for populations aged 5–19 years	1
Treatment, NCDs	Paralytic ileus and intestinal obstruction treatment	MIR	Mortality from paralytic ileus and intestinal obstruction for populations aged 5–19 years	Incidence of paralytic ileus and intestinal obstruction for populations aged 5–19 years	DALYs due to paralytic ileus and intestinal obstruction for populations aged 5–19 years	1
**Adults (20–64 years)**						
Treatment, communicable diseases and MNCH	ART coverage	Coverage	Population aged 20–64 years living with HIV/AIDS and on ART	Population aged 20–64 years living with HIV/AIDS	DALYs due to HIV for populations aged 20–64 years	1
Treatment, communicable diseases and MNCH	Tuberculosis treatment	MIR	Mortality from tuberculosis for populations aged 20–64 years	Incidence of tuberculosis for populations aged 20–64 years	DALYs due to tuberculosis for populations aged 20–64 years	1
Treatment, NCDs	Diabetes treatment	MPR	Mortality from diabetes for populations aged 20–64 years	Prevalence of diabetes for populations aged 20–64 years	DALYs due to diabetes for populations aged 20–64 years	3
Treatment, NCDs	IHD treatment	RSDR	Risk-standardised deaths from IHD for populations aged 20–64 years	Population aged 20–64 years	DALYs due to IHD for populations aged 20–64 years	2
Treatment, NCDs	Stroke treatment	MIR	Mortality from stroke for populations aged 20–64 years	Incidence of stroke for populations aged 20–64 years	DALYs due to stroke for populations aged 20–64 years	2
Treatment, NCDs	CKD treatment	MPR	Mortality from CKD for populations aged 20–64 years	Incidence of CKD for populations aged 20–64 years	DALYs due to CKD for populations aged 20–64 years	1
Treatment, NCDs	COPD treatment	MPR	Mortality from COPD for populations aged 20–64 years	Prevalence of COPD for populations aged 20–64 years	DALYs due to COPD for populations aged 20–64 years	3
Treatment, NCDs	Cervical cancer treatment	MIR	Mortality from cervical cancer for females aged 20–64 years	Incidence of cervical cancer for females aged 20–64 years	DALYs due to cervical cancer for females aged 20–64 years	1
Treatment, NCDs	Breast cancer treatment	MIR	Mortality from breast cancer for females aged 20–64 years	Incidence of breast cancer for females aged 20–64 years	DALYs due to breast cancer for females aged 20–64 years	1
Treatment, NCDs	Uterine cancer treatment	MIR	Mortality from uterine cancer for females aged 20–64 years	Incidence of uterine cancer for females aged 20–64 years	DALYs due to uterine cancer for females aged 20–64 years	1
Treatment, NCDs	Colon/rectum cancer treatment	MIR	Mortality from colon/rectum cancer for populations aged 20–64 years	Incidence of colon/rectum for populations aged 20–64 years	DALYs due to colon/rectum cancer for populations aged 20–64 years	1
Treatment, NCDs	Epilepsy treatment	MPR	Mortality from epilepsy for populations aged 20–64 years	Prevalence of epilepsy for populations aged 20–64 years	DALYs due to epilepsy for populations aged 20–64 years	3
Treatment, NCDs	Appendicitis treatment	MIR	Mortality from appendicitis for populations aged 20–64 years	Incidence of appendicitis for populations aged 20–64 years	DALYs due to appendicitis for populations aged 20–64 years	1
Treatment, NCDs	Paralytic ileus and intestinal obstruction treatment	MIR	Mortality from paralytic ileus and intestinal obstruction for populations aged 20–64 years	Incidence of paralytic ileus and intestinal obstruction for populations aged 20–64 years	DALYs due to paralytic ileus and intestinal obstruction for populations aged 20–64 years	1
**Older adults (≥65 years)**						
Treatment, communicable diseases and MNCH	ART coverage	Coverage	Population aged ≥65 years living with HIV/AIDS and on ART	Population aged ≥65 years living with HIV/AIDS	DALYs due to HIV for populations aged ≥65 years	2
Treatment, communicable diseases and MNCH	Tuberculosis treatment	MIR	Mortality from tuberculosis for populations aged ≥65 years	Incidence of tuberculosis for populations aged ≥65 years	DALYs due to tuberculosis for populations aged ≥65 years	2
Treatment, NCDs	Diabetes treatment	MPR	Mortality from diabetes for populations aged ≥65 years	Prevalence of diabetes for populations aged ≥65 years	DALYs due to diabetes for populations aged ≥65 years	4
Treatment, NCDs	IHD treatment	RSDR	Risk-standardised deaths from IHD for populations aged ≥65 years	Population aged ≥65 years	DALYs due to IHD for populations aged ≥65 years	3
Treatment, NCDs	Stroke treatment	MIR	Mortality from stroke for populations aged ≥65 years	Incidence of stroke for populations aged ≥65 years	DALYs due to stroke for populations aged ≥65 years	3
Treatment, NCDs	CKD treatment	MPR	Mortality from CKD for populations aged ≥65 years	Incidence of CKD for populations aged ≥65 years	DALYs due to CKD for populations aged ≥65 years	2
Treatment, NCDs	COPD treatment	MPR	Mortality from COPD for populations aged ≥65 years	Prevalence of COPD for populations aged ≥65 years	DALYs due to COPD for populations aged ≥65 years	4
Treatment, NCDs	Cervical cancer treatment	MIR	Mortality from cervical cancer for females aged ≥65 years	Incidence of cervical cancer for females aged ≥65 years	DALYs due to cervical cancer for females aged ≥65 years	2
Treatment, NCDs	Breast cancer treatment	MIR	Mortality from breast cancer for females aged ≥65 years	Incidence of breast cancer for females aged ≥65 years	DALYs due to breast cancer for females aged ≥65 years	2
Treatment, NCDs	Uterine cancer treatment	MIR	Mortality from uterine cancer for females aged ≥65 years	Incidence of uterine cancer for females aged ≥65 years	DALYs due to uterine cancer for females aged ≥65 years	2
Treatment, NCDs	Colon/rectum cancer treatment	MIR	Mortality from colon/rectum cancer for populations aged ≥65 years	Incidence of colon/rectum cancer for populations aged ≥65 years	DALYs due to colon/rectum cancer for populations aged ≥65 years	2
Treatment, NCDs	Epilepsy treatment	MPR	Mortality from epilepsy for populations aged ≥65 years	Prevalence of epilepsy for populations aged ≥65 years	DALYs due to epilepsy for populations aged ≥65 years	4
Treatment, NCDs	Appendicitis treatment	MIR	Mortality from appendicitis for populations aged ≥65 years	Incidence of appendicitis for populations aged ≥65 years	DALYs due to appendicitis for populations aged ≥65 years	2
Treatment, NCDs	Paralytic ileus and intestinal obstruction treatment	MIR	Mortality from paralytic ileus and intestinal obstruction for populations aged ≥65 years	Incidence of paralytic ileus and intestinal obstruction for populations aged ≥65 years	DALYs due to paralytic ileus and intestinal obstruction for populations aged ≥65 years	2

Additional information about the framework development process and selection of effective coverage indicators can be found in appendix 1 (pp 12–28). UHC=universal health coverage. DALYs=disability-adjusted life-years. MNCH=maternal, neonatal, and child health. DTP3=diphtheria-tetanus-pertussis vaccine, 3 doses. MCV1=measles-containing-vaccine, 1 dose. LRI=lower respiratory infection. MIR=mortality-to-incidence ratio. NCDs=non-communicable diseases. ART=antiretroviral therapy. MPR=mortality-to-prevalence ratio. IHD=ischaemic heart disease. RSDR=risk-standardised death rate. CKD=chronic kidney disease. COPD=chronic obstructive pulmonary disease.

As applied in this analysis, the UHC effective coverage measurement framework involves 30 unique cells from a matrix of five health service types—promotion, prevention, treatment, rehabilitation, and palliation—against five population-age groups (reproductive and newborn, children younger than 5 years, children and adolescents aged 5–19 years, adults aged 20–64 years, and older adults aged ≥65 years). Treatment is subdivided into two separate groups: first, communicable diseases and maternal, newborn, and child health; and second, non-communicable diseases. Effective coverage indicators were then mapped to these cells to represent needed health services across the life course.

23 effective coverage indicators were included in the present analysis (table 1). As recognised in previous studies,^19^, ^20^, ^21^, ^22^, ^23^, ^24^, ^25^, ^26^ data for directly measuring effective intervention coverage are rarely available across health services, locations, and over time. Subsequently, we used viable proxy measures and analytical techniques to approximate effective coverage for conditions considered amenable to health care.^40^, ^41^, ^42^, ^43^ Criteria set forth by the WHO ERG guided selection of effective coverage indicators and preferred measurement approaches (appendix 1 pp 12–28).^33^ Such criteria stipulated that effective coverage indicators should be currently measurable (ie, data and methods that support indicator measurement today); reflect differences in effective health services and not factors outside the immediate scope of health systems and UHC (eg, tobacco taxation and physical infrastructure such as roads and water systems); and use indicators already encompassed within the SDGs and GPW13, or draw from data systems required for monitoring of SDGs and GPW13. Several other indicator candidates were considered from 2017 to 2019 (appendix 1 pp 12–28), but inadequate data availability, access, or quality, or a combination of these factors, impeded their inclusion in the current analysis.

Four effective coverage indicators were measures of intervention coverage and 19 were mortality-based measures to proxy access to quality of care (table 1; appendix 1 pp 30–32). For the mortality-based measures, we primarily used mortality-to-incidence ratios (MIRs) and mortality-to-prevalence ratios (MPRs) for chronic or longer-term conditions (eg, diabetes or asthma). Without better data on effective coverage, such mortality-based measures are viewed as suitable proxies,^33^, ^44^, ^45^, ^46^ providing good signals on what access to quality care should, at minimum, avert or protect against even if the onset of disease cannot be wholly prevented. The main exception was ischaemic heart disease, for which GBD input data coverage and quality on non-fatal outcomes were less robust than data on causes of death and related risks; subsequently, we used risk-standardised death rates instead of MIRs or MPRs to proxy effective coverage. As a statistical approach used in previous GBD analyses^41^, ^43^ and further described in the appendix 1 (pp 31–32), risk standardisation aims to better isolate variations in mortality associated with health-care access and quality from differences in underlying risk exposures mainly related to factors outside the health system.

Effective coverage indicators for intervention coverage were kept on their natural scale (0–100%), whereas the 19 other effective coverage indicators were transformed to values on a 0–100 scale (appendix pp 31–33). Across locations and from 1990 to 2019, 0 was set by values at the 97·5th percentile or higher (ie, “worst” levels of MIRs) and 100 by the 2·5th percentile or lower (ie, “best” levels of MIRs).

#### Construction of UHC effective coverage index

As outlined by previous work,^14^, ^15^, ^16^, ^17^ population-level measures of effective coverage should represent the fraction of total health gains a health system could potentially provide, given currently available interventions, that a health system actually delivers. This construct is thus grounded in the principle of comparability—all health systems ought to maximise potential health gains for their populations—but also requires accounting for local health needs and epidemiological profiles. For instance, if a country currently experiences a high burden of diabetes and a comparatively lower burden of HIV, at least equal or even higher priority in expanding services for diabetes should occur relative to HIV in order to further support health gains.

To construct the UHC effective coverage index, we weighted each effective coverage indicator relative to their health gain weights, a metric approximating the population health gains potentially deliverable by health systems for each location-year. More detail is provided in the appendix 1 (pp 32–35), but in brief, calculations were based on three inputs for each effective coverage indicator and corresponding population-age group: estimates on the 0–100 scale, targeted disease burden, and effectiveness categories of associated interventions or services (table 1). For effectiveness, incremental values were assumed by category (ie, 90% effectiveness for category 1, 70% for category 2, 50% for category 3, and so on), as informed by studies published in the Cochrane Database of Systematic Reviews, the Tufts Cost-Effectiveness Analysis Registry and Global Health Cost-Effectiveness Analysis Registry, and Disease Control Priorities, third edition (DCP3); sensitivity analyses on shifting each effective coverage indicator by one category (ie, moving each category 2 indicator up to category 1 and then down to category 3) showed high correlations with current assignments (appendix 1 p 35).

**Figure 2:**
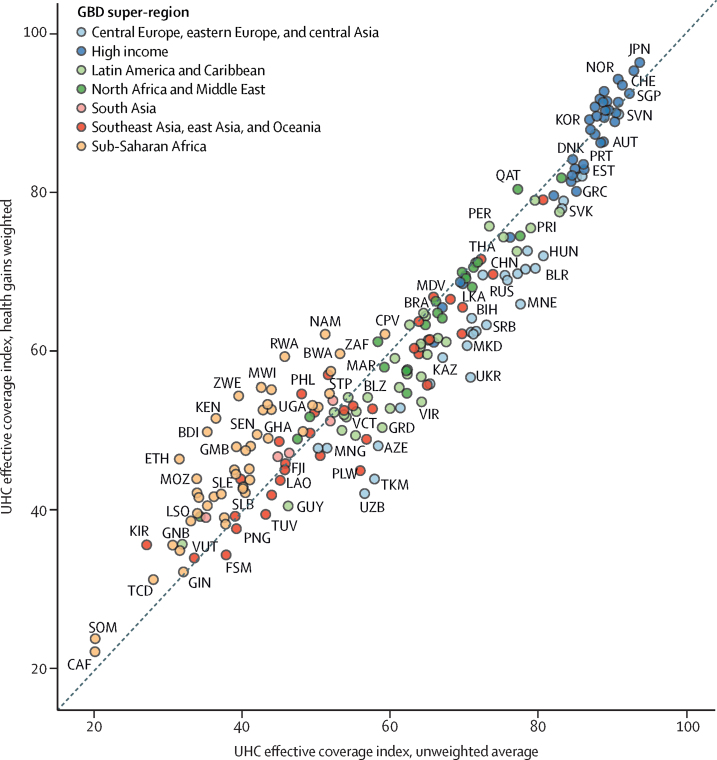
Comparing the UHC effective coverage index in 2019 with health gains weighting to the unweighted index (unweighted average of effective coverage indicators) in 2019 Locations are colour-coded by GBD super-region, and are abbreviated according to their ISO3 codes. ISO3 codes and corresponding location names are listed in appendix 1 (pp 64–68). UHC=universal health coverage. GBD=Global Burden of Diseases, Injuries, and Risk Factors Study.

As shown in figure 2, UHC effective coverage index estimates based on health gain weighting and an unweighted average across effective coverage indicators were positively associated (*r*=0·95); however, effects differed across countries.

### Validation

Since no gold-standard measures of UHC service coverage currently exist, we used three types of validity testing to compare UHC effective coverage index performance to previously published multi-country indices of UHC service coverage: the WHO UHC service coverage index for 2017;^21^ UHC service coverage index from GBD 2017;^26^ and service coverage index values from the World Bank.^24^ Further details of these analyses are provided in the appendix 1 (pp 38–52), with results summarised in table 2.

**Table 2:** Results for content, known-groups, and construct validity across multi-country health service indices for UHC service coverage measurement

	**Source**	**Content validity (proportion of cells covered)**	**Known-groups validity (proportion of 16 country pairs)**		**Convergent validity (variation of HALE explained, accounting for SDI)**		
			Based on mean values	With uncertainty	Beta coefficient	Standard error	R^2^
UHC effective coverage index, health gains weighted (reported 2019)	GBD 2019	40%	94%	63%	5·00	1·72	0·073
UHC effective coverage index, unweighted average (reported 2019)	GBD 2019	40%	94%	56%	4·19	1·49	0·068
UHC service coverage index for SDGs (reported 2017)	GBD 2017	33%	94%	69%	4·30	1·76	0·053
UHC service coverage index for SDG indicator 3.8.1 (reported 2017)	WHO 2019	20%	75%	..	4·21	1·88	0·044
Service coverage index (for most recent year reported)	World Bank 2020	17%	56%	..	1·24	1·18	0·010

Content validity was evaluated on the basis of the percentage of 30 matrix cells of health service types against population-age groups covered by each index. Known-groups validity was evaluated on the basis of the percentage of 16 country pairs correctly ranked based on country A's UHC or health-system performance being recognised as better than country B's performance; details are found in appendix 1 (pp 45–47). Convergent validity was evaluated on the basis of how much index performance could explain variation in HALE after controlling for levels of sociodemographic development (as measured by SDI). UHC=Universal health coverage. HALE=healthy life expectancy. SDI=Socio-demographic Index. GBD=Global Burden of Diseases, Injuries, and Risk Factors Study. SDGs=UN Sustainable Development Goals.

For content validity, we computed the percentage of 30 cells (ie, combinations of health services and population-age groups) from the UHC effective coverage framework that were represented by indicators for each index. For known-groups validity, we assessed how well each index could discriminate between 16 country-pairs for which previous studies show “country A” as having better performance or progress on UHC service coverage than a similar “country B”.^11^, ^23^, ^47^, ^48^, ^49^, ^50^, ^51^, ^52^, ^53^, ^54^, ^55^ These pairs were selected a priori, and for each index we calculated the fraction of pairs correctly ordered on the basis of mean estimates and accounting for uncertainty where available. For convergent validity, we quantified how much variation in healthy life expectancy could be explained by each index after removing the average relationship between each index and overall sociodemographic development (as measured by Socio-demographic Index [SDI]). In general, the UHC effective coverage index based on health gain weights showed stronger performance across these three validity measures than previous UHC service coverage measures and the unweighted UHC effective coverage index (table 2; appendix 1 pp 38–52).

### Relationship between health spending and UHC effective coverage

To better understand potential drivers of UHC effective coverage, we used stochastic frontier metaregression to quantify UHC effective coverage frontiers—estimated maximum levels of UHC effective coverage index achieved given any amount of health spending per capita—and compared country-level UHC effective coverage performance relative to these frontiers. The magnitude of these gaps between the frontier and UHC effective coverage index values provides insights into potential inefficiencies, as well as measurement error, in translating health spending into improved UHC effective coverage at the population level. Further analytical details are in the appendix 1 (pp 53–59).

Since UHC aims to minimise financial hardship associated with receiving essential health services, we focused on assessing the relationship between pooled health spending per capita (ie, government spending, prepaid private health spending, and development assistance for health)^56^ and UHC effective coverage performance. Alternative analyses, wherein out-of-pocket spending was included (ie, total health expenditure) and then development assistance for health was excluded (ie, pooled domestic health expenditures), were also done but are not reported here (appendix 2 pp 6–7).

### Counting population equivalents with UHC effective coverage

Spurred by the GPW13 UHC billion target,^6^ which calls for 1 billion more people benefiting from UHC by 2023, various approaches have been considered for translating performance metrics into the number of people covered by health services.^20^, ^21^, ^57^, ^58^ For this analysis, we used a similar approach currently recommended by WHO:^58^ we applied index estimates as fractional metrics and multiplied these values by populations to approximate population equivalents with UHC effective coverage.

To assess UHC effective coverage trajectories and their contributions towards meeting the UHC 1 billion target, we first projected country-level UHC effective coverage index estimates through to 2023. These projections were based on stochastic frontier metaregression modelled relationships between UHC effective coverage index and total health spending per capita; a related method has been used previously by GBD^26^, ^59^ and is described further in the appendix 1 (pp 60–61). Taking UHC effective coverage index as a fraction, we multiplied these values by country-level GBD-based population forecasts through to 2023.^60^ Last, we aggregated these estimates globally and by GBD super-region, and calculated additional population equivalents with UHC effective coverage from 2018 (the GPW13 baseline) to 2023.

### Uncertainty analysis

GBD aims to propagate sources of uncertainty through its estimation process,^34^, ^35^, ^36^ resulting in 1000 draws from the posterior distribution for each measure by location, age, sex, and year. We incorporated uncertainty quantified for each effective coverage indicator and associated disease burden based on GBD 2019 estimates, and did scaling, index construction, and UHC effective coverage index projections at the draw-level to reflect uncertainty. We report 95% uncertainty intervals (95% UIs) based on the ordinal 25th and 975th draws for each measure.

### Role of the funding source

The funder of the study had no role in study design, data collection, data analysis, data interpretation, or writing of the report. The corresponding author had full access to all the data in the study and had final responsibility for the decision to submit for publication.

## Results

### National UHC effective coverage patterns in 2019

**Figure 3:**
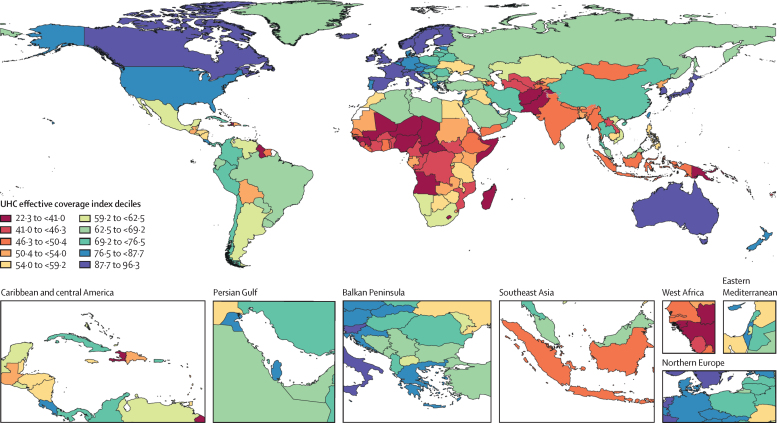
Map of the UHC effective coverage index, by decile, in 2019 Deciles are based on the distribution of UHC effective coverage index values in 2019. UHC=universal health coverage.

In 2019, UHC effective coverage performance showed some strong geographical patterns (figure 3), but sizeable heterogeneities also emerged. Various European countries, including Iceland, as well as Australia, Canada, Japan, Singapore, and South Korea, comprised the highest decile, followed by a more geographically diverse group in the ninth decile (eg, Costa Rica, Israel, New Zealand, Portugal, and the USA). Sub-Saharan Africa had among the widest range of UHC effective coverage performances in 2019, with two countries ranking in the sixth decile (Rwanda and South Africa) and 11 countries in the first decile; the countries in the first decile were mainly in western or central sub-Saharan Africa, but also spanned the continent (eg, Angola, Lesotho, Madagascar, and Somalia). Outside of sub-Saharan Africa, ten countries, including Afghanistan, Haiti, Pakistan, and Papua New Guinea, were also in the lowest decile in 2019. In east, southeast, and south Asia, countries largely fell between the eighth (China and Thailand) and second deciles (Laos), with India and Indonesia occupying the third decile. Within Latin America, various countries scored in the eighth or seventh deciles (eg, Chile, Colombia, Peru, and Brazil) but others saw UHC effective coverage index values within the fourth to fifth deciles (eg, Bolivia, Guatemala, and Nicaragua).

**Figure 4:**
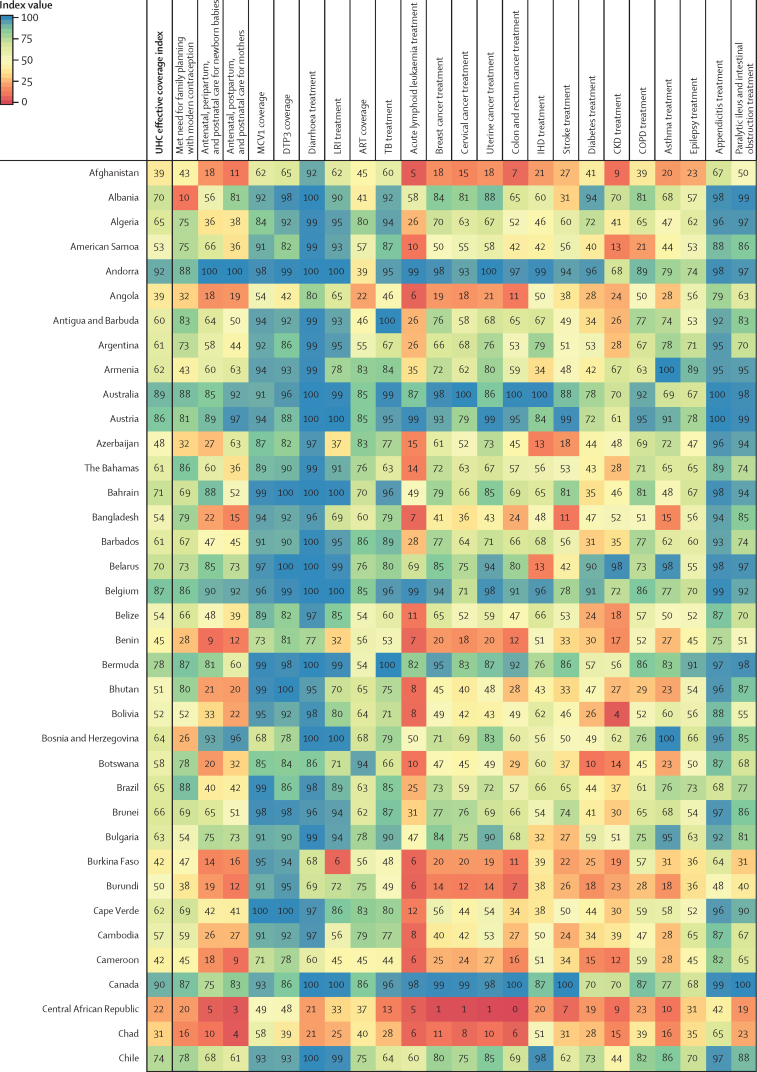

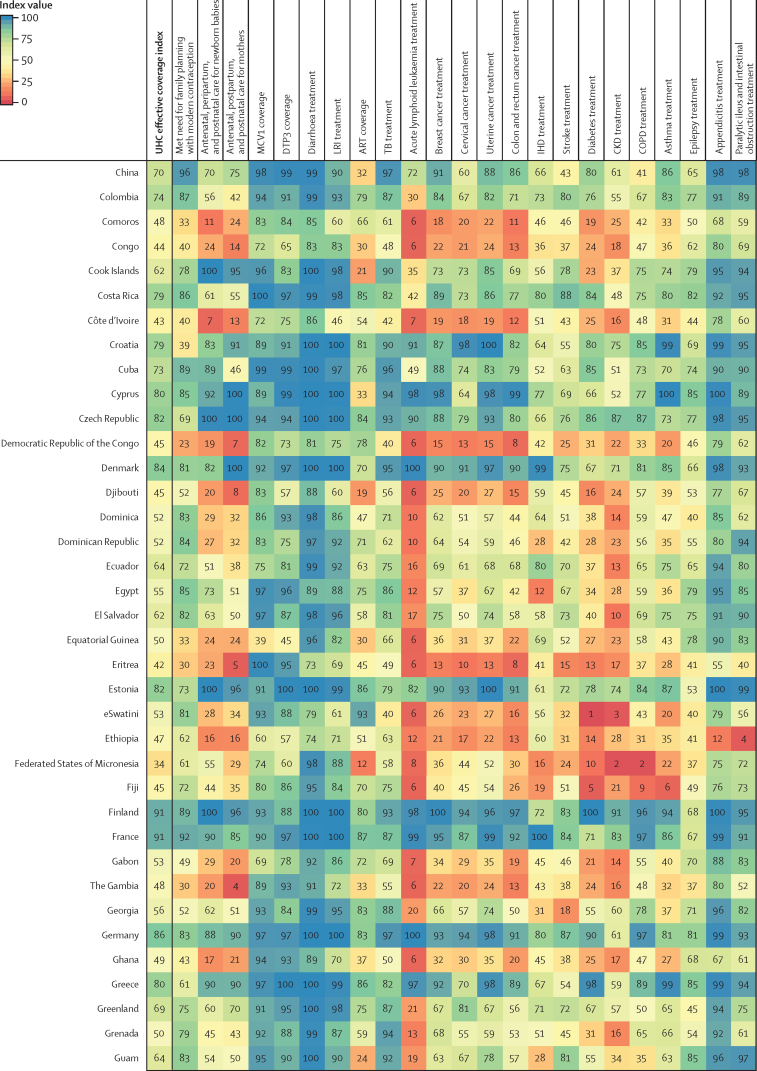

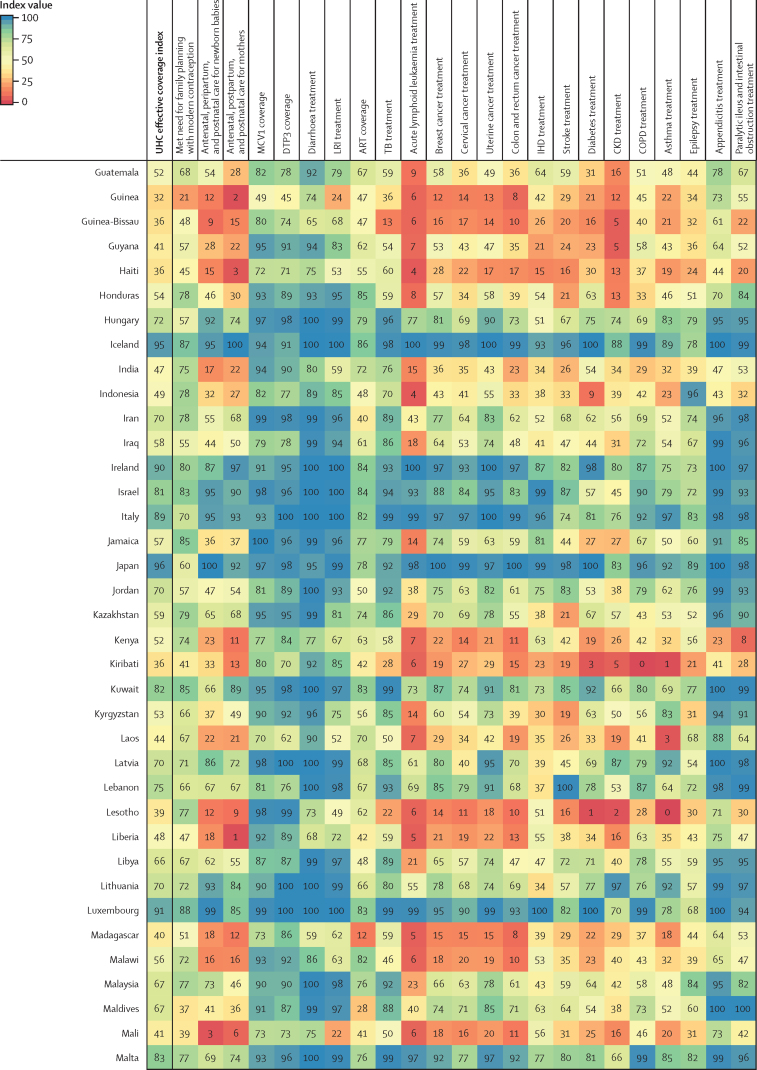

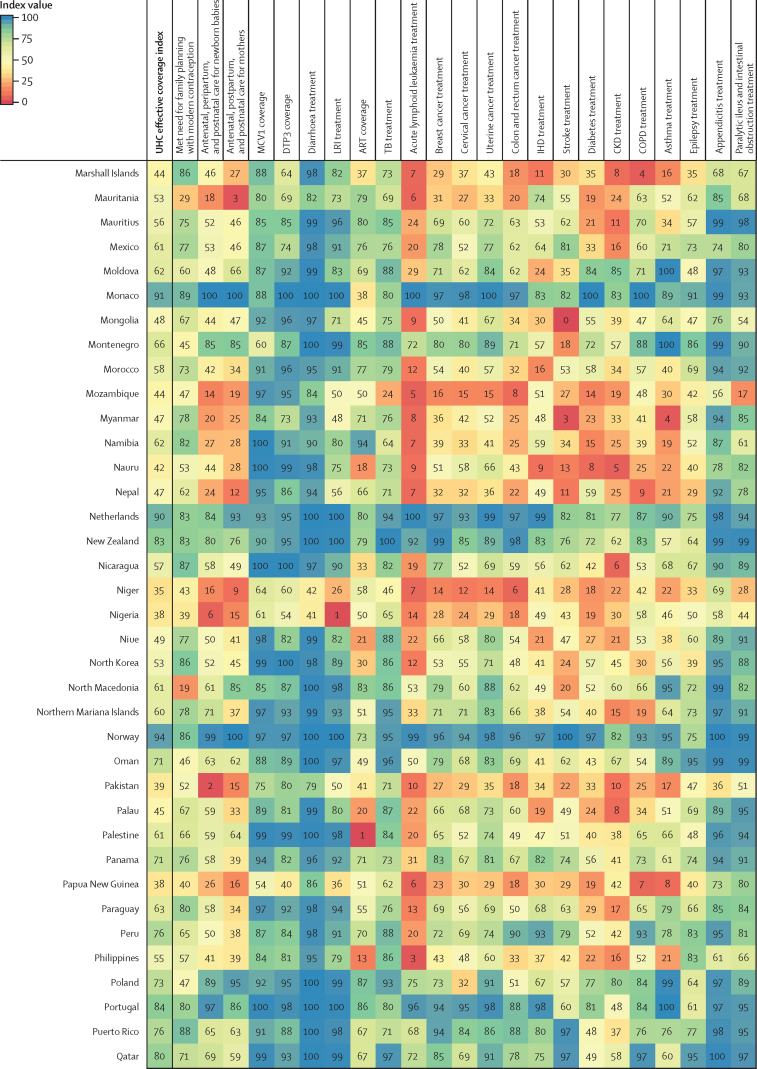

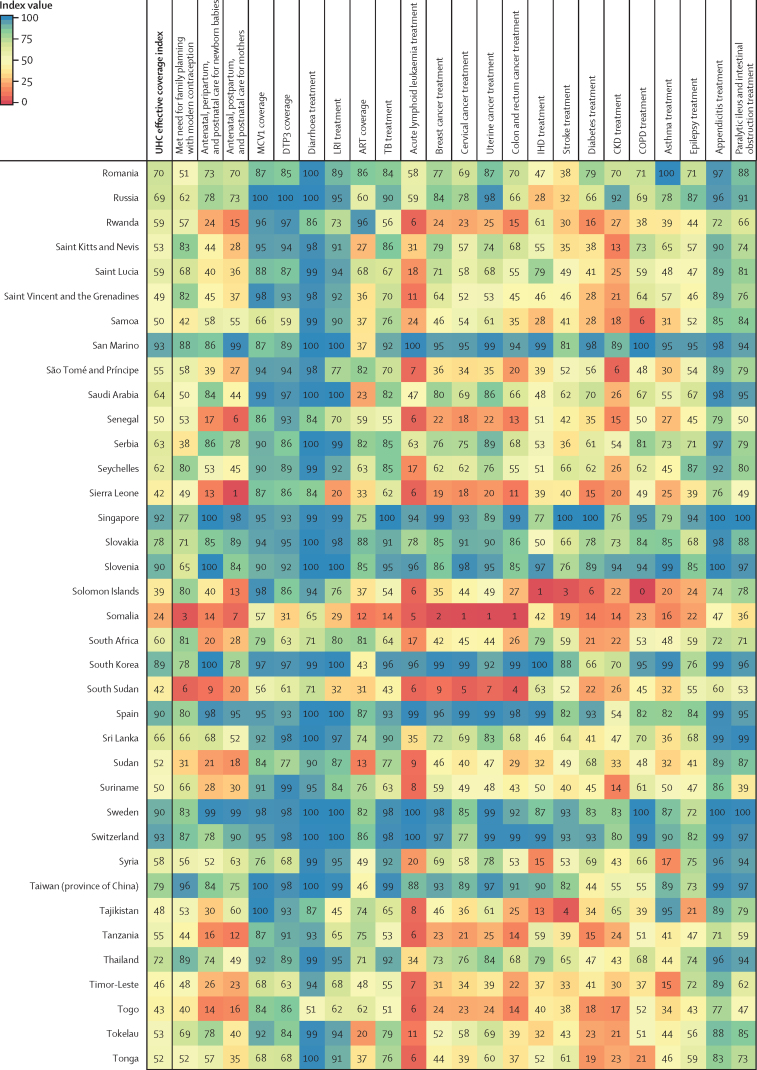

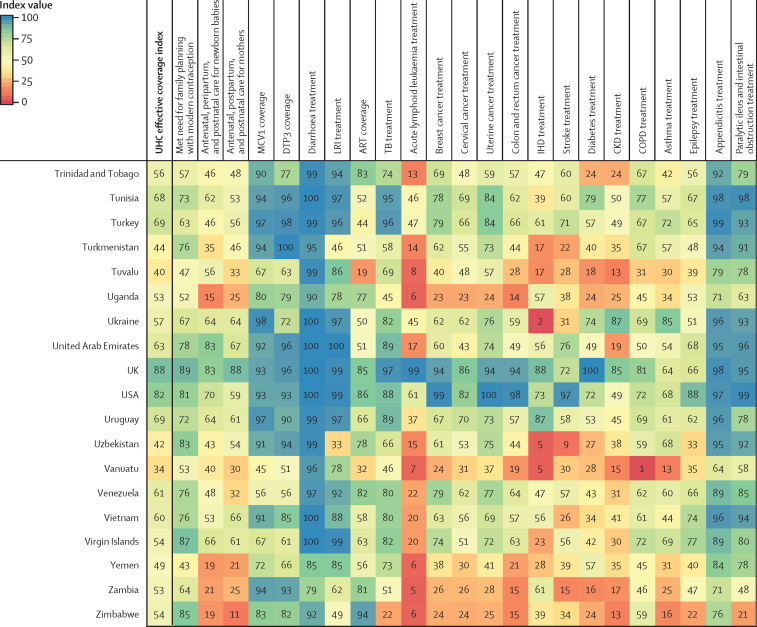
Performance on the UHC effective coverage index and 23 effective coverage indicators, by location, in 2019 Locations are reported in alphabetical order. The UHC effective coverage index and individual effective coverage indicators are reported on a scale of 0–100. Four indicators (met need for family planning, MCV1 coverage, DTP3 coverage, and ART coverage) are based on intervention coverage, whereas the remaining effective coverage indicators use measures such as mortality-to-incidence ratios to approximate access to quality care; inputs and measurement approaches for each indicator and index are further described in appendix 1 (pp 30–32). ART=antiretroviral therapy. CKD=chronic kidney disease. COPD=chronic obstructive pulmonary disease. DTP3=diphtheria, tetanus, pertussis vaccine, 3 doses. IHD=ischaemic heart disease. LRI=lower respiratory infection. MCV1=measles-containing vaccine, 1 dose. TB=tuberculosis. UHC=universal health coverage.

Performance on the overall UHC effective coverage index often corresponded with levels achieved across individual effective coverage indicators (figure 4); for instance, countries with effective coverage index values of 85 or higher generally had the vast majority of effective coverage indicators exceeding 80. Although high-performing locations usually had lower values for at least some subsets of indicators (eg, met need for family planning or antiretroviral therapy coverage), such indicators often represented areas of lower potential health gains—especially relative to effective coverage indicators proxying health services or interventions for conditions with higher potential health gains in these countries (eg, cardiovascular diseases, cancers, and diabetes). Countries and territories with fairly low overall UHC effective coverage index performance in 2019 (ie, <40) scored similarly low across most effective coverage indicators, although vaccine coverage and proxies for lower respiratory infection and diarrhoea treatment were among the main exceptions.

Many countries with middle-range performance on UHC effective coverage (ie, about 45–70) in 2019 had a mixture of fairly high values on most indicators for communicable diseases and reproductive, neonatal, maternal, and child health but comparatively lower scores on many non-communicable diseases, likely mirroring their variable epidemiological profiles and thus populations' health needs. For some countries, especially those in sub-Saharan Africa (eg, Namibia, Rwanda, and Kenya), communicable diseases (eg, HIV) and reproductive, neonatal, maternal, and child health still ranked among indicators with highest potential health gains in 2019, even though non-communicable diseases such as cardiovascular diseases and diabetes are on the rise.^35^ With their fairly high levels of coverage or services proxied by effective coverage indicators for communicable diseases and for reproductive, neonatal, maternal, and child health, several of these countries had higher UHC effective coverage index performance under a health gains weighting approach than under the assumption that each effective coverage indicator could deliver equal health gains to populations across different settings (figure 2). By contrast, in many other countries—especially those in Latin America, central and eastern Europe, and Oceania—non-communicable diseases accounted for a greater proportion of potential health gains by 2019; consequently, these countries' relatively poor performances on several effective coverage indicators proxying non-communicable disease services underpinned lower overall UHC effective coverage index values. High levels of vaccine coverage and performance on effective coverage indicators such as maternal care still contributed to UHC effective coverage performance for such countries; however, these health areas generally represented a smaller fraction of population-level health gains than many non-communicable diseases in these settings. Health gain weights, by country and territory, for each effective coverage indicator are available in the appendix 2 (pp 11–13) and via online data tools.

### Pace of progress on UHC effective coverage

**Figure 5:**
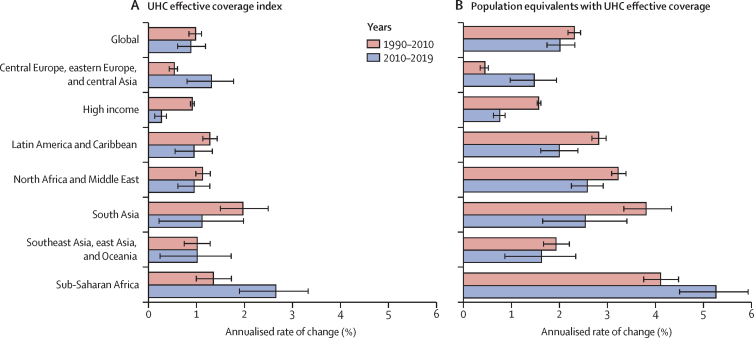
Annualised rate of change in the UHC effective coverage index (A) and population equivalents with UHC effective coverage (B), globally and by GBD super-region, 1990–2010 and 2010–2019 Values reflect the average annualised rate of change on the UHC effective coverage index and population equivalents with UHC effective coverage between each time period. Population equivalents are based on taking the UHC effective coverage index as a fraction and multiplying these values by the total population for a given location-year to approximate populations covered with UHC effective coverage. UHC=universal health coverage. GBD=Global Burden of Diseases, Injuries, and Risk Factors Study.

Since 1990, UHC effective coverage performance improved, albeit at variable rates of progress over time and across GBD super-regions (figure 5). The global average increased from 45·8 (95% UI 44·2–47·5) in 1990 to 60·3 (58·7–61·9) in 2019, while the absolute range in performance essentially remained the same (ie, 73·0-point difference in 1990 *vs* 74·0-point difference in 2019). By 2019, the UHC effective coverage index spanned from 95 or higher in Japan (96·3 [95·0–97·4]) and Iceland (95·3 [93·6–96·8]) to lower than 25 in the Central African Republic (22·3 [16·3–29·3]) and Somalia (23·9 [17·1–31·1]; appendix 2 pp 14–20). Globally, the pace of progress on UHC effective coverage was somewhat slower, albeit not significantly, from 2010 to 2019 (0·9% [0·6–1·2] annualised increase) than from 1990 to 2010 (1·0% [0·8–1·1] annualised increase). Similarly, at the global level, annualised rates of change for population equivalents with effective coverage were slightly lower from 2010 to 2019 (2·0% [1·7–2·3]) than from 1990 to 2010 (2·3% [2·2–2·4]), although this difference was not significant. However, some of these patterns diverged by GBD super-region (figure 5), as well as at the country level (appendix 2, pp 14–20). For instance, in sub-Saharan Africa, UHC effective coverage index performance improved at an average of 2·6% (1·9–3·3) per year from 2010 to 2019, surpassing its annualised rate of change from 1990 to 2010 (1·3% [1·0–1·7] average increase per year). Central Europe, eastern Europe, and central Asia also had significantly faster progress from 2010 to 2019 (1·4% [0·8–1·8] average annual increase) than from 1990 to 2010 (0·5% [0·4–0·6] annual increase).

### Relationship between health expenditure and UHC effective coverage

**Figure 6:**
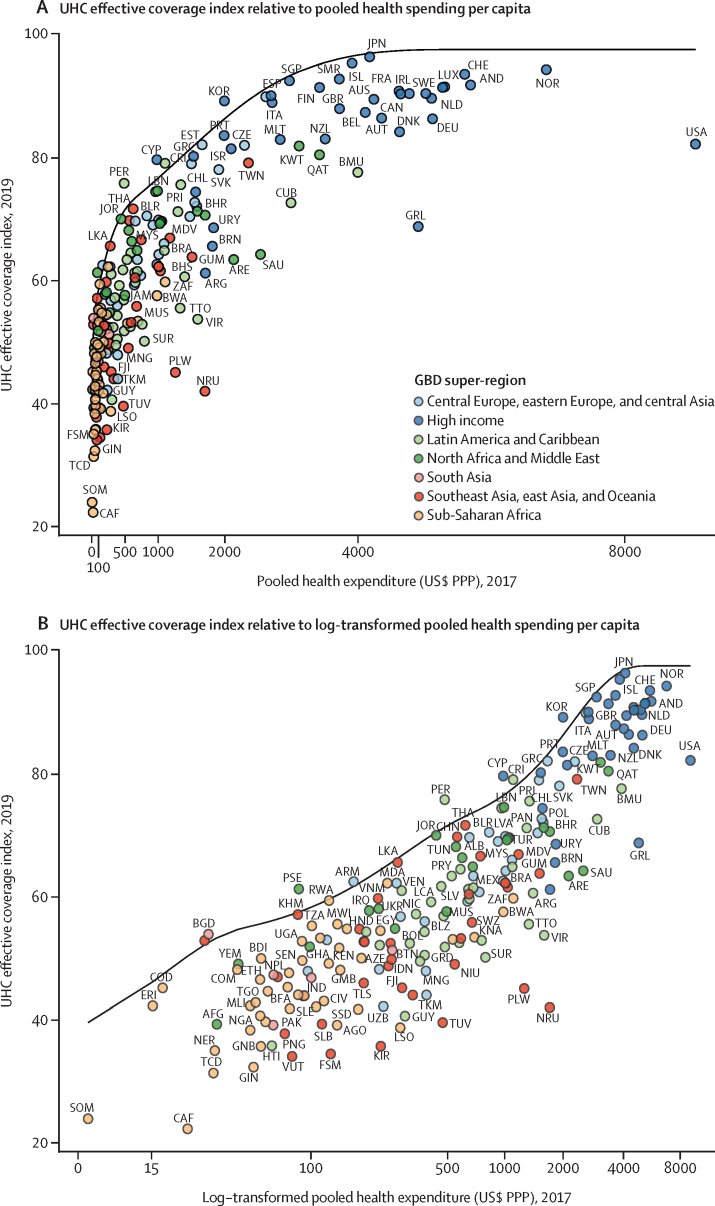
UHC effective coverage index frontier relative to pooled health spending per capita (A) and log-transformed pooled health spending per capita (B) Pooled health spending per capita includes government health expenditures, prepaid private expenditures, and development assistance for health. All health spending estimates are for 2017 measured in 2019 PPP-adjusted US$ adjusted for inflation. The black line represents the frontier values estimated for UHC effective coverage in 2019 relative to spending per capita in 2017. Locations are colour-coded by GBD super-region, with a subset abbreviated according to their ISO3 codes. ISO3 codes and corresponding location names are listed in appendix 1 (pp 64–68). UHC=universal health coverage. GBD=Global Burden of Diseases, Injuries, and Risk Factors Study. PPP=purchasing-power parity.

Country-level performance on UHC effective coverage widely varied across different levels of pooled health spending per capita (figure 6), highlighting how increased health spending is necessary but insufficient on its own to improve UHC effective coverage. Overall, the UHC effective coverage index was associated with pooled health spending per capita (*r*=0·79), but this relationship was varied at different levels of spending. Up to about $2500 (US$, adjusted for purchasing power parity) in pooled health spending per capita, increasingly higher expenditures generally paralleled higher performance on UHC effective coverage index; beyond that, higher expenditures did not correspond as consistently with further improvements in UHC effective coverage performance.

The UHC effective coverage frontier charts the highest UHC effective coverage performances, as achieved by countries in 2019, across different levels of pooled health spending per capita (figure 6); in other words, this frontier represents the relative efficiency—or inefficiency—with which countries could translate their health spending into improved UHC effective coverage. Countries including South Korea, Cyprus, Costa Rica, Peru, and Rwanda were among those setting this performance frontier at their corresponding levels of pooled health expenditure per capita. Conversely, countries across the sociodemographic spectrum (ie, Central African Republic, Lesotho, Turkmenistan, Saudi Arabia, and the USA) showed large gaps between their estimated UHC effective coverage index performances in 2019 and what could have been achievable on the UHC effective coverage frontier given these countries' levels of pooled health spending. To reach a UHC effective coverage index of at least 80, under maximum efficiency, countries would need to reach US$1398 in pooled health spending per capita (per year). Equivalent analyses and figures for total health expenditure per capita (ie, pooled health spending plus out-of-pocket spending) and pooled domestic health expenditure per capita (ie, pooled health spending minus development assistance for health) are provided in appendix 2 (pp 6–7).

### Counting population equivalents with effective coverage for the UHC billion target

Based on current projections, an estimated 5·0 billion (95% UI 4·8–5·1) population equivalents would have UHC effective coverage in 2023 (table 3). This would translate to 388·9 million (358·6–421·3) more population equivalents with UHC effective coverage over the five-year GPW13 evaluation period (2019–23, with 2018 as the baseline), or the equivalent of adding an average of 77·8 million (71·7–84·3) population equivalents per year during this time. From 2018 to 2023, sub-Saharan Africa was estimated to contribute the most additional population equivalents with UHC effective coverage (ie, 94·5 million [83·6–104·8]). By 2023, an estimated 3·1 billion (3·0–3·2) population equivalents would not have UHC effective coverage, with nearly a third residing in south Asia (ie, an estimated 968·1 million [903·5–1040·3]).

**Table 3:** Projected UHC effective coverage performance in 2023 and additional population equivalents with UHC effective coverage from 2018 to 2023, globally and by GBD super-region

	**UHC effective coverage index (95% UIs)**		Population equivalents with UHC effective coverage (95% UI)*		
	2018	2023	Added from 2018–23	Covered in 2023	Not covered in 2023
Global	59·8 (58·3 to 61·3)	61·7 (60·1 to 63·3)	388·9 (358·6 to 421·3)	5·0 billion (4·8 to 5·1)	3·1 billion (3·0 to 3·2)
Central Europe, eastern Europe, and central Asia	63·2 (61·0 to 65·5)	65·2 (62·7 to 67·6)	9·1 (7·5 to 10·9)	273·0 (262·5 to 282·8)	145·5 (135·7 to 156·1)
High income	85·8 (84·3 to 87·1)	87·1 (85·5 to 88·5)	31·6 (28·8 to 34·3)	958·3 (940·7 to 972·8)	141·5 (127·0 to 159·1)
Latin America and Caribbean	63·2 (61·1 to 65·1)	65·6 (63·3 to 67·8)	33·6 (30·8 to 36·5)	398·5 (384·7 to 412·0)	209·0 (195·6 to 222·8)
North Africa and Middle East	60·0 (57·9 to 61·9)	61·9 (59·6 to 64·0)	43·0 (39·8 to 45·9)	402·3 (387·6 to 416·1)	247·8 (233·9 to 262·5)
South Asia	46·0 (42·6 to 49·2)	48·4 (44·6 to 51·9)	88·9 (73·5 to 102·8)	909·4 (837·2 to 974·0)	968·1 (903·5 to 1040·3)
Southeast Asia, east Asia, and Oceania	64·2 (60·7 to 67·6)	66·9 (63·0 to 70·5)	88·2 (74·4 to 102·8)	1·5 billion (1·4 to 1·5)	726·3 (647·9 to 811·6)
Sub-Saharan Africa	43·9 (41·4 to 46·5)	46·2 (43·3 to 49·1)	94·5 (83·6 to 104·8)	555·6 (521·1 to 590·1)	647·1 (612·7 to 681·7)

Population equivalents based on taking the UHC effective coverage index as a fraction and multiplying these values by total population for a given location-year to approximate populations covered with UHC effective coverage. UHC=universal health coverage. GBD=Global Burden of Diseases, Injuries, and Risk Factors Study. 95% UI=95% uncertainty interval.

* Reported in millions unless otherwise indicated.

## Discussion

### Summary of the main findings

The present study offers a new approach to monitoring progress on UHC service coverage: measuring country-level effective coverage and thus better representing how well health systems are delivering health gains relative to their populations' health needs. Amid global advances on the UHC effective coverage index since 1990, our findings show a gap of more than 70 points between locations with the highest and lowest levels of UHC effective coverage remained in 2019. Particularly among low-middle to middle-SDI countries, performance of effective coverage indicators for non-communicable diseases was far lower than levels reached for several communicable diseases and maternal and child health indicators—a pattern suggesting that many countries' health systems and financing priorities are not moving as quickly as their epidemiological and demographic transitions. Higher pooled health spending per capita generally corresponded with higher UHC effective coverage. Nonetheless, country-level performance varied widely and many countries fell well below levels achieved by other countries with similar amounts of health expenditures, emphasising the importance of increasing both health-system efficiencies and funding for UHC. To achieve at least 80 on the UHC effective coverage index, countries would need to reach $1398 pooled spending per capita—and do so under maximum efficiency. An estimated 388·9 million more population equivalents would have UHC effective coverage between 2018 and 2023, falling well short of the GPW13 target of 1 billion more people benefiting from UHC during this time. Genuinely advancing toward UHC requires prioritising—and thus monitoring—effective coverage and health systems' capacities for improving outcomes for all people throughout the world.

### Past progress, current challenges, and accelerating future gains on UHC effective coverage

By 2019, UHC effective coverage improved substantially for many countries, and for some countries the pace of progress has accelerated since 2010. This was particularly evident in sub-Saharan Africa; this GBD super-region nearly doubled its average annual improvements from 2010 to 2019 compared to 1990–2010. Such gains could be related to heightened funding—and thus prioritisation—for HIV, vaccines and childhood infectious diseases, and maternal health during the Millennium Development Goal (MDG) era.^61^, ^62^ As further illustrated by the UHC effective coverage frontier, up to about $2500 per capita, rising levels of UHC effective coverage index generally paralleled pooled health spending; this trend highlights the important role of increasing funding for UHC to jumpstart progress, particularly for countries that still have very low UHC effective coverage in 2019. Yet even at the frontier, reaching better UHC effective coverage performance requires much higher pooled health spending per year: an estimated $1398 per capita to reach 80, and then $2538 per capita to reach 90 and $3424 per capita to reach 95. At present, the only countries achieving 90 or higher on the UHC effective coverage index and such levels of pooled health spending per capita are within the high-income GBD super-region. Substantially increasing total health spending could be one avenue for elevating UHC effective coverage performance; however, many countries still have high out-of-pocket spending relative to their total spending,^56^, ^61^ which is strongly related to household catastrophic health expenditures and directly counter to improving financial risk protection within UHC. Focusing on domestic heath spending while also elevating efficiency could be another viable route; our results show that many countries would theoretically achieve much higher UHC effective coverage if they could better translate current amounts of pooled spending per capita into improved performance. How to best address such inefficiencies will markedly vary across contexts, and will require accounting for country-level differences in health-system orientations and structures, political stability and governance systems, and distribution of health resources among populations. Further examination of approaches used by countries near or at the UHC effective coverage frontier relative to their pooled health spending (eg, Rwanda, Peru, South Korea, and Costa Rica) might help identify tractable policy pathways to improved efficiency.

Poor performance on various non-communicable diseases has severely hindered progress on UHC effective coverage in many countries—a trend that is likely to only worsen until quality health services for non-communicable diseases are better prioritised by countries and development partners alike. Especially among low-middle SDI to middle-SDI countries, earlier advances on UHC effective coverage were mainly propelled by improving health services focused on communicable diseases, child health, and maternal care. As cardiovascular disease, diabetes, cancers, and other non-communicable diseases became leading causes of early death and disability, they also emerged as population health needs with the highest potential health gains—that is, where health systems could increasingly deliver the most improved outcomes via effective coverage of interventions and services. Re-orienting countries' health systems towards providing effective health services for non-communicable disease is not trivial, especially if their prior focus (and funding) had a more limited scope for the types of services provided, equipment used, and health workforce training required. However, continued inaction also has likely costs: if health systems remain too focused on health problems of the past, and fail to effectively respond to where the largest potential health gains exist today, it can be increasingly difficult to translate current levels of health spending into improved UHC effective coverage. For instance, only a few high-SDI countries (eg, Japan, Switzerland, and South Korea) averaged non-communicable disease performance equal to or higher than effective coverage indicators focused on communicable diseases and maternal and child health by 2019.^63^ Unless deliberate efforts are taken now to recalibrate health-system and funding priorities, the ability to alter current trajectories for UHC effective coverage could diminish.

To catalyse faster gains in the SDG era, WHO's GPW13 set forth its bold billion targets,^6^ with the UHC target calling for 1 billion more people benefiting from UHC by 2023, relative to 2018. Current projections have the world falling well short of this ambition, with an estimated 388·9 million (358·6–421·3) more population equivalents having UHC effective coverage by 2023. Even these estimates are likely to be optimistic, as they do not account for trends in financial risk protection—the other key dimension of UHC—nor do they explicitly account for populations' needs for multiple health services. Nonetheless, this initial assessment offers important considerations for the remaining years of GPW13 and then through to 2030. With more than 3 billion population equivalents estimated to lack UHC effective coverage in 2023, targeting populous regions or countries that currently have low UHC effective coverage and investing in service expansion could be one option to accelerating future progress. For instance, south Asia, in combination with southeast Asia, east Asia, and Oceania, was estimated to have nearly 1·7 billion population equivalents without UHC effective coverage in 2023. However, on the basis of current levels of health spending, many countries in these regions already fell below their potential UHC effective coverage performance in 2019. For most countries, heightened health spending alone is unlikely to deliver on ambitious UHC targets; rather, a combination of improving alignment of health systems with population health needs and bolstering efficiencies is likely to chart faster and perhaps more sustained gains.

### Current challenges and future directions for measuring UHC effective coverage

Our measurement framework is grounded in the construct of effective coverage at the health-system level,^16^ aiming to represent a country's ability to improve health outcomes in accordance with the health needs and disease burden of its population. From this perspective, effective coverage should capture the fraction of potential population-level health gains actually delivered by the health system, relative to what the health system could have provided at maximum performance of current interventions or services. As such, we used health gain weights to construct the overall UHC effective coverage index and to more heavily weight effective coverage indicators for which a given country's health could produce greater health gains through available interventions. By contrast, the unweighted average of effective coverage indicators implies equal potential health gains irrespective of a country's epidemiological profile or effectiveness of the associated interventions or services, or a combination of both. Equally weighting interventions and their potential for improving health is directly counter to the reality of UHC programmes, which are subject to each country's unique health-system structures, political demands, and health priorities. To capture what can—or should—be achievable through health systems' provision of effective services, we believe the health gains weighting approach can better track country-led UHC investments and policy implementation. Going forward, assessments of UHC effective coverage should strive to apply this method beyond the national level, aiming to capture inequalities in potential health gains not only by location but also within population-age groups, by sex, and across other important sociodemographic dimensions (eg, race/ethnicity and migrant status).

Routinely measuring UHC effective coverage requires the existence and maintenance of several functional data systems. Many, if not most, indicators or data systems, or both, that are needed to measure effective coverage indicators are already encompassed within the health-related SDGs, which UN member states have committed to monitoring. These include functional vital registration systems that accurately record causes of death; periodic household surveys that include biomarker data and information on intervention coverage; and disease incidence registries based on administrative systems and notifications for specific causes (eg, cancers and kidney disease).^64^ Deliberate investments by national governments, as well as international agencies where appropriate, are important for strengthening these data systems and identifying how they can be used together to monitor trends in effective coverage.

The UHC effective coverage index and corresponding UHC effective coverage measurement framework represent important steps towards capturing a range of needed health services across the life course; nonetheless, as underscored by its multi-year development process (appendix 1 pp 6–21), considerable gaps persist between the breadth of the original candidate effective coverage indicators and those used in the present analysis. Minimal data on rehabilitative services and palliation across countries and over time hindered their direct inclusion or the use of suitable proxy effective coverage indicators. Recent steps by WHO (ie, publishing its first world report on vision^65^ and upcoming report on hearing,^66^ and its GPW13 indicator on oral morphine availability^6^) suggest that data collection for these areas could be increasingly prioritised. A similar paucity of routinely collected data on mental health services and substance use disorder interventions precluded their use in the current UHC effective coverage index. In the future, triangulation of data sources including administrative records, health facility records, and community-based surveys might inform such measurements.^67^ Effective coverage indicators on emergency services and trauma care were also considered but ultimately excluded because of data limitations and ongoing methodological challenges (ie, appropriately isolating improvements in effective health services from advances in transportation safety).

For non-communicable diseases, we relied on outcome-based effective coverage indicators, preferring to approximate access to quality non-communicable disease care through measures such as MIRs rather than assuming that risk exposure, screening rates, or health-system inputs, or a combination of these factors, can appropriately capture effective service provision for non-communicable diseases. Many national data systems already collect data on cause-specific mortality and disease incidence or prevalence, and when analysed together they should reflect variations in access to and quality of health services and serve as good proxy measures amid imperfect data realities for non-communicable disease services. Conversely, using indicators such as non-tobacco use and non-elevated blood pressure^4^, ^19^, ^21^ or inpatient admission rates pushes the world further away from understanding improved outcomes delivered by health systems and effective service provision. If, or when, the quantity and quality of data for measuring health services for non-communicable diseases improve, we would prefer to use more direct measures of effective coverage over outcome-based proxy indicators. For instance, our ideal effective coverage indicator for diabetes treatment would be the proportion of people with diabetes on treatment and meeting specified treatment targets such as glycated haemoglobin lower than 8%. Household surveys such as the WHO STEPwise approach to surveillance (STEPS) are increasingly collecting these data, and time series estimates by location and population-age group could be easily derived if sufficient access to such microdata is possible.

In sum, the indicators included in the present study are not meant to be prescriptive; rather, our primary objective was to establish a robust, comparable measurement framework from which UHC effective coverage could be assessed across settings and inform efforts to incorporate effective coverage into UHC monitoring. Continuing to advance effective coverage measurement of UHC in the future, especially if the main alternative is adhering to past measures with known drawbacks and narrow operationalisations of health services for all populations, is strongly supported by the broader GBD study and its collaborators.

### Limitations

Our study is subject to limitations beyond those already described. First, this analysis draws from GBD 2019 estimates of outcomes, intervention coverage, and SDG indicators,^34^, ^35^, ^36^ and thus broader GBD 2019 limitations also apply to the present study (eg, availability and quality of vital registration data, model coherence between cause-specific mortality and non-fatal measures, and new modelling approaches for risk factors and related outcomes). In the case of ischaemic heart disease, for example, new data on the interplay of household air pollution, blood pressure, and ischaemic heart disease mortality resulted in implausible risk-standardised death rates for many low-SDI to low-middle SDI countries when we only accounted for joint exposures to metabolic risks considered amenable to health care.^41^, ^43^ We thus included household and outdoor air pollution in risk standardisation and plan to further examine these risk mediation pathways in the future.

Second, health gain weights were based on classifying intervention sets into five effectiveness categories, as informed by published literature provided by Cochrane, the Tufts Cost-Effectiveness Analysis Registry, and DCP3.For some effective coverage indicators, especially treatment of more chronic conditions, distilling a wide range of reported effectiveness on available interventions into a summary assessment was quite difficult. Sensitivity analyses based on shifting each indicator's categorisation up and down one group showed similar overall UHC effective coverage index values (appendix 1 p 35). Formally simulating the range of effectiveness across interventions and incorporating this uncertainty into health gains weighting is an important future avenue for measurement of the UHC effective coverage index.

Third, due to limited data quantity or quality (or both), we could not include several original candidates for effective coverage indicators (appendix 1 pp 12–28), including seven expressly recommended by the GPW13 ERG: HPV vaccination, hepatitis C treatment, effective management of hypertension and diabetes, cataract surgery, refractive error correction, and dental care.^33^ As data availability improves alongside methods for estimating these indicators across countries, we plan to test the inclusion of these indicators, and thus some country-level UHC effective coverage index values and rankings might change. Since data are generally more easily available for better-funded interventions and health areas, it is possible that our current estimates of UHC effective coverage are overly optimistic.

Fourth, we excluded several effective coverage indicators for which high potential health gains could only be achieved in select locations because of local exposures (ie, malaria and neglected tropical diseases) or current introduction status (eg, pneumococcal conjugate vaccine [PCV]). Subsequently, our results might under-estimate UHC effective coverage in some locations (eg, countries with high coverage of effective malaria interventions) or over-estimate performance in others (eg, countries that have a high pneumonia burden but have yet to introduce PCV). Additional methodological testing is needed to better incorporate these locally relevant intervention needs within a global measurement framework.

Fifth, we did not explicitly account for the effects of potential community-level interventions and their contribution to potential health gains (eg, herd immunity garnered from very high coverage of MCV1 or DTP3). Future work should consider whether or how such effects can be incorporated into this measurement framework, particularly given the toll of recent measles outbreaks worldwide.^68^

Sixth, results of our known-groups validity testing might have varied if more or different country-pairs were selected (appendix 1 pp 45–47). Showing performance based on country means and uncertainty underscores the need to further strengthen data collection and overarching measurement for UHC effective coverage at the country level. Furthermore, it stresses the importance of estimating and reporting uncertainty in monitoring UHC, a limitation of current WHO and World Bank service coverage indices.

Seventh, approximating populations with UHC effective coverage by assuming the UHC effective coverage index as a fractional metric and multiplying by population does not account for multimorbidities, nor does it represent the distribution of needed services received within a given population. Measuring UHC effective coverage at increasing granularity (ie, subnational locations and by disaggregated age groups or sex, or both) could help improve our understanding of the distribution of health services within a given population.

### Conclusion

This study provides a new measurement framework and metric on UHC effective coverage, supporting country and global stakeholders in their efforts to track improved performance over time. By striving to capture potential health gains delivered by health systems, we hope to better diagnose and address challenges that otherwise impede the ultimate objective of UHC: improving health for all people and leaving no one behind. If current trends hold, the world will fall short of delivering on its UHC ambitions for the GPW13 and SDGs. Although this outcome is not yet inevitable, the window for meaningful action and health-system changes is rapidly narrowing. By focusing on UHC effective coverage and populations' health needs across the lifespan, we strengthen the evidence base for bringing UHC closer to reality for all.

## Data sharing

To download the data used in these analyses, please visit the Global Health Data Exchange at http://ghdx.healthdata.org/gbd-2019.
