# Supplementary material for: Measuring universal health coverage based on an index of effective coverage of health services in 204 countries and territories, 1990–2019: a systematic analysis for the Global Burden of Disease Study 2019
Source: Lancet. 2020 Oct 17;396(10258):1250–84. doi: 10.1016/S0140-6736(20)30750-9 (PMC7562819; doi:10.1016/S0140-6736(20)30750-9)
Supplement: Supplementary appendix 1 [file mmc1.pdf]

THE LANCET

## Supplementary appendix

This appendix formed part of the original submission and has been peer reviewed. We post it as supplied by the authors.

Supplement to: GBD 2019 Universal Health Coverage Collaborators. Measuring universal health coverage based on an index of effective coverage of health services in 204 countries and territories, 1990–2019: a systematic analysis for the Global Burden of Disease Study 2019. *Lancet* 2020; published online Aug 27. http://dx.doi.org/10.1016/S0140-6736(20)30750-9.

# Methods appendix to Measuring universal health coverage based on an index of effective coverage of health services in 204 countries and territories, 1990–2019: a systematic analysis for the Global Burden of Disease Study 2019

This appendix provides further methodological detail on measuring UHC effective coverage. The appendix is organized into broad sections following the structure of the main paper.

# Table of contents

Preamble ....................................................................................................3
GATHER statement.................................................................................................. 4
Author contributions....................................................................................................5
Part 1. UHC effective coverage measurement development process ...........................12

Section 1. Overview ....................................................................................12
Section 2. Background on UHC measurement considerations in the SDG era ..........12
Section 3. UHC effective coverage framework development and consultation .........14
Section 4. UHC effective coverage indicator selection .............................................18
References ....................................................................................................25

Part 2. UHC effective coverage measurement............................................................29

Section 1. Overview ....................................................................................29
Section 2. Estimating effective coverage indicators..................................................30
Section 3. Calculating health gain weights............................................................. 32
Section 4. Constructing the UHC effective coverage index .....................................35
Section 5. Sensitivity analyses ....................................................................................35
Section 6. Uncertainty analysis ....................................................................................36
References ....................................................................................................36

Part 3. Validation......................................................................................................38

Section 1. Overview ....................................................................................38
Section 2. Content validity ....................................................................................38
Section 3. Known-groups validity ............................................................................39
Section 4. Convergent validity ....................................................................................40
References ....................................................................................................41
Tables and figures.........................................................................................................43

Part 4. Frontier Analysis.............................................................................................53

Section 1. Overview ....................................................................................53
Section 2. Stochastic frontier meta-analysis............................................................53
References ....................................................................................................58

Part 5. Projections for UHC effective coverage ........................................................60

Section 1. Overview ....................................................................................60
Section 2. Forecasting steps ....................................................................................60
Section 3. Meta-stochastic frontier analysis .........................................................60
References ....................................................................................................61

Part 6. Online tools and glossary of terms...................................................................62

Online tools ....................................................................................................62
List of abbreviations....................................................................................................62

Part 7. GATHER checklist ..........................................................................................69

References ....................................................................................................72

## Preamble

This appendix provides methodological detail for estimating effective coverage to inform universal health coverage (UHC) service coverage monitoring. This study complies with the Guidelines for Accurate and Transparent Health Estimates Reporting (GATHER) recommendations.[1] It includes detailed information on our analytical steps in an effort to maximise transparency in estimation processes.

Some of the methods outlined in this appendix have been described further in other GBD publications.[2–5] Portions of Part 4 in this appendix have been directly reproduced from a pre-print manuscript by Peng and colleagues[6] in order to preserve the accuracy of model documentation.

The accompanying results appendix provides supplementary figures and tables.

## GATHER statement

This study complies with the Guidelines for Accurate and Transparent Health Estimates Reporting (GATHER) recommendations.[1] We have documented the steps involved in our analytical procedures and detailed the data sources used in compliance with GATHER. For additional GATHER reporting, please refer to the GATHER table at the end of this appendix.

# Author contributions

## Managing the estimation process

Rafael Lozano, Celine Barthelemy, Stephen S Lim, and Christopher J L Murray.

## Writing the first draft of the manuscript

Rafael Lozano, Nancy Fullman, John Everett Mumford, Megan Knight, Celine Barthelemy, and Christopher J L Murray.

## Providing data or critical feedback on data sources

Rafael Lozano, Nancy Fullman, Dash A P, Cristiana Abbafati, Hedayat Abbastabar, Hassan Abolhassani, Akine Abosetugn, Lucas Guimarães Abreu, Michael RM Abrigo, Abdelrahman Ibrahim Abushouk, Oladimeji Adebayo, Victor Adekanmbi, Olatunji Adetokunboh, Shailesh Advani, Gina Agarwal, Fares Alahdab, Khurshid Alam, Muhammad Ali, Saqib Ali, Syed Aljunid, François Alla, Amir Almasi-Hashiani, Nelson Alvis-Guzman, Nelson J Alvis-Zakzuk, Saeed Amini, Sofia Androudi, Fereshteh Ansari, Davood Anvari, Razique Anwer, Jalal Arabloo, Morteza Arab-Zozani, Olatunde Aremu, Johan Ärnlöv, Marcel Ausloos, Beatriz Paulina Ayala Quintanilla, Martin Ayanore, Samad Azari, Shelly Balassyano, Ahmed Bali, Maciej Banach, Palash Chandra Banik, Simachew Bante, Till Bärnighausen, Lope Barrero, Sanjay Basu, Vo Bay, Ettore Beghi, Masoud Behzadifar, Adam Berman, Eduardo Bernabe, Akshaya Bhagavathula, Boris Bikbov, Antonio Biondi, Archith Boloor, Sharath Burugina Nagaraja, Reinhard Busse, Luis Cámera, Juan Jesus Carrero, Deborah Carvalho Malta, Joao Mauricio Castaldelli-Maia, Carlos Castañeda-Orjuela, Franz Castro, Ferrán Catalá-López, Vijay Kumar Chattu, Dinh Toi Chu, Massimo Cirillo, Haley Comfort, Kelly Compton, Traian Vasile Constantin, Vera M Costa, Ewerton Cousin, Giovanni Damiani, Lalit Dandona, Aso Darwesh, Ahmad Daryani, Nikolaos Dervenis, Rupak Desai, Samath Dharmaratne, Govinda Dhungana, Diana Dias Da Silva, Fariba Dorostkar, Leila Doshmangir, Bruce Duncan, Arielle Eagan, David Edvardsson, Iman El Sayed, Islam Elgendy, Iqbal Elyazar, Khalil Eskandari, Saman Esmaeilnejad, Alireza Esteghamati, Mohammad Farahmand, Emerito Jose Faraon, Carla Farinha, Andrea Farioli, Andre Faro, Valery Feigin, Irina Filip, Morenike Oluwatoyin Folayan, Artem Fomenkov, Weijia Fu, Takeshi Fukumoto, Mohamed Gad, Abhay Gaidhane, Emmanuela Gakidou, Leake Gebremeskel, Hailay Gesesew, Mansour Ghafourifard, Shabnam Ghasemyani, Ahmad Ghashghaee, Nermin Ghith, Asadollah Gholamian, Syed Amir Gilani, Giorgia Giussani, Myron Anthony Godinho, Salime Goharinezhad, Bárbara Goulart, Teklemariam Gultie, Yuming Guo, Nima Hafezi-Nejad, Sajid Hameed, Chieh Han, Hannah Han, Demelash Woldeyohannes Handiso, Josep Maria Haro, Shoaib Hassan, Soheil Hassanipour, Reza Heidari-Soureshjani, Delia Hendrie, Claudiu Herteliu, Thomas Hird, Hung Chak Ho, Praveen Hoogar, Nobuyuki Horita, Mehdi Hosseinzadeh, Mowafah Househ, Guoqing Hu, Nayu Ikeda, Leeberk Raja Inbaraj, Usman Iqbal, Chidozie Declan Iwu, Morteza Jafarinia, Mohammad Ali Jahani, Nader Jahanmehr, Mihajlo Jakovljevic, Tahereh Javaheri, Ahamarshan Jayaraman Nagarajan, Achala Jayatilleke, John Ji, Peng Jia, Jost B Jonas, Farahnaz Joukar, Jacek Jozwiak, Mikk Jürisson, Zubair Kabir, Leila R Kalankesh, Rohollah Kalhor, Tanuj Kanchan, Seyed M Karimi, Nicholas J Kassebaum, Srinivasa Vittal Katikireddi, Gbenga Kayode, Yousef Khader, Ejaz Khan, Maseer Khan, Young-Ho Khang, Khaled Khatab, Amir Khater, Mahalaqua Nazli Khatib, Jagdish Khubchandani, Neda Kianipour, Young-Eun Kim, Yun Jin Kim, Adnan Kisa, Jonathan Kocarnik, Soewarta Kosen, Parvaiz Koul, Kewal Krishan, Manasi Kumar, Dian Kusuma, Hmwe Kyu, Getie Lake, Dharmesh Lal, Huong Lan Nguyen, Van Lansingh, Savita Lasrado, Kate Legrand, James Leigh, Shanshan Li, Lee-Ling Lim, Alton Lu, Jeadran Malagón-Rojas, Borhan Mansouri, Mohammad Ali Mansournia, Francisco Rogerlândio Martins-Melo, Ira Martopullo, Benjamin Massenburg, Manu Mathur, Colm Mcalinden, Addisu Melese, Walter Mendoza, Ritesh G Menezes, Atte Meretoja, Tuomo Meretoja, Bartosz Miazgowski, Binyam Minuye, Erkin Mirrakhimov, Maryam Mirzaei, Babak Moazen, Masoud Moghadaszadeh, Dara Mohammad, Naser Mohammad Gholi Mezerji, Abdollah Mohammadian-Hafshejani, Shafiu Mohammed, Ali Mokdad, Lorenzo Monasta, Masoud Moradi, Joana Morgado-Da-Costa, John Everett Mumford, Ghulam Mustafa, Mohsen Naghavi, Seyed Sina Naghibi Irvani, Mukhammad

David Naimzada, Javad Nazari, Ionut Negoi, Ruxandra Irina Negoi, Kiirithio Ngari, Josephine Ngunjiri, Diep Nguyen, Shuhei Nomura, Jean Jacques Noubiap, Christoph Nowak, Felix Ogbo, Andrew T Olagunju, Bolajoko Olusanya, Jacob Olusanya, Kanyin Ong, Obinna Onwujekwe, Alberto Ortiz, Sergej Ostojic, Adrian Oțoiu, Nikita Otstavnov, Stanislav Otstavnov, Simon Øverland, Mayowa Owolabi, Jagadish Rao Padubidri, Smita Pakhale, Raffaele Palladino, Adrian Pana, Songhomitra Panda-Jonas, Mona Pathak, Spencer Pease, Jeevan Pereira, David Pigott, Marina Pinheiro, Khem Pokhrel, Maarten Postma, Hadi Pourjafar, Reza Pourmirza Kalhori, Sergio Prada, Elisabetta Pupillo, Zahiruddin Quazi Syed, Amir Radfar, Alireza Rafiei, Saleem Rana, Chhabi Lal Ranabhat, Sowmya J Rao, Goura Rath, Priya Rathi, David L Rawaf, Salman Rawaf, Lal Rawal, Reza Rawassizadeh, Christian Razo, Vishnu Renjith, Andre Renzaho, Bhageerathy Reshmi, Seyed Mohammad Riahi, Jennifer Rickard, Nicholas Roberts, Leonardo Roever, Luca Ronfani, Enrico Rubagotti, Godfrey Rwegerera, Basema Saddik, ProfEhsan Sadeghi, Yahya Safari, Rajesh Sagar, Mohammad Reza Salahshoor, Hosni Salem, Marwa Salem, Abdallah M Samy, Milena Santric-Milicevic, Benn Sartorius, Arash Sarveazad, Brijesh Sathian, Deepak Saxena, Alyssa Sbarra, Maria Schmidt, David C Schwebel, Feng Sha, Amira Shaheen, Masood Ali Shaikh, Morteza Shamsizadeh, Mohd Shannawaz, Sheikh Mohammed Shariful Islam, David Shaw, Maral Shayesteh Bonyan, B Suresh Shetty, Jae Il Shin, Soraya Siabani, Diego Augusto Santos Silva, Jasvinder Singh, Valentin Skryabin, Amin Soheili, Joan B Soriano, Luisa Sorio Flor, Chandrashekhar T Sreeramareddy, Mark Stokes, Hafiz Ansar Rasul Suleria, Rafael Tabarés-Seisdedos, Degena Tadesse, Amir Taherkhani, Animut Tagele Tamiru, Frank Tanser, Cuong Tat Nguyen, Cuong Tat Nguyen, Hoa Thi Hoa, Maria Titova, Marcos Roberto Tovani-Palone, Bach Tran Xuan, Ravensara S Travillian, Christopher Troeger, Riaz Uddin, Irfan Ullah, Bhaskaran Unnikrishnan, Era Upadhyay, Olalekan A Uthman, Marco Vacante, Pascual Valdez, Tommi Vasankari, Yasser Vasseghian, Narayanaswamy Venketasubramanian, Vasiliy Vlassov, Yasir Waheed, Haidong Wang, Yuan-Pang Wang, Robert Weintraub, Jordan Weiss, Ronny Westerman, Gebremariam Woldu, Ai-Min Wu, Sarah Wulf Hanson, Yang Xie, Sanni Yaya, Jamal Yearwood, Naohiro Yonemoto, Mustafa Younis, Siddhesh Zadey, Syedsaoud Zaidi, Sojib Bin Zaman, Mikhail Zastrozhin, Yunquan Zhang, Xiu-Ju Zhao, Arash Ziapour, Bianca Zlavog, and Christopher J L Murray.

### Developing methods or computational machinery

Rafael Lozano, Nancy Fullman, Ashkan Afshin, Amir Almasi-Hashiani , Saeed Amini, Davood Anvari, Samad Azari, Till Bärnighausen, Paul Briant, Kate Causey, Kelly Cercy, Angela Chang, Ahmad Daryani, Ilse Dippenaar, Leila Doshmangir, Arielle Eagan, Khalil Eskandari, Abhay Gaidhane, William Gardner, Shabnam Ghasemyani, Ahmad Ghashghaee, Amir Hossein Goudarzian, Chieh Han, Shoaib Hassan, Mowafah Househ, Nicholas J Kassebaum, Neda Kianipour, Young-Eun Kim, Adnan Kisa, Jonathan Kocarnik, Jorge Ledesma, James Leigh, Alton Lu, Borhan Mansouri, Ira Martopullo, Claudia Mastrogiacomo, Masoud Moghadaszadeh, Ali Mokdad, John Everett Mumford, Mohsen Naghavi, Javad Nazari, Mona Pathak, Reza Pourmirza Kalhori, Zahiruddin Quazi Syed, Fakher Rahim, Chhabi Lal Ranabhat, Seyed Mohammad Riahi, Nicholas Roberts, Enrico Rubagotti, Mohammad Reza Salahshoor, Abdallah M Samy, Soraya Seedat, David Shaw, Maral Shayesteh Bonyan, Reed Sorensen, Jeffrey Stanaway, Christopher Troeger, Yasser Vasseghian, Ronny Westerman, Lauren Wilner, Biruk Wogayehu, Sarah Wulf Hanson, Vahid Yazdi-Feyzabadi, Jamal Yearwood, Syedsaoud Zaidi, Mikhail Zastrozhin, Arash Ziapour, Bianca Zlavog, and Christopher J L Murray.

### Providing critical feedback on methods or results

Rafael Lozano, Nancy Fullman, Dinesh Bhandari, Dash A P, Cristiana Abbafati, Hedayat Abbastabar, Foad Abd-Allah, Mustefa Glagn Abdilwohab, Bayisa Abdissa, Mohammad Abdollahi, Aidin Abedi, Hassan Abolhassani, Akine Abosetugn, Lucas Guimarães Abreu, Michael Rm Abrigo, Abdulaziz Abuhaimed, Abdelrahman Ibrahim Abushouk, Maryam Adabi, Oladimeji Adebayo, Victor Adekanmbi, Olatunji Adetokunboh, Davoud Adham, Shailesh Advani, Ashkan Afshin, Gina Agarwal, Pradyumna Agasthi, Seyed Mohamad Kazem Aghamir, Anurag Agrawal, Tauseef Ahmad, Fares Alahdab, Ziyad Al-Aly, Khurshid Alam, Yihun Mulugeta Alemu, Robert Alhassan, Muhammad Ali, Saqib Ali, Vahid Alipour, Syed Aljunid, Amir Almasi-Hashiani , Nihad Almasri,

Hesham Al-Mekhlafi, Abdulaziz Almulhim, Jordi Alonso, Rajaa Al-Raddadi, Khalid Altirkawi, Nelson Alvis-Guzman, Nelson J Alvis-Zakzuk, Saeed Amini, Fatemeh Amiri, Arianna Maever Amit, Dickson Amugsi, Fereshteh Ansari, Alireza Ansari-Moghaddam, Carl Abelardo Antonio, Davood Anvari, Razique Anwer, Jalal Arabloo, Morteza Arab-Zozani, Olatunde Aremu, Johan Ärnlöv, Charlie Ashbaugh, Marcel Ausloos, Beatriz Paulina Ayala Quintanilla, Getinet Ayano, Martin Ayanore, Muluken Altaye Ayza, Samad Azari, Peter Azzopardi, Ebrahim Babaee, Ashish Badiye, Mohammad Hossein Bakhshaei, Ahad Bakhtiari, Shankar Bakkannavar, Arun Balachandran, Shelly Balassyano, Ahmed Bali, Maciej Banach, Srikanta Banerjee, Palash Chandra Banik, Agegnehu Bante, Simachew Bante, Suzanne Barker-Collo, Lope Barrero, Quique Bassat, Sanjay Basu, Bernhard Baune, Vo Bay, Mohsen Bayati, Darshan Bb, Neeraj Bedi, Ettore Beghi, Masoud Behzadifar, Tariku Tesfaye Bekuma, Michelle Bell, Isabela Bensenor, Adam Berman, Eduardo Bernabe, Robert Bernstein, Akshaya Bhagavathula, Pankaj Bhardwaj, Anusha Ganapati Bhat, Krittika Bhattacharyya, Suraj Bhattarai, Zulfiqar Bhutta, Ali Bijani, Boris Bikbov, Ver Bilano, Antonio Biondi, Moses Bockarie, Hunduma Bojia, Srinivasa Rao Bolla, Archith Boloor, Oliver Brady, Dejana Braithwaite, Nikolay Briko, Sharath Burugina Nagaraja, Reinhard Busse, Zahid Butt, Florentino Luciano Caetano Dos Santos, Lucero Cahuana-Hurtado, Luis Cámera, Rosario Cárdenas, Deborah Carvalho Malta, Joao Mauricio Castaldelli-Maia, Carlos Castañeda-Orjuela, Franz Castro, Ferrán Catalá-López, Christopher Cederroth, Ester Cerin, Joht Singh Chandan, Jaykaran Charan, Vijay Kumar Chattu, Sarika Chaturvedi, Ken Chin, Daniel Cho, Jee-Young Choi, Dinh Toi Chu, Michael Chung, Liliana Ciobanu, Traian Vasile Constantin, Paolo Angelo Cortesi, Vera M Costa, Giovanni Damiani, Lalit Dandona, Jiregna Darega Gela, Aso Darwesh, Ahmad Daryani, Claudio Davila-Cervantes, Kairat Davletov, Jan-Walter De Neve, Edgar Denova-Gutiérrez, Kebede Deribe, Nikolaos Dervenis, Rupak Desai, Diana Dias Da Silva, Daniel Diaz, Fariba Dorostkar, Leila Doshmangir, Arielle Eagan, David Edvardsson, Iman El Sayed, Islam Elgendy, Iqbal Elyazar, Khalil Eskandari, Sharareh Eskandarieh, Saman Esmaeilnejad, Alireza Esteghamati, Oluchi Ezekannagha, Tamer Farag, Mohammad Farahmand, Emerito Jose Faraon, Carla Farinha, Pawan Faris, Andre Faro, Valery Feigin, Pietro Ferrara, Garumma Tolu Feyissa, Irina Filip, Florian Fischer, James Fisher, Nataliya Foigt, Morenike Oluwatoyin Folayan, Artem Fomenkov, Masoud Foroutan, Joel Francis, Weijia Fu, Takeshi Fukumoto, Mohamed Gad, Abhay Gaidhane, Natalie C Galles, Leake Gebremeskel, Hailay Gesesew, Mansour Ghafourifard, Shabnam Ghasemyani, Ahmad Ghashghaee, Nermin Ghith, Paramjit Gill, Tiffany K Gill, Giorgia Giussani, Yordanos Gizachew, Elena Gnedovskaya, Myron Anthony Godinho, Salime Goharinezhad, Sameer Gopalani, Amir Hossein Goudarzian, Bárbara Goulart, Rashid A Guled, Teklemariam Gultie, Yuming Guo, Rahul Gupta, Rajeev Gupta, Nima Hafezi-Nejad, Sajid Hameed, Samer Hamidi, Chieh Han, Demelash Woldeyohannes Handiso, Asif Hanif, Josep Maria Haro, Md Mehedi Hasan, Abdiwahab Hashi, Shoaib Hassan, Soheil Hassanipour, Rasmus Havmoeller, Simon Hay, Golnaz Heidari, Reza Heidari-Soureshjani, Delia Hendrie, Claudiu Herteliu, Thomas Hird, Hung Chak Ho, Michael Hole, Ramesh Holla, Bruce Hollingsworth, Praveen Hoogar, Kathleen Hopf, Nobuyuki Horita, Mehdi Hosseinzadeh, Mihaela Hostiuc, Sorin Hostiuc, Mowafah Househ, Vivian Chia-Rong Hsieh, Guoqing Hu, Tanvir Huda, Ayesha Humayun, Bing-Fang Hwang, Mohammed Im Gubari, Ivo Iavicoli, Segun Emmanuel Ibitoye, Olayinka Ilesanmi, Irena Ilic, Milena Ilic, Leeberk Raja Inbaraj, Usman Iqbal, Caleb Irvine, Farhad Islami, Chidozie Declan Iwu, Chinwe Juliana Iwu, Farhad Jadidi-Niaragh, Morteza Jafarinia, Nader Jahanmehr, Mihajlo Jakovljevic, Hosna Janjani, Tahereh Javaheri, Ahamarshan Jayaraman Nagarajan, Achala Jayatilleke, Ensiyeh Jenabi, Ravi Prakash Jha, Vivekanand Jha, John Ji, Peng Jia, Jost B Jonas, Farahnaz Joukar, Jacek Jozwiak, Mikk Jürisson, Zubair Kabir, Leila R Kalankesh, Rohollah Kalhor, Tanuj Kanchan, Neeti Kapoor, Behzad Karami Matin, Marina Karanikolos, Seyed M Karimi, Nicholas J Kassebaum, Srinivasa Vittal Katikireddi, Gbenga Kayode, Ali Kazemi Karyani, Peter Keiyoro, Yousef Khader, Mohammad Khammarnia, Ejaz Khan, Maseer Khan, Khaled Khatab, Mona Khater, Mahalaqua Nazli Khatib, Maryam Khayamzadeh, Neda Kianipour, Young-Eun Kim, Yun Jin Kim, Ruth Kimokoti, Yohannes Kinfu, Adnan Kisa, Katarzyna Kissimova-Skarbek, Mika Kivimaki, Jonathan Kocarnik, Stefan Kohler, Jacek Kopec, Vladimir Korshunov, Anirudh Kotlo, Ai Koyanagi, Kewal Krishan, Nuworza Kugbey, Vaman Kulkarni, Manasi Kumar, Nithin Kumar, Om Kurmi, Dian Kusuma, Hmwe Kyu, Carlo La Vecchia, Getie Lake, Dharmesh Lal,

Ratilal Lalloo, Huong Lan Nguyen, Savita Lasrado, Paolo Lauriola, Jorge Ledesma, Shaun Lee, Kate Legrand, James Leigh, Matilde Leonardi, Shanshan Li, Lee-Ling Lim, Giancarlo Logroscino, Alan D Lopez, Platon Lopukhov, Paulo Lotufo, Alton Lu, Mohammed Madadin, Phetole Mahasha, Morteza Mahmoudi, Azeem Majeed, Jeadran Malagón-Rojas, Shokofeh Maleki, Borhan Mansouri, Mohammad Ali Mansournia, Santi Martini, Francisco Rogerlândio Martins-Melo, Benjamin Massenburg, Manu Mathur, Colm Mcalinden, Martin Mckee, Carlo Eduardo Medina-Solís, Birhanu Geta Meharie, Teferi Mekonnen, Addisu Melese, Peter Memiah, Walter Mendoza, Ritesh G Menezes, Atte Meretoja, Irmina Maria Michalek, Binyam Minuye, Erkin Mirrakhimov, Maryam Mirzaei, Mehdi Mirzaei-Alavijeh, Philip Mitchell, Babak Moazen, Masoud Moghadaszadeh, Efat Mohamadi, Dara Mohammad, Yousef Mohammad, Naser Mohammad Gholi Mezerji, Abdollah Mohammadian-Hafshejani, Shafiu Mohammed, Jemal Abdu Mohammed , Ali Mokdad, Stefania Mondello, Maziar Moradi-Lakeh, Rahmatollah Moradzadeh, Joana Morgado-Da-Costa, Jonathan Mosser, Amin Mousavi Khaneghah, John Everett Mumford, Moses Muriithi, Ghulam Mustafa, Ashraf Nabhan, Mehdi Naderi, Mohsen Naghavi, Seyed Sina Naghibi Irvani, Behshad Naghshtabrizi, Mukhammad David Naimzada, Vinay Nangia, Jobert Richie Nansseu, Vinod C Nayak, Javad Nazari, Rawlance Ndejjo, Ionut Negoi, Ruxandra Irina Negoi, Subas Neupane, Kiirithio Ngari, Georges Nguefack-Tsague, Josephine Ngunjiri, Diep Nguyen, Chukwudi Nnaji, Shuhei Nomura, Ole F Norheim, Jean Jacques Noubiap, Christoph Nowak, Felix Ogbo, Onome Oghenetega, In-Hwan Oh, Emmanuel Okunga, Andrew T Olagunju, Bolajoko Olusanya, Jacob Olusanya, Mojisola Oluwasanu, Muktar Omer, Obinna Onwujekwe, Doris V Ortega-Altamirano, Alberto Ortiz, Sergej Ostojic, Adrian Oțoiu, Nikita Otstavnov, Stanislav Otstavnov, Simon Øverland, Mayowa Owolabi, Jagadish Rao Padubidri, Smita Pakhale, Raffaele Palladino, Adrian Pana, Songhomitra Panda-Jonas, Mona Pathak, George Patton, Sagun Paudel, Hamidreza Pazoki Toroudi, Amy Peden, Emmanuel Peprah, Jeevan Pereira, David Pigott, Marina Pinheiro, Meghdad Pirsaheb , Khem Pokhrel, Maarten Postma, Hadi Pourjafar, Farshad Pourmalek, Reza Pourmirza Kalhori, Akram Pourshams, Sergio Prada, Dimas Ria Angga Pribadi, Elisabetta Pupillo, Zahiruddin Quazi Syed, Amir Radfar, Ata Rafiee, Alireza Rafiei, Alberto Raggi, Fakher Rahim, Muhammad Aziz Rahman, Ali Rajabpour-Sanati, Chhabi Lal Ranabhat, Sowmya J Rao, Goura Rath, Priya Rathi, David L Rawaf, Salman Rawaf, Lal Rawal, Reza Rawassizadeh, Christian Razo, Vishnu Renjith, Andre Renzaho, Bhageerathy Reshmi, Nima Rezaei, Seyed Mohammad Riahi, Daniel Cury Ribeiro, Jennifer Rickard, Leonardo Roever, Luca Ronfani, Gholamreza Roshandel, Enrico Rubagotti, Godfrey Rwegerera, Siamak Sabour, Perminder Sachdev, Basema Saddik, Profehsan Sadeghi, Yahya Safari, Rajesh Sagar, Biniyam Sahiledengle, Mohammad Ali Sahraian, Mohammad Reza Salahshoor, Hosni Salem, Marwa Salem, Hossein  Samadi Kafil , Abdallah M Samy, Juan Sanabria, Milena Santric-Milicevic, Sivan Saraswathy, Rodrigo Sarmiento-Suárez, Benn Sartorius, Arash Sarveazad, Brijesh Sathian, Thirunavukkarasu Sathish, Davide Sattin, Miloje Savic, Deepak Saxena, Lauren Schaeffer, Silvia Schiavolin, Aletta Schutte, David C Schwebel, Falk Schwendicke, Feng Sha, Saeed Shahabi, Amira Shaheen, Masood Ali Shaikh, Morteza Shamsizadeh, Mohd Shannawaz, Kiomars Sharafi, Sheikh Mohammed Shariful Islam, David Shaw, Maral Shayesteh Bonyan, Aziz Sheikh, Abbas Sheikhtaheri, Wondimeneh Shiferaw, Mika Shigematsu, Jae Il Shin, Rahman Shiri, Km Shivakumar Shivakumar Km, Mark Shrime, Kerem Shuval, Soraya Siabani, Diego Augusto Santos Silva, Biagio Simonetti, Jasvinder Singh, Pushpendra Singh, Valentin Skryabin, Emma Smith, Amin Soheili, Shahin Soltani, Reed Sorensen, Joan B Soriano, Luisa Sorio Flor, Muluken Sorrie, Ireneous Soyiri, Chandrashekhar T Sreeramareddy, Jeffrey Stanaway, Nicholas Steel, Mark Stokes, Muawiyyah Babale Sufiyan, Hafiz Ansar Rasul Suleria, Santosh Tadakamadla, Animut Tagele Tamiru, Frank Tanser, Md Tareque, Ingan Tarigan, Cuong Tat Nguyen, Cuong Tat Nguyen, Fabrizio Tediosi, Bhaskar Thakur, Hoa Thi Hoa, Maria Titova, Marcello Tonelli, Fotis Topouzis, Marcos Roberto Tovani-Palone, Bach Tran Xuan, Ravensara S Travillian, Riaz Uddin, Irfan Ullah, Chukwuma Umeokonkwo, Bhaskaran Unnikrishnan, Era Upadhyay, Olalekan A Uthman, Marco Vacante, Pascual Valdez, Santosh Varughese, Yasser Vasseghian, Narayanaswamy Venketasubramanian, Francesco S Violante, Stein Emil Vollset, Theo Vos, Yasir Waheed, Magdalene Walters, Richard Wamai, Haidong Wang, Yuan-Pang Wang, Robert Weintraub, Jordan Weiss, Ronny Westerman, Biruk Wogayehu, Gebremariam Woldu, Charles Wolfe

Ai-Min Wu, Sarah Wulf Hanson, Yang Xie, Yuichiro Yano, Sanni Yaya, Vahid Yazdi-Feyzabadi, Jamal Yearwood, Paul Yip, Naohiro Yonemoto, Mustafa Younis, Zabihollah Yousefi, Taraneh Yousefinezhadi, Hasan Yusefzadeh, Siddhesh Zadey, Telma Zahirian Moghadam , Syedsaoud Zaidi, Leila Zaki, Sojib Bin Zaman, Maryam Zamanian, Hamed Zandian, Mikhail Zastrozhin, Kaleab Alemayehu Zewdie, Yunquan Zhang, Yingxi Zhao, Cong Zhu, Arash Ziapour, Sanjay Zodpey, and Christopher J L Murray.

### Drafting the work or revising it critically for important intellectual content

Rafael Lozano, Nancy Fullman, Cristiana Abbafati, Hedayat Abbastabar, Foad Abd-Allah, Mustefa Glagn Abdilwohab, Mohammad Abdollahi, Hassan Abolhassani, Lucas Guimarães Abreu, Abdulaziz Abuhaimed, Abdelrahman Ibrahim Abushouk, Oladimeji Adebayo, Victor Adekanmbi, Olatunji Adetokunboh, Shailesh Advani, Gina Agarwal, Pradyumna Agasthi, Rufus Akinyemi, Fares Alahdab, Khurshid Alam, Muhammad Ali, Saqib Ali, Majid Almadi, Ali Almasi, Nihad Almasri, Hesham Al-Mekhlafi, Abdulaziz Almulhim, Nelson Alvis-Guzman, Saeed Amini, Mostafa Amini-Rarani, Dickson Amugsi, Deanna Anderlini, Carl Abelardo Antonio, Ernoiz Antriyandarti, Jalal Arabloo, Morteza Arab-Zozani, Olatunde Aremu, Johan Ärnlöv, Malke Asaad, Mehran Asadi-Aliabadi, Ali Asadi-Pooya, Seyyed Shamsadin Athari, Maha Atout, Marcel Ausloos, Leticia Avila-Burgos, Beatriz Paulina Ayala Quintanilla, Martin Ayanore, Peter Azzopardi, Ashish Badiye, Atif Baig, Mohammad Hossein Bakhshaei, Shankar Bakkannavar, Arun Balachandran, Ahmed Bali, Maciej Banach, Srikanta Banerjee, Palash Chandra Banik, Agegnehu Bante, Suzanne Barker-Collo, Till Bärnighausen, Lope Barrero, Celine Barthelemy, Quique Bassat, Sanjay Basu, Bernhard Baune, Vo Bay, Darshan Bb, Neeraj Bedi, Ettore Beghi, Masoud Behzadifar, Michelle Bell, Isabela Bensenor, Adam Berman, Akshaya Bhagavathula, Dinesh Bhandari, Anusha Ganapati Bhat, Krittika Bhattacharyya, Suraj Bhattarai, Zulfiqar Bhutta, Boris Bikbov, Antonio Biondi, Moses Bockarie, Hunduma Bojia, Srinivasa Rao Bolla, Oliver Brady, Dejana Braithwaite, Andrew Briggs, Nikolay Briko, Sharath Burugina Nagaraja, Florentino Luciano Caetano Dos Santos, Lucero Cahuana-Hurtado, Giulia Carreras, Juan Jesus Carrero, Felix Carvalho, Deborah Carvalho Malta, Joao Mauricio Castaldelli-Maia, Giulio Castelpietra, Franz Castro, Ferrán Catalá-López, Christopher Cederroth, Ester Cerin, Joht Singh Chandan, Vijay Kumar Chattu, Sarika Chaturvedi, Ken Chin, Daniel Cho, Jee-Young Choi, Hanne Christensen, Dinh Toi Chu, Michael Chung, Liliana Ciobanu, Massimo Cirillo, Traian Vasile Constantin, Paolo Angelo Cortesi, Vera M Costa, Ewerton Cousin, Giovanni Damiani, Aso Darwesh, Ahmad Daryani, Gail Davey, Claudio Davila-Cervantes, Jan-Walter De Neve, Edgar Denova-Gutiérrez, Kebede Deribe, Nikolaos Dervenis, Rupak Desai, Samath Dharmaratne, Govinda Dhungana, Diana Dias Da Silva, Daniel Diaz, Bruce Duncan, Andre Duraes, Arielle Eagan, David Edvardsson, Iman El Sayed, Maha El Tantawi, Islam Elgendy, Khalil Eskandari, Sharareh Eskandarieh, Saman Esmaeilnejad, Alireza Esteghamati, Tamer Farag, Andrea Farioli, Andre Faro, Mehdi Fazlzadeh, Valery Feigin, Eduarda Fernandes, Irina Filip, Florian Fischer, James Fisher, Nataliya Foigt, Morenike Oluwatoyin Folayan, Masoud Foroutan, Joel Francis, Takeshi Fukumoto, Joao Furtado, Mohamed Gad, Abhay Gaidhane, Silvano Gallus, Gebreamlak Gebremeskel, Leake Gebremeskel, Shabnam Ghasemyani, Ahmad Ghashghaee, Nermin Ghith, Paramjit Gill, Tiffany K Gill, Mojgan Gitimoghaddam, Giorgia Giussani, Yordanos Gizachew, Myron Anthony Godinho, Sameer Gopalani, Bárbara Goulart, Rafael Guimarães, Teklemariam Gultie, Yuming Guo, Rahul Gupta, Rajeev Gupta, Nima Hafezi-Nejad, Abdul Hafiz, Teklehaimanot Haile, Randah Hamadeh, Demelash Woldeyohannes Handiso, Graeme Hankey, Josep Maria Haro, Ahmed Hasaballah, Abdiwahab Hashi, Amr Hassan, Rasmus Havmoeller, Khezar Hayat, Golnaz Heidari, Claudiu Herteliu, Thomas Hird, Michael Hole, Ramesh Holla, Bruce Hollingsworth, Praveen Hoogar, Nobuyuki Horita, Mostafa Hosseini, Mehdi Hosseinzadeh, Mihaela Hostiuc, Sorin Hostiuc, Mowafah Househ, Ivo Iavicoli, Segun Emmanuel Ibitoye, Nayu Ikeda, Olayinka Ilesanmi, Irena Ilic, Milena Ilic, Usman Iqbal, Caleb Irvine, M Mofizul Islam, Farhad Islami, Hiroyasu Iso, Chidozie Declan Iwu, Chinwe Juliana Iwu, Jalil Jaafari, Morteza Jafarinia, Mihajlo Jakovljevic, Tahereh Javaheri, Ahamarshan Jayaraman Nagarajan, Ensiyeh Jenabi, Ravi Prakash Jha, Vivekanand Jha, Peng Jia, Jost B Jonas, Jacek Jozwiak, Mikk Jürisson, Aruna Kamath, Neeti Kapoor, Marina Karanikolos, Nicholas J Kassebaum, Srinivasa Vittal Katikireddi, Gbenga Kayode, Yousef Khader, Ejaz Khan, Maseer Khan,

Young-Ho Khang, Khaled Khatab, Mona Khater, Mahalaqua Nazli Khatib, Jagdish Khubchandani, Neda Kianipour, Young-Eun Kim, Adnan Kisa, Mika Kivimaki, Cameron Kneib, Dr Sonali Kochhar, Vladimir Korshunov, Parvaiz Koul, Ai Koyanagi, Kewal Krishan, Nuworza Kugbey, Om Kurmi, Dian Kusuma, Carlo La Vecchia, Ben Lacey, Getie Lake, Ratilal Lalloo, Huong Lan Nguyen, Iván Landires, Anders Larsson, Paolo Lauriola, Jeffrey Lazarus, Paul Lee, Kate Legrand, Matilde Leonardi, Shanshan Li, Lee-Ling Lim, Platon Lopukhov, Paulo Lotufo, Alton Lu, Mohammed Madadin, Azeem Majeed, Jeadran Malagón-Rojas, Borhan Mansouri, Mohammad Ali Mansournia, Francisco Rogerlândio Martins-Melo, Benjamin Massenburg, Colm Mcalinden, Carlo Eduardo Medina-Solís, Addisu Melese, Walter Mendoza, Ritesh G Menezes, George Mensah, Atte Meretoja, Tuomo Meretoja, Tomislav Mestrovic, Irmina Maria Michalek, Binyam Minuye, Philip Mitchell, Babak Moazen, Masoud Moghadaszadeh, Dara Mohammad, Yousef Mohammad, Abdollah Mohammadian-Hafshejani, Shafiu Mohammed, Ali Mokdad, Lorenzo Monasta, Stefania Mondello, Maziar Moradi-Lakeh, Rahmatollah Moradzadeh, Paula Moraga, Joana Morgado-Da-Costa, Shane Morrison, Abbas Mosapour, Jonathan Mosser, Amin Mousavi Khaneghah, John Everett Mumford, Ghulam Mustafa, Ashraf Nabhan, Mehdi Naderi, Mohsen Naghavi, Seyed Sina Naghibi Irvani, Behshad Naghshtabrizi, Mukhammad David Naimzada, Vinay Nangia, Jobert Richie Nansseu, Vinod C Nayak, Javad Nazari, Rawlance Ndejjo, Ionut Negoi, Ruxandra Irina Negoi, Georges Nguefack-Tsague, Josephine Ngunjiri, Diep Nguyen, Ole F Norheim, Jean Jacques Noubiap, Christoph Nowak, Virginia Nunez-Samudio, Felix Ogbo, Onome Oghenetega, In-Hwan Oh, Morteza Oladnabi, Andrew T Olagunju, Bolajoko Olusanya, Jacob Olusanya, Mojisola Oluwasanu, Muktar Omer, Obinna Onwujekwe, Doris V Ortega-Altamirano, Alberto Ortiz, Sergej Ostojic, Adrian Oțoiu, Nikita Otstavnov, Stanislav Otstavnov, Simon Øverland, Mayowa Owolabi, Jagadish Rao Padubidri, Smita Pakhale, Raffaele Palladino, Adrian Pana, Songhomitra Panda-Jonas, Mona Pathak, Amy Peden, Jeevan Pereira, Thomas Pilgrim, Marina Pinheiro, Khem Pokhrel, Maarten Postma, Farshad Pourmalek, Reza Pourmirza Kalhori, Sergio Prada, Elisabetta Pupillo, Zahiruddin Quazi Syed, Amir Radfar, Ata Rafiee, Alberto Raggi, Fakher Rahim, Muhammad Aziz Rahman, Chhabi Lal Ranabhat, Sowmya J Rao, Davide Rasella, Vahid Rashedi, Goura Rath, David L Rawaf, Salman Rawaf, Lal Rawal, Reza Rawassizadeh, Vishnu Renjith, Andre Renzaho, Bhageerathy Reshmi, Nima Rezaei, Seyed Mohammad Riahi, Daniel Cury Ribeiro, Jennifer Rickard, Leonardo Roever, Michele Romoli, Luca Ronfani, Gholamreza Roshandel, Godfrey Rwegerera, Siamak Sabour, Perminder Sachdev, Basema Saddik, Masoumeh Sadeghi, Rajesh Sagar, Amirhossein Sahebkar, Mohammad Ali Sahraian, Mohammad Reza Salahshoor, Hosni Salem, Marwa Salem, Hossein Samadi Kafil , Abdallah M Samy, Juan Sanabria, Milena Santric-Milicevic, Rodrigo Sarmiento-Suárez, Arash Sarveazad, Davide Sattin, Miloje Savic, Susan Sawyer, Silvia Schiavolin, Maria Schmidt, Aletta Schutte, David C Schwebel, Falk Schwendicke, Saeed Shahabi, Sheikh Mohammed Shariful Islam, Maral Shayesteh Bonyan, Aziz Sheikh, Kenij Shibuya, Mika Shigematsu, Km Shivakumar Shivakumar Km, Mark Shrime, Kerem Shuval, Inga Dora Sigfusdottir, Rannveig Sigurvinsdottir, Diego Augusto Santos Silva, João Pedro Silva, Jasvinder Singh, Dhirendra Narain Sinha, Emma Smith, Amin Soheili, Joan B Soriano, Muluken Sorrie, Ireneous Soyiri, Chandrashekhar T Sreeramareddy, Caroline Stein, Mark Stokes, Muawiyyah Babale Sufiyan, Iyad Sultan, Rafael Tabarés-Seisdedos, Takahiro Tabuchi, Santosh Tadakamadla, Zemenu Tadesse, Cuong Tat Nguyen, Cuong Tat Nguyen, Bhaskar Thakur, Hoa Thi Hoa, Marcello Tonelli, Roman Topor-Madry, Fotis Topouzis, Marcos Roberto Tovani-Palone, Bach Tran Xuan, Ravensara S Travillian, Riaz Uddin, Irfan Ullah, Chukwuma Umeokonkwo, Bhaskaran Unnikrishnan, Era Upadhyay, Olalekan A Uthman, Marco Vacante, Tommi Vasankari, Yasser Vasseghian, Narayanaswamy Venketasubramanian, Francesco S Violante, Vasiliy Vlassov, Stein Emil Vollset, Theo Vos, Richard Wamai, Yuan-Pang Wang, Robert Weintraub, Ronny Westerman, Gebremariam Woldu, Ai-Min Wu, Hossein Yahyazadeh, Kazumasa Yamagishi, Sanni Yaya, Vahid Yazdi-Feyzabadi, Naohiro Yonemoto, Syedsaoud Zaidi, Sojib Bin Zaman, Mohammad Zamani, Maryam Zamanian, Xiu-Ju Zhao, Yingxi Zhao, Cong Zhu, Arash Ziapour, and Christopher J L Murray.

## Extracting, cleaning, or cataloging data; designing or coding figures and tables

Rafael Lozano, Nancy Fullman, Olatunji Adetokunboh, Samuel Albertson, Amir Almasi-Hashiani , Fatemeh Amiri, Shelly Balassyano, Franz Castro, Kate Causey, Haley Comfort, Giovanni Damiani, Ahmad Daryani, Ilse Dippenaar, Arielle Eagan, Khalil Eskandari, Saman Esmaeilnejad, Takeshi Fukumoto, Abhay Gaidhane, William Gardner, Leake Gebremeskel, Chieh Han, mowafah househ, Caleb Irvine, Morteza Jafarinia, Nicholas J Kassebaum, Young-Eun Kim, Adnan Kisa, Jorge Ledesma, Andrew Leever, Alton Lu, Jianing Ma, Borhan Mansouri, Fereshteh Mehri, Masoud Moghadaszadeh, Ali Mokdad, John Everett Mumford, Mohsen Naghavi, Kanyin Ong, Alyssa Pennini, Zahiruddin Quazi Syed, Chhabi Lal Ranabhat, Nima Rezaei, Seyed Mohammad Riahi, Enrico Rubagotti, Siamak Sabour, Abdallah M Samy, Fablina Sharara, Kyle Simpson, Emma Spurlock, Jeffrey Stanaway, Whitney Teagle, Christopher Troeger, Yasser Vasseghian, Avina Vongpradith, Biruk Wogayehu, Gebremariam Woldu, Sarah Wulf Hanson, Syedsaoud Zaidi, Mikhail Zastrozhin, and Christopher J L Murray.

## Managing the overall research enterprise

Rafael Lozano, Ashkan Afshin, Charlie Ashbaugh, Celine Barthelemy, Kelly Compton, Lalit Dandona, Nicholas J Kassebaum, Yohannes Kinfu, Alan D Lopez, Deborah Carvalho Malta, George Mensah, Ali Mokdad, Mohsen Naghavi, Simon Øverland, George Patton, Benn Sartorius, Roman Topor-Madry, Stein Emil Vollset, Theo Vos, Haidong Wang, Andrea Werdecker, and Christopher J L Murray.

# Part 1. UHC effective coverage measurement development process

## Section 1. Overview

Broadly, achieving "universal health coverage (UHC) means that all people receive the quality services they need without...financial hardship."[1] Making progress toward and ultimately attaining UHC for all rose to global policy prominence with the adoption of the UN Sustainable Development Goals (SDGs) in 2015,[2] wherein an explicit target – SDG 3.8 – called for achieving UHC by 2030. Two SDG indicators comprise this UHC target, one of which aims to represent service coverage (SDG 3.8.1)[3] and one of which reflects financial risk protection (SDG 3.8.2).[4]

In the next sections, we summarise the background and events leading to the development of the UHC effective coverage index (UHC ECI) (Section 2). We also summarise consultative steps on the UHC effective coverage measurement framework and indicator selection (Sections 3 and 4).

## Section 2. Background on UHC measurement considerations in the SDG era

Since the initial release of the global SDG indicator framework in 2016,[5] the measurement of each UHC component has been subject to considerable debate.[6–10] For instance, the original SDG 3.8.2 indicator, "number of people covered by health insurance or a public health system per 1,000 population," was met with such negative feedback and critique that hundreds of organisations and individuals effectively campaigned for its replacement by the Inter-agency Expert Group on SDG Indicators (IAEG-SDGs) and UN Statistical Commission in March 2017.[11] The revised 3.8.2 indicator, "proportion of population with large household expenditures on health as a share of total household expenditure or income,"[4] is viewed as a more direct measure of financial risk protection within UHC.

Measuring UHC service coverage, especially for global monitoring purposes within the SDG framework, has faced two main challenges: (1) defining what should constitute health services needed by populations; and (2) optimally quantifying receipt of these services and corresponding quality. At the global level, the definition of UHC has varied over time, as well as by group and/or resolution focused on its pursuit (examples shown in Table 1.1).[1,2,12–19] Definitional differences are most striking for the service coverage component of UHC – whether the emphasis is on access to services versus their actual use or receipt; types of services expressly referenced (eg, inclusion of palliation, incorporation of public health programmes); and how service effectiveness or quality is incorporated.

Global UHC measurement needs were further elevated by the announcement of WHO's draft Thirteenth General Programme of Work (GPW13) and its "triple billion targets" in November 2017.[20] Designed to guide WHO priorities for 2019–2023 and accelerate progress during the SDG era, the GPW13 explicitly included UHC under ones of its billion targets. This target was eventually formalised as "1 billion more people benefiting from UHC" by 2023, with 2018 serving as the baseline for the five-year evaluation period.[17] In late 2017, the WHO Director General established the GPW13 Expert Reference Group (ERG) in order to develop measurement approaches for the triple billion targets, including the one focused on UHC. During its consultative process in 2017–2018, the GPW13 ERG and its Metrics Taskforce recommended developing a UHC service coverage measure that could capture the construct of effective coverage,[21] or the fraction of potential health gain associated with receiving a service or intervention that is actually delivered by a population.[22–24] It was also recommended to establish a measurement framework reflective of health service areas that address the needs of major population groups across the life course, and then aim to fill the resulting matrix with corresponding indicators.

**Table 1.1: Examples of global definitions of UHC**

| Year | Source | UHC definition |
|---|---|---|
| 2005 | World Health Assembly Resolution 58.33[13] | "Access to key promotive, preventive, curative, and rehabilitative interventions for all at an affordable cost, thereby achieving equity in access." |
| 2010 | WHO. *The World Health Report 2010: Health systems financing: the path to universal coverage.*[14] | "Financing systems need to be specifically designed to:<br>- provide all people with access to needed health services (including prevention, promotion, treatment and rehabilitation) of sufficient quality to be effective;<br>- ensure that the use of these services does not expose the user to financial hardship." |
| 2014 | WHO/World Bank. *Monitoring progress towards universal health coverage at country and global levels: framework, measures, and targets.*[15] | "All people who need health services (promotion, prevention, treatment, rehabilitation and palliation) receive them, without undue financial hardship."<br><br>"UHC has two interrelated components: the full spectrum of good-quality essential health services according to need, and protection from financial hardship, including possible impoverishment, due to out-of-pocket payments for health services." |
| 2015 | UN Sustainable Development Goals (SDGs), Target 3.8[2] | "Achieve UHC, including financial risk protection, access to quality essential health care services and access to safe, effective, quality and affordable essential medicines and vaccines for all." |
| 2015 | WHO/World Bank. *Tracking universal health coverage: first global monitoring report.*[1] | "UHC means all people receiving the health services they need, including health initiatives designed to promote better health (such as anti-tobacco policies), prevent illness (such as vaccinations), and to provide treatment, rehabilitation, and palliative care (such as end-of-life care) of sufficient quality to be effective while at the same time ensuring that the use of these services does not expose the user to financial hardship." |
| 2017 | WHO/World Bank. *Tracking universal health coverage: 2017 Global Monitoring Report.*[16] | "Universal health coverage means that all people receive the health services they need, including public health services designed to promote better health (such as anti-tobacco information campaigns and taxes), prevent illness (such as vaccinations), and to provide treatment, rehabilitation and palliative care (such as end-of-life care) of sufficient quality to be effective, while at the same time ensuring that the use of these services does not expose the user to financial hardship." |
| 2019 | WHO. *Thirteenth General Programme of Work 2019–2023: Promote Health, Keep the World Safe, Serve the Vulnerable.*[17] | "The goal of ensuring that all people and communities have access to and can use the high quality promotive, preventive, curative, rehabilitative and palliative health services that are appropriate to their needs and expectations, while not exposing the user to financial hardship." |
| 2019 | WHO. *Primary Health Care on the Road to Universal Health Coverage: 2019 Monitoring Report.*[18] | "Universal health coverage means that all people receive the health services they need, including public health services designed to promote better health (such as anti-tobacco information campaigns and taxes), prevent illness (such as vaccinations), and to provide treatment, rehabilitation and palliative care (such as end-of-life care) of sufficient quality to be effective, while at the same time ensuring that the use of these services does not expose the user to financial hardship." |
| 2019 | United Nations General Assembly Resolution 74/2[19]<br><br>Political Declaration of the High-Level Meeting on Universal Health Coverage. *Universal health coverage: moving together to build a healthier world*[25] | "Universal health coverage implies that all people have access, without discrimination, to nationally determined sets of needed promotive, preventive, curative, rehabilitative, and palliative essential health services, and essential, safe, affordable, effective and quality medicines and vaccines, while ensuring that the use of these services does not expose the users to financial hardship, with a special emphasis on the poor, vulnerable, and marginalized segments of the population." |

In March 2018, the UN Statistical Commission endorsed the UHC service coverage index (SCI) as the metric for monitoring SDG indicator 3.8.1.[26] The UHC SCI sought to directly translate the SDG indicator 3.8.1 definition – "Coverage of essential health services (defined as the average coverage of essential services based on tracer interventions that include reproductive, maternal, newborn and child health, infectious diseases, non-communicable diseases and service capacity and access, among the general and the most disadvantaged population)" – by including three to four tracer indicators mapped to each listed category. Some UHC SCI indicators are direct measures of intervention coverage (eg, met need for family planning with modern contraception; antiretroviral therapy [ART] coverage among people living with HIV), while others included rescaled measures of risk factor exposure (eg, prevalence of non-tobacco use; prevalence of non-elevated blood pressure) and health system inputs (eg, hospital beds per capita; health worker density).[3] Limitations of the UHC SCI have been acknowledged by WHO, the indicator's custodial agency,[16,18,27] namely in terms of not expressly accounting for the effectiveness of services received and approximating coverage of non-communicable disease (NCD) interventions with prevalence-based measures. Other critiques involve the UHC SCI's over-emphasis on infectious diseases and reproductive, maternal, neonatal, and child health (RMNCH) interventions;[8,28] its omission of indicators representative of high-priority health needs across the life course;[29] and inadequate representation of health priorities across the development spectrum,[30] among others.

Approximately one year later, in May 2019, the World Health Assembly (WHA) Resolution A72/5 emphasised the importance of moving toward measuring effective coverage for UHC service coverage,[31] a sentiment echoed by the IAEG-SDGs. Such work would be used to inform monitoring of progress toward GPW13 milestones, namely the UHC billion target, conditional on methodological completion, peer-review, and country pilots. Per the GPW13's emphasis on the continuum of care in UHC,[17] the updated UHC effective coverage metric would "include tracer indicators by type of care (promotion, prevention, treatment, rehabilitation, and palliation) and by age group (life course)."[31] The overall index would then be calculated by combining tracers weighted by the potential health gain achievable by the interventions or services represented by each tracer.

As described more in Section 3, the development and consultation process on the UHC effective coverage framework primarily spanned 2017 to 2019. This work was guided by the GPW13's UHC billion target and the prevailing recognition that current measures of UHC service coverage to date were not expressly capturing interventions across the life course and incorporating effective coverage of services received.

## Section 3. UHC effective coverage framework development and consultation

As established by the WHO Director-General in late 2017, the ERG on the Draft GPW13 Impact Framework 2019–2023[32] sought to advise on the development and refinement of methods to measure the "triple billion" targets, as well as indicators and targets for the broader WHO Impact Framework. Measuring UHC billion target, which called for "1 billion more people benefiting from UHC," served as the foundation for developing the UHC effective coverage framework and the corresponding UHC effective coverage index (ECI). The high-level timeline for this process is summarised in Table 1.2, as informed by ERG documentation, presentations, and the WHO Secretariat.[24,32]

**Table 1.2: High-level timeline for UHC effective coverage methods development and consultation**

| Year | Months | Methods and/or consultation step |
|---|---|---|
| 2017 | August | WHO Technical programmes propose targets and triple billion methods |
| | September | WHO Executive Board session on triple billion measurement, WHO Impact Framework targets, and SDG alignment |
| | October-December | WHO Director-General constitutes GPW13 Expert Reference Group (ERG); ERG initiates deliberations |
| | November | Draft GPW13 released |
| 2018 | January-May | Series of WHO Secretariat and ERG/Taskforce on Metrics meetings on triple billion methods |
| | February | ERG meeting and Taskforce on Metrics establishment |
| | May | GPW13 approved at World Health Assembly; initial triple billion methods and ERG/Taskforce on Metrics published[21] |
| | June | WHO Secretariat, WHO regions, and ERG Taskforce meeting in Seattle, WA, on triple billions methods |
| | August-October | WHO Secretariat and ERG Taskforce meeting in Geneva on triple billion methods; continued methods refinement and consultation via Regional Committee meetings, web-based engagement with partners, including recommendations to present effective coverage as improved approach to measuring SDG 3.8.1 to the IAEG-SDGs |
| | November | IAEG-SDGs presentation on 3.8.1 measurement; African Union Statistical commission engagement |
| | December | Pan-American Health Organization (PAHO) consultation; Mission briefing |
| 2019 | January-May | Continued methods refinement and consultation coordinated via WHO Secretariat |
| | January | IAEG-SDGs presentation on 3.8.1 measurement;[24] WHO Executive Board meeting briefing; UHC 2030 and SDG3 action plan partners briefing |
| | May | World Health Assembly Resolution 72/5 on GPW13 and WHO Impact Framework measurement[31] |
| | October | WHO Secretariat and Member State meeting in Geneva on triple billion methods |
| | December | UHC effective coverage index methodology submitted for peer-review |

Per the ERG and consultation feedback,[21] we used the following recommendations to inform the overall UHC effective coverage measurement framework and methods development:

1) **Include the main services households seek and/or receive that can lead to catastrophic health spending**, thus representing the two key dimensions of measuring UHC (ie, service coverage and financial risk protection).
    i. WHO has expressly included public health programmes and policy like tobacco taxation in its definition of UHC under health promotion for several years (see Table 1.1).[1,16,18] While multisectoral actions to improve health have vital roles alongside or in coordination with UHC initiatives, to understand and measure progress toward achieving overall UHC (ie, all people receiving quality health services they need without financial hardship), the focus should be on interventions and services provided within the immediate health system.

2) **Incorporate constructs of effective coverage**, such that tracer indicators of UHC service coverage ideally reflect access to quality care and health gains associated with effective intervention receipt. At the health system level, effective coverage reflects the fraction of potential health gain received through services or interventions that is actually delivered to a given population.[22,23]

3) **Expand included health service areas to better represent the range of service types** (ie, promotive, preventive, curative, rehabilitative, and palliative services) emphasised by the GPW13 and prior WHO definitions of UHC.
    i. Past and current measures of UHC service coverage,[16,18,27,33] including those proposed by the GBD collaboration for health-related SDG monitoring,[28,34,35] have not sought to structure measurement around these types of services. Rehabilitation and palliative care indicators are not included in any global UHC service coverage measure to date.

4) **Represent priority health needs across settings based on tracer indicators of UHC service coverage**. The specific interventions or packages of services that are ultimately included in UHC will vary by country or Member State due to differences in local epidemiological profiles, health system organisation, financial resources, and political or societal demands.

5) **As appropriate, use outcome-based tracer indicators to better approximate health-care access and quality to key services**. This approach enables comparable measurement while recognising differences in Member State UHC service packages and implementation.
    i. Such outcome-based indicators should be risk-adjusted to account for factors and risks that can affect health outcomes outside of the immediate purview of UHC and health systems. Robust methods on risk-adjustment or standardisation should be used.[36,37]
    ii. Other types of outcome-based measures, like mortality-to-incidence ratios (MIRs), should be considered where there are sufficient, high-quality data on both cause-specific deaths and non-fatal outcomes (eg, cancer registries). In the case of cancers, MIRs have been shown to be good measures of access to quality cancer diagnostic and treatment services.[38–41]

**6) Ground UHC service coverage measurement on a framework mapping needs of major population groups against main service types relevant to UHC.** This matrix will be composed of $x$ cells representing different combinations of population groups and service areas.

**7) Map one or several UHC service coverage indicators per health service area–population group** based on these criteria:
    **i.** Tracer indicators should be correlated with a broader set of interventions or services that provide health gains in that area;
    **ii.** Tracer indicators should be measurable (ie, currently available data and methods support their measurement today);
    **iii.** Tracer indicators should be important in their own right for UHC and thus improving health;
    **iv.** Tracer indicators should reflect variations in UHC service coverage and not factors that are outside of the immediate scope of health systems and UHC (ie, social and environmental determinants);
    **v.** Tracer indicators should be drawn from indicators already included in the SDGs or GWP13, and/or the data systems required for SDG or GPW13 monitoring.

**8) Represent a range of health areas where UHC can provide health gains across conditions, including communicable, RMNCH, NCDs, and injuries.** Where possible, seek balance in indicator representation.

**9) Combine individual indicators into an overall measure of UHC service coverage by reflecting the magnitude of health gain potentially achievable through health service or interventions.** Weighting

indicators relative to potential disease burden addressable under maximum performance of interventions or service received would be a recommended approach, per prior effective coverage methodologies.[22,23]

In May 2018, the initial UHC effective coverage framework, as proposed to the ERG and Taskforce on Metrics, was a matrix composed of seven population groups – neonates (< 28 days); infants and children under 5 (29 days–4 years); children and adolescents (5–19 years); adults (20–64 years), separated by sex (male and female); reproductive health, female; older adults (≥65 years) – against health services subdivided by platform (ie, levels of care), function (ie, prevention and treatment), and service areas.[42] A total of 36 indicators were mapped to this matrix, drawing from tracer indicators included the UHC SCI,[27] the GBD 2016 measure for UHC service coverage for health-related SDGs monitoring,[28] and the draft GPW13 monitoring framework at that time. This exercise was viewed as a helpful start, but likely too complex or unwieldy to garner further traction.

From May to December 2018, a streamlined UHC effective coverage framework was developed in consultation with the ERG, WHO Secretariat, and partners (eg, WHO regional office representatives). This matrix consolidated population-age groups to a total of five categories – reproductive and newborn, children under 5 years, children and adolescents (5–19 years), adults (20–64 years), and older adults (≥65 years) – to represent the life course and focused on five main health service areas (promotion, prevention, treatment, rehabilitation, and palliation), with treatment sub-divided into communicable diseases and maternal and child health (MCH) and then NCDs.[24] A total of 30 unique "cells" (ie, combinations of population–age groups and health service types) resulted from this matrix, which became the UHC effective coverage measurement framework used in the present study (Figure 1.1; Figure 1A in the main manuscript).

**Figure 1.1: UHC effective coverage framework as a matrix of population age groups and health service types**

| | Health service type | | | | | |
|---|---|---|---|---|---|---|
| **Population age-group** | **Promotion** | **Prevention** | **Treatment** | | **Rehabilitation** | **Palliation** |
| | | | **Communicable diseases and MNCH** | **NCDs** | | |
| **Reproductive and newborn** | | | | | | |
| **Children under 5 years** | | | | | | |
| **Children and adolescents (5-19** | | | | | | |
| **Adults (20-64 years)** | | | | | | |
| **Older adults (≥ 65 years)** | | | | | | |

Consolidating each portion of the measurement matrix was recognised as a potential drawback, a decision that could mask important differences within particular population age groups (eg, older children and adolescents; adults aged 20–40 versus 40–64 years) and subtypes of important health services. However, providing a more streamlined initial framework was viewed as necessary for facilitating further progress on UHC effective coverage measurement. Future iterations of this work could further assess data availability and methods for quantifying further sub-divisions, especially within population age groups.

## Section 4. UHC effective coverage indicator selection

In line with ERG recommendations and feedback from consultation processes, we sought to identify tracer indicators that met inclusion criteria (Section 2) and could be mapped to the UHC effective coverage measurement framework. First, we examined currently available multi-country UHC service coverage measures from WHO, the World Bank, and the GBD collaboration, as well as individual indicators included in the global SDG indicator framework and GPW13. We also considered indicators that had been previously omitted from these sources to date but were considered important for capturing priority health services across the life course (eg, receipt of hearing aids or rehabilitative hearing services for hearing impaired; dental care). We then consider if, and then how, these indicators could be directly measured through the effective coverage construct or could be well-approximated through outcome-based measures like mortality-to-incidence ratios (MIRs) or risk-adjusted mortality rates. Last, we sought to identify a parsimonious set of indicators that could (1) appropriately represent the range of health services and population–age groups within the UHC effective coverage framework; and (2) be measured via data systems required for monitoring SDG or GPW13 progress. For instance, as shown in Table 1.3, some interventions and services may have been over-represented in GBD UHC service coverage measure (eg, DTP3 coverage and risk-standardised death rates from diphtheria, tetanus, and pertussis were all included in this index).

Following consultation and feedback from the GPW13 ERG and Metrics Taskforce, WHO Secretariat, and partners (eg, WHO regional official representatives), a total of 39 tracer indicators were proposed for an updated measure of UHC service coverage to the IAEG-SDGs in January 2019 (Table 1.4).[24] Of this total, 31 indicators were considered potentially measurable at that point in time (though some were conditional on gaining access to more microdata), while eight indicators were considered "aspirational" due to their inadequate data availability and/or measurement strategy.

The IAEG-SDGs requested additional refinement as well as formal peer review before taking any further action on considering methodological updates to SDG indicator 3.8.1. This feedback was also reflected in the WHA 72/5 resolution adopted in May 2019, wherein: "The IAEG-SDGs agreed that there was a need to make progress towards measuring 'effective' coverage and recommended that in the interim the Secretariat continue to use the current service coverage index. Once the methodological work related to the updated index has been completed, peer-reviewed and piloted in some countries, the Secretariat will approach the IAEG-SDGs to request approval of the updated methodology."[31]

Coordinated by WHO Secretariat, continued consultation on the UHC effective coverage framework and effective coverage indicators pointed to the need to further consolidate proposed indicators for methodological testing and future country pilots. Focusing on currently measurable indicators for which sufficient data across countries and methods for measurement existed today, all aspirational indicators were excluded. An additional seven indicators were then excluded, as further investigation into data availability and quality for directly measuring intervention coverage or developing proxy indicators showed serious gaps and/or challenges in appropriately estimating performance across countries and over time.

**Table 1.3: UHC service coverage indicators included in multi-country indices published by WHO, World Bank, and the GBD collaboration.** UHC=universal health coverage. SCI=service coverage index. SC=service coverage. RMNCH=reproductive, maternal, neonatal, and child health. NCDs=non-communicable diseases. ANC=antenatal care. DTP=diphtheria, tetanus, pertussis vaccine. ART=antiretroviral therapy. TB=tuberculosis. ITN=insecticide-treated net. IHR=International Health Regulations. BCG= Bacillus Calmette-Guérin. MCV1=measles-containing vaccine, 1 dose. HAQ=Healthcare Access and Quality.

| **UHC SCI**[16,27] | **SC index**[43]* | **GBD UHC service coverage index**[28,35] |
|---|---|---|
| **Total indicators:** 14<br><br>**Categorisation**: 4 (RMNCH, infectious diseases, NCDs, service access and capacity)<br><br>**Indicators**<br>RMNCH<br>-Met need for family planning with modern contraception<br>-ANC4<br>-DTP3<br>-Care-seeking for suspected pneumonia for under-5 children<br><br>Infectious diseases<br>-ART coverage<br>-TB case detection and treatment<br>-ITN use for under-5 children (*high malaria transmission countries only*)<br><br>NCDs<br>-Prevalence of non-tobacco use<br>-Prevalence of non-raised blood pressure<br>-Mean fasting plasma glucose<br><br>Service capacity access<br>-Hospital beds per capita<br>-Health worker density (physicians, surgeons, psychiatrists)<br>-IHR score | **Total indicators:** 8<br><br>**Categorisation:** 2 (prevention and treatment)<br><br>**Indicators**<br>Prevention<br>-ANC4<br>-Full vaccination (BCG, DTP3, Polio3, MCV1)<br>-Cervical cancer screening<br>-Breast cancer screening<br><br>Treatment<br>-Skilled birth attendance<br>-Treatment for acute respiratory infection (ARI)<br>-Treatment for diarrhoea<br>-Inpatient admission rates<br><br>*A study using these SC indicators was published in 2020.[33] | Total indicators: 41<br><br>**Categorisation:** no formal; 9 intervention coverage measures and 32 outcome-based measures from the HAQ Index[36,37]<br><br>**Indicators**<br>Intervention coverage:<br>-Met need for family planning with modern contraception<br>-ANC1<br>-ANC4<br>-Skilled birth attendance<br>-In-facility delivery rate<br>-DTP3<br>-Polio3<br>-MCV1<br>-ART coverage<br><br>Outcome-based (risk-standardised death rates or mortality-to-incidence ratios)<br>-Tuberculosis<br>-Diarrhoeal diseases<br>-Lower respiratory infections<br>-Upper respiratory infections<br>-Asthma<br>-Diphtheria<br>-Whooping cough<br>-Tetanus<br>-Measles<br>-Maternal disorders<br>-Neonatal disorders<br>-Colon and rectum cancer<br>-Non-melanoma skin cancer<br>-Breast cancer<br>-Cervical cancer<br>-Uterine cancer<br>-Testicular cancer<br>-Hodgkin lymphoma<br>-Leukaemia<br>-Rheumatic heart disease<br>-Ischaemic heart disease<br>-Cerebrovascular disease<br>-Hypertensive heart disease<br>-Peptic ulcer disease<br>-Appendicitis<br>-Hernia<br>-Gallbladder and biliary diseases<br>-Epilepsy<br>-Diabetes<br>-Chronic kidney disease<br>-Chronic obstructive pulmonary disease<br>-Congenital heart anomalies<br>-Adverse effects of medical treatment |

**Table 1.4: Proposed effective coverage (EC) tracer indicators for UHC service coverage measurement, January 2019.** For additional detail, please refer to addendum documents found on the IAEG-SDGs website.[24]

| No | EC indicator | Service type(s) | Population age groups | Proposal status |
|---|---|---|---|---|
| 1 | **Met need for FP with modern contraception**: fraction of women with need for family planning met by using modern contraceptives | Promotion | Reproductive and newborn | Include |
| 2 | **Breastfeeding promotion**: fraction of newborns initiating breastfeeding within 1 hour of birth | Promotion | Reproductive and newborn | Include |
| 3 | **Antenatal care:** fraction of women who received at least 4 ANC visits with quality indicators | Prevention | Reproductive and newborn | Aspirational |
| 4 | **Immunisation:** proportion of children receiving DTP3, MCV2, PCV3 | Prevention | Children under 5 | Include |
| 5 | **NTD preventive services**: geometric mean of treatment coverage of 5 NTDs | Prevention | Children under 5; children and adolescents (5-19); adults (20-64); older adults (≥ 65 years) | Include |
| 6 | **Malaria vector control**: fraction of a population sleeping under an ITN or with effective IRS | Prevention | Children under 5; children and adolescents (5-19); adults (20-64); older adults (≥ 65) | Include |
| 7 | **HPV vaccination:** fraction of girls receiving final HPV dose | Prevention | Children and adolescents (5-19) | Include |
| 8 | **Elevated BP management**: fraction of individuals with hypertension reaching treatment targets of SBP/DBP <140/90 | Prevention | Adults (20-64); older adults (≥ 65) | Include |
| 9 | **Elevated blood glucose management**: fraction of individuals with elevated blood sugar reaching the treatment target of FPG < 126 mg/dL | Prevention | Adults (20-64); older adults (≥ 65) | Include |
| 10 | **Management of labour and delivery**: proportion of livebirths delivered with a skilled birth attendant present | Treatment, Comm & MCH | Reproductive and newborn | Include |
| 11 | **Antenatal, peripartum, and postnatal care for the newborn**: proxied using rescaled early neonatal death rate | Treatment, Comm & MCH | Reproductive and newborn | Include |
| 12 | **Antenatal, peripartum, and postnatal care for the mother:** proxied using rescaled maternal mortality ratio | Treatment, Comm & MCH | Reproductive and newborn | Include |
| 13 | **Perinatal care:** proxied using ratio of stillbirths to total births (livebirths and stillbirths) | Treatment, Comm & MCH | Reproductive and newborn | Include |
| 14 | **Treatment for pneumonia (LRIs):** proxied using rescaled death to incidence ratio for pneumonia (LRIs) | Treatment, Comm & MCH | Children under 5 | Include |
| 15 | **Treament of severe acute nutrition:** proxied using rescaled death to incidence ratio for severe acute malnutrition. | Treatment, Comm & MCH | Children under 5 | Include |
| 16 | **Treatment for diarrhoea**: proxied using rescaled death to incidence ratio for diarrhoea | Treatment, Comm & MCH | Children under 5 | Include |
| 17 | **ART**: fraction of individuals with HIV/AIDS receiving ART | Treatment, Comm & MCH | Children and adolescents (5-19); adults (20-64); older adults (≥ 65) | Include |

| No | EC indicator | Service type(s) | Population age groups | Proposal status |
|---|---|---|---|---|
| **18** | **TB treatment:** proxied by ratio of notified and treated tuberculosis cases (all forms) to estimated incidence | Treatment, Comm & MCH | Adults (20-64); older adults (≥ 65) | Include |
| **19** | **Hepatitis C virus treatment:** fraction of persons diagnosed with chronic hepatitis C virus infection receiving treatment | Treatment, Comm & MCH | Adults (20-64); older adults (≥ 65) | Include |
| **20** | **Congenital heart disease treatment:** proxied using rescaled death to prevalence ratio for congenital heart disease | Treatment, NCDs | Children under 5 | Include |
| **21** | **Surgical care for abdominal emergencies**: proxied using rescaled death to incidence ratio for appendicitis, paralytic ileus, and intestinal obstruction | Treatment, NCDs | Children and adolescents (5-19); adults (20-64); older adults (≥ 65) | Include |
| **22** | **Refractive error correction:** proxied using rescaled prevalence of moderate distance vision loss, severe distance vision loss, blindness due to uncorrected refractive error | Treatment, NCDs | Children and adolescents (5-19); adults (20-64); older adults (≥ 65) | Include |
| **23** | **Treatment of childhood leukaemias:** proxied using rescaled death to incidence ratio for childhood leukaemias | Treatment, NCDs | Children and adolescents (5-19) | Include |
| **24** | **Treatment of asthma:** proxied using rescaled death to prevalence ratio for asthma | Treatment, NCDs | Children and adolescents (5-19) | Include |
| **25** | **Dental care:** proxied using rescaled prevalence of caries in permanent teeth | Treatment, NCDs | Children and adolescents (5-19); adults (20-64); older adults (≥ 65) | Include |
| **26** | **Treatment for breast, cervical, colorectal, and uterine cancers:** proxied using rescaled death to incidence ratio of each cancer | Treatment, NCDs | Adults (20-64); older adults (≥ 65) | Include |
| **27** | **Treatment for ischaemic heart disease:** proxied using rescaled death to incidence ratio for ischaemic heart disease | Treatment, NCDs | Adults (20-64); older adults (≥ 65) | Include |
| **28** | **Treatment of stroke**: proxied using rescaled death to incidence ratio for stroke | Treatment, NCDs | Adults (20-64); older adults (≥ 65) | Include |
| **29** | **Treatment of COPD**: proxied using rescaled death to prevalence ratio for COPD | Treatment, NCDs | Adults (20-64); older adults (≥ 65) | Include |
| **30** | **Treatment of end-stage renal disease**: proxied using rescaled ratio of deaths to CKD to prevalence of end-stage renal disease | Treatment, NCDs | Adults (20-64); older adults (≥ 65) | Include |
| **31** | **Cataract surgery**: proxied using proportion of individuals with cataracts who have received cataract surgery | Treatment, NCDs | Older adults (≥ 65) | Include |
| **32** | **Treatment of endentulism (individuals with zero remaining permanent teeth):** proxied using rescaled prevalence of endentulism | Treatment, NCDs | Older adults (≥ 65) | Aspirational |
| **33** | **Treatment of severe mental health conditions**: proxied using rescaled coverage of treatment for severe mental health conditions | Treatment, NCDs | Adults (20-64); older adults (≥ 65) | Aspirational |
| **34** | **Treatment for substance abuse:** proxied using fraction of people with substance abuse disorders who receiving some type of treatment | Treatment, NCDs | Adults (20-64); older adults (≥ 65) | Aspirational |
| **35** | **Prehospital emergency care services:** proxied using rescaled proportion of adults and children dying of acute injury in hospital among all acute injury deaths | Treatment, NCDs | Children and adolescents (5-19); adults (20-64); older adults (≥ 65) | Aspirational |

| No | EC indicator | Service type(s) | Population age-groups | Proposal status |
|---|---|---|---|---|
| 36 | **Rehabilitation after complex injury:** proxied using proportion of individuals with complex injuries who receive multiple modes of rehabilitation | Rehabilitation | Adults (20-64); older adults (≥ 65) | Aspirational |
| 37 | **Treatment for hip osteoarthritis**: proxied using ratio of people with hip replacements with people who need a hip replacement | Rehabilitation | Older adults (≥ 65) | Aspirational |
| 38 | **Rehabilitation for hearing loss for infants**: proxied by proportion of deaf or hard-of-hearing infants receiving hearing loss services | Rehabilitation | Children under 5 | Aspirational |
| 39 | **Palliation for cancer:** proxied using morphine-equivalent strong opioid analgesics (excluding methadone) per death from cancer | Palliation | Adults (20-64); older adults (≥ 65) | Include |

These 7 omitted indicators were (1) preventive therapy for select NTDs; (2) HPV vaccination; (3) hepatitis C treatment; (4) cataract surgery; (5) refraction error correction; (6) dental care; and (7) palliation for cancer. Breastfeeding, malnutrition treatment, and stillbirths proxying perinatal care were then omitted due to mixed evidence on effectiveness at the population level, followed by skilled birth attendance because the indicator using maternal mortality to ratio (MMR) to proxy antenatal, peripartum, and postnatal care for the mother was viewed as preferable for representing access to quality care. Congenital heart disease was then excluded due to data quantity and quality issues with consistently capturing prevalence across countries and over time.

From September to December 2019, additional indicator refinements and/or exclusions took place based on testing individual indicator and overall UHC effective coverage index measurement with GBD 2019 results. PCV3 coverage was excluded due to its partial introduction worldwide; for countries that had yet to introduce PCV3, neither of the measurement options explored for its inclusion – applying 0% for the effective coverage indicator value or not counting its associated burden in the health gains calculation – was considered sufficient to merit either risking over- or under-counting the potential health gains deliverable by health systems. MCV2 coverage was replaced with MCV1 for similar reasons: using MCV1 was preferred to implying 0% coverage – and thus no protection against measles – for countries that had not yet introduced MCV2, especially given the high efficaciousness of MCV1. The malaria vector control indicator was simplified to all-age ITN use, as no cohesive estimates of ITN use or indoor residual spraying (IRS) had yet to be generated across all malaria-endemic countries. Correspondingly, we limited the all-age ITN use to 39 medium-to-high malaria transmission countries in sub-Saharan Africa, following similar restrictions used by WHO for the UHC SCI.[3,18] This decision was made to avoid "penalizing" malaria-endemic countries where ITNs are not widely used and other types of interventions (eg, IRS, seasonal chemoprevention), which likely support population-level health gains against malaria, are more prevalent. Insufficient access to microdata on elevated blood pressure management and elevated blood glucose management ultimately hindered the generation of time series estimates across the 204 countries and territories included in the GBD 2019 study. Inclusion of these NCD treatment indicators will be revisited in future iterations of the GBD.

Last, in recognising that some countries may could have variable performance on subsets of cancers or types of surgical care for abdominal emergencies, the previously combined indicator on cancer (ie, breast, cervical, colon/rectum, and uterine cancers) were divided into four site-specific indicators, as was appendicitis and then paralytic ileus and intestinal obstruction.

After receiving feedback via formal peer review and commentary collected through the GBD collaboration, the following effective coverage indicator revisions were made in January to March 2020:

- The exclusion of all-age ITN use, as its currently limited application to a subset of countries made it similar to indicators (eg, PCV3) previously excluded;
- The addition of epilepsy for three population–age groups (children and adolescents [5–19 years], adults [20–64 years], and older adults [≥65 years]), which offers an effective coverage indictor for a neurological condition with good data availability and for which access to quality care should prevent mortality;[36,37,44,45]
- The refinement of the childhood leukaemia treatment indicator, such that the indicator is now limited to acute lymphoid leukaemia for two population–age groups (children under 5 years, children and adolescents [5–19 years]). This update is viewed as a better reflection of amenability to current cancer care for childhood leukaemias.

As also emphasised in the main manuscript, the currently included indicators were based on their mapping to health service types and population–age groups, as well as their fulfillment of inclusion criteria. Currently excluded indicators could certainly fulfill these specifications, particularly as data systems are strengthened and involve more high-priority indicator measurement (eg, palliation services). The indicators included in the present analysis are not meant to be prescriptive; rather, the primary objective was to establish a robust, comparable measurement framework from which UHC effective coverage could be assessed across settings and inform efforts to incorporate effective coverage into UHC monitoring. Continuing to improve UHC effective coverage measurement in the future is a high priority for the GBD collaboration and its partners.

**Figure 1.2: Mapping of proposed effective coverage indicators to the UHC effective coverage measurement framework.** Indicators coloured in blue are those included in the present framework; indicators in yellow were part of the January 2019 proposal, while indicators in orange were considered aspirational but not currently measurable due to data and/or methodological limitations.

| | Health service type | | | | | |
|---|---|---|---|---|---|---|
| **Population age-group** | **Promotion** | **Prevention** | **Treatment: Communicable diseases and MNCH** | **Treatment: NCDs** | **Rehabilitation** | **Palliation** |
| **Reproductive and newborn** | Met need for FP with modern contraception<br>Breastfeeding initiated within 1 hour of birth | Antenatal, peripartum, and postnatal care for newborns<br>Antenatal, peripartum, and postnatal care for mothers | Skilled birth attendance<br>Perinatal care<br>Antenatal care measured with quality indicators | | | |
| **Children under 5 years** | | DTP3 coverage<br>MCV2 coverage<br>Malaria vector control coverage<br>Preventive therapy against select NTDs | Pneumonia (LRI) treatment<br>Diarrhea treatment<br>Severe acute malnutrition treatment | Congenital heart disease treatment<br>Refractive error correction<br>Prehospital emergency care services | Rehabilitation for hearing loss among infants | |
| **Children and adolescents (5-19 years)** | | HPV vaccination<br>Malaria vector control<br>Preventive therapy against select NTDs | ART coverage | Leukaemia treatment<br>Asthma treatment<br>Surgical care for abdominal emergencies (appendicitis, paralytic ileus, and intestinal obstruction treatment)<br>Refractive error correction<br>Dental care (proxied by dental caries)<br>Prehospital emergency care services | Rehabilitation after complex injury | |
| **Adults (20-64 years)** | | Elevated BP management<br>Elevated blood glucose management<br>Malaria vector control<br>Preventive therapy against select NTDs | ART coverage<br>TB treatment<br>Hepatitis C treatment | Diabetes treatment<br>IHD treatment<br>Stroke treatment<br>CKD treatment<br>COPD treatment<br>Cancer treatment (breast, cervical, colorectal, uterine<br>Surgical care for abdominal emergencies (appendicitis, paralytic ileus, and intestinal obstruction treatment)<br>Refractive error correction<br>Dental care (proxied by dental caries)<br>Treatment of severe mental health conditions<br>Treatment for substance abuse<br>Prehospital emergency care services | Rehabilitation after complex injury | Palliation for cancer care |
| **Older adults (≥ 65 years)** | | Elevated BP management<br>Elevated blood glucose management<br>Malaria vector control<br>Preventive therapy against select NTDs | ART coverage<br>TB treatment<br>Hepatitis C treatment | Diabetes treatment<br>IHD treatment<br>Stroke treatment<br>CKD treatment<br>COPD treatment<br>Cancer treatment (breast, cervical, colorectal, uterine cancers)<br>Surgical care for abdominal emergencies (appendicitis, paralytic ileus, and intestinal obstruction treatment)<br>Refractive error correction<br>Cataract surgery<br>Dental care (proxied by dental caries)<br>Treatment of severe mental health conditions<br>Treatment for substance abuse<br>Prehospital emergency care<br>Edentulism | Rehabilitation after complex injury<br>Treatment for hip osteoarthritis | Palliation for cancer care |

## References


1 World Health Organization (WHO), World Bank. Tracking universal health coverage: First global monitoring report. Geneva, Switzerland; Washington, DC: WHO, World Bank, 2015 https://www.who.int/healthinfo/universal_health_coverage/report/2015/en/.

2 United Nations (UN). Transforming our world: the 2030 Agenda for Sustainable Development. New York, NY: UN, 2015.

3 UN Statistical Commission. SDG indicator 3.8.1 metadata. 2018 https://unstats.un.org/sdgs/metadata/files/Metadata-03-08-01.pdf.

4 UN Statistical Commission. SDG indicator 3.8.2 metadata. 2019 https://unstats.un.org/sdgs/metadata/files/Metadata-03-08-02.pdf.

5 Inter-agency Expert Group on the SDG Indicators (IAEG-SDGs). Final list of proposed Sustainable Development Goal indicators. IAEG-SDGs, 2016 https://sustainabledevelopment.un.org/content/documents/11803Official-List-of-Proposed-SDG-Indicators.pdf.

6 McIntyre D, McKee M, Balabanova D, Atim C, Reddy KS, Patcharanarumol W. Open letter on the SDGs: a robust measure for universal health coverage is essential. *The Lancet* 2016; **388**: 2871–2.

7 Reddock J. Seeking consensus on universal health coverage indicators in the sustainable development goals: *Journal of Health Services Research & Policy* 2017; published online May 8. DOI:10.1177/1355819617704676.

8 Fullman N, Lozano R. Towards a meaningful measure of universal health coverage for the next billion. *The Lancet Global Health* 2018; **6**: e122–3.

9 Jamison DT, Alwan A, Mock CN, *et al.* Universal health coverage and intersectoral action for health: key messages from Disease Control Priorities, 3rd edition. *Lancet* 2018; **391**: 1108–20.

10 Horton R. Offline: UHC—one promise and two misunderstandings. *The Lancet* 2018; **391**: 1342.

11 Inter-agency Expert Group on the SDG Indicators (IAEG-SDGs). Report of the Inter-agency and Expert Group on Sustainable Development Goal Indicators. New York, NY: United Nations, 2017 https://unstats.un.org/unsd/statcom/48th-session/documents/2017-2-IAEG-SDGs-E.pdf.

12 Ji JS, Chen L. UHC Presents Universal Challenges. *Health Systems & Reform* 2016; **2**: 11–4.

13 World Health Organization (WHO). World Health Assembly Resolution 58.33. Sustainable health financing, universal coverage and social health insurance. Geneva, Switzerland: WHO, 2005.

14 World Health Organization (WHO). The World Health Report 2010. Health Systems Financing: the Path to Universal Coverage. Geneva, Switzerland: WHO, 2010 https://apps.who.int/medicinedocs/documents/s20169en/s20169en.pdf.

15 World Health Organization (WHO), World Bank. Monitoring progress towards universal health coverage at country and global levels: framework, measures, and targets. Geneva, Switzerland; Washington, DC: WHO, World Bank, 2014.

16 World Health Organization (WHO), World Bank. Tracking universal health coverage: 2017 Global Monitoring Report. Geneva, Switzerland; Washington, DC: WHO, World Bank, 2017 https://www.who.int/healthinfo/universal_health_coverage/report/2017/en/.

17 World Health Organization (WHO). Thirteenth General Programme of Work 2019-2023: Promote Health, Keep the World Safe, Serve the Vulnerable. Geneva, Switzerland: WHO, 2019 https://apps.who.int/iris/bitstream/handle/10665/324775/WHO-PRP-18.1-eng.pdf.

18 World Health Organization (WHO). Primary Health Care on the Road to Universal Health Coverage: 2019 Monitoring Report. Geneva, Switzerland: WHO, 2019.

19 United Nations (UN). Resolution 74/2: Political declaration of the high-level meeting on universal health coverage. UN, 2019 https://undocs.org/en/A/RES/74/2 (accessed Nov 15, 2019).

20 World Health Organization (WHO). Draft thirteenth general programme of work 2019-2023: Promote health, keep the world safe, serve the vulnerable. Geneva, Switzerland: WHO, 2017 http://apps.who.int/gb/ebwha/pdf_files/EBSS4/EBSS4_2-en.pdf?ua=1&ua=1.

21 WHO Expert Reference Group on the Draft GPW 13. Preliminary Report 2018: Metrics for the GPW13. 2018 https://www.who.int/docs/default-source/documents/about-us/expert-reference-group-preliminary-report.pdf?sfvrsn=27a03101_2.

22 Shengelia B, Tandon A, Adams OB, Murray CJL. Access, utilization, quality, and effective coverage: an integrated conceptual framework and measurement strategy. *Soc Sci Med* 2005; **61**: 97–109.

23 Ng M, Fullman N, Dieleman JL, Flaxman AD, Murray CJL, Lim SS. Effective Coverage: A Metric for Monitoring Universal Health Coverage. *PLOS Medicine* 2014; **11**: e1001730.

24 World Health Organization (WHO). An updated method for the essential health services coverage index. 2019; published online Jan 17. https://unstats.un.org/sdgs/files/meetings/webex-17jan2019/1_3.8.1_Tier%20Reclassification%20Request_WHO.pdf.

25 Political Declaration of the High-level Meeting on Universal Health Coverage. "Universal health coverage: Moving Together to Build a Healthier World. Draft v4. 2019 https://www.healthpolicy-watch.org/wp-content/uploads/2019/07/UHC-Political-Declaration-Co-facilitators-revised-draft-REV.4.pdf.

26 Inter-agency Expert Group on the SDG Indicators (IAEG-SDGs). Report of the Inter-Agency and Expert Group on Sustainable Development Goal Indicators. New York, NY: United Nations, 2018 https://unstats.un.org/unsd/statcom/49th-session/documents/2018-2-SDG-IAEG-E.pdf.

27 Hogan DR, Stevens GA, Hosseinpoor AR, Boerma T. Monitoring universal health coverage within the Sustainable Development Goals: development and baseline data for an index of essential health services. *The Lancet Global Health* 2018; **6**: e152–68.

28 Fullman N, Barber RM, Abajobir AA, *et al.* Measuring progress and projecting attainment on the basis of past trends of the health-related Sustainable Development Goals in 188 countries: an analysis from the Global Burden of Disease Study 2016. *The Lancet* 2017; **390**: 1423–59.

29 Ataguba JE, Ingabire M-G. Universal Health Coverage: Assessing Service Coverage and Financial Protection for All. *Am J Public Health* 2016; **106**: 1780–1.

30 Bergen N, Ruckert A, Labonté R. Monitoring Frameworks for Universal Health Coverage: What About High-Income Countries? *International Journal of Health Policy and Management* 2019; **8**: 387–93.

31 World Health Organization (WHO). Resolution A72/5. Thirteenth General Programme of Work, 2019-2023. WHO Impact Framework. Geneva, Switzerland: WHO, 2019 http://apps.who.int/gb/ebwha/pdf_files/WHA72/A72_5-en.pdf.

32 Expert Reference Group on the Draft GPW 13 Impact Framework 2019-2023. https://www.who.int/about/what-we-do/thirteenth-general-programme-of-work-2019---2023/expert-reference-group-on-the-draft-gpw-13-impact-framework-2019-2023 (accessed Feb 25, 2020).

33 Wagstaff A, Neelsen S. A comprehensive assessment of universal health coverage in 111 countries: a retrospective observational study. *The Lancet Global Health* 2020; **8**: e39–49.

34 Lim SS, Allen K, Bhutta ZA, *et al.* Measuring the health-related Sustainable Development Goals in 188 countries: a baseline analysis from the Global Burden of Disease Study 2015. *The Lancet* 2016; **388**: 1813–50.

35 Lozano R, Fullman N, Abate D, *et al.* Measuring progress from 1990 to 2017 and projecting attainment to 2030 of the health-related Sustainable Development Goals for 195 countries and territories: a systematic analysis for the Global Burden of Disease Study 2017. *The Lancet* 2018; **392**: 2091–138.

36 Barber RM, Fullman N, Sorensen RJD, *et al.* Healthcare Access and Quality Index based on mortality from causes amenable to personal health care in 195 countries and territories, 1990–2015: a novel analysis from the Global Burden of Disease Study 2015. *The Lancet* 2017; **390**: 231–66.

37 Fullman N, Yearwood J, Abay SM, *et al.* Measuring performance on the Healthcare Access and Quality Index for 195 countries and territories and selected subnational locations: a systematic analysis from the Global Burden of Disease Study 2016. *The Lancet* 2018; **391**: 2236–71.

38 Coleman MP, Quaresma M, Berrino F, *et al.* Cancer survival in five continents: a worldwide population-based study (CONCORD). *The Lancet Oncology* 2008; **9**: 730–56.

39 De Angelis R, Sant M, Coleman MP, *et al.* Cancer survival in Europe 1999–2007 by country and age: results of EUROCARE-5—a population-based study. *The Lancet Oncology* 2014; **15**: 23–34.

40 Allemani C, Weir HK, Carreira H, *et al.* Global surveillance of cancer survival 1995–2009: analysis of individual data for 25 676 887 patients from 279 population-based registries in 67 countries (CONCORD-2). *The Lancet* 2015; **385**: 977–1010.

41 Allemani C, Matsuda T, Carlo VD, *et al.* Global surveillance of trends in cancer survival 2000–14 (CONCORD-3): analysis of individual records for 37 513 025 patients diagnosed with one of 18 cancers from 322 population-based registries in 71 countries. *The Lancet* 2018; **391**: 1023–75.

42 WHO Expert Reference Group on the Draft GPW13. Annex: Task Force Preliminary Mapping of UHC indicators. 2018; published online May. https://www.who.int/docs/default-source/documents/about-us/annex-expert-group-mapping.xlsx?sfvrsn=34c4a0a0_2.

43 Wagstaff A, Dmytraczenko T, Almeida G, *et al.* Assessing Latin America's Progress Toward Achieving Universal Health Coverage. *Health Affairs* 2015; **34**: 1704–12.

44 Nolte E, McKee M. Does healthcare save lives? Avoidable mortality revisited. London, UK: London School of Hygiene & Tropical Medicine, 2004 https://researchonline.lshtm.ac.uk/15535/1/does-healthcare-save-lives-mar04.pdf.

45 Nolte E, McKee CM. Measuring The Health Of Nations: Updating An Earlier Analysis. *Health Affairs* 2008; **27**: 58–71.

## Part 2. UHC effective coverage measurement

### Section 1. Overview

As also illustrated below, three main steps comprise measuring the UHC effective coverage index, which are detailed more in the following sections: 1) estimating effective coverage (EC) indicators; 2) calculating health gain weights; and 3) constructing the overall UHC effective coverage index.

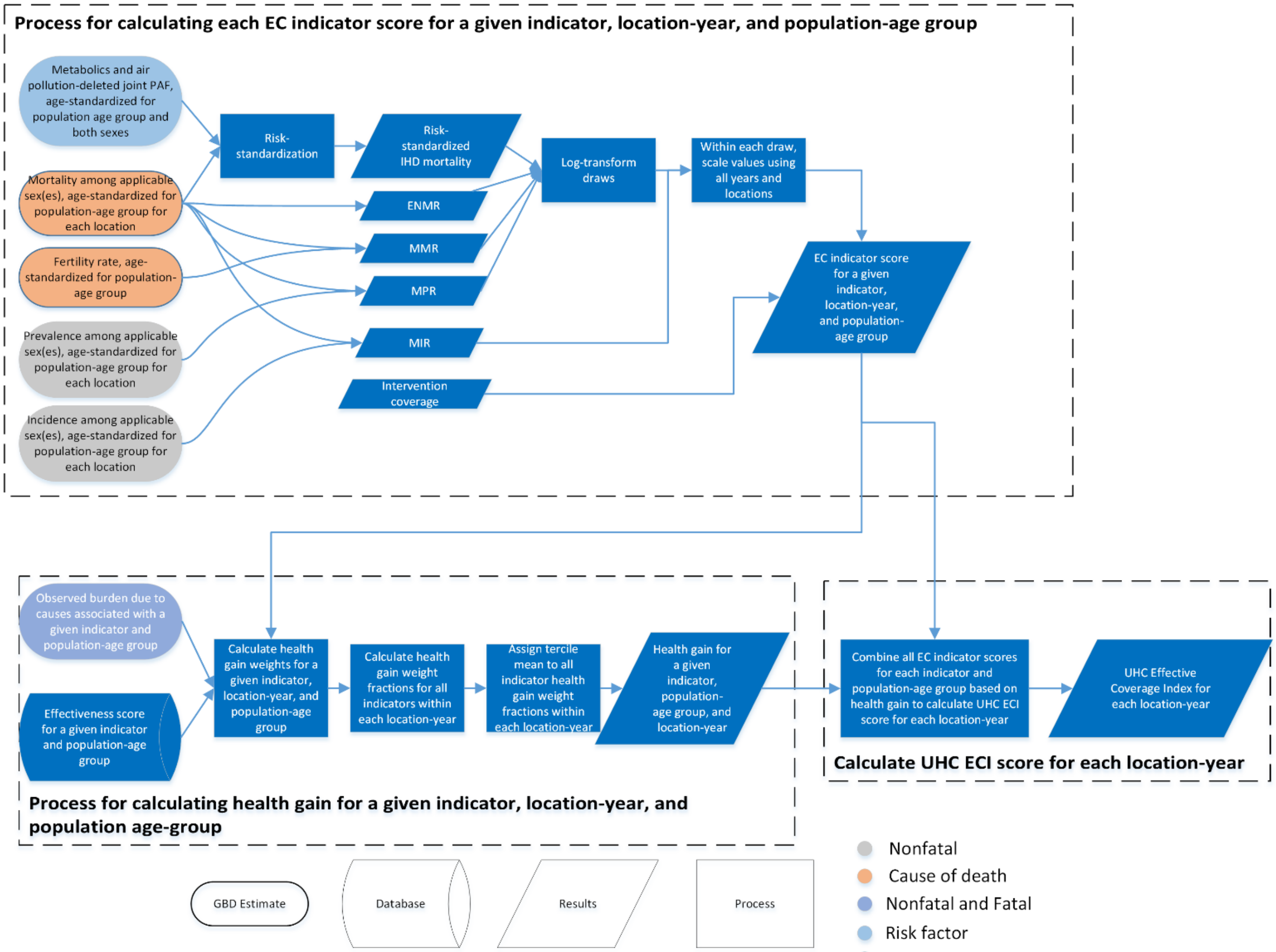


These analytical steps were informed by the UHC effective coverage measurement framework, which is described in the main manuscript and further in Part 1 of this methods appendix. This framework was informed by the WHO General Programme of Work 13 (GPW13) Task Force on Metrics and Expert Reference Group's recommendations,[1] as well as prior UHC monitoring efforts by WHO and the World Bank.[2,3]

Unless indicated otherwise, all inputs used for estimating UHC effective coverage index come from the broader Global Burden of Diseases, Injuries, and Risk Factors Study 2019 (GBD 2019); subsequently, greater detail on data inclusion, modelling processes and selection, and sensitivity analyses pertaining to each indicator, corresponding disease burden, and populations can be found in the GBD 2019 capstone publications.[4–6]

Analyses were conducted and figures were generated with R version 3.6.2 or Python 3.7.0.

## Section 2. Estimating effective coverage indicators

EC indicators fall into two broad categories: 1) direct estimates of intervention coverage (also known as crude coverage); and 2) outcome-based indicators that approximate health-care access and quality. Three different types of outcome-based indicators were used, as they each could better approximate health-care access and quality for a given set of interventions or services due to data or measurement considerations. These outcome-based indicators were ratio metrics (ie, mortality-to-incidence ratios [MIRs] or mortality-to-prevalence ratios [MPRs]), mortality measures transformed to a scale of 0-100 (ie, neonatal mortality and maternal mortality ratio [MMR]), and risk-standardised death rates (RSDRs).

EC indicator values, all of which ultimately were on a scale of 0 to 100 to represent coverage, were estimated separately for each population–age group relevant to the indicator. For instance, for the indicator on ischaemic heart disease (IHD) treatment, EC indicator values (for coverage scores and health gain weights) were computed separately for the population–age groups of adults 20–64 years and ≥65 years. Whenever single EC indicator values were required (eg, figure 1 in the main manuscript), they were based on combining individual population-age group indicator scores. Figure 1B in the main manuscript further details population–age groups associated with each EC indicator, as well as what comprises each indicator.

### Direct estimates of intervention coverage indicators

Four indicators were direct measures of intervention coverage (or crude coverage): antiretroviral therapy (ART) coverage among people living with HIV; diphtheria-tetanus-pertussis vaccine, three doses (DTP3) coverage; measles-containing vaccine, 1 dose (MCV1); and met need for family planning satisfied with modern contraception.

Intervention coverage estimates were drawn from GBD 2019, many of which are computed as part of broader models for GBD (eg, ART coverage as part of HIV modelling with Spectrum EPP;[7] vaccine coverage for vaccine-preventable disease modelling;[5] met need for family planning for fertility estimation[4]) and/or monitoring the health-related Sustainable Development Goals (SDGs) (eg, SDG indicator 3.7.1 on met need for family planning with modern contraception[8]), and thus have corresponding uncertainty intervals (UIs) computed across 1000 draws associated with each location-year estimate.

In terms of alignment with population–age groups within the measurement framework, vaccine coverage indicators (DTP3 and MCV1) were based on coverage among children aged 12–23 months but were applied to represent coverage among children under 5 years of age. Met need for family planning with modern methods was estimated for women 15–49 years of age without age-standardisation.

### Outcome-based indicators

#### *Mortality-to-incidence and mortality-to-prevalence ratios*

MIRs were used for ten causes, while MPRs were used for six causes (as listed in figure 1B in the main manuscript). MPRs were preferred over MIRs for these six causes given the long duration of cases relative to potential mortality. For these 16 causes, MIRs and MPRs were viewed as good indicators approximating access to quality services targeting these conditions, particularly in the absence of robust coverage data across settings and over time.

To construct MIRs or MPRs for each cause and corresponding population–age group in the measurement framework, deaths and cases (incident or prevalent) were age-standardised by location-year and population–age group (eg, 5–19 years, 20–64, ≥65). Since our inputs are estimates from GBD 2019, 1000 draws of MIRs and MPRs were generated. Age-standardised mortality rates were then divided by the non-fatal equivalent to produce MIRs or MPRs. MPRs were logged using the natural log prior to scaling, though MIRs were not. Logging was not appropriate for some MIR indicators (eg, diarrhoea) since we found that logging MIRs led to arbitrarily decreased high coverage values in high-income places where we would expect coverage to consistently be very high. Last, these values were transformed to the 0–100 scale by rescaling locations $i$ for years $j$ 1990 to 2019, taking the 2.5th percentile (ie, lowest levels of MIRs or MPRs) observed of indicator $I$ across location-years as the "best" (100) and the 97.5th percentile (ie, highest levels of MIRs or MPRs) as the "worst" (0):

$$S_{i,j} = \frac{I_{i,j} - I_{2.5th}}{I_{97.5th} - I_{2.5th}}$$

This scaling approach has been widely used for other GBD-based index measures.[8–11] It is preferred over taking the absolute minimum or maximum values to set the rescaling bounds to minimise sensitivity to outliers or particular fluctuations over time. Only GBD Level 3 locations (countries and territories, no subnational locations) were included in the scaling process.

### *Mortality measures*

Early neonatal mortality and MMR were used to approximate care for newborns and mothers, respectively. We used the same scaling approach as described for MIRs and MPRs; prior to scaling, values were transformed on the natural log scale at the draw level. Uncertainty estimates in the form of 1000 draws were used for both early neonatal mortality and MMR.

### *Risk-standardised deaths*

We used risk-standardised deaths for IHD. Only acute incidence of IHD was estimated for GBD 2019, whereas mortality included all IHD sub-causes.[5] The use of MPRs would also not be ideal given that IHD deaths can be due to chronic or acute IHD.

For the present analysis, we drew from the risk-standardisation process used in previous GBD studies, namely those used for estimating the Healthcare Access and Quality (HAQ) Index.[9,10] Uncertainty, in the form of 1000 draws, was propagated through for each quantity in the risk-standardisation process. First, we risk-deleted deaths jointly attributable to risk factors, not including deaths attributable to high systolic blood pressure, high LDL cholesterol, high fasting plasma glucose, and air pollution. Like for the HAQ Index,[9,10] we did not standardise deaths attributable to high systolic blood pressure, high LDL cholesterol, and high fasting plasma glucose, as those risk factors are considered to be directly amenable to high-quality personal health care. According to GBD 2019, around 25% of deaths due to IHD in low-income countries were due to air pollution (almost two-thirds related to household air pollution).[6] The impact of air pollution on IHD death rate estimates in low-income countries led to very low risk-standardised death rates in low-income countries relative to high-income countries when air pollution deaths were deleted from the total IHD death rate. Based on the lack of face validity, we decided to not standardise for air pollution in addition to high systolic blood pressure, high LDL cholesterol, and high fasting plasma glucose. We will further evaluate the effect of risk factors on IHD mortality and the use of risk-standardisation in future iterations of the UHC effective coverage index. We used the joint exposure, referred to as the joint population attributable fraction (PAF), for all risk factors minus the PAF for those four risks to arrive at a new PAF (NPAF). We then scaled the NPAF so that 0.9 (out of 1) is the maximum mean of the draws for a given age group, sex, location, and year. Deaths attributable (ND) to the NPAF were calculated by

multiplying cause-specific deaths (D) by NPAFs for each location $l$, year $y$, GBD age group $a$ within population-age group $p$ (eg, 20–24, 25–29, etc., for the population–age group 20–64 years), sex $s$, and indicator $i$:

$$ND_{l,y,a,s,i,p} = D_{l,y,a,s,i,p} * NPAF_{l,y,a,s,i,p}$$

We combined sex-specific estimates to arrive at both-sex counts of ND:

$$ND_{l,y,a,i,p} = ND_{l,y,a,males,i,p} + ND_{l,y,a,females,i,p}$$

We then age-standardised ND based on the GBD age groups within each included population–age group for a given indicator, and converted these estimates into proportions by dividing the both-sex, age-standardised ND by the both-sex, age-standardised number of total cause-specific deaths.

$$NPAF_{l,y,i,p} = \frac{ND_{l,y,i,p}}{D_{l,y,i,p}}$$

We then multiplied one minus the NPAF by all deaths to get the both-sex, age-standardised number of risk-deleted deaths (RDD):

$$RDD_{l,y,i,p} = D_{l,y,i,p} * (1 - NPAF_{l,y,i,p})$$

We then estimated risk-standardised deaths (RSD) by adding back deaths due to the global level of NPAF (as computed by one minus the global NPAF, or GPAF) averaged across all years using the following formula:

$$RSD_{l,y,i,p} = \frac{RDD_{l,y,i,p}}{(1 - GPAF_{i,p})}$$

We divided risk-standardised deaths by corresponding populations to convert these values into rates. After taking the natural log of these values, we then transformed these indicators to the 0–100 scale based on the rescaling approach described above.

## Section 3. Calculating health gain weights

In following the effective coverage conceptual framework at the health system level,[12] we sought to quantify the fraction of potential health gains delivered to populations relative to the health gains that could have been delivered given intervention or service effectiveness and health needs of a given setting. To do this across multiple health service areas, we needed a principled way of representing potential health gains and then weighting each indicator of health service area relative to potential health gains provided. In the present study, we developed health gain weights based on a given location's observed effective coverage indicator values and associated disease burden (as measured by disability-adjusted life-years [DALYs]), relative to what burden could have been addressed and effectiveness associated with EC indicators. These weights were applied at the indicator-population-age group level to ultimately compute the overall UHC effective coverage index for each location-year.

### Burden associated with EC indicators

As provided in figure 1 in the main manuscript, each EC indicator was linked to DALYs considered addressable by the interventions or services represented by the indicator. Of note, total DALYs due to maternal disorders were equally split (50%/50%) between met need for family planning with modern methods and the maternal care

indicator based on MMR; this was done to avoid double-counting of burden due to maternal disorders. To estimate burden associated with early neonatal mortality, we multiplied the number of early neonatal deaths by life expectancy at birth, per the theoretical minimum risk life table from GBD 2019.[4] We used 1000 draws of DALYs for each cause.

## Effective coverage indicator effectiveness

EC indicator effectiveness categories were informed by previously published literature (ie, Cochrane, Tufts Cost-Effectiveness Analysis [CEA] Registry, and DCP3[13–15]). Five categories were ultimately assigned, ranging from 1 (most effective) to 5 (least effective), with incremental values assumed for each category (ie, 90% effectiveness for category 1, 70% for category 2, 50% for category 3, 30% for category 4, and 10% for category 5). Assignments were made at the population–age group level for each EC indicator, as provided in Figure 1B in the main manuscript.

For some EC indicators, a large number of potential interventions or services have been identified for targeting a given condition, and reported effectiveness of these interventions or services is equally variable. Distilling such heterogeneous information into a summary measure of effectiveness was very challenging for some EC indicators, a recognised limitation of the present analysis.

## Health gain weights computation

First, we calculated two hypothetical burden scenarios ("zero coverage" and "full coverage") for a given indicator ($i$), population–age group ($p$), and location-year ($l, y$).

The "zero coverage" burden scenario was based on observed estimates of DALYs divided by one minus the effective coverage indicator value, which represented intervention coverage, multiplied by intervention effectiveness value assigned to the indicator; the zero coverage burden scenario represented expected disease burden in the total absence of interventions or health services.

$$Zero\ Coverage_{i,p,l,y} = \frac{Observed\ burden_{i,p,l,y}}{1 - intervention\ coverage_{i,p,l,y} * intervention\ effectiveness_{i,p}}$$

Second, the "full coverage" burden scenario was calculated by multiplying the burden estimated under "zero coverage" by one minus intervention effectiveness; the full coverage burden scenario represented expected disease burden if maximum levels of service coverage were achieved:

$$Full\ Coverage_{i,p,l,y} = Zero\ Coverage_{i,p,l,y} * (1 - 1 * intervention\ effectiveness_{i,p})$$

Subtracting levels of "full coverage" burden from burden under the "zero coverage" scenario resulted in the health gain weight – the population health gains potentially deliverable by health systems:

$$Health\ gain\ weight_{i,p,l,y} = Zero\ Coverage_{i,p,l,y} - Full\ Coverage_{i,p,l,y}$$

Below are examples of how the health gain weights are calculated for two hypothetical locations and two EC indicators.

**EC indicator on ART coverage and HIV burden**

| | Country 1 | Country 2 |
|---|---|---|
| **2019 estimates and effectiveness** | | |
| EC coverage for 20-64 year olds | 0.93 | 0.47 |
| Estimated burden (DALYs) | 5,837,148 | 593,241 |
| Effectiveness: Category 1 (90%) | 0.90 | 0.90 |
| **Zero coverage scenario** | | |
| Estimated burden (DALYs) | 5,837,148 | 593,241 |
| EC coverage*effectiveness | 0.84 | 0.42 |
| 1-(EC coverage*effectiveness) | 0.16 | 0.58 |
| **Hypothetical burden (at 0%)** | **35,810,724** | **1,028,147** |
| *HB (at 0%) = DALYs / (1-(EC coverage * effectiveness))* | | |
| **Full coveage scenario** | | |
| Hypothetical burden (at 0%) | 35,810,724 | 1,028,147 |
| 1-(1*effectiveness) | 0.10 | 0.10 |
| **Hypothetical burden (at full %)** | **3,581,072** | **102,815** |
| *HB (at full %) = HB (at 0%)*(1-(1*effectiveness))* | | |
| **Potential health gain** | | |
| Hypothetical burden (at 0%) | 35,810,724 | 1,028,147 |
| Hypothetical burden (at full %) | 3,581,072 | 102,815 |
| **Potential health gain** | **32,229,652** | **925,333** |
| *PHG = HB (at 0%) - HB (at full %)* | | |

**EC indicator on stroke treatment and stroke burden**

| | Country 3 | Country 4 |
|---|---|---|
| **2019 estimates and effectiveness** | | |
| EC coverage for ≥ 65 year olds | 0.33 | 0.85 |
| Estimated burden (DALYs) | 296,892 | 15,744 |
| Effectiveness: Category 2 (70%) | 0.70 | 0.70 |
| **Zero coverage scenario** | | |
| Estimated burden (DALYs) | 296,892 | 15,744 |
| EC coverage*effectiveness | 0.23 | 0.60 |
| 1-(EC coverage*effectiveness) | 0.77 | 0.41 |
| **Hypothetical burden (at 0%)** | **386,075** | **38,874** |
| *HB (at 0%) = DALYs / (1-(EC coverage * effectiveness))* | | |
| **Full coveage scenario** | | |
| Hypothetical burden (at 0%) | 386,075 | 38,874 |
| 1-(1*effectiveness) | 0.30 | 0.30 |
| **Hypothetical burden (at full %)** | **115,823** | **11,662** |
| *HB (at full %) = HB (at 0%)*(1-(1*effectiveness))* | | |
| **Potential health gain** | | |
| Hypothetical burden (at 0%) | 386,075 | 38,874 |
| Hypothetical burden (at full %) | 115,823 | 11,662 |
| **Potential health gain** | **270,253** | **27,212** |
| *PHG = HB (at 0%) - HB (at full %)* | | |

Health gain weights were transformed into fractions by dividing weights for each population–age group and EC indicator combination by the sum of all weights for a given location-year:

$$Health\ gain\ weight\ fraction_{i,p,l,y} = \frac{Health\ gain\ weight_{i,p,l,y}}{\sum(Health\ gain\ weight_{i,p,l,y})}$$

To ensure that the overall UHC effective coverage index was not dominated by one or a small subset of coverage scores, we grouped all health gain weight fractions for a given location-year into terciles, based on means of all draws. Then, within each tercile, location-year, and over each draw, we take mean health gain weight fraction of all of the EC indicator and population-age group combinations. That mean health gain weight fraction value is then used as the health gain weight fraction value for that draw for all indicators within that tercile.

## Section 4. Constructing the UHC effective coverage index

The UHC effective coverage index was constructed based on weighting each EC indicator by its corresponding health gain weight fractions at the population–age group level. This aggregation approach approximates the fraction of total health gains delivered at the population level by location-year. We also considered the effects of constructing the UHC effective coverage index without accounting for health gain weights; to do this, we took the arithmetic mean of each EC indicator, by population–age group, by location-year.

As shown in figure 2 in the main manuscript, index values were highly correlated ($r$=0.95) across locations. However, the effects varied by location and over GBD super-regions, with many countries in sub-Saharan Africa as well as some high-income countries showing higher index values under the weighted index versus unweighted index. In contrast, many locations in central Europe, eastern Europe, and central Asia, as well as Oceania, had higher index values under the unweighted approach versus health gains weighting scheme.

## Section 5. Sensitivity analyses

### Scaling percentiles

We implemented a scaling procedure for our outcome-based measures in order transform ratio metrics, mortality measures, and risk-standardised death rates into coverage measures on a 0 to 100 scale. Part of the scaling process involved trimming the outcome-based measures so that the scale was set using 2.5$^{th}$ percentile as the minimum and the 97.5$^{th}$ percentile as the maximum, in order to control for outliers. We tested varying the scaling cutoffs, using the 1$^{st}$ percentile as the minimum and the 99$^{th}$ percentile as the maximum. Varying the cutoffs to use the wider 1$^{st}$ percentile and 99$^{th}$ percentile caused some EC indicator-specific and overall index scores to vary, but the correlation between the overall index scores remained quite high ($r$=0.999) across all years.

### Varying effectiveness scores

Each EC indicator was ascribed an effectiveness score from 1 to 5. We tested the sensitivity of our index results to changes in the effectiveness scores. We subtracted 1 from each effectiveness score, thereby making each indicator 20 percentage points more effective (eg, shifting a category 2 [70%] EC indicator to category 1 [90%]; the exception being indicators that already had a score of 1, which remained the same), which resulted in overall index scores that were highly correlated ($r$=0.996) with our results. We also added 1 to each effectiveness score, thereby making each indicator 20 percentage points less effective (eg, shifting a category 3 [50%] EC indicator to category 2 [30%]; the exception being indicators that already had a score of 5, which remained the same), which also resulted in overall index scores that were highly correlated ($r$=0.996) with our current results.

### Using a different number of bands for the health gain weight fractions

The weighting process involved breaking up the health gain weight fractions into terciles and assigning the mean tercile weight to each indicator within a tercile. We tested using quartiles instead of terciles and found that the correlation between overall index scores in all location-years was quite high ($r$=0.992). We also tested using two groups, splitting at the median for each location-year, and again found that the correlation between overall index scores was quite high ($r$=0.986).

## Section 6. Uncertainty analysis

GBD aims to propagate sources of uncertainty throughout its estimation process, resulting in uncertainty intervals (UIs) that accompany each point estimate. For each GBD estimate, UIs are computed based on 1000 draws from the posterior distribution, and 95% UIs are based on the 2.5$^{th}$ and 97.5$^{th}$ percentile of draws for each quantity of interest.

For this analysis, we take the 1000 draws produced for the underlying values for each EC indicator and corresponding disease burden estimates from the GBD 2019 study.[4–6] Uncertainty was not formally propagated for effectiveness categories in health gain weights estimation as we used single values per category, or for GBD 2019 population estimates as the mean population estimate was used for each location, year, sex, and age group.

## References


1 WHO Expert Reference Group on the Draft GPW 13. Preliminary Report 2018: Metrics for the GPW13. 2018 https://www.who.int/docs/default-source/documents/about-us/expert-reference-group-preliminary-report.pdf?sfvrsn=27a03101_2.

2 World Health Organization (WHO), World Bank. Monitoring progress towards universal health coverage at country and global levels: framework, measures, and targets. Geneva, Switzerland; Washington, DC: WHO, World Bank, 2014.

3 Boerma T, Eozenou P, Evans D, Evans T, Kieny M-P, Wagstaff A. Monitoring Progress towards Universal Health Coverage at Country and Global Levels. *PLOS Medicine* 2014; **11**: e1001731.

4 GBD 2019 Demographics Collaborators. Global age-sex-specific fertility, mortality, healthy life expectancy, (HALE), and population estimates in 204 countries and territories, 1950–2019: a comprehensive demographic analysis for the Global Burden of Disease Study 2019. *The Lancet* In press.

5 GBD 2019 Diseases and Injuries Collaborators. Global burden of 369 diseases and injuries in 204 countries and territories, 1990–2019: a systematic analysis for the Global Burden of Disease Study 2019. *The Lancet* In press.

6 GBD 2019 Risk Factors Collaborators. Global burden of 87 risk factors in 204 countries and territories, 1990–2019; a systematic analysis for the Global Burden of Disease Study 2019. *The Lancet* In press.

7 Frank TD, Carter A, Jahagirdar D, *et al.* Global, regional, and national incidence, prevalence, and mortality of HIV, 1980–2017, and forecasts to 2030, for 195 countries and territories: a systematic analysis for the Global Burden of Diseases, Injuries, and Risk Factors Study 2017. *The Lancet HIV* 2019; **6**: e831–59.

8 Lozano R, Fullman N, Abate D, *et al.* Measuring progress from 1990 to 2017 and projecting attainment to 2030 of the health-related Sustainable Development Goals for 195 countries and territories: a systematic analysis for the Global Burden of Disease Study 2017. *The Lancet* 2018; **392**: 2091–138.

9 Barber RM, Fullman N, Sorensen RJD, *et al.* Healthcare Access and Quality Index based on mortality from causes amenable to personal health care in 195 countries and territories, 1990–2015: a novel analysis from the Global Burden of Disease Study 2015. *The Lancet* 2017; **390**: 231–66.

10 Fullman N, Yearwood J, Abay SM, *et al.* Measuring performance on the Healthcare Access and Quality Index for 195 countries and territories and selected subnational locations: a systematic analysis from the Global Burden of Disease Study 2016. *The Lancet* 2018; **391**: 2236–71.

11 Fullman N, Barber RM, Abajobir AA, *et al.* Measuring progress and projecting attainment on the basis of past trends of the health-related Sustainable Development Goals in 188 countries: an analysis from the Global Burden of Disease Study 2016. *The Lancet* 2017; **390**: 1423–59.

12 Shengelia B, Tandon A, Adams OB, Murray CJL. Access, utilization, quality, and effective coverage: an integrated conceptual framework and measurement strategy. *Soc Sci Med* 2005; **61**: 97–109.

13 Cochrane Database of Systematic Reviews (CDSR). /editorial-and-publishing-policy-resource/overview-cochrane-library-and-related-content/databases-included-cochrane-library/cochrane-database-systematic-reviews-cdsr (accessed Sept 19, 2018).

14 CEA Registry. http://healtheconomics.tuftsmedicalcenter.org/cear4/home.aspx (accessed Sept 19, 2018).

15 Global Health Cost-Effectiveness Analysis Registry. http://healtheconomics.tuftsmedicalcenter.org/orchard (accessed Sept 19, 2018).

# 3. Validation

## Section 1. Overview

Since no gold standard of UHC service coverage exists presently, we compared UHC effective coverage index performance against previous multi-country UHC service coverage indices on three validity tests: content validity, known-groups validity, and convergent validity. Details are provided in the following sections and are accompanied by figures and tables with each validity test's results.

These approaches and inputs were selected *a priori* in October 2018 based on inputs and guidance from the WHO GPW13 Expert Reference Group (ERG). Since then, the only updates or changes incorporated into these validity tests were on the basis of newly published estimates or studies: WHO published new UHC SCI estimates for 2017 in September 2019;[1] a study on overall UHC was first published in December 2019[2] and corresponding SC index estimates were made available in January 2020;[3] and Global Burden of Disease (GBD) 2019 estimates were finalised over the course of late December 2019 to early 2020.[4–6] Beyond the GBD 2019-based UHC effective coverage index, GBD 2019 estimates also were used for healthy life expectancy (HALE) and the Socio-demographic Index (SDI) as inputs for the convergent validity.

We compared performance for four multi-country index measures of UHC service coverage: (1) the GBD 2019-based UHC effective coverage index; (2) the GBD 2017-based UHC service coverage index,[7] which has been used previously for monitoring the health-related SDGs; (3) the WHO UHC SCI,[1] which is the current measure for SDG indicator 3.8.1;[8] and (4) the World Bank's SC index.[3] We did not include earlier estimates of the GBD-based UHC service coverage index[9] or the WHO UHC SCI;[10,11] because more recent analyses draw from the most up-to-date data sources and otherwise use the same measurement approach, earlier iterations of these indices are considered superseded by their more recent publications. For known-groups and convergent validity, we also included the unweighted average for the GBD 2019 UHC effective coverage index since the resulting index values differed from the health-gains weighted UHC effective coverage index.

## Section 2. Content validity

This test of validity was based on the UHC effective coverage framework, as developed by the GPW13 ERG and WHO Secretariat (see Part 1 under the methods appendix for more detail on this process). This framework involves a matrix of health service types and population–age groups, resulting in 30 unique "cells" that are meant to represent the range of potential health services needed across the life course. For each index, we mapped their individual indicators to each cell and calculated what percentage of those 30 cells were represented by currently included indicators. As shown in Table 3.1, the GBD 2019 UHC effective coverage index had 40% coverage, followed by the GBD 2017-based UHC service coverage index (33%), the UHC SCI (20%), and SC index (17%).

No index included indicators representing rehabilitation or palliation, a limitation for all measurement in trying to optimally capture UHC service coverage needs across populations. In addition, nearly all indices lacked indicators for promotion or prevention beyond a subset of population–age groups (namely reproductive and newborn, and children under 5); the main exception was the SC index, which included two cancer screening indicators. However, the SC index lacked NCD treatment indicators or proxies across population–age groups.

**Figure 3.1: UHC effective coverage framework as a matrix of population–age groups and health service types**

| | Health service type | | | | | |
|---|---|---|---|---|---|---|
| Population age-group | Promotion | Prevention | Treatment | | Rehabilitation | Palliation |
| | | | Communicable diseases and MNCH | NCDs | | |
| Reproductive and newborn | | | | | | |
| Children under 5 years | | | | | | |
| Children and adolescents (5-19 | | | | | | |
| Adults (20-64 years) | | | | | | |
| Older adults (≥ 65 years) | | | | | | |

## Section 3. Known-groups validity

For known-groups validity, we compared how well each index could discriminate between country-pairs for which previous studies show "country A" as having higher performance or faster progress on UHC service coverage than "country B", a country of similar geographical location and/or sociodemographic status with historically lower performance. 16 country-pairs and thus a total of 32 countries were identified for this test (Table 3.2); selections were made in October 2018 via inputs of the GPW13 ERG and thus were based on a combination of expert input and documentation by journal articles and reports.[12–22]

For each index, we calculated the percentage of total country-pairs (16) for which the mean for "country A" was higher than that of "country B." We also reported the ratio of "country A" to "country B" to approximate the distance between each country's performance (ie, values closer to 1 indicate greater similarity between the countries' means). For indices with reported uncertainty associated with their mean values, we also determined whether the performance of "country A" still exceeded that of "country B" when accounting for uncertainty (ie, each country's uncertainty bounds do not overlap). The WHO UHC SCI and World Bank SC index do not report uncertainty associated with their index estimates.

As shown in Table 3.2, using mean values, GBD-based indices (both methods for UHC effective coverage index and then the GBD 2017-based UHC service coverage index) each had 94% (15/16) of country-pairs with "country A" exceeding the average index performance of "country B." The UHC SCI for 2017 had 75% (12/16) and the SC index had 56% (9/16); of note, the latter did not have service coverage measures for five countries (Bolivia, Cuba, Belarus, Singapore, and Brunei). When uncertainty was accounted for, the GBD-based indices had lower percentages of country-pairs (56% to 69%). Five country pairs had overlapping uncertainty for GBD-based indices (ie, Brazil-Paraguay; Ethiopia-DRC; Rwanda-Burundi; Thailand-Sri Lanka; Turkey-Iran). In terms of "country A" to "country B" ratios, the GBD 2019 effective coverage index with health weight gains ranged from 0.99 (Turkey-Iran) to 1.41 (Singapore-Brunei). A somewhat similar range was found for the UHC SCI, spanning from 0.92 (Chile-Ecuador) to 1.37 (Rwanda-Burundi). In contrast, the World Bank SC index had a much wider range, from 0.48 (Turkey-Iran) to 3.05 (Ghana-Côte d'Ivoire). This may be related to country-level SC index

values being reported on the basis of most recent year of data available; subsequently, results for each country-pair could be from very different years and may not be wholly comparable.

To some degree this exercise was inherently subjective. No gold-standard measure of UHC service coverage exists, and thus comprehensive, consistently quantified time series of country-level UHC service coverage are not available outside of the indices being compared in the current study. It is very possible that a different set or number of country-pairs may been selected if this analysis occurred at a different point in time; on the basis of different types of consultation with different groups; and/or captured articles or reports published local languages. We do not view these results on their own as definitively conclusive of superior (or inferior) index performance; rather, they are meant to contribute toward a better understanding of how different UHC service coverage indices may correspond with prior perceptions of different countries' UHC performance.

## Section 4. Convergent validity

For convergent validity, we estimated how much variation in healthy life expectancy (HALE) – a measure of overall population health – could be explained by each index after accounting for values of the Socio-demographic Index (SDI),[4,23] an indicator of overall development based on income per capita, educational attainment among populations aged 15 and older, and fertility rates among women under 25.

We viewed controlling for SDI's effect on HALE before assessing the relationship between HALE and UHC service coverage measures as necessary due to the high co-linearity of SDI with these metrics (eg, $r$=0.84 for SDI and HALE in 2019; $r$=0.87 for SDI and the health gains weighted UHC effective coverage index in 2019); Figures 3.1–3.5 depict the relationships between each index, SDI, and HALE. In an effort to avoid exaggerating the relationship between HALE and measures of UHC service coverage, we focused on the extent to which each index explained variation on HALE above and beyond what was associated with SDI.

To do this, we regressed country-level values of SDI in 2019, as estimated for GBD 2019, on corresponding estimates of HALE (also from GBD 2019). Second, we took the residuals from the first model – ie, variations in HALE not explained by SDI – and then regressed country-level index values against those residuals to determine how much of HALE could be explained by UHC service coverage performance. This two-step regression was conducted for each index separately, dropping corresponding values of SDI and HALE for indices that had fewer than the 204 countries and territories included in GBD 2019 (ie, 195 for GBD 2017, 183 for UHC SCI, 111 for the SC index). As also reported in Table 1 in the main manuscript, $R^2$ values – or how much UHC service coverage measures explained variation in HALE after accounting for SDI – spanned from 0.073 for the GBD 2019 UHC effective coverage index with health gains weighting to 0.010 for the World Bank SC index.

| **Index (year reported)** | **Source** | **Convergent validity (variation of HALE explained, accounting for SDI)** | | |
|---|---|---|---|---|
| | | Beta coefficient | Standard error | $R^2$ |
| UHC effective coverage index, health gains weighted (2019) | GBD 2019 | 5.00 | 1.72 | 0.073 |
| UHC effective coverage index, unweighted average (2019) | GBD 2019 | 4.19 | 1.49 | 0.068 |
| UHC service coverage index for SDGs (2017) | GBD 2017 | 4.30 | 1.76 | 0.053 |
| UHC SCI for SDG indicator 3.8.1 (2017) | WHO 2019 | 4.21 | 1.88 | 0.044 |
| Service coverage index (most recent year) | World Bank 2020 | 1.24 | 1.18 | 0.010 |

## References


1 World Health Organization (WHO). Primary Health Care on the Road to Universal Health Coverage: 2019 Monitoring Report. Geneva, Switzerland: WHO, 2019.

2 Wagstaff A, Neelsen S. A comprehensive assessment of universal health coverage in 111 countries: a retrospective observational study. *The Lancet Global Health* 2020; **8**: e39–49.

3 Wagstaff, A. (World Bank), Sven Neelsen (World Bank). A comprehensive assessment of universal health coverage in 111 countries: a retrospective observational study. 2020; published online Jan. https://datacatalog.worldbank.org/dataset/comprehensive-assessment-universal-health-coverage-111-countries-retrospective-observational.

4 GBD 2019 Demographics Collaborators. Global age-sex-specific fertility, mortality, healthy life expectancy, (HALE), and population estimates in 204 countries and territories, 1950–2019: a comprehensive demographic analysis for the Global Burden of Disease Study 2019. *The Lancet* In press.

5 GBD 2019 Diseases and Injuries Collaborators. Global burden of 369 diseases and injuries in 204 countries and territories, 1990–2019: a systematic analysis for the Global Burden of Disease Study 2019. *The Lancet* In press.

6 GBD 2019 Risk Factors Collaborators. Global burden of 87 risk factors in 204 countries and territories, 1990–2019; a systematic analysis for the Global Burden of Disease Study 2019. *The Lancet* In press.

7 Lozano R, Fullman N, Abate D, *et al.* Measuring progress from 1990 to 2017 and projecting attainment to 2030 of the health-related Sustainable Development Goals for 195 countries and territories: a systematic analysis for the Global Burden of Disease Study 2017. *The Lancet* 2018; **392**: 2091–138.

8 UN Statistical Commission. SDG indicator 3.8.1 metadata. 2018 https://unstats.un.org/sdgs/metadata/files/Metadata-03-08-01.pdf.

9 Fullman N, Barber RM, Abajobir AA, *et al.* Measuring progress and projecting attainment on the basis of past trends of the health-related Sustainable Development Goals in 188 countries: an analysis from the Global Burden of Disease Study 2016. *The Lancet* 2017; **390**: 1423–59.

10 Hogan DR, Stevens GA, Hosseinpoor AR, Boerma T. Monitoring universal health coverage within the Sustainable Development Goals: development and baseline data for an index of essential health services. *The Lancet Global Health* 2018; **6**: e152–68.

11 World Health Organization (WHO), World Bank. Tracking universal health coverage: 2017 Global Monitoring Report. Geneva, Switzerland; Washington, DC: WHO, World Bank, 2017 https://www.who.int/healthinfo/universal_health_coverage/report/2017/en/.

12 Wagstaff A, Dmytraczenko T, Almeida G, *et al.* Assessing Latin America's progress toward achieving universal health coverage. *Health Affairs* 2015; **34**: 1704–12.

13 Marten R, McIntyre D, Travassos C, *et al.* An assessment of progress towards universal health coverage in Brazil, Russia, India, China, and South Africa (BRICS). *The Lancet* 2014; **384**: 2164–71.

14 Atun R, de Andrade LOM, Almeida G, *et al.* Health-system reform and universal health coverage in Latin America. *The Lancet* 2015; **385**: 1230–47.

15 Aguilera X, Castillo-Laborde C, Ferrari MN-D, Delgado I, Ibañez C. Monitoring and Evaluating Progress towards Universal Health Coverage in Chile. *PLoS Med* 2014; **11**. DOI:10.1371/journal.pmed.1001676.

16 de Andrade LOM, Filho AP, Solar O, *et al.* Social determinants of health, universal health coverage, and sustainable development: case studies from Latin American countries. *The Lancet* 2015; **385**: 1343–51.

17 Reich MR, Harris J, Ikegami N, *et al.* Moving towards universal health coverage: lessons from 11 country studies. *The Lancet* 2016; **387**: 811–6.

18 Nyonator F, Ofosu A, Segbafah M, d'Almeida S. Monitoring and Evaluating Progress towards Universal Health Coverage in Ghana. *PLOS Medicine* 2014; **11**: e1001691.

19 Chemouni B. The political path to universal health coverage: Power, ideas and community-based health insurance in Rwanda. *World Development* 2018; **106**: 87–98.

20 Tan KB, Tan WS, Bilger M, Ho CWL. Monitoring and Evaluating Progress towards Universal Health Coverage in Singapore. *PLOS Medicine* 2014; **11**: e1001695.

21 Tangcharoensathien V, Limwattananon S, Patcharanarumol W, Thammatacharee J. Monitoring and Evaluating Progress towards Universal Health Coverage in Thailand. *PLOS Medicine* 2014; **11**: e1001726.

22 Savedoff WD, Smith AL. Achieving Universal Health Coverage: Learning from Chile, Japan, Malaysia, and Sweden. Results for Development (R4D), 2011 https://www.r4d.org/resources/achieving-universal-health-coverage-learning-chile-japan-malaysia-sweden/.

23 Murray CJL, Callender CSKH, Kulikoff XR, *et al.* Population and fertility by age and sex for 195 countries and territories, 1950–2017: a systematic analysis for the Global Burden of Disease Study 2017. *The Lancet* 2018; **392**: 1995–2051.

**Table 3.1 Mapping multi-country UHC health service coverage indicators against the UHC effective coverage measurement framework matrix of health service types and population-age groups.** Indicators that do not directly map to specific service types and/or population-age groups are listed in the farthest right column. The percentage of matrix cells covered are calculated based on whether at least 1 indicator occupies a given cell of health services for a population-age group. Indicators are not included for population-age groups in which at least some portion of the population-age group is excluded (eg, ceiling of 74 years will not map to ≥ 65). UHC=universal health coverage. GBD=Global Burden of Disease. MNCH=maternal, neonatal, and child health. NCDs=Non-communicable diseases. FP=family planning. ENMR= early neonatal mortality rate. MMR=maternal mortality ratio. MIR=mortality-to-incidence ratio. MPR=mortality-to-prevalence ratio. RSDR=risk-standardised death rate. DTP=diptheria-tetanus-pertussis vaccine. MCV=measles-containing vaccine. ITN=insecticide-treated net. ART=antiretroviral therapy. TB=tuberculosis. ANC=antenatal care. SBA=skilled birth attendance. LRI=lower respiratory infection. URI=upper respiratory infection. IHD=ischaemic heart disease. RHD=rheumatic heart disease. HHD=hypertensive heart disease. CKD=chronic kidney disease. COPD=chronic obstructive pulmonary disease. BP=blood pressure. FPG=fasting-plasma glucose. IHR=International Health Regulations. ORS=oral rehydration solution. BCG=Bacille Calmette-Guerin vaccine.

| Population-age group | Health service type | | | | | | |
|---|---|---|---|---|---|---|---|
| | Promotion | Prevention | Treatment: Communicable diseases and MNCH | Treatment: NCDs | Rehabilitation | Palliation | Not mappable to specific health service types |
| **UHC effective coverage index (GBD 2019)** | **40% (12 of 30 matrix cells)** | | | | | | |
| Reproductive and newborn | Met need for FP with modern contraception | Antenatal, peripartum, and postnatal care for newborns based on ENMR<br>Antenatal, peripartum, and postnatal care for mothers based on MMR | | | | | |
| Children under 5 years | | DTP3 coverage<br>MCV1 coverage | LRI treatment based on MIR<br>Diarrhea treatment based on MIR | Leukaemia treatment based on MIR | | | |
| Children and adolescents (5-19 years) | | | ART coverage | Leukaemia treatment based on MIR<br>Asthma treatment based on MPR<br>Epilepsy treatment based on MPR<br>Appendicitis treatment based on MIR<br>Paralytic ileus and intestinal obstruction treatment based on MIR | | | |
| Adults (20-64 years) | | | ART coverage<br>TB treatment based on MIR | Diabetes treatment based on MPR<br>IHD treatment based on RSDR<br>Stroke treatment based on MIR<br>CKD treatment based on MPR<br>COPD treatment based on MPR<br>Cervical cancer treatment based on MIR<br>Breast cancer treatment based on MIR<br>Uterine cancer treatment based on MIR<br>Colon/rectum cancer treatment based on MIR<br>Epilepsy treatment based on MPR<br>Appendicitis treatment based on MIR<br>Paralytic ileus and intestinal obstruction treatment based on MIR | | | |
| Older adults (≥ 65 years) | | | ART coverage<br>TB treatment based on MIR | Diabetes treatment based on MPR<br>IHD treatment based on RSDR<br>Stroke treatment based on MIR<br>CKD treatment based on MPR<br>COPD treatment based on MPR<br>Cervical cancer treatment based on MIR<br>Breast cancer treatment based on MIR<br>Uterine cancer treatment based on MIR<br>Colon/rectum cancer treatment based on MIR<br>Epilepsy treatment based on MPR<br>Appendicitis treatment based on MIR<br>Paralytic ileus and intestinal obstruction treatment based on MIR | | | |
| **UHC service coverage index (GBD 2017)** | **33% (10 of 30 matrix cells)** | | | | | | |
| Reproductive and newborn | Met need for FP with modern contraception | ANC1 coverage<br>ANC4 coverage<br>Neonatal care based on RSDR<br>Maternal care based on RSDR | SBA coverage<br>In-facility delivery rate<br>Neonatal care based on RSDR<br>Maternal care based on RSDR | | | | Avoidance of adverse effects of medical treatment based on RSDR |
| Children under 5 years | | DTP3 coverage<br>MCV1 coverage<br>Polio3 coverage<br>Diphtheria prevention based on RSDR<br>Pertussis prevention based on RSDR<br>Tetanus prevention based on RSDR<br>Measles prevention based on RSDR | Diarrhea treatment based on RSDR<br>LRI treatment based on RSDR<br>URI treatment based on RSDR | Treatment of congenital heart anomalies based on RSDR | | | |
| Children and adolescents (5-19 | | | ART coverage<br>LRI treatment based on RSDR<br>URI treatment based on RSDR | Treatment of leukaemia based on MIR<br>Treatment of asthma based on RSDR | | | |

| Population-age group | Promotion | Prevention | Treatment: Communicable diseases and MNCH | Treatment: NCDs | Rehabilitation | Palliation | Not mappable to specific health service types |
|---|---|---|---|---|---|---|---|
| Adults (20-64 years) | | | ART coverage<br>LRI treatment based on RSDR<br>URI treatment based on RSDR | Treatment of colon/rectum cancer based on MIR<br>Treatment of breast cancer based MIR<br>Treatment of cervical cancer based on MIR<br>Treatment of uterine cancer based on MIR<br>Treatment of non-melanoma skin cancer based on MIR<br>Treatment of testicular cancer based on MIR<br>Treatment of Hodgkin's lymphoma based on MIR<br>Treatment of RHD based on RSDR<br>Treatment of IHD based on RSDR<br>Treatment of stroke based on RSDR<br>Treatment of HHD based on RSDR<br>Treatment of diabetes based on RSDR<br>Treatment of CKD based on RSDR<br>Treatment of epilepsy based on RSDR<br>Surgical care for peptic ulcer disease based on RSDR<br>Surgical care for appendicitis based on RSDR<br>Surgical care for paralytic ileus and intestinal obstruction based on RSDR<br>Surgical care for gallbladder and biliary diseases based on RSDR | | | |
| Older adults (≥ 65 years) | | | | | | | |
| **UHC SCI, SDG 3.8.1 indicator (WHO)** | **20% (6 of 30 matrix cells)** | | | | | | |
| Reproductive and newborn | Met need for FP with modern contraception | ANC4 coverage | | | | | Basic sanitation; prevalence of non-smoking; hospital beds per 1,000; health worker density (physicians, surgeons, psychiatrists); IHR score |
| Children under 5 years | | DTP3 coverage<br>ITN use by children under 5 (for high malaria burden countries) | Care-seeking for ARI | | | | |
| Children and adolescents (5-19 years) | | | | | | | |
| Adults (20-64 years) | | | ART coverage<br>TB case detection and treatment | Prevalence of non-elevated BP<br>Mean FPG | | | |
| Older adults (≥ 65 years) | | | | | | | |
| **SC index (World Bank 2020)** | **17% (5 of 30 matrix cells)** | | | | | | |
| Reproductive and newborn | | ANC4 coverage | SBA coverage | | | | Inpatient admission rates |
| Children under 5 years | | Full vaccination (DTP3, MCV1, BCG, Polio3) | Care-seeking for ARI<br>ORS treatment for diarrhea | | | | |
| Children and adolescents (5-19 years) | | | | | | | |
| Adults (20-64 years) | | Breast cancer screening rates<br>Cervical cancer screening rates | | | | | |
| Older adults (≥ 65 years) | | | | | | | |

**Table 3.2. Known-group validity results based on 16 country-pair comparisons on UHC service coverage measures.** Based on previous literature and expert inputs, Country A was expected to have higher performance on UHC service coverage than Country B. Mean index values are reported for the following: UHC effective coverage index in 2019, both with health gains weights and the unweighted average (GBD 2019); UHC service coverage index for the health-related SDGs in 2017 (GBD 2017); and UHC SCI, the SDG 3.8.1 measure, in 2017 (WHO 2019); and service coverage (SC) index for most recent years (World Bank 2020). GBD-based indices have estimates of uncertainty, which we include and also assess Country A versus Country B performance on the basis of uncertainty (i.e., no overlap in uncertainty bounds). The WHO and World Bank indices to not do report estimates of uncertainty accompanying their mean index values. UHC=universal health coverage. GBD=Global Burden of Disease. SDGs=Sustainable Development Goals. SCI=Service coverage index.

| Known-group validity country pairs | | Index values | | Validity indicators | | |
|---|---|---|---|---|---|---|
| **Country A** | **Country B** | **Country A** | **Country B** | **A > B (mean)** | **A > B (with uncertainty)** | **A / B (mean)** |
| **UHC effective coverage index for 2019, with health gains weights (GBD 2019)** | | | | **94% (15/16)** | **63% (10/16)** | |
| Brazil | Paraguay | 64.8 (63.0 to 66.7) | 63.4 (58.0 to 68.3) | Yes | No | 1.02 |
| Chile | Ecuador | 74.6 (69.7 to 77.8) | 64.5 (60.4 to 68.4) | Yes | Yes | 1.16 |
| Colombia | Plurinational State of Bolivia | 74.5 (69.3 to 79.0) | 52.5 (47.2 to 58.1) | Yes | Yes | 1.42 |
| Costa Rica | El Salvador | 79.1 (74.2 to 83.1) | 61.7 (56.3 to 66.6) | Yes | Yes | 1.28 |
| Cuba | Dominican Republic | 72.5 (69.0 to 76.4) | 52.7 (46.6 to 58.0) | Yes | Yes | 1.38 |
| Czechia | Belarus | 82.1 (77.6 to 85.9) | 70.5 (66.7 to 73.8) | Yes | Yes | 1.16 |
| Ethiopia | Democratic Republic of the Congo | 46.5 (42.3 to 51.1) | 45.1 (39.5 to 51.1) | Yes | No | 1.03 |
| France | United States | 91.0 (86.6 to 93.6) | 82.2 (80.4 to 83.6) | Yes | Yes | 1.11 |
| Ghana | Cote d'Ivoire | 49.2 (45.5 to 53.1) | 42.7 (37.8 to 49.3) | Yes | No | 1.15 |
| Japan | Portugal | 96.4 (95.0 to 97.4) | 83.8 (80.3 to 85.8) | Yes | Yes | 1.15 |
| Malaysia | Indonesia | 66.6 (62.3 to 70.7) | 48.6 (42.2 to 54.9) | Yes | Yes | 1.37 |
| Rwanda | Burundi | 59.3 (54.5 to 64.4) | 50.0 (43.5 to 55.8) | Yes | No | 1.19 |
| Singapore | Brunei | 92.6 (90.1 to 93.9) | 65.6 (62.6 to 67.9) | Yes | Yes | 1.41 |
| Spain | Peru | 90.3 (87.1 to 91.6) | 76.0 (70.4 to 79.9) | Yes | Yes | 1.19 |
| Thailand | Sri Lanka | 71.6 (66.3 to 76.6) | 65.6 (59.7 to 71.2) | Yes | No | 1.09 |
| Turkey | Islamic Republic of Iran | 69.2 (64.6 to 73.9) | 69.6 (67.4 to 71.4) | No | No | 0.99 |
| **UHC effective coverage index for 2019, unweighted average (GBD 2019)** | | | | **94% (15/16)** | **56% (9/16)** | |
| Brazil | Paraguay | 64.5 (62.6 to 66.4) | 62.7 (57.8 to 67.0) | Yes | No | 1.03 |
| Chile | Ecuador | 76.5 (72.0 to 79.5) | 64.9 (60.5 to 68.1) | Yes | Yes | 1.18 |
| Colombia | Plurinational State of Bolivia | 75.5 (70.6 to 79.3) | 52.4 (47.2 to 57.2) | Yes | Yes | 1.44 |
| Costa Rica | El Salvador | 79.7 (75.4 to 83.0) | 66.5 (61.9 to 70.5) | Yes | Yes | 1.20 |
| Cuba | Dominican Republic | 77.2 (73.6 to 80.4) | 55.5 (50.3 to 60.0) | Yes | Yes | 1.39 |
| Czechia | Belarus | 85.4 (81.6 to 88.0) | 79.8 (76.2 to 82.5) | Yes | No | 1.07 |
| Ethiopia | Democratic Republic of the Congo | 31.2 (26.5 to 37.8) | 39.0 (34.4 to 43.2) | No | No | 0.80 |
| France | United States | 87.9 (84.0 to 90.3) | 84.7 (82.9 to 86.1) | Yes | No | 1.04 |
| Ghana | Cote d'Ivoire | 43.5 (38.9 to 47.7) | 40.1 (35.2 to 44.9) | Yes | No | 1.08 |

| Known-group validity country pairs | | Index values | | Validity indicators | | |
|---|---|---|---|---|---|---|
| Country A | Country B | Country A | Country B | A > B (mean) | A > B (with uncertainty) | A / B (mean) |
| Japan | Portugal | 93.8 (92.6 to 94.7) | 86.4 (83.1 to 88.2) | Yes | Yes | 1.09 |
| Malaysia | Indonesia | 68.2 (64.1 to 71.5) | 44.9 (38.6 to 52.4) | Yes | Yes | 1.52 |
| Rwanda | Burundi | 45.7 (41.1 to 50.2) | 35.3 (29.6 to 41.0) | Yes | Yes | 1.29 |
| Singapore | Brunei | 92.6 (89.9 to 94.0) | 67.2 (63.9 to 69.7) | Yes | Yes | 1.38 |
| Spain | Peru | 90.8 (87.5 to 92.2) | 73.6 (68.5 to 77.2) | Yes | Yes | 1.23 |
| Thailand | Sri Lanka | 72.4 (67.7 to 75.9) | 69.9 (65.5 to 73.8) | Yes | No | 1.04 |
| Turkey | Islamic Republic of Iran | 70.3 (66.7 to 73.5) | 70.2 (68.2 to 72.0) | Yes | No | 1.00 |
| **UHC service coverage index for the health-related SDGs for 2017 (GBD 2017)** | | | | **94% (15/16)** | **69% (11/16)** | |
| Brazil | Paraguay | 70.0 (69.4 to 70.7) | 69.6 (66.7 to 72.4) | Yes | No | 1.01 |
| Chile | Ecuador | 81.1 (79.2 to 83.1) | 67.9 (66.0 to 69.7) | Yes | Yes | 1.19 |
| Colombia | Plurinational State of Bolivia | 74.8 (73.0 to 76.7) | 61.0 (57.8 to 65.0) | Yes | Yes | 1.23 |
| Costa Rica | El Salvador | 76.7 (75.5 to 77.8) | 70.1 (67.1 to 73.0) | Yes | Yes | 1.09 |
| Cuba | Dominican Republic | 78.1 (76.2 to 79.9) | 64.3 (61.4 to 66.8) | Yes | Yes | 1.21 |
| Czechia | Belarus | 88.6 (87.7 to 89.6) | 83.6 (82.0 to 84.9) | Yes | Yes | 1.06 |
| Ethiopia | Democratic Republic of the Congo | 44.2 (41.4 to 47.1) | 43.9 (40.5 to 47.1) | Yes | No | 1.01 |
| France | United States | 93.6 (92.7 to 94.4) | 87.8 (87.3 to 88.2) | Yes | Yes | 1.07 |
| Ghana | Cote d'Ivoire | 57.1 (54.2 to 60.0) | 50.3 (47.5 to 53.3) | Yes | Yes | 1.14 |
| Japan | Portugal | 93.7 (93.2 to 94.1) | 90.2 (89.1 to 91.1) | Yes | Yes | 1.04 |
| Malaysia | Indonesia | 72.9 (71.1 to 74.7) | 61.8 (60.6 to 63.1) | Yes | Yes | 1.18 |
| Rwanda | Burundi | 50.1 (46.5 to 54.1) | 45.1 (41.3 to 49.2) | Yes | No | 1.11 |
| Singapore | Brunei | 92.4 (91.7 to 93.2) | 76.5 (74.9 to 78.0) | Yes | Yes | 1.21 |
| Spain | Peru | 93.6 (92.9 to 94.3) | 71.9 (69.5 to 74.4) | Yes | Yes | 1.30 |
| Thailand | Sri Lanka | 76.4 (74.6 to 78.4) | 77.1 (74.4 to 79.8) | No | No | 0.99 |
| Turkey | Islamic Republic of Iran | 77.8 (76.2 to 79.3) | 76.7 (75.3 to 77.9) | Yes | No | 1.01 |
| **UHC SCI for 2017, SDG 3.8.1 measure (WHO 2019)** | | | | **75% (12/16)** | **-** | |
| Brazil | Paraguay | 78.6 | 68.5 | Yes | - | 1.15 |
| Chile | Ecuador | 70.2 | 76.5 | No | - | 0.92 |
| Colombia | Plurinational State of Bolivia | 75.9 | 68.3 | Yes | - | 1.11 |
| Costa Rica | El Salvador | 77.0 | 75.6 | Yes | - | 1.02 |
| Cuba | Dominican Republic | 82.8 | 74.3 | Yes | - | 1.12 |
| Czechia | Belarus | 76.0 | 76.5 | No | - | 0.99 |
| Ethiopia | Democratic Republic of the Congo | 39.4 | 41.5 | No | - | 0.95 |
| France | United States | 77.7 | 83.9 | No | - | 0.93 |
| Ghana | Cote d'Ivoire | 47.4 | 47.3 | Yes | - | 1.00 |
| Japan | Portugal | 83.1 | 81.5 | Yes | - | 1.02 |
| Malaysia | Indonesia | 73.3 | 57.3 | Yes | - | 1.28 |

| Known-group validity country pairs | | Index values | | Validity indicators | | |
|---|---|---|---|---|---|---|
| **Country A** | **Country B** | **Country A** | **Country B** | **A > B (mean)** | **A > B (with uncertainty)** | **A / B (mean)** |
| Rwanda | Burundi | 56.9 | 41.6 | Yes | - | 1.37 |
| Singapore | Brunei | 85.8 | 81.5 | Yes | - | 1.05 |
| Spain | Peru | 82.7 | 76.9 | Yes | - | 1.08 |
| Thailand | Sri Lanka | 79.8 | 66.0 | Yes | - | 1.21 |
| Turkey | Islamic Republic of Iran | 74.4 | 71.7 | Yes | - | 1.04 |
| **SC index for most recent year (World Bank 2020)** | | | | **56% (9/16)** | **-** | |
| Brazil | Paraguay | 75.4 | 52.5 | Yes | - | 1.44 |
| Chile | Ecuador | 83.3 | 54.8 | Yes | - | 1.52 |
| Colombia | Plurinational State of Bolivia | 65.3 | - | No | - | - |
| Costa Rica | El Salvador | 63.1 | 48.1 | Yes | - | 1.31 |
| Cuba | Dominican Republic | - | 73.6 | No | - | - |
| Czechia | Belarus | 92.0 | - | No | - | - |
| Ethiopia | Democratic Republic of the Congo | 16.2 | 33.1 | No | - | 0.49 |
| France | United States | 93.1 | 89.5 | Yes | - | 1.04 |
| Ghana | Cote d'Ivoire | 46.1 | 15.1 | Yes | - | 3.05 |
| Japan | Portugal | 70.2 | 91.9 | No | - | 0.76 |
| Malaysia | Indonesia | 67.7 | 27.4 | Yes | - | 2.47 |
| Rwanda | Burundi | 38.2 | 29.8 | Yes | - | 1.28 |
| Singapore | Brunei | - | - | No | - | - |
| Spain | Peru | 86.2 | 52.5 | Yes | - | 1.64 |
| Thailand | Sri Lanka | 57.8 | 52.9 | Yes | - | 1.09 |
| Turkey | Islamic Republic of Iran | 31.9 | 66.6 | No | - | 0.48 |

**Figure 3.1. Comparing the the UHC effective coverage index in 2019 with health gains weighting to HALE (A) and SDI (B).** Locations are colour-coded by GBD super-region and abbreviated by ISO3 code. UHC=universal health coverage. GBD=Global Burden of Disease. SDI=Socio-democraphic index. HALE=healthy life expectancy.

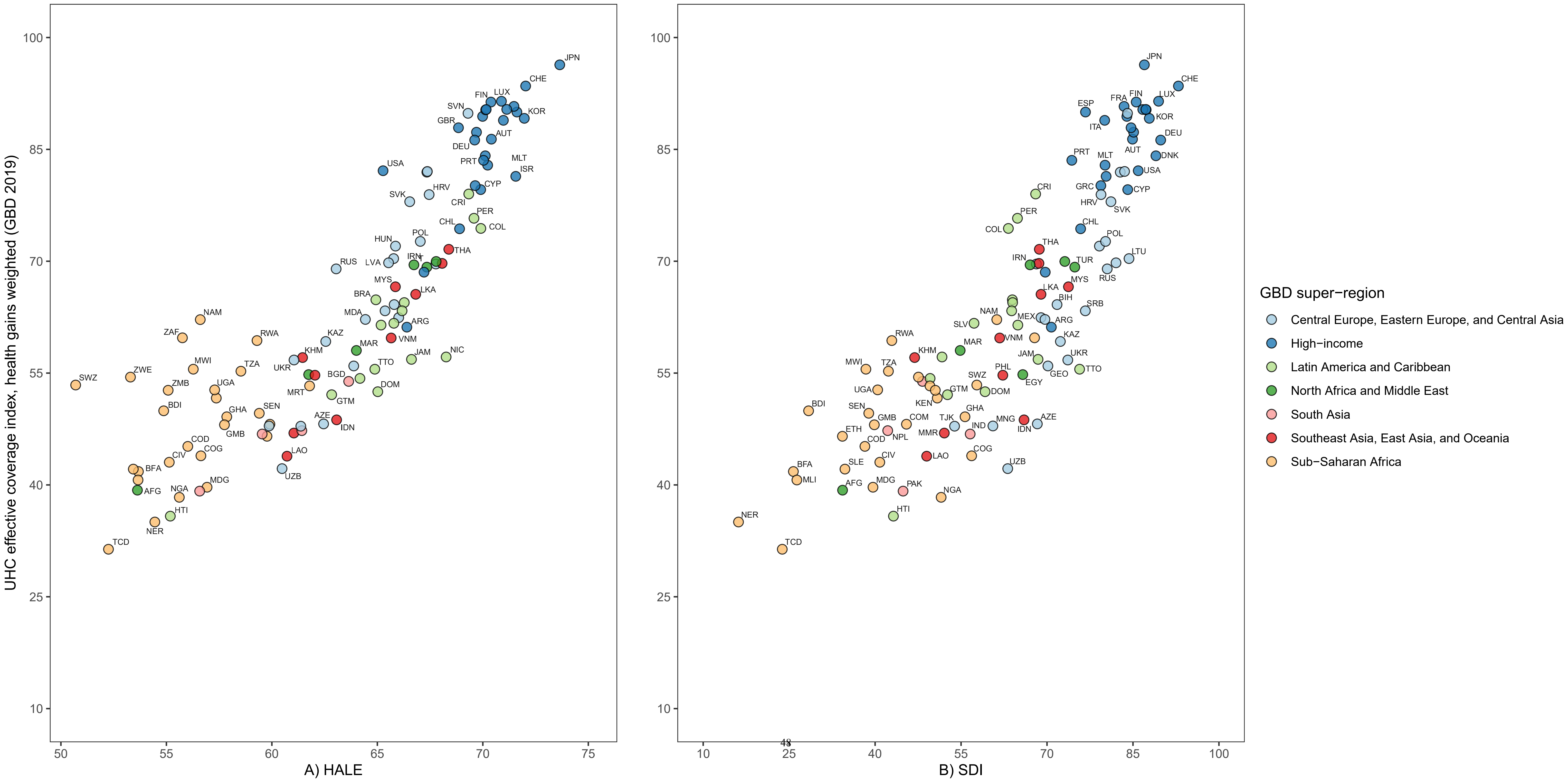

**Figure 3.2. Comparing the the UHC effective coverage index in 2019 (unweighted average) to HALE (A) and SDI (B).** Locations are colour-coded by GBD super-region and abbreviated by ISO3 code. UHC=universal health coverage. GBD=Global Burden of Disease. SDI=Socio-democraphic index. HALE=healthy life expectancy.

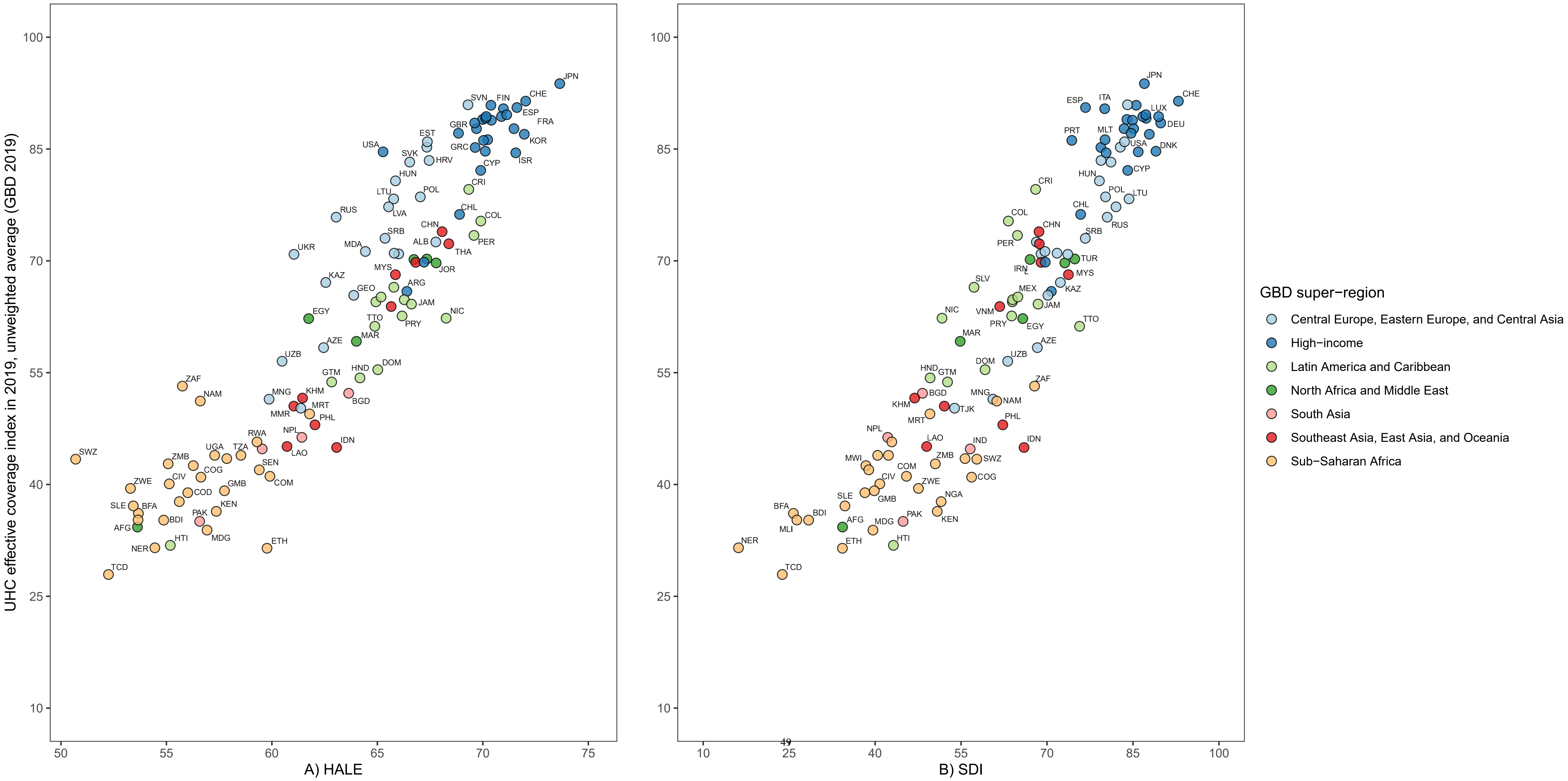

**Figure 3.3. Comparing the GBD 2017 UHC service coverage index in 2017 to HALE (A) and SDI (B).** Locations are colour-coded by GBD super-region and abbreviated by ISO3 code. UHC=universal health coverage. GBD=Global Burden of Disease. SDI=Socio-demographic index. HALE=healthy life expectancy

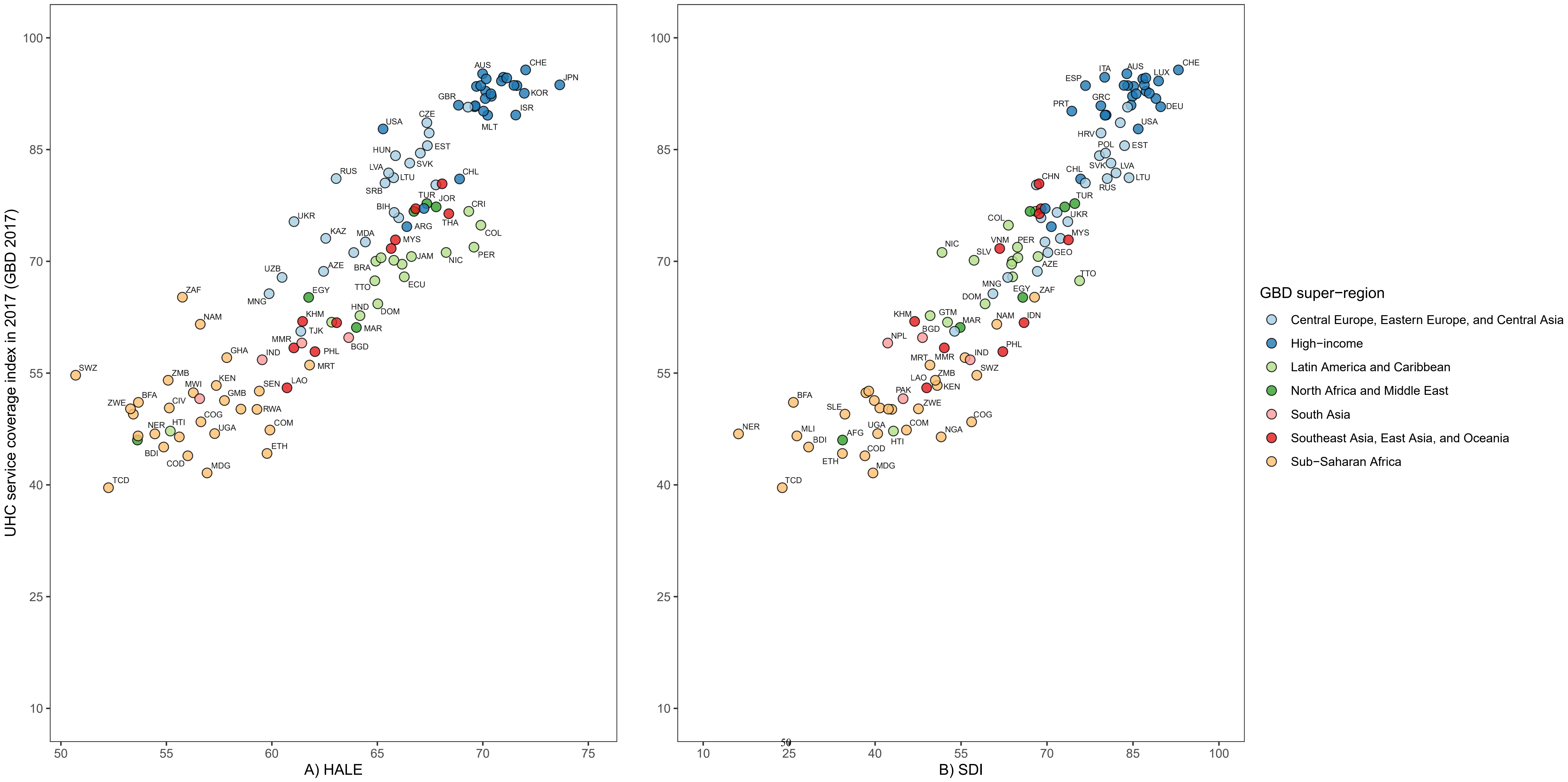

**Figure 3.4. Comparing the WHO UHC SCI in 2017 to HALE (A) and SDI (B)**. Locations are colour-coded by GBD super-region and abbreviated by ISO3 code. UHC=universal health coverage. SCI=service coveage index. GBD=Global Burden of Disease. SDI=Socio-democraphic index. HALE=healthy life expectancy.

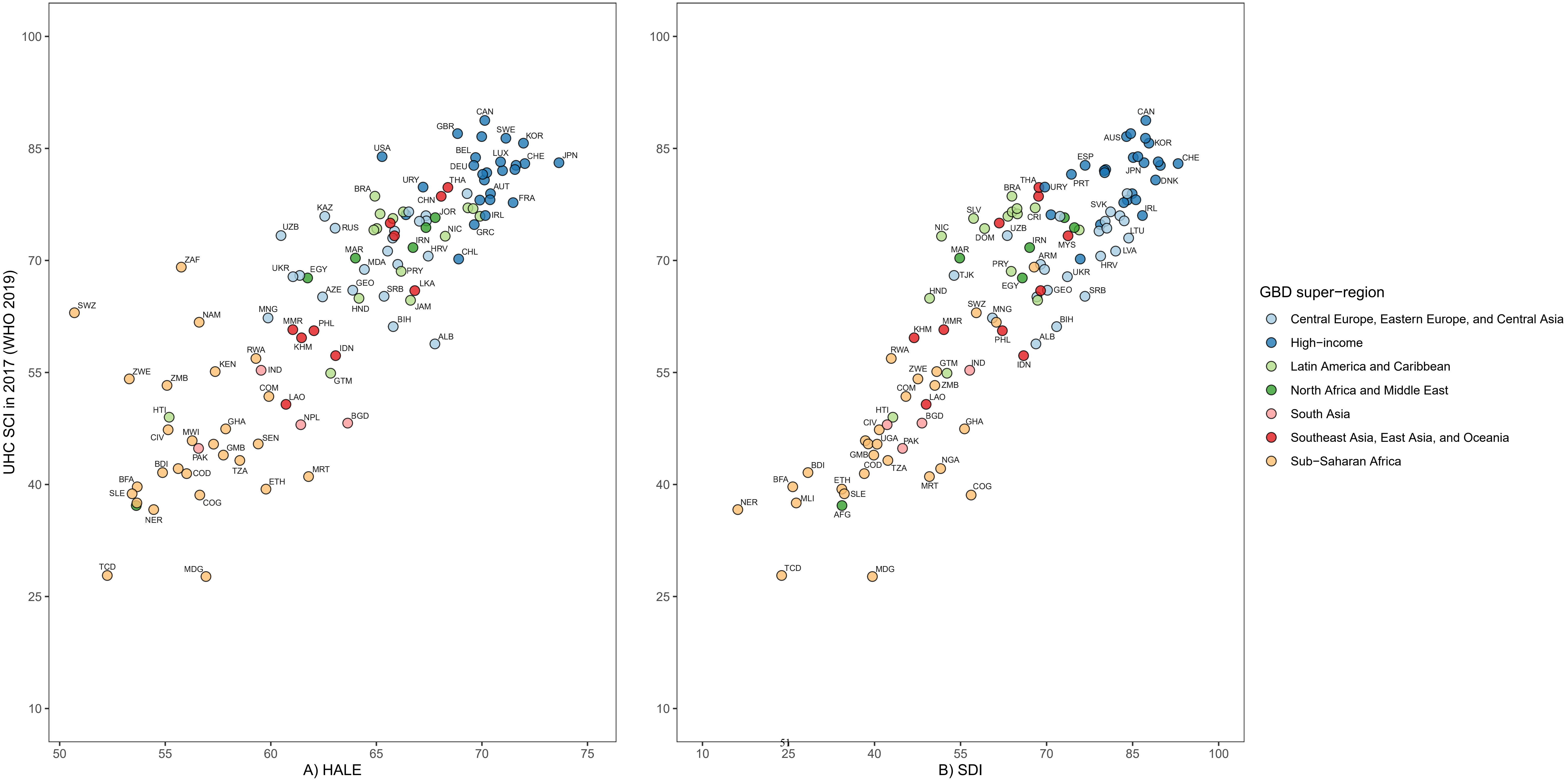

**Figure 3.5 Comparing the World Bank SC index (most recent year) to HALE (A) and SDI (B)**. Locations are colour-coded by GBD super-region and abbreviated by ISO3 code. UHC=universal health coverage. SCI=service coveage. GBD=Global Burden of Disease. SDI=Socio-democraphic index. HALE=healthy life expectancy.

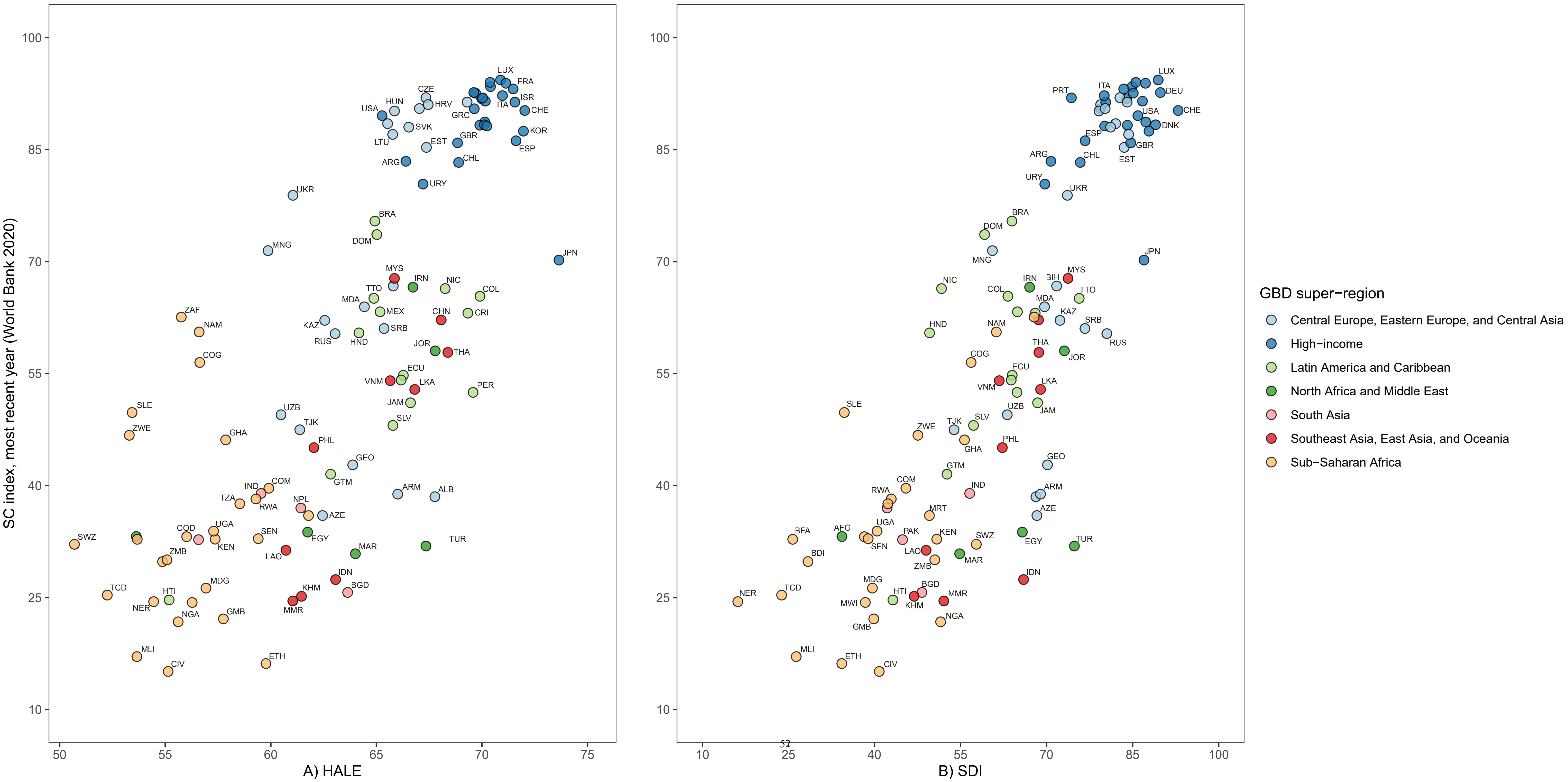

# Part 4. Frontier Analysis.

## Section 1. Overview

In our frontier analysis, we measured the impact of three types of health expenditure (pooled health spending, domestic pooled health spending, and total health expenditure) on the UHC Effective Coverage Index. In this analysis, pooled health spending is equal to total health expenditure minus out of pocket expenditure and domestic pooled health spending is equal to pooled health spending minus development assistance for health. Based on a priori knowledge, we expected that frontiers would monotonically increase and would exhibit diminishing returns. More explicitly, specifying a monotonically increasing function means that an increase in health expenditure will not decrease the maximum potential UHC effective coverage achievement. Additionally, the presumption of diminishing returns specifies that the contribution of an additional dollar of health expenditure per capita is more significant where spending levels are lower in the frontier. We incorporated both as constraints in the model specification of the frontiers. SFM analyses were run using third degree polynomial splines with six knots and the following constraints: linear tail, monotonically increasing curve, and concave curve. These constraints ensured that where data were sparse (for example, at very high levels of health expenditure), stochastic variation did not drive the flexible frontier toward two unrealistic trends. Firstly, these constraints avoid the incorrect insinuation that at certain level of health expenditure, additional health spending would be detrimental to UHC effective coverage attainment. Secondly, the constraints prevent extrapolation beyond observed levels of UHC effective coverage attainment to ensure scores cannot exceed 100. Given our use of modelled inputs, we did not trim any observations from the frontiers that were constructed. Each of the production frontiers provides an estimate of the theoretical maximum UHC effective coverage attainment under the assumption countries or territories are translating health spending into UHC at an optimal level of efficiency. By comparing observed UHC effective coverage performance to the frontier, our analysis identifies the productive inefficiencies in UHC effective coverage given a location's existing level of health spending. For example, a country with a relatively small pooled health spending may have a UHC effective coverage performance close to the theoretical maximum; therefore, the country has a high efficiency score. Conversely, a country with a relatively larger pooled health spending and higher UHC effective coverage score could have a lower efficiency score if they are not as close to the theoretical maximum. While the frontier is produced relative to different types of health expenditure, the productive inefficiency estimates implicitly entail location-specific capacities related to health system components and contexts beyond levels of health expenditure, as governance framework, human resources for health workforce distribution, transportation infrastructure, and social and gender norms. For more technical details, please see following technical supplement.

## Section 2. Stochastic frontier meta-analysis technical supplement

Stochastic frontier analysis (SFA) [1] is a stochastic analysis of the frontier production function, which expresses the maximum amount of output obtainable from a linear combination of variables of interest. The SFA model we start with is given by

$$y_i = \langle \boldsymbol{x}_i, \boldsymbol{\beta} \rangle - v_i, \tag{1}$$

where $y_i$ are observations, $\langle \boldsymbol{x}_i, \boldsymbol{\beta} \rangle$ is the linear model (linear combination of variables in $\boldsymbol{x}_i$ with weights $\boldsymbol{\beta}$), while $v_i$ is the deviation from the *maximum output*, and so is modeled as a *non-negative* random effect. In the context of Universal Health Coverage (UHC), the observations $y_i$ are measures of UHC, and the model $\langle \boldsymbol{x}_i, \boldsymbol{\beta} \rangle$ is a spline that relates health spending to UHC. The spline model is explained in detail in Section 2.3.

We develop a meta-analytic extension of SFA, which we call Stochastic Frontier Meta-analysis (SFM). Every observation $y_i$ is subject to random error (computed from aggregated data). We consider the modified model

$$\boldsymbol{y} = \boldsymbol{X}\boldsymbol{\beta}^* - \boldsymbol{v} + \boldsymbol{\epsilon}, \tag{2}$$

with each entry $v_i$ of $\boldsymbol{v}$ a half-normal non-negative random effect with unknown variance $\eta$, while each entry $\epsilon_i$ of $\boldsymbol{\epsilon}$ is Gaussian $\mathcal{N}(0, \sigma_i^2)$, and represents the reported study-specific error sources with known variances $\sigma_i^2$. In order to carry out the UHC analysis we introduce three innovations.

- We formulate the explicit likelihood problem for the SFM model, assuming a half-normal model for the non-negative random effects $v_i$.
- Outliers are a big problem for SFA [1]. We apply the trimmed robust approach [2] in order to automatically identify and remove outliers from each dataset.
- We allow priors and constraints for the SFM model. In particular this lets us incorporate shape constraints on the spline, similar to what was proposed by [8].

The resulting approach lets us model inherently nonlinear relationships through the linear model (2) using splines, remove outliers, and incorporate reported errors across geographic regions. Each of the pieces listed above is now described in detail.

# 1. SFM: modeling non-negative random effects.

In this section we derive all likelihood formulations for the Stochastic Frontier Meta-analysis (SFM) approach. We use the half-normal model for the random effects $v_i$:

$$f(v_i|\eta) = \begin{cases} \frac{\sqrt{2}}{\sqrt{\pi\eta}} \exp\left(-\frac{v_i^2}{2\eta}\right) & v_i \geq 0 \\ 0 & v_i < 0. \end{cases}$$

The goal is to estimate $\boldsymbol{\beta}^*$ and $\eta^*$ from observations. The mixed effects framework provides a natural statistical model which can be used for this inference. The joint distribution of fixed and random effects is then given by

$$\begin{aligned} p(\boldsymbol{\beta}, \eta, \boldsymbol{v}|\boldsymbol{y}) &= p(\boldsymbol{\beta}, \eta, |\boldsymbol{v}, \boldsymbol{y}) p(\boldsymbol{v}|\boldsymbol{y}) \\ &\propto \prod_{i=1}^m \frac{1}{\sqrt{\sigma_i^2 \eta}} \exp\left(-\frac{(y_i - \langle \boldsymbol{x}_i, \boldsymbol{\beta}\rangle + v_i)^2}{2\sigma_i^2}\right) \exp\left(-\frac{v_i^2}{2\eta}\right) \mathbb{1}_{\mathbb{R}_+}(v_i) \end{aligned} \tag{3}$$

Integrating out the random effects, and taking the negative log of the resulting distribution, we arrive at equivalent maximum likelihood formulation that does not depend on the random effects $\boldsymbol{v}$, but only depends on $\boldsymbol{\beta}$ and $\eta$. Define $\widetilde{\Phi}$ to be the complementary error function

$$\widetilde{\Phi}(z) = 1 - \frac{2}{\sqrt{\pi}} \int_0^z \exp(-t^2) dt.$$

Then we have the following closed form likelihood.

$$\begin{aligned} \mathcal{M}(\boldsymbol{\beta}, \eta|\boldsymbol{y}) &= -\ln\left(\int_{\mathbb{R}_+^m} p(\boldsymbol{\beta}, \eta, \boldsymbol{v}|\boldsymbol{y})\, d\boldsymbol{v}\right) \\ &= \sum_{i=1}^m \frac{(y_i - \langle \boldsymbol{x}_i, \boldsymbol{\beta}\rangle)^2}{2(\eta + \sigma_i^2)} + \frac{1}{2}\ln\left(\eta + \sigma_i^2\right) - \ln\ \widetilde{\Phi}\left(\frac{\sqrt{\eta}(y_i - \langle \boldsymbol{x}_i, \boldsymbol{\beta}\rangle)}{\sqrt{2(\eta + \sigma_i^2)(\sigma_i^2)}}\right) \end{aligned} \tag{4}$$

The SFM approach optimizes these likelihoods to estimate $(\boldsymbol{\beta}, \eta)$.

# 2. Priors, Constraints, and Splines.

In this section we describe how to set up Bayesian priors, constraints for parameters of interest, and spline models for nonlinear relationships in the SFM setup.

## 2.1. Priors

The likelihood $\mathcal{M}$ can be updated using prior information. Imposing priors is equivalent to adding penalties to the likelihood function. For the SFM analysis, the only priors we use are those related to the final section of the frontier.

Given a Gaussian prior on $\boldsymbol{\beta} \sim N(\overline{\boldsymbol{\beta}})$, we find the *a posteriori* estimate by solving the problem

$$\min_{\boldsymbol{\beta},\eta} \mathcal{M}(\boldsymbol{\beta},\eta) + \frac{1}{2}(\boldsymbol{\beta} - \overline{\boldsymbol{\beta}})^T \boldsymbol{\Sigma}_{\boldsymbol{\beta}}^{-1}(\boldsymbol{\beta} - \overline{\boldsymbol{\beta}}). \tag{5}$$

## 2.2. Constraints

We allow box constraints and general linear inequality constraints on $(\boldsymbol{\beta},\eta)$. Taking (5) as a running example, we can impose constraints of the form

$$\begin{aligned} \min_{\boldsymbol{\beta},\eta} \quad & \mathcal{M}(\boldsymbol{\beta},\eta) + \frac{1}{2}(\boldsymbol{\beta} - \overline{\boldsymbol{\beta}})^T \boldsymbol{\Sigma}_{\boldsymbol{\beta}}^{-1}(\boldsymbol{\beta} - \overline{\boldsymbol{\beta}}) + \frac{1}{2}(\eta - \overline{\eta})^T \boldsymbol{\Sigma}_{\eta}^{-1}(\eta - \overline{\eta}) \\ \text{such that} \quad & \boldsymbol{l}_f \le \begin{bmatrix} \boldsymbol{\beta} \\ \eta \end{bmatrix} \le \boldsymbol{u}_f, \quad \boldsymbol{C} \begin{bmatrix} \boldsymbol{\beta} \\ \eta \end{bmatrix} \le \boldsymbol{c}, \end{aligned} \tag{6}$$

where $(\boldsymbol{l}_f, \boldsymbol{u}_f)$ are lower and upper bounds on the variables, while $\boldsymbol{C}$ is any matrix. This functionality can be used to impose shape constraints on spline models, including increasing/decreasing, convex/concave, and combinations of these designs.

## 2.3. Splines

In this section we discuss spline models for dose-response relationships. For general background on splines and spline regression see [5] and [6].

**B-splines and bases.** A spline basis is a set of piecewise polynomial functions with designated degree and domain. If we denote polynomial order by $p$, and the number of knots by $k$, we need $p + k$ basis elements $s_j^p$, which can be generated recursively as illustrated in Figure 1.

Given such a basis, we can represent any dose-response relationship as the linear combination of the spline basis elements, with coefficients $\boldsymbol{\beta} \in \mathbb{R}^{p+k}$:

$$f(t) = \sum_{j=1}^{p+k} \beta_j^p s_j^p(t). \tag{7}$$

An explicit representation of (7) is obtained by building a design matrix $\mathbf{X}$. Given a set of $t$ values at which we have data, the $j$th column of $\mathbf{X}$ is given by the expression

$$\mathbf{X}_{\cdot,j} = \begin{bmatrix} s_j^p(t_0) \\ \vdots \\ s_j^p(t_k) \end{bmatrix}.$$

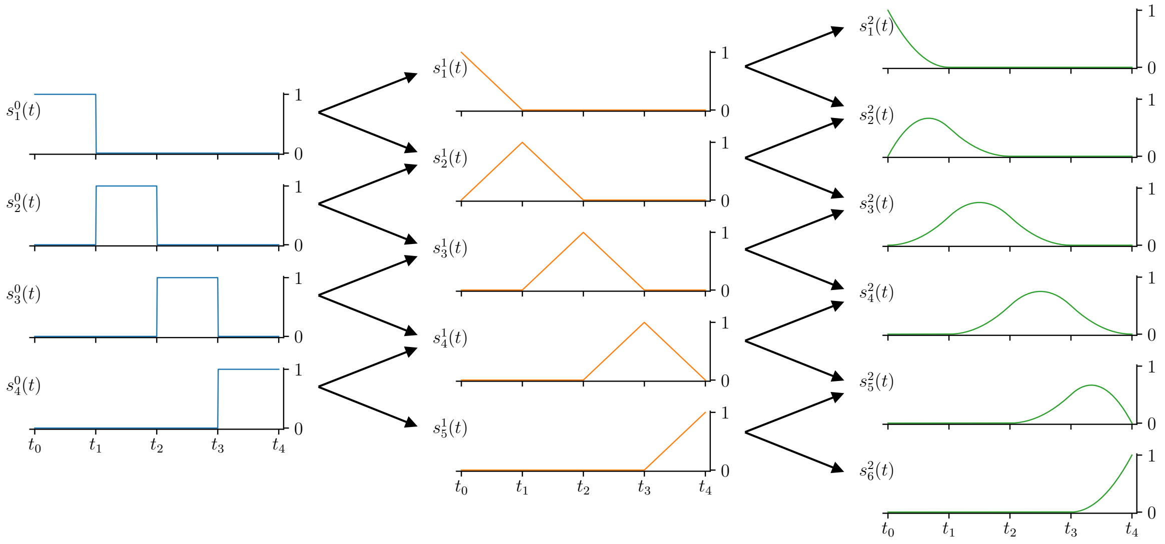


**Figure 1.** Recursive generation of bspline basis elements (orders 0, 1, 2).

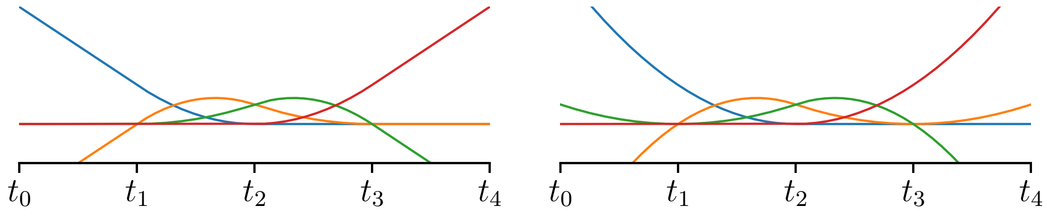


**Figure 2.** Spline extrapolation. Left: linear extrapolation. Right: nonlinear extrapolation.

The model for direct observations data coming from the spline (7) can now be written compactly as

$$\boldsymbol{y} = \mathbf{X}\boldsymbol{\beta} + \boldsymbol{v} + \boldsymbol{\epsilon}_i,$$

and has the same form as (1).

**Enforcing linear tails.** For the frontier analysis, we need to ensure that the last segment of the spline does not go above a theoretical limit, typically set at $1$. To do this, we allow an option to make the last segment linear. The prior capabilities can then be used to set a prior for the slope of this segment to be $0$ (i.e. flat). The estimated spline is then a best fit to the data, subject to this specification.

In general, using linear head and/or tail pieces to extrapolate outside the original domain or interpolate in the data sparse region is far more stable that using higher order polynomials, see Figure 2. The figure shows symmetric linear tail modifications, but for the analyses in the paper we only impose a right linear tail shape constraint.

**Shape constraints.** We can use constraints to enforce monotonicity, convexity, and concavity. Monotonicity across the domain of interest follows from monotonicity of the spline coefficients. This relationship is derived for particular basis constructions by [5], and has been used in the literature to enforce shape constraints [8]. Current approaches work around the natural inequality constraints by using additional 'exponentiated' variables. Instead we impose these constraints directly as described below.

Focusing just on
, the relationship
$_1 \leq$
$_2$ can be written as
$_1 -$
$_2 \leq 0$. Stacking these inequality

constraints for each pair $(\alpha_i, \alpha_{i+1})$ we can write all constraints simultaneously as

$$\underbrace{\begin{bmatrix} 1 & -1 & 0 & \dots & 0 \\ 0 & 1 & -1 & \dots & 0 \\ \ddots & \ddots & \ddots & \ddots & \vdots \\ 0 & \dots & \dots & 1 & -1 \end{bmatrix}}_{\boldsymbol{C}} \begin{bmatrix} \alpha_1 \\ \alpha_2 \\ \alpha_3 \\ \vdots \\ \alpha_n \end{bmatrix} \le \begin{bmatrix} 0 \\ 0 \\ \vdots \\ 0 \end{bmatrix}.$$

These constraints are directly imposed through the IPOPT interface, along with any lower- and upper-limit constraints on $\alpha$.

**Convexity and Concavity.** For any $\mathcal{C}^2$ (twice continuously differentiable) function $f : \mathbb{R} \to \mathbb{R}$, convexity and concavity are captured by the signs of the second derivative. Specifically, $f$ is convex if $f''(t) \ge 0$ is everywhere, an concave if $f''(t) \le 0$ everywhere. We impose linear inequality constraints on the expressions for $f''(t)$ over each interval. We can therefore easily pick any of the eight shape combinations given in [8, Table 1], as well as imposing any other constraints on $\alpha$ (including bounds).

# 3. Robust Extension via Trimming

Trimming estimators is a general methodology for robust estimation [10, 2]. For convenience, define

$$\boldsymbol{\theta} = \begin{bmatrix} \beta \\ \eta \end{bmatrix}.$$

Given any likelihood problem of form

$$\min_{\boldsymbol{\theta}} \sum_{i=1}^{m} f_i(\boldsymbol{\theta}) + R(\boldsymbol{\theta}),$$

with $f_i$ is the contribution from the $i$th datapoint, while $R(\boldsymbol{\theta})$ collects all terms that do not depend on the data, including priors in Section 2.1 and constraints in Section 2.2 [1].

Then the trimmed estimator is formulated as

$$\min_{\boldsymbol{\theta}, \boldsymbol{w}} \sum_{i=1}^{m} w_i f_i(\boldsymbol{\theta}) + R(\boldsymbol{\theta}), \quad 0 \le w_i \le 1, \quad \mathbf{1}^T \boldsymbol{w} = h \tag{8}$$

where $h \le m$ is the estimate of inlier datapoints. The set

$$\Delta_h := \left\{ \boldsymbol{w} : 0 \le w_i \le 1, \mathbf{1}^T \boldsymbol{w} = h \right\}$$

is known as the *capped simplex*, since it is the intersection of the simplex with the unit box [2]. The estimator (8) is compactly written as

$$\min_{\boldsymbol{\theta}, \boldsymbol{w} \in \Delta_h} \sum_{i=1}^{m} w_i f_i(\boldsymbol{\theta}) + R(\boldsymbol{\theta}). \tag{9}$$

# 4. Optimization

The SFM model is fit using an algorithm based on variable projection [4, 7, 3], which allows us to leverage a third-party solver, IPOPT [9] to optimize over $\boldsymbol{\theta}$, significantly reducing complexity. Consider

[1] In particular, since $R$ includes constraints, it is infinite-valued off of the feasible region.

the joint likelihood (9) and define the value function $v(\boldsymbol{w})$ and values $(\boldsymbol{\theta}(\boldsymbol{w}), \gamma(\boldsymbol{w}))$ by

$$\begin{aligned} v(\boldsymbol{w}) &= \min_{\boldsymbol{\theta}} \sum_{i=1}^{m} w_i f_i(\boldsymbol{\theta}) + R(\boldsymbol{\theta}) \\ \boldsymbol{\theta}(\boldsymbol{w}) &= \arg\min_{\boldsymbol{\theta}} \sum_{i=1}^{m} w_i f_i(\boldsymbol{\theta}) + R(\boldsymbol{\theta}). \end{aligned} \tag{10}$$

We use IPOPT to solve this problem for each $\boldsymbol{w}$, reducing the problem to

$$\min_{\boldsymbol{w} \in \Delta_h} v(\boldsymbol{w}).$$

where $v(\boldsymbol{w})$ is differentiable with derivative given by

$$\nabla v(\boldsymbol{w}) = \begin{bmatrix} f_1(\boldsymbol{\theta}) \\ \vdots \\ f_m(\boldsymbol{\theta}) \end{bmatrix} \tag{11}$$

The top level algorithm is simply a projected gradient method

$$\boldsymbol{w}^+ = \mathrm{proj}_{\Delta_h}(\boldsymbol{w} - \alpha \nabla v(\boldsymbol{w}))$$

for an appropriately chosen stepsize. Each evaluation of $\nabla v$ requires a full minimization step over the constrained weighted likelihood with respect to $\boldsymbol{\theta}$ using IPOPT, see (11) and (10). The capped simplex $\Delta_h$ is a closed convex set with a simple projection [2]; a simple proximal gradient with line search converges in this case.

# 5. Estimating Random Effects (Inefficiencies).

Once fixed effects $\boldsymbol{\theta}$ have been estimated, we want to obtain estimates of inefficiency from the joint likelihood (3). We optimize

$$\min_{v_i \ge 0} \frac{(y_i - \langle \boldsymbol{x}_i, \boldsymbol{\beta} \rangle + v_i)^2}{2\sigma_i^2} + \frac{v_i^2}{2\eta} \tag{12}$$

We get the closed form solution

$$\hat{v}_i = \max \left( 0, \frac{\frac{1}{\sigma_i^2}(\boldsymbol{x}_i^T \boldsymbol{\beta} - y_i)}{\frac{1}{\sigma_i^2} + \frac{1}{\eta}} \right) = \max \left( 0, \frac{\eta(\boldsymbol{x}_i^T \boldsymbol{\beta} - y_i)}{\sigma_i^2 + \eta} \right). \tag{13}$$

# References


[1] D. Aigner, C. K. Lovell, and P. Schmidt. Formulation and estimation of stochastic frontier production function models. *Journal of econometrics*, 6(1):21–37, 1977.

[2] A. Aravkin and D. Davis. Trimmed statistical estimation via variance reduction. *Mathematics of Optimization Research*, 2019.

[3] A. Y. Aravkin and T. Van Leeuwen. Estimating nuisance parameters in inverse problems. *Inverse Problems*, 28(11):115016, 2012.

[4] B. M. Bell, J. V. Burke, and A. Schumitzky. A relative weighting method for estimating parameters and variances in multiple data sets. *Computational statistics & data analysis*, 22(2):119–135, 1996.

[5] C. De Boor, C. De Boor, E.-U. Mathématicien, C. De Boor, and C. De Boor. *A practical guide to splines*, volume 27. springer-verlag New York, 1978.

[6] J. H. Friedman. Multivariate adaptive regression splines. *The annals of statistics*, pages 1–67, 1991.

[7] G. Golub and V. Pereyra. Separable nonlinear least squares: the variable projection method and its applications. *Inverse problems*, 19(2):R1, 2003.

[8] N. Pya and S. N. Wood. Shape constrained additive models. *Statistics and Computing*, 25(3):543–559, 2015.

[9] A. Wächter and L. T. Biegler. On the implementation of an interior-point filter line-search algorithm for large-scale nonlinear programming. *Mathematical programming*, 106(1):25–57, 2006.

[10] E. Yang, A. C. Lozano, and A. Aravkin. A general family of trimmed estimators for robust high-dimensional data analysis. *Electronic Journal of Statistics*, 12(2):3519–3553, 2018.

# Part 5. Projections for UHC effective coverage

## Section 1. Overview

UHC has emerged as both a global and national health priority, with achieving UHC viewed as a critical path to improved health outcomes and greater equity in health across all populations. This section focuses on the method used for forecasting the UHC effective coverage index from 2020 through 2030 using our health financing variables, particularly the sum of government health expenditure (GHE), lead-distributed development assistance for health (DAH), out-of-pocket (OOP) and prepaid private spending (PPP) per capita, hereby referred as "total health spending per capita."

## Section 2. Forecasting steps

We forecasted the UHC effective coverage index from 2020 through 2030 in the following steps:

1. Forecasts of the total health spending were developed by adding the forecasts of GHE, lead-distributed DAH, OOP, and PPP per capita, modelled previously using ensembles.[1]
2. A meta-stochastic frontier model was fit for 1000 draws, using UHC index as the dependent variable and total health spending per capita estimates as the independent variable, and including all countries and years that health spending data were available.
3. Country- and year-specific inefficiencies were then extracted from the model, and separately forecasted to 2030 in log-transformed space for each country using a non-increasing exponentially weighted ordinary linear regression (using a linear time trend as a covariate), where recent time periods were weighted higher than the further past.
4. Using the draws of reference, better, and worse scenarios of total health spending per capita along with reference scenario forecasts of the efficiency term from (iii), we created reference, better, and worse projections of the UHC effective coverage index from 2020 through 2030.

## Section 3. Meta-stochastic frontier analysis

We used a meta-stochastic frontier model[2] to forecast the level of UHC effective coverage index achievable by all countries between 2020 through 2030. This approach used for forecasting, as summarized below, draws from the methods detailed in Part 4 of the methods appendix.

In brief, this model allows for specification of the variance of the datapoints when fitting the frontier, priors and constraints on all parameters, polynomial splines, and outlier trimming. Our SFA model, with a production function specification, was such:

$$\begin{aligned} UHC_{i,t} &= \beta(X_{i,t}) - \nu_{i,t} + \epsilon_{i,t} \\ \nu_{i,t} &\sim N^{+}(0, \sigma_{\nu}^{2}) \\ \epsilon_{i,t} &\sim N(0, \sigma_{\epsilon}^{2}) \end{aligned}$$

where our observed outcome was the UHC effective cvoreage index, with our single covariate $X$ being the country-year specific total health spending per capita, $\epsilon_{i,t}$ is the noise component and $\nu_{i,t}$ is the estimated technical efficiency that a country would need to achive the optimal, frontier goal. The prior distribution of technical efficiency is a half-normal distribution, describing an unbounded distribution between zero and very high efficiency.

## References

1 Micah AE, Su Y, Bachmeier SD, *et al.* Health sector spending and spending on HIV/AIDS, tuberculosis, and malaria, and development assistance for health: progress towards Sustainable Development Goal 3. *The Lancet* 2020; **0**. DOI:10.1016/S0140-6736(20)30608-5.

2 Aigner D, Lovell CAK, Schmidt P. Formulation and estimation of stochastic frontier production function models. *Journal of Econometrics* 1977; **6**: 21–37.

# Part 6. Online tools and glossary of terms

## Online tools

GBD 2019 data sources and additional results are presented in a series of tools and dynamic visualisations, which will be updated and available at the time of publication at http://ghdx.healthdata.org/gbd-2019.

Analytic source code for estimates will be updated and available at time of publication at http://ghdx.healthdata.org/gbd-2019 under GBD 2019 code for the paper "Measuring universal health coverage based on an index of effective coverage of health services in 204 countries and territories, 1990–2019: a systematic analysis for the Global Burden of Disease Study 2019."

## List of abbreviations

| | |
|---|---|
| ART | antiretroviral therapy |
| ANC1 | antenatal care, at least 1 visit |
| ANC4 | antenatal care, at least 4 visits |
| BCG | Bacillus Calmette–Guérin vaccine |
| BP | blood pressure |
| CKD | chronic kidney disease |
| Comm & MCH | communicable diseases and maternal, child, health |
| COPD | chronic obstructive pulmonary disease |
| DAH | development assistance for health |
| DALY | disability-adjusted life-year |
| DCP3 | Disease Control Priorities, third edition |
| DBP | diastolic blood pressure |
| DTP | diphtheria-tetanus-pertussis |
| DTP3 | diphtheria-tetanus-pertussis vaccine, 3 doses |
| EC | effective coverage |
| ERG | Expert Reference Group |
| FP | family planning |
| FPG | fasting plasma glucose |
| GATHER | Guidelines for Accurate and Transparent Health Estimates Reporting |
| GBD | Global Burden of Diseases, Injuries, and Risk Factors Study |
| GPW13 | General Programme of Work 13 |
| GHDx | Global Health Data Exchange |
| GHE | government health expenditure |
| HALE | healthy life expectancy |
| HAQ Index | Healthcare Access and Quality Index |
| HIV/AIDS | human immunodeficiency virus/acquired immunodeficiency syndrome |
| HPV | human papillomavirus |
| IHD | ischaemic heart disease |
| IHR | International Health Regulations |
| IRS | indoor residual spraying |
| IAEG-SDGs | Inter-agency Expert Group on SDG Indicators |
| ITN | insecticide-treated net |

| | |
|---|---|
| LRI | lower respiratory infection |
| MAP | Malaria Atlas Project |
| MCV | measles-containing vaccine |
| MCV1 | measles-containing-vaccine, 1 dose |
| MCV2 | measles-containing-vaccine, 2 doses |
| MDG | Millennium Development Goal |
| MIRs | mortality-to-incidence ratios |
| MMR | maternal mortality ratio |
| MNCH | maternal, neonatal, and child health |
| MPRs | mortality-to-prevalence ratios |
| NCDs | non-communicable diseases |
| ND | deaths attributable to the new population attributable fraction |
| NMD | non-metabolic deaths |
| NM PAF | non-metabolic joint population attributable fraction |
| NPAF | new population attributable fraction |
| NTD | neglected tropical disease |
| OOP | out-of-pocket |
| PAF | population attributable fraction |
| PAHO | Pan-American Health Organization |
| PCV | pneumococcal conjugate vaccine |
| PCV3 | pneumococcal conjugate vaccine, 3 doses |
| Polio3 | polio vaccine, 3 doses |
| RMNCH | reproductive, maternal, neonatal, and child health |
| PPP | prepaid private spending |
| RSD | risk-standardised deaths |
| RSDR | risk-standardised death rate |
| SBP | systolic blood pressure |
| SC | service coverage |
| SCI | service coverage index |
| SDG | Sustainable Development Goal |
| SDI | Socio-demographic Index |
| SFA | stochastic frontier analysis |
| SFM | stochastic frontier meta-analysis |
| STEPS | WHO STEPwise approach to surveillance |
| TB | tuberculosis |
| UHC | universal health coverage |
| UHC ECI | universal health coverage effective coverage index |
| UHC SCI | universal health coverage service coverage index |
| UI | uncertainty interval |
| UN | United Nations |
| WHO | World Health Organization |

## List of ISO3 codes and location names

| | |
|---|---|
| AFG | Afghanistan |
| AGO | Angola |
| ALB | Albania |
| AND | Andorra |
| ARE | United Arab Emirates |
| ARG | Argentina |
| ARM | Armenia |
| ASM | American Samoa |
| ATG | Antigua and Barbuda |
| AUS | Australia |
| AUT | Austria |
| AZE | Azerbaijan |
| BDI | Burundi |
| BEL | Belgium |
| BEN | Benin |
| BFA | Burkina Faso |
| BGD | Bangladesh |
| BGR | Bulgaria |
| BHR | Bahrain |
| BHS | Bahamas |
| BIH | Bosnia and Herzegovina |
| BLR | Belarus |
| BLZ | Belize |
| BMU | Bermuda |
| BOL | Bolivia (Plurinational State of) |
| BRA | Brazil |
| BRB | Barbados |
| BRN | Brunei Darussalam |
| BTN | Bhutan |
| BWA | Botswana |
| CAF | Central African Republic |
| CAN | Canada |
| CHE | Switzerland |
| CHL | Chile |
| CHN | China |
| CIV | Côte d'Ivoire |
| CMR | Cameroon |
| COD | Democratic Republic of the Congo |
| COG | Congo |
| COK | Cook Islands |
| COL | Colombia |
| COM | Comoros |
| CPV | Cabo Verde |
| CRI | Costa Rica |
| CUB | Cuba |
| CYP | Cyprus |

| | |
|---|---|
| CZE | Czechia |
| DEU | Germany |
| DJI | Djibouti |
| DMA | Dominica |
| DNK | Denmark |
| DOM | Dominican Republic |
| DZA | Algeria |
| ECU | Ecuador |
| EGY | Egypt |
| ERI | Eritrea |
| ESP | Spain |
| EST | Estonia |
| ETH | Ethiopia |
| FIN | Finland |
| FJI | Fiji |
| FRA | France |
| FSM | Micronesia (Federated States of) |
| GAB | Gabon |
| GBR | United Kingdom |
| GEO | Georgia |
| GHA | Ghana |
| GIN | Guinea |
| GMB | Gambia |
| GNB | Guinea-Bissau |
| GNQ | Equatorial Guinea |
| GRC | Greece |
| GRD | Grenada |
| GRL | Greenland |
| GTM | Guatemala |
| GUM | Guam |
| GUY | Guyana |
| HND | Honduras |
| HRV | Croatia |
| HTI | Haiti |
| HUN | Hungary |
| IDN | Indonesia |
| IND | India |
| IRL | Ireland |
| IRN | Iran |
| IRQ | Iraq |
| ISL | Iceland |
| ISR | Israel |
| ITA | Italy |
| JAM | Jamaica |
| JOR | Jordan |
| JPN | Japan |
| KAZ | Kazakhstan |

| | |
|---|---|
| KEN | Kenya |
| KGZ | Kyrgyzstan |
| KHM | Cambodia |
| KIR | Kiribati |
| KNA | Saint Kitts and Nevis |
| KOR | South Korea |
| KWT | Kuwait |
| LAO | Laos |
| LBN | Lebanon |
| LBR | Liberia |
| LBY | Libya |
| LCA | Saint Lucia |
| LKA | Sri Lanka |
| LSO | Lesotho |
| LTU | Lithuania |
| LUX | Luxembourg |
| LVA | Latvia |
| MAR | Morocco |
| MCO | Monaco |
| MDA | Republic of Moldova |
| MDG | Madagascar |
| MDV | Maldives |
| MEX | Mexico |
| MHL | Marshall Islands |
| MKD | North Macedonia |
| MLI | Mali |
| MLT | Malta |
| MMR | Myanmar |
| MNE | Montenegro |
| MNG | Mongolia |
| MNP | Northern Mariana Islands |
| MOZ | Mozambique |
| MRT | Mauritania |
| MUS | Mauritius |
| MWI | Malawi |
| MYS | Malaysia |
| NAM | Namibia |
| NER | Niger |
| NGA | Nigeria |
| NIC | Nicaragua |
| NIU | Niue |
| NLD | Netherlands |
| NOR | Norway |
| NPL | Nepal |
| NRU | Nauru |
| NZL | New Zealand |
| OMN | Oman |

| | |
|---|---|
| PAK | Pakistan |
| PAN | Panama |
| PER | Peru |
| PHL | Philippines |
| PLW | Palau |
| PNG | Papua New Guinea |
| POL | Poland |
| PRI | Puerto Rico |
| PRK | North Korea |
| PRT | Portugal |
| PRY | Paraguay |
| PSE | Palestine |
| QAT | Qatar |
| ROU | Romania |
| RUS | Russia |
| RWA | Rwanda |
| SAU | Saudi Arabia |
| SDN | Sudan |
| SEN | Senegal |
| SGP | Singapore |
| SLB | Solomon Islands |
| SLE | Sierra Leone |
| SLV | El Salvador |
| SMR | San Marino |
| SOM | Somalia |
| SRB | Serbia |
| SSD | South Sudan |
| STP | São Tomé and Príncipe |
| SUR | Suriname |
| SVK | Slovakia |
| SVN | Slovenia |
| SWE | Sweden |
| SWZ | Eswatini |
| SYC | Seychelles |
| SYR | Syria |
| TCD | Chad |
| TGO | Togo |
| THA | Thailand |
| TJK | Tajikistan |
| TKL | Tokelau |
| TKM | Turkmenistan |
| TLS | Timor-Leste |
| TON | Tonga |
| TTO | Trinidad and Tobago |
| TUN | Tunisia |
| TUR | Turkey |
| TUV | Tuvalu |

| | |
|---|---|
| TWN | Taiwan (province of China) |
| TZA | Tanzania |
| UGA | Uganda |
| UKR | Ukraine |
| URY | Uruguay |
| USA | United States of America |
| UZB | Uzbekistan |
| VCT | Saint Vincent and the Grenadines |
| VEN | Venezuela |
| VIR | Virgin Islands |
| VNM | Vietnam |
| VUT | Vanuatu |
| WSM | Samoa |
| YEM | Yemen |
| ZAF | South Africa |
| ZMB | Zambia |
| ZWE | Zimbabwe |

## Part 7. GATHER checklist

***GATHER checklist of information that should be included in reports of global health estimates, with description of compliance and location of information.***[1]

| # | GATHER checklist item | Description of compliance | Reference |
|---|---|---|---|
| **Objectives and funding** | | | |
| 1 | Define the indicators, populations, and time periods for which estimates were made. | Description of indicators, definitions, relevant time periods, and populations in the paper and appendix. | Provided in the main text and methods appendix. |
| 2 | List the funding sources for the work. | Funding sources listed in the paper. | Provided in the main text summary and methods |
| **Data inputs** | | | |
| *For all data inputs from multiple sources that are synthesized as part of the study:* | | | |
| 3 | Describe how the data were identified and how the data were accessed. | This analysis uses estimates produced through the broader GBD 2019 study; data-seeking methodologies are described in GBD 2019 publications.[2–4] References to the non-GBD data sources used to estimate intervention effectiveness are provided in the main text.<br><br>Overarching data inputs used for estimating met need for family planning, ART coverage, and vaccination coverage are found in the GBD 2017 SDG analysis.[5] Updated data inputs and modeling parameters for GBD 2019 will be covered in corresponding papers; further, such information is available upon request. | Methods described in GBD 2019. Topic-specific write-ups for each cause are provided in capstone papers.[2–4] Data sources and the process used to ascribe effectiveness to indicators are provided in the main text and section 3 of the methods appendix. |
| 4 | Specify the inclusion and exclusion criteria. Identify all ad-hoc exclusions. | This analysis uses estimates produced through the broader GBD 2019 study; data-seeking methodologies are described in GBD 2019 publications.[2–4] The indicator selection process is described in both the main text and methods appendix. | Primary data for underlying GBD models are decribed in topic-specific write-ups for each cause in the GBD 2019 capstone papers.[2–4] The process used to select and exclude indicators |

| | | | |
|---|---|---|---|
| | | | is described in the main text and, in more detail, in part 1 of the methods appendix. |
| 5 | Provide information on all included data sources and their main characteristics. For each data source used, report reference information or contact name/institution, population represented, data collection method, year(s) of data collection, sex and age range, diagnostic criteria or measurement method, and sample size, as relevant. | An interactive, online data source tool for GBD 2019 that provides metadata for data sources by location and over time. | Online data tools can be accessed here: http://ghdx.healthdata.org/gbd-2019 |
| 6 | Identify and describe any categories of input data that have potentially important biases (eg, based on characteristics listed in item 5). | This analysis uses estimates produced through the broader GBD 2019 study; data-seeking methodologies are described in GBD 2019 publications.[2–4] | Methodology described in GBD 2019. Topic-specific write-ups, which may include discussions of bias and bias-correction, for each cause are provided in capstone papers.[2–4] |
| *For data inputs that contribute to the analysis but were not synthesized as part of the study:* | | | |
| 7 | Describe and give sources for any other data inputs. | This analysis uses estimates produced through the broader GBD 2019 study; data-seeking methodologies are described in GBD 2019 publications.[2–4] | Primary data for underlying GBD models are decribed in topic-specific write-ups for each cause in the GBD 2019 capstone papers.[2–4] |
| *For all data inputs:* | | | |
| 8 | Provide all data inputs in a file format from which data can be efficiently extracted (eg, a spreadsheet as opposed to a PDF), including all relevant meta-data listed in item 5. For any data inputs that cannot be shared due to ethical or legal reasons, such as third-party ownership, provide a contact name or the name of the institution that retains the right to the data. | Downloads of input data are available through online tools, including data visualisation tools and data query tools. | Online data tools can be accessed here: http://ghdx.healthdata.org/gbd-2019 |
| **Data analysis** | | | |

| | | | |
|---|---|---|---|
| 9 | Provide a conceptual overview of the data analysis method. A diagram may be helpful. | Overview of analysis provided in the manuscript and appendix | Described in the methods section of the main text and part 2 of the methods appendix. |
| 10 | Provide a detailed description of all steps of the analysis, including mathematical formulae. This description should cover, as relevant, data cleaning, data pre-processing, data adjustments and weighting of data sources, and mathematical or statistical model(s). | UHC methodology is provided in the methodological write-ups. Methodology for underlying GBD models are described in the GBD 2019 capstone publications.[2–4]<br><br>Overarching modeling approaches used for estimating met need for family planning, ART coverage, and vaccination coverage are found in the GBD 2017 SDG analysis.[5] Updated data inputs and modeling parameters for GBD 2019 will be covered in corresponding papers; further, such information is available upon request | UHC methodology described in the methods section of the main text and part 2 of the methods appendix. GBD methodology described in topic specific write-ups.[2–4] |
| 11 | Describe how candidate models were evaluated and how the final model(s) were selected. | UHC methodology is provided in the methodological write-ups. Methodology for underlying GBD models are described in the GBD 2019 capstone publications.[2–4] | UHC methodology described in the methods section of the main text and part 2, 4, and 5 of the methods appendix. GBD methodology described in topic-specific write-ups.[2–4] |
| 12 | Provide the results of an evaluation of model performance, if done, as well as the results of any relevant sensitivity analysis. | We evaluated the UHC effective coverage index using three separate validation criteria and provided results for multiple sensitivity analyses in the main text and appendix. | Validation criteria results are provided in main text table 2 and part 3 of the methods appendix. The results of sensitivity analyses are mentioned briefly in the main text and covered in detail in part 2, section 5 of the methods appendix. |
| 13 | Describe methods for calculating uncertainty of the estimates. State which sources of uncertainty were, and were not, accounted for in the uncertainty analysis. | Inputs for this analysis draw from uncertainty estimates produced in GBD 2019 study.[2–4] | Detail on where uncertainty was and was not propagated is included in section 6, part 2 and parts 4 and 5 |

| | | | |
|---|---|---|---|
| | | | of the methods appendix. |
| 14 | State how analytic or statistical source code used to generate estimates can be accessed. | Access statement provided. | Links to code can be found here: http://ghdx.healthdata.org/gbd-2019. |
| **Results and discussion** | | | |
| 15 | Provide published estimates in a file format from which data can be efficiently extracted. | GBD 2019 results are available through online data visualisation tools, the GHDx, and the online data query tool. | Online data tools are findable at this link: http://ghdx.healthdata.org/gbd-2019 |
| 16 | Report a quantitative measure of the uncertainty of the estimates (eg, uncertainty intervals). | Uncertainty intervals are provided with results. | Provided in the main text results and supplementary results. |
| 17 | Interpret results in light of existing evidence. If updating a previous set of estimates, describe the reasons for changes in estimates. | Discussion provided in narrative of the main paper. | Provided in the summary, research in context, and throughout the main text. |
| 18 | Discuss limitations of the estimates. Include a discussion of any modelling assumptions or data limitations that affect interpretation of the estimates. | Discussion of limitations provided in narrative of the main paper. | Provided in limitations section of the main text and in the methods appendix. |

## References


1 Stevens GA, Alkema L, Black RE, *et al.* Guidelines for Accurate and Transparent Health Estimates Reporting: the GATHER statement. *The Lancet* 2016; **388**: e19–23.

2 GBD 2019 Demographics Collaborators. Global age-sex-specific fertility, mortality, healthy life expectancy (HALE), and population estimates in 204 countries and territories, 1950–2019: a comprehensive demographic analysis for the Global Burden of Disease Study 2019. *The Lancet* In press.

3 GBD 2019 Diseases and Injuries Collaborators. Global burden of 369 diseases and injuries in 204 countries and territories, 1990–2019: a systematic analysis for the Global Burden of Disease Study 2019. *The Lancet* In press.

4 GBD 2019 Risk Factors Collaborators. Global burden of 87 risk factors in 204 countries and territories, 1990–2019; a systematic analysis for the Global Burden of Disease Study 2019. *The Lancet* In press.

5 Lozano R, Fullman N, Abate D, *et al.* Measuring progress from 1990 to 2017 and projecting attainment to 2030 of the health-related Sustainable Development Goals for 195 countries and territories: a systematic analysis for the Global Burden of Disease Study 2017. *The Lancet* 2018; **392**: 2091–138.

6 Zheng P, Aravkin AY, Barber R, Sorensen RJD, Murray CJL. Trimmed Constrained Mixed Effects Models: Formulations and Algorithms. 2019; published online Sept 24. https://arxiv.org/abs/1909.10700v1
