# Supplementary material for: Measuring universal health coverage based on an index of effective coverage of health services in 204 countries and territories, 1990–2019: a systematic analysis for the Global Burden of Disease Study 2019
Source: Lancet. 2020 Oct 17;396(10258):1250–84. doi: 10.1016/S0140-6736(20)30750-9 (PMC7562819; doi:10.1016/S0140-6736(20)30750-9)
Supplement: Supplementary appendix 2 [file mmc2.pdf]

# Results appendix to Measuring universal health coverage based on an index of effective coverage of health services in 204 countries and territories, 1990–2019: a systematic analysis for the Global Burden of Disease Study 2019

This appendix provides supplemental figures and tables.

## List of supplementary figures and tables

**Supplementary figure 1. Map of the UHC effective coverage index, by decile, in 1990.** Deciles are based on the distribution of UHC effective coverage index values in 2019. Where lower and upper bounds of deciles appear to overlap, they should be interpreted as values up to but not equalling the upper bound in the preceding decile (ie, exclusive of the upper bound value) and values equalling the lower bound of the following decile (ie, inclusive of the lower bound value). UHC=universal health coverage.

**Supplementary figure 2. Map of the UHC effective coverage index, by decile, in 2010.** Deciles are based on the distribution of UHC effective coverage index values in 2019. Where lower and upper bounds of deciles appear to overlap, they should be interpreted as values up to but not equalling the upper bound in the preceding decile (ie, exclusive of the upper bound value) and values equalling the lower bound of the following decile (ie, inclusive of the lower bound value). UHC=universal health coverage.

**Supplementary figure 3. UHC effective coverage index frontier relative to total health spending per capita (A) and log-transformed total health spending per capita (B).** Total health spending per capita includes government health expenditures, prepaid private expenditures, out-of-pocket expenditures, and development assistance for health. All health spending estimates are for 2017 measured in 2019 PPP dollars adjusted for inflation. The black line represents the frontier values estimated for UHC effective coverage in 2019 relative to spending per capita in 2017. Locations are colour-coded by GBD super-region, with a subset abbreviated according to their ISO3 codes. UHC=universal health coverage. GBD=Global Burden of Disease. PPP=purchasing-power parity.

**Supplementary figure 4. UHC effective coverage index frontier relative to domestic pooled health spending per capita (A) and log-transformed domestic pooled health spending per capita (B).** Domestic pooled health spending per capita includes government health expenditures and prepaid private expenditures. All health spending estimates are for 2017 measured in 2019 PPP dollars adjusted for inflation. The black line represents the frontier values estimated for UHC effective coverage in 2019 relative to spending per capita in 2017. Locations are colour-coded by GBD super-region, with a subset abbreviated according to their ISO3 codes. UHC=universal health coverage. GBD=Global Burden of Disease. PPP=purchasing-power parity.

**Supplementary figure 5. Comparing the UHC effective coverage index in 2019 with GBD 2017 UHC service coverage index in 2017.** Locations are colour-coded by GBD super-region, and are abbreviated according to their ISO3 codes. UHC=universal health coverage. GBD=Global Burden of Disease.

**Supplementary figure 6. Comparing the UHC effective coverage index in 2019 with UHC SCI in 2017.** Locations are colour-coded by GBD super-region, and are abbreviated according to their ISO3 codes. UHC SCI is used to measure SDG indicator 3.8.1 for UHC service coverage, with WHO as its custodial agency. UHC=universal health coverage. SCI=service coverage index. GBD=Global Burden of Disease. SDG=Sustainable Development Goal.

**Supplementary figure 7. Comparing the UHC effective coverage index in 2019 with the World Bank service coverage index.** Locations are colour-coded by GBD super-region, and are abbreviated according to their ISO3 codes. The World Bank's service coverage index is reported for the most recent year for each country. UHC=universal health coverage. GBD=Global Burden of Disease.

**Supplementary table 1. Health gain weights, by effective coverage indicator, for each location in 2019**. Estimation of health gain weights are further detailed within the methods appendix. FP=family planning. MCV1=measles-containing vaccine, 1 dose. DTP3=diphtheria, tetanus, pertussis vaccine, 3 doses. LRI=lower respiratory infection. ART=antiretroviral therapy. TB=tuberculosis. IHD=ischaemic heart disease. CKD=chronic kidney disease. COPD=chronic obstructive pulmonary disease.

**Supplementary table 2. UHC effective coverage index values for 1990, 2010, and 2019, and annualised rates of change for 1990-2019, 1990-2010, and 2010-2019.** All estimates are accompanied by 95% uncertainty intervals in parentheses. Annualised rate of change is reported in terms of percentages. UHC=universal health coverage.

**Supplementary figure 1. Map of the UHC effective coverage index, by decile, in 1990.** Deciles are based on the distribution of UHC effective coverage index values in 2019. Where lower and upper bounds of deciles appear to overlap, they should be interpreted as values up to but not equalling the upper bound in the preceding decile (ie, exclusive of the upper bound value) and values equalling the lower bound of the following decile (ie, inclusive of the lower bound value). UHC=universal health coverage.

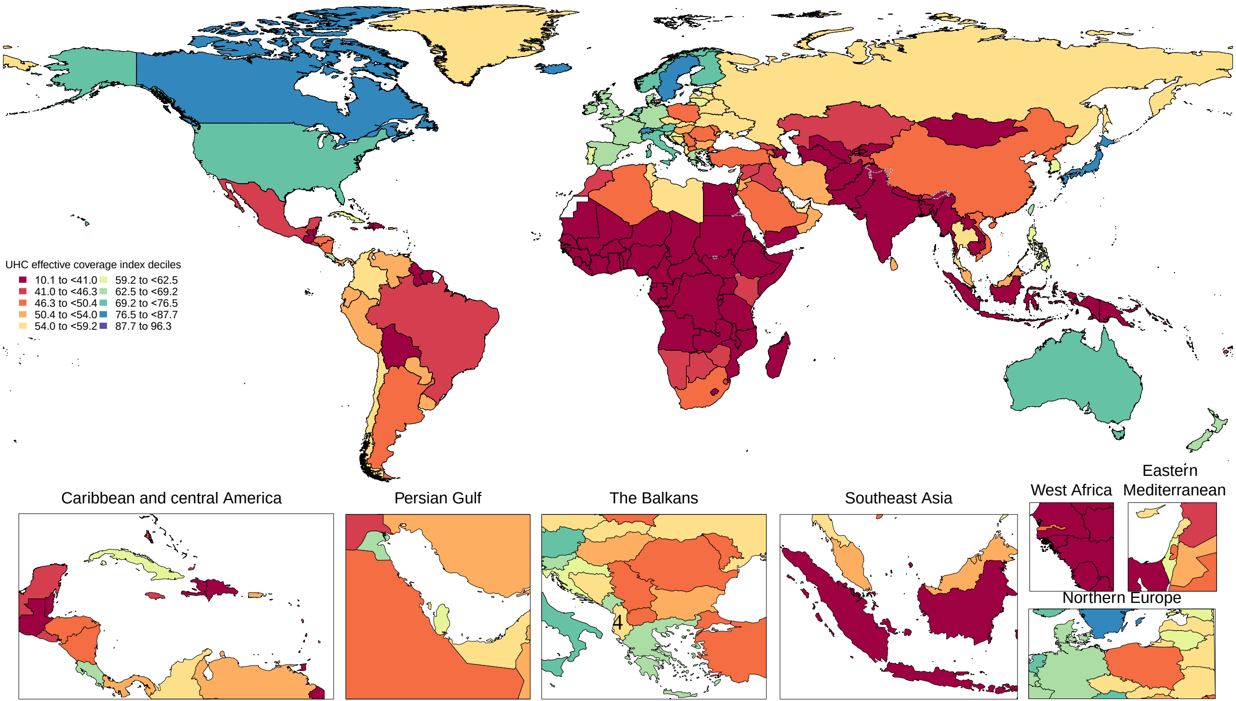

**Supplementary figure 2. Map of the UHC effective coverage index, by decile, in 2010.** Deciles are based on the distribution of UHC effective coverage index values in 2019. Where lower and upper bounds of deciles appear to overlap, they should be interpreted as values up to but not equalling the upper bound in the preceding decile (ie, exclusive of the upper bound value) and values equalling the lower bound of the following decile (ie, inclusive of the lower bound value). UHC=universal health coverage.

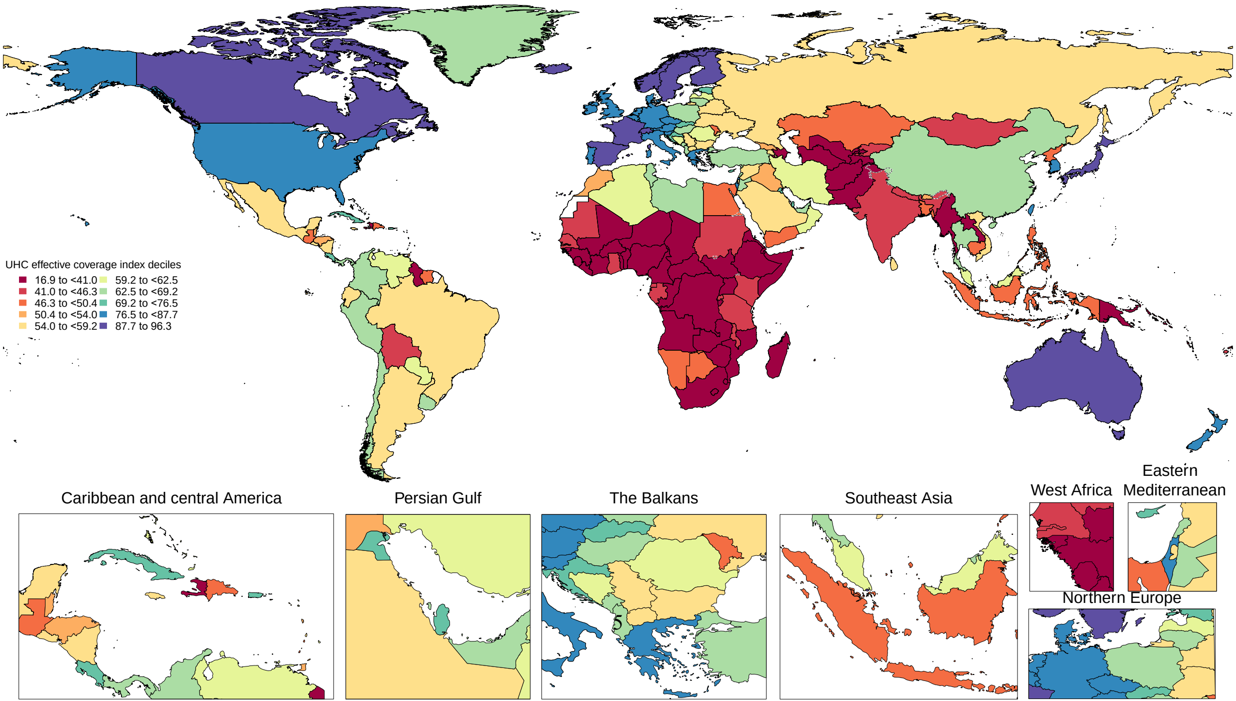

**Supplementary figure 3. UHC effective coverage index frontier relative to total health spending per capita (A) and log-transformed total health spending per capita (B).** Total health spending per capita includes government health expenditures, prepaid private expenditures, out-of-pocket expenditures, and development assistance for health. All health spending estimates are for 2017 measured in 2019 PPP dollars adjusted for inflation. The black line represents the frontier values estimated for UHC effective coverage in 2019 relative to spending per capita in 2017. Locations are colour-coded by GBD super-region, with a subset abbreviated according to their ISO3 codes. UHC=universal health coverage. GBD=Global Burden of Disease. PPP=purchasing-power parity.

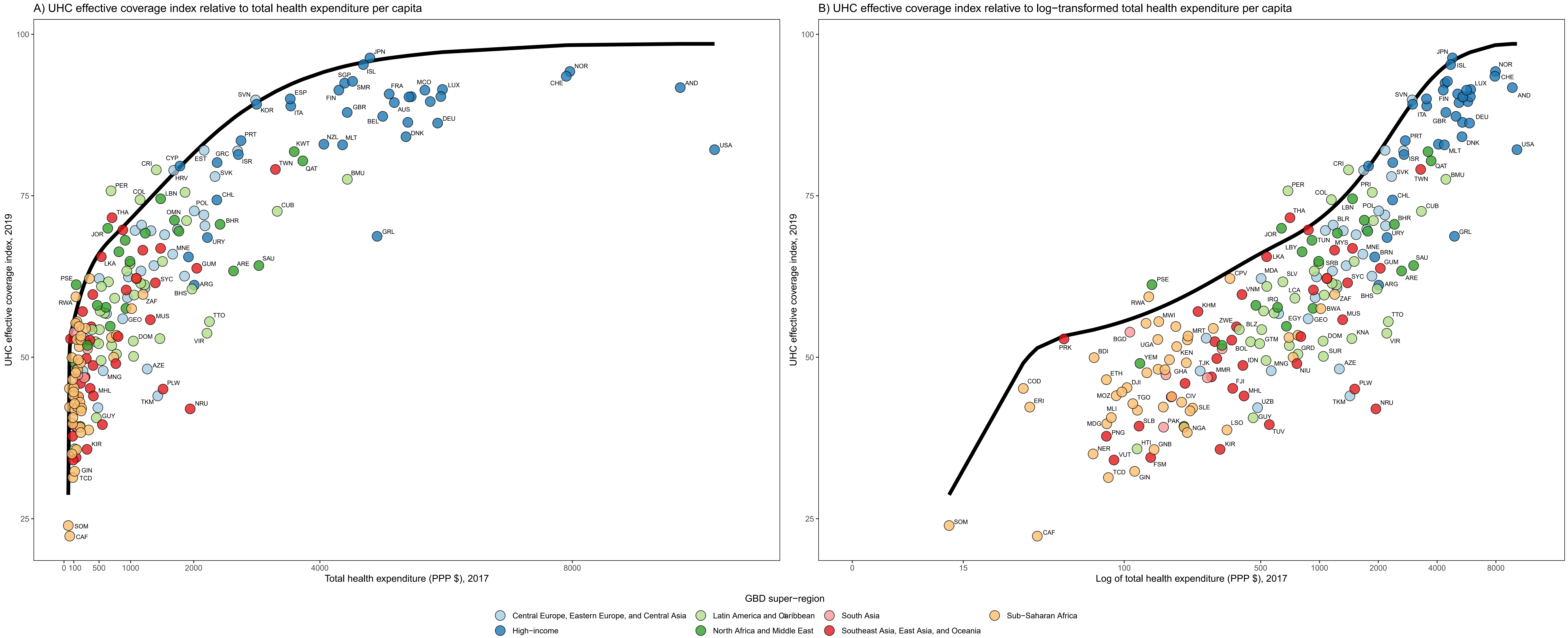

**Supplementary figure 4. UHC effective coverage index frontier relative to domestic pooled health spending per capita (A) and log-transformed domestic pooled health spending per capita (B).** Domestic pooled health spending per capita includes government health expenditures and prepaid private expenditures. All health spending estimates are for 2017 measured in 2019 PPP dollars adjusted for inflation. The black line represents the frontier values estimated for UHC effective coverage in 2019 relative to spending per capita in 2017. Locations are colour-coded by GBD super-region, with a subset abbreviated according to their ISO3 codes. UHC=universal health coverage. GBD=Global Burden of Disease. PPP=purchasing-power parity.

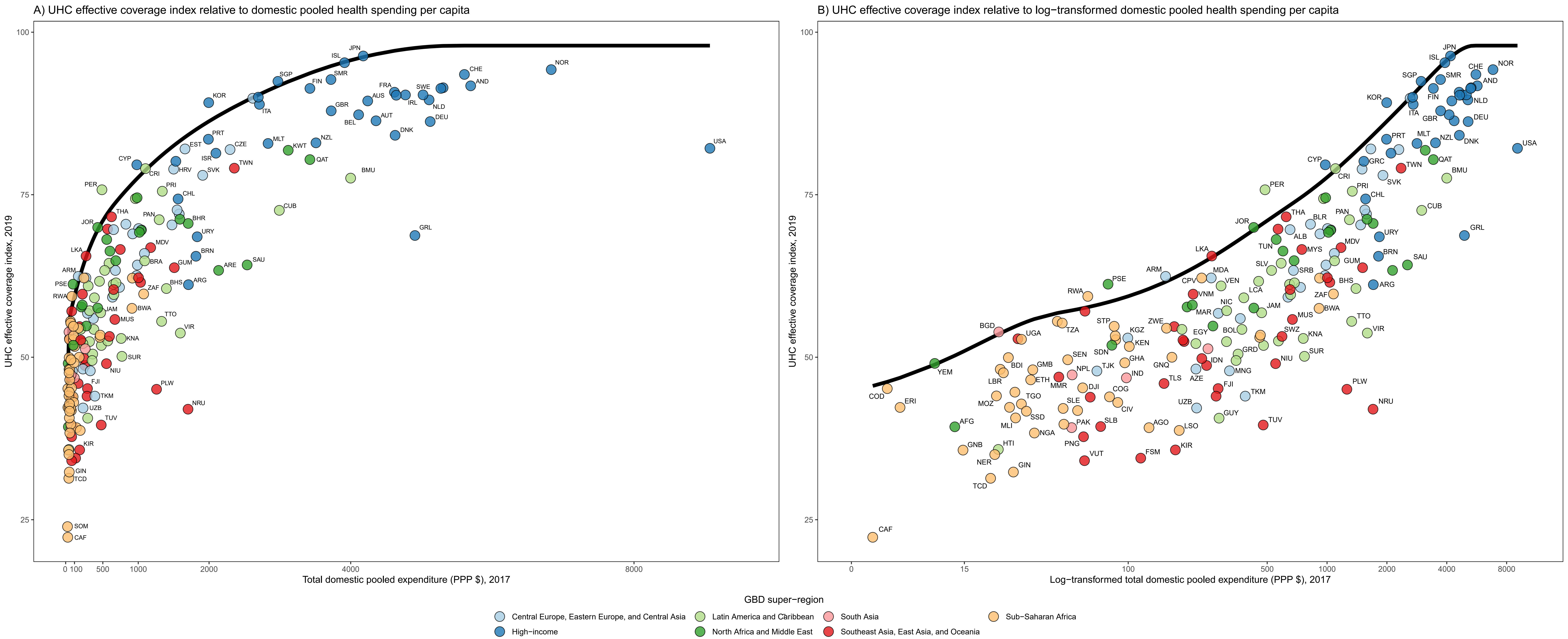

**Supplementary figure 5. Comparing the UHC effective coverage index in 2019 with GBD 2017 UHC service coverage index in 2017.** Locations are colour-coded by GBD super-region, and are abbreviated according to their ISO3 codes. UHC=universal health coverage. GBD=Global Burden of Disease.

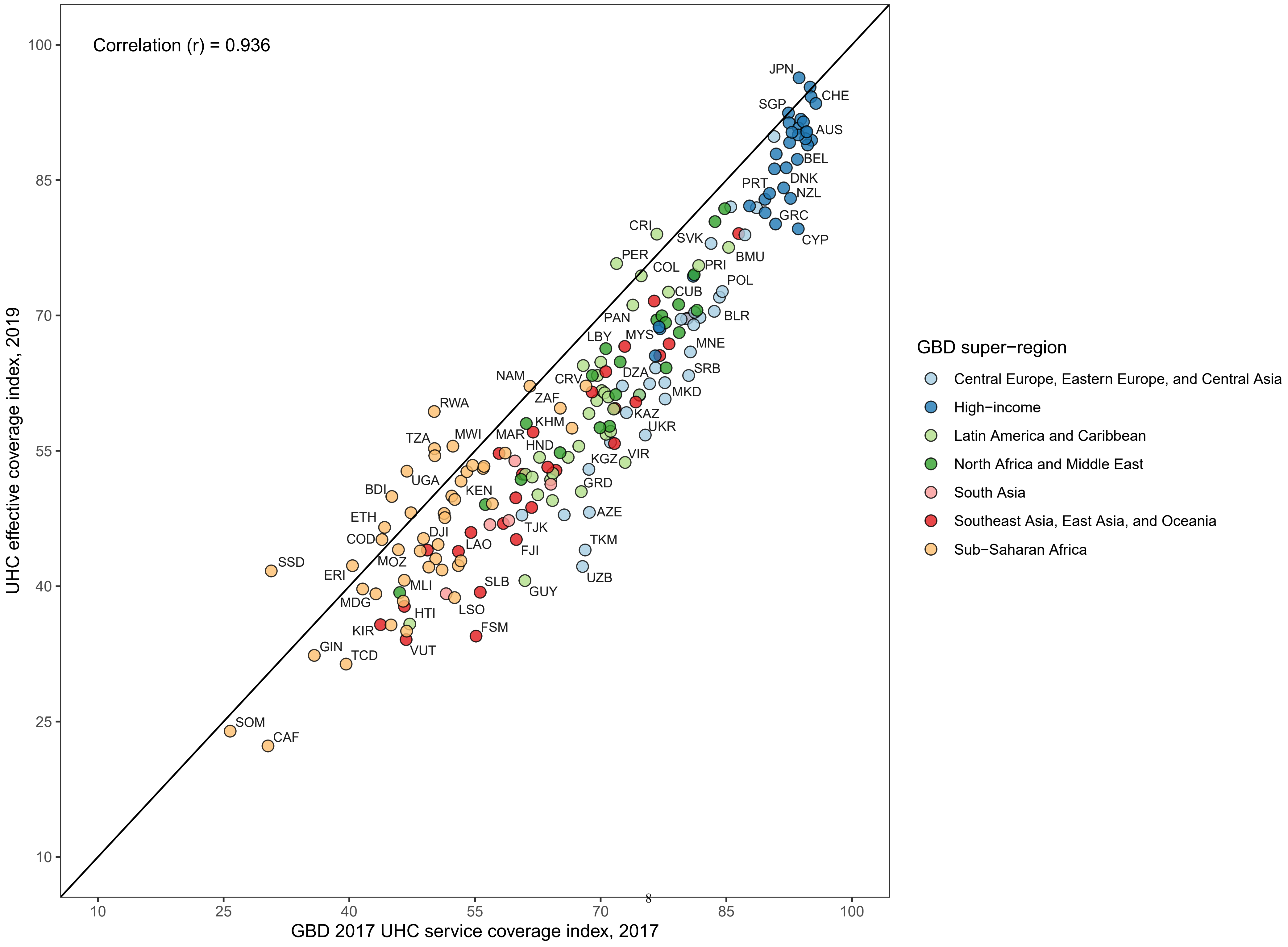

**Supplementary figure 6. Comparing the UHC effective coverage index in 2019 with UHC SCI in 2017.** Locations are colour-coded by GBD super-region, and are abbreviated according to their ISO3 codes. UHC SCI is used to measure SDG indicator 3.8.1 for UHC service coverage, with WHO as its custodial agency. UHC=universal health coverage. SCI=service coverage index. GBD=Global Burden of Disease. SDG=Sustainable Development Goal.

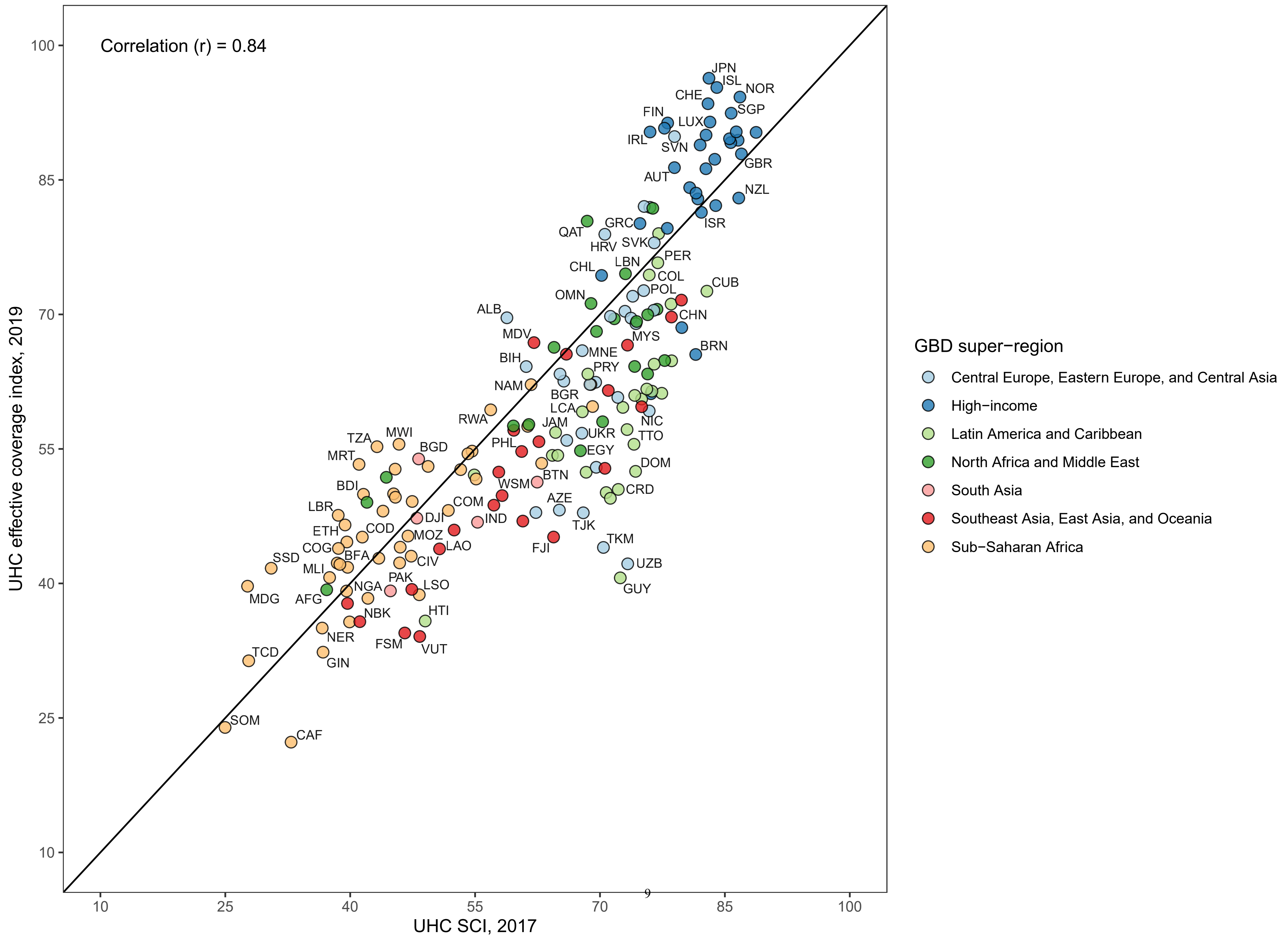

**Supplementary figure 7. Comparing the UHC effective coverage index in 2019 with the World Bank service coverage index.** Locations are colour-coded by GBD super-region, and are abbreviated according to their ISO3 codes. The World Bank's service coverage index is reported for the most recent year for each country. UHC=universal health coverage. GBD=Global Burden of Disease.

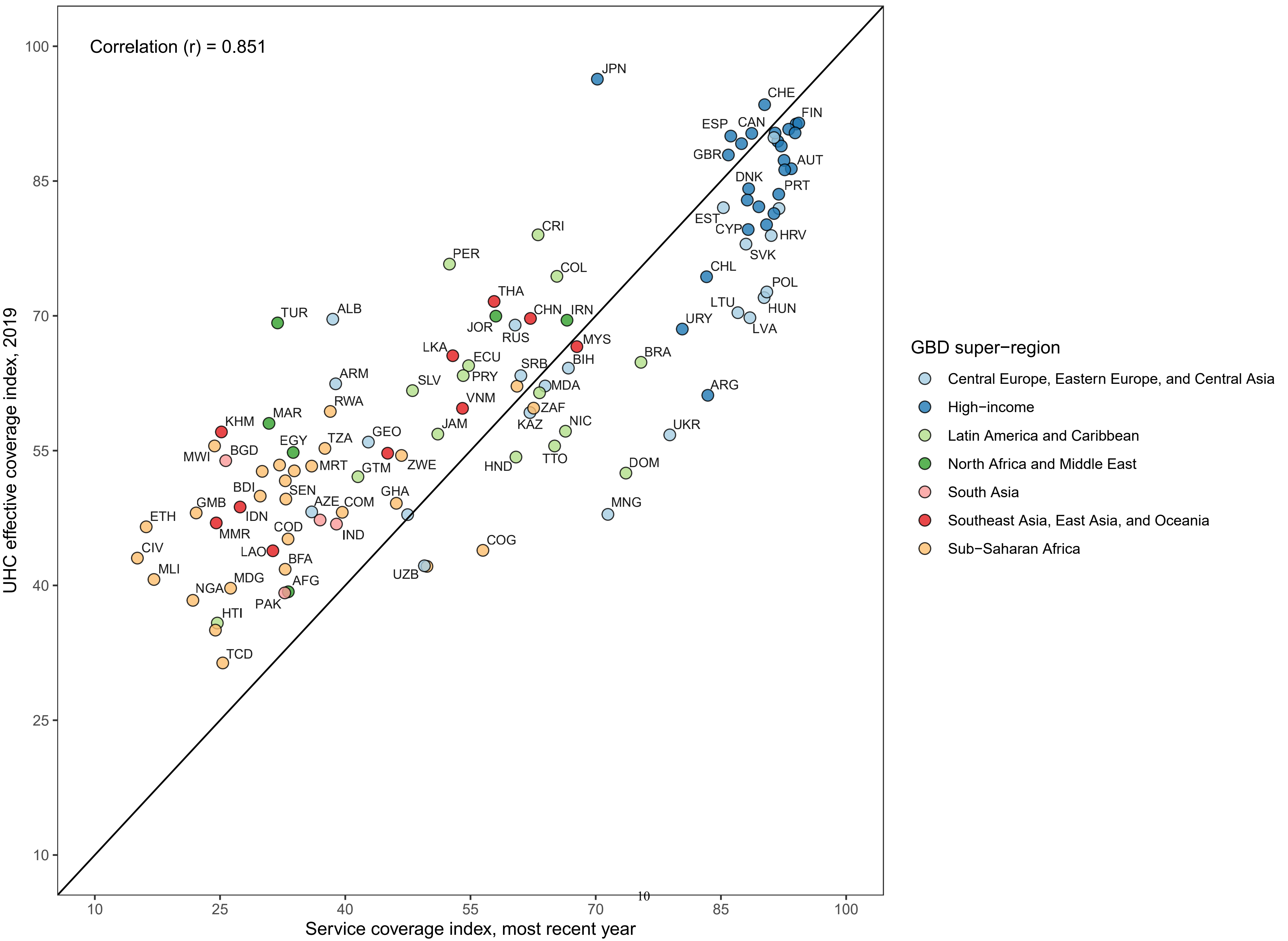

**Supplementary table 1. Health gain weights, by effective coverage indicator, for each location in 2019.** Estimation of health gain weights are further detailed within the methods appendix. FP=family planning. MCV1=measles-containing vaccine, 1 dose. DTP3=diphtheria, tetanus, pertussis vaccine, 3 doses. LRI=lower respiratory infection. ART=antiretroviral therapy. TB=tuberculosis. IHD=ischaemic heart disease. CKD=chronic kidney disease. COPD=chronic obstructive pulmonary disease.

| | Met need for FP with modern contraception | Antenatal, peripartum and postnatal care for newborns | Antenatal, postpartum and postnatal care for mothers | MCV1 coverage | DTP3 coverage | Diarrhoea treatment | LRI treatment | ART coverage | TB treatment | Acute lymphoid leukaemia treatment | Breast cancer treatment | Cervical cancer treatment | Uterine cancer treatment | Colon/rectum cancer treatment | IHD treatment | Stroke treatment | Diabetes treatment | CKD treatment | COPD treatment | Asthma treatment | Epilepsy treatment | Appendicitis treatment |
|---|---|---|---|---|---|---|---|---|---|---|---|---|---|---|---|---|---|---|---|---|---|---|
| Afghanistan | 0.000 | 0.068 | 0.068 | 0.068 | 0.068 | 0.068 | 0.068 | 0.004 | 0.071 | 0.003 | 0.003 | 0.003 | 0.001 | 0.003 | 0.137 | 0.137 | 0.071 | 0.071 | 0.071 | 0.003 | 0.006 | 0.006 |
| Albania | 0.001 | 0.063 | 0.001 | 0.001 | 0.007 | 0.063 | 0.063 | 0.003 | 0.008 | 0.008 | 0.070 | 0.064 | 0.014 | 0.127 | 0.127 | 0.127 | 0.070 | 0.127 | 0.014 | 0.007 | 0.015 | 0.003 |
| Algeria | 0.001 | 0.063 | 0.008 | 0.008 | 0.063 | 0.063 | 0.063 | 0.009 | 0.064 | 0.002 | 0.071 | 0.008 | 0.002 | 0.071 | 0.126 | 0.126 | 0.071 | 0.126 | 0.015 | 0.008 | 0.016 | 0.009 |
| American Samoa | 0.001 | 0.060 | 0.010 | 0.060 | 0.010 | 0.060 | 0.060 | 0.012 | 0.011 | 0.002 | 0.070 | 0.011 | 0.020 | 0.070 | 0.121 | 0.121 | 0.121 | 0.121 | 0.020 | 0.010 | 0.012 | 0.003 |
| Andorra | 0.001 | 0.007 | 0.001 | 0.001 | 0.001 | 0.007 | 0.007 | 0.009 | 0.008 | 0.008 | 0.127 | 0.071 | 0.071 | 0.127 | 0.127 | 0.127 | 0.071 | 0.127 | 0.071 | 0.007 | 0.009 | 0.002 |
| Angola | 0.001 | 0.067 | 0.067 | 0.067 | 0.067 | 0.067 | 0.067 | 0.134 | 0.071 | 0.001 | 0.005 | 0.005 | 0.001 | 0.005 | 0.071 | 0.133 | 0.071 | 0.071 | 0.005 | 0.004 | 0.009 | 0.005 |
| Antigua and Barbuda | 0.001 | 0.012 | 0.001 | 0.001 | 0.001 | 0.058 | 0.058 | 0.060 | 0.014 | 0.002 | 0.070 | 0.070 | 0.025 | 0.070 | 0.115 | 0.115 | 0.115 | 0.115 | 0.025 | 0.012 | 0.015 | 0.015 |
| Argentina | 0.001 | 0.055 | 0.015 | 0.001 | 0.001 | 0.015 | 0.055 | 0.058 | 0.016 | 0.003 | 0.070 | 0.070 | 0.016 | 0.111 | 0.111 | 0.111 | 0.070 | 0.111 | 0.030 | 0.015 | 0.018 | 0.018 |
| Armenia | 0.001 | 0.060 | 0.001 | 0.001 | 0.001 | 0.060 | 0.060 | 0.013 | 0.060 | 0.002 | 0.071 | 0.071 | 0.022 | 0.071 | 0.119 | 0.119 | 0.119 | 0.071 | 0.022 | 0.011 | 0.013 | 0.013 |
| Australia | 0.001 | 0.008 | 0.001 | 0.001 | 0.001 | 0.008 | 0.008 | 0.010 | 0.009 | 0.002 | 0.125 | 0.071 | 0.016 | 0.125 | 0.125 | 0.125 | 0.071 | 0.125 | 0.071 | 0.063 | 0.010 | 0.010 |
| Austria | 0.001 | 0.008 | 0.001 | 0.001 | 0.001 | 0.008 | 0.008 | 0.010 | 0.009 | 0.009 | 0.124 | 0.017 | 0.017 | 0.124 | 0.124 | 0.124 | 0.124 | 0.124 | 0.124 | 0.008 | 0.010 | 0.002 |
| Azerbaijan | 0.001 | 0.065 | 0.001 | 0.001 | 0.065 | 0.065 | 0.065 | 0.008 | 0.071 | 0.001 | 0.071 | 0.007 | 0.007 | 0.071 | 0.129 | 0.129 | 0.071 | 0.071 | 0.071 | 0.006 | 0.013 | 0.008 |
| Bahrain | 0.001 | 0.008 | 0.008 | 0.001 | 0.008 | 0.062 | 0.062 | 0.010 | 0.063 | 0.002 | 0.070 | 0.009 | 0.009 | 0.070 | 0.125 | 0.125 | 0.125 | 0.125 | 0.070 | 0.008 | 0.017 | 0.010 |
| Bangladesh | 0.001 | 0.061 | 0.009 | 0.009 | 0.061 | 0.061 | 0.061 | 0.003 | 0.070 | 0.002 | 0.070 | 0.010 | 0.002 | 0.018 | 0.123 | 0.123 | 0.070 | 0.070 | 0.070 | 0.001 | 0.011 | 0.019 |
| Barbados | 0.001 | 0.011 | 0.001 | 0.001 | 0.001 | 0.011 | 0.011 | 0.061 | 0.012 | 0.002 | 0.118 | 0.070 | 0.023 | 0.118 | 0.118 | 0.118 | 0.118 | 0.118 | 0.023 | 0.011 | 0.013 | 0.013 |
| Belarus | 0.001 | 0.007 | 0.001 | 0.001 | 0.001 | 0.007 | 0.007 | 0.065 | 0.064 | 0.001 | 0.127 | 0.071 | 0.014 | 0.127 | 0.127 | 0.127 | 0.071 | 0.071 | 0.014 | 0.064 | 0.008 | 0.008 |
| Belgium | 0.001 | 0.009 | 0.001 | 0.001 | 0.001 | 0.009 | 0.009 | 0.011 | 0.010 | 0.010 | 0.124 | 0.018 | 0.018 | 0.124 | 0.124 | 0.124 | 0.124 | 0.124 | 0.124 | 0.009 | 0.011 | 0.003 |
| Belize | 0.001 | 0.061 | 0.009 | 0.001 | 0.001 | 0.061 | 0.061 | 0.071 | 0.062 | 0.003 | 0.062 | 0.070 | 0.011 | 0.018 | 0.122 | 0.122 | 0.070 | 0.122 | 0.018 | 0.009 | 0.020 | 0.012 |
| Benin | 0.003 | 0.068 | 0.068 | 0.068 | 0.068 | 0.068 | 0.068 | 0.136 | 0.071 | 0.001 | 0.004 | 0.004 | 0.001 | 0.004 | 0.135 | 0.135 | 0.006 | 0.071 | 0.006 | 0.003 | 0.007 | 0.002 |
| Bermuda | 0.001 | 0.008 | 0.001 | 0.001 | 0.001 | 0.008 | 0.008 | 0.064 | 0.009 | 0.002 | 0.125 | 0.071 | 0.016 | 0.125 | 0.125 | 0.125 | 0.125 | 0.125 | 0.016 | 0.008 | 0.010 | 0.010 |
| Bhutan | 0.001 | 0.060 | 0.010 | 0.001 | 0.010 | 0.060 | 0.060 | 0.063 | 0.070 | 0.003 | 0.012 | 0.012 | 0.003 | 0.021 | 0.119 | 0.119 | 0.070 | 0.070 | 0.119 | 0.001 | 0.013 | 0.022 |
| Bolivia | 0.002 | 0.061 | 0.008 | 0.002 | 0.061 | 0.061 | 0.061 | 0.065 | 0.070 | 0.004 | 0.010 | 0.070 | 0.004 | 0.017 | 0.122 | 0.122 | 0.070 | 0.122 | 0.017 | 0.008 | 0.012 | 0.019 |
| Bosnia and Herzegovina | 0.001 | 0.008 | 0.001 | 0.001 | 0.008 | 0.008 | 0.008 | 0.002 | 0.016 | 0.001 | 0.125 | 0.071 | 0.016 | 0.125 | 0.125 | 0.125 | 0.125 | 0.125 | 0.016 | 0.062 | 0.010 | 0.002 |
| Botswana | 0.000 | 0.069 | 0.002 | 0.002 | 0.069 | 0.069 | 0.069 | 0.207 | 0.071 | 0.001 | 0.002 | 0.069 | 0.001 | 0.004 | 0.071 | 0.138 | 0.071 | 0.071 | 0.004 | 0.002 | 0.003 | 0.001 |
| Brazil | 0.001 | 0.059 | 0.011 | 0.001 | 0.001 | 0.059 | 0.059 | 0.061 | 0.070 | 0.003 | 0.070 | 0.060 | 0.013 | 0.070 | 0.117 | 0.117 | 0.070 | 0.070 | 0.023 | 0.011 | 0.014 | 0.014 |
| Brunei | 0.001 | 0.061 | 0.001 | 0.001 | 0.010 | 0.010 | 0.061 | 0.012 | 0.070 | 0.002 | 0.070 | 0.070 | 0.011 | 0.121 | 0.121 | 0.121 | 0.121 | 0.070 | 0.019 | 0.010 | 0.012 | 0.012 |
| Bulgaria | 0.001 | 0.008 | 0.001 | 0.001 | 0.001 | 0.008 | 0.063 | 0.009 | 0.008 | 0.001 | 0.126 | 0.071 | 0.071 | 0.126 | 0.126 | 0.126 | 0.071 | 0.126 | 0.016 | 0.008 | 0.009 | 0.009 |
| Burkina Faso | 0.003 | 0.068 | 0.068 | 0.068 | 0.068 | 0.068 | 0.068 | 0.137 | 0.071 | 0.001 | 0.003 | 0.069 | 0.001 | 0.003 | 0.136 | 0.071 | 0.006 | 0.071 | 0.003 | 0.003 | 0.006 | 0.001 |
| Burundi | 0.003 | 0.068 | 0.068 | 0.068 | 0.068 | 0.068 | 0.068 | 0.136 | 0.135 | 0.001 | 0.004 | 0.004 | 0.001 | 0.004 | 0.071 | 0.135 | 0.006 | 0.071 | 0.006 | 0.003 | 0.007 | 0.002 |
| Cambodia | 0.001 | 0.064 | 0.007 | 0.064 | 0.064 | 0.064 | 0.064 | 0.066 | 0.127 | 0.002 | 0.008 | 0.008 | 0.008 | 0.013 | 0.127 | 0.127 | 0.070 | 0.070 | 0.013 | 0.007 | 0.009 | 0.009 |
| Cameroon | 0.001 | 0.067 | 0.067 | 0.067 | 0.067 | 0.067 | 0.067 | 0.138 | 0.071 | 0.001 | 0.004 | 0.004 | 0.001 | 0.004 | 0.134 | 0.134 | 0.007 | 0.071 | 0.007 | 0.004 | 0.008 | 0.002 |
| Canada | 0.001 | 0.009 | 0.001 | 0.001 | 0.001 | 0.009 | 0.009 | 0.010 | 0.009 | 0.009 | 0.125 | 0.071 | 0.071 | 0.125 | 0.125 | 0.125 | 0.071 | 0.125 | 0.071 | 0.009 | 0.010 | 0.002 |
| Cape Verde | 0.002 | 0.060 | 0.010 | 0.060 | 0.010 | 0.060 | 0.060 | 0.071 | 0.070 | 0.004 | 0.062 | 0.012 | 0.004 | 0.020 | 0.119 | 0.119 | 0.070 | 0.070 | 0.020 | 0.010 | 0.022 | 0.005 |
| Central African Republic | 0.000 | 0.068 | 0.068 | 0.068 | 0.068 | 0.068 | 0.068 | 0.139 | 0.136 | 0.001 | 0.003 | 0.003 | 0.001 | 0.003 | 0.071 | 0.071 | 0.071 | 0.071 | 0.006 | 0.003 | 0.006 | 0.001 |
| Chad | 0.002 | 0.069 | 0.069 | 0.069 | 0.069 | 0.069 | 0.069 | 0.138 | 0.071 | 0.001 | 0.002 | 0.002 | 0.001 | 0.002 | 0.138 | 0.138 | 0.004 | 0.071 | 0.004 | 0.002 | 0.004 | 0.001 |
| Chile | 0.002 | 0.056 | 0.002 | 0.002 | 0.002 | 0.014 | 0.056 | 0.060 | 0.027 | 0.003 | 0.070 | 0.070 | 0.015 | 0.112 | 0.112 | 0.112 | 0.070 | 0.112 | 0.027 | 0.014 | 0.017 | 0.017 |
| China | 0.001 | 0.008 | 0.001 | 0.008 | 0.008 | 0.008 | 0.062 | 0.010 | 0.070 | 0.009 | 0.070 | 0.009 | 0.009 | 0.124 | 0.124 | 0.124 | 0.070 | 0.124 | 0.124 | 0.008 | 0.010 | 0.003 |
| Colombia | 0.002 | 0.057 | 0.012 | 0.002 | 0.002 | 0.057 | 0.057 | 0.061 | 0.014 | 0.014 | 0.069 | 0.059 | 0.014 | 0.069 | 0.114 | 0.114 | 0.069 | 0.114 | 0.024 | 0.012 | 0.016 | 0.016 |
| Comoros | 0.001 | 0.065 | 0.005 | 0.065 | 0.065 | 0.065 | 0.065 | 0.003 | 0.131 | 0.002 | 0.010 | 0.066 | 0.002 | 0.010 | 0.131 | 0.131 | 0.071 | 0.071 | 0.010 | 0.005 | 0.011 | 0.003 |
| Congo (Brazzaville) | 0.001 | 0.065 | 0.006 | 0.065 | 0.065 | 0.065 | 0.065 | 0.135 | 0.071 | 0.002 | 0.007 | 0.007 | 0.002 | 0.007 | 0.129 | 0.129 | 0.071 | 0.071 | 0.012 | 0.006 | 0.013 | 0.002 |
| Cook Islands | 0.001 | 0.009 | 0.001 | 0.009 | 0.009 | 0.061 | 0.061 | 0.063 | 0.019 | 0.002 | 0.070 | 0.010 | 0.010 | 0.070 | 0.122 | 0.122 | 0.122 | 0.122 | 0.070 | 0.009 | 0.011 | 0.003 |
| Costa Rica | 0.002 | 0.057 | 0.002 | 0.002 | 0.002 | 0.057 | 0.057 | 0.060 | 0.014 | 0.014 | 0.070 | 0.059 | 0.014 | 0.070 | 0.114 | 0.114 | 0.070 | 0.114 | 0.025 | 0.013 | 0.027 | 0.016 |
| Croatia | 0.001 | 0.008 | 0.001 | 0.001 | 0.001 | 0.008 | 0.008 | 0.009 | 0.016 | 0.001 | 0.126 | 0.071 | 0.071 | 0.126 | 0.126 | 0.126 | 0.126 | 0.126 | 0.016 | 0.008 | 0.009 | 0.002 |
| Cuba | 0.001 | 0.012 | 0.001 | 0.001 | 0.001 | 0.012 | 0.012 | 0.060 | 0.013 | 0.002 | 0.071 | 0.071 | 0.071 | 0.117 | 0.117 | 0.117 | 0.117 | 0.117 | 0.024 | 0.012 | 0.014 | 0.014 |
| Cyprus | 0.001 | 0.008 | 0.001 | 0.001 | 0.001 | 0.008 | 0.008 | 0.002 | 0.008 | 0.008 | 0.126 | 0.015 | 0.015 | 0.126 | 0.126 | 0.126 | 0.126 | 0.126 | 0.071 | 0.063 | 0.009 | 0.009 |
| Czech Republic | 0.001 | 0.007 | 0.007 | 0.001 | 0.001 | 0.007 | 0.007 | 0.009 | 0.008 | 0.001 | 0.127 | 0.071 | 0.014 | 0.127 | 0.127 | 0.127 | 0.127 | 0.127 | 0.071 | 0.007 | 0.009 | 0.002 |
| Côte d'Ivoire | 0.001 | 0.067 | 0.067 | 0.004 | 0.067 | 0.067 | 0.067 | 0.138 | 0.071 | 0.001 | 0.004 | 0.004 | 0.001 | 0.004 | 0.135 | 0.135 | 0.071 | 0.071 | 0.007 | 0.004 | 0.008 | 0.002 |
| DR Congo | 0.001 | 0.066 | 0.066 | 0.066 | 0.066 | 0.066 | 0.066 | 0.071 | 0.132 | 0.001 | 0.005 | 0.005 | 0.001 | 0.005 | 0.071 | 0.132 | 0.071 | 0.071 | 0.009 | 0.005 | 0.010 | 0.006 |
| Denmark | 0.001 | 0.009 | 0.001 | 0.001 | 0.001 | 0.009 | 0.009 | 0.010 | 0.010 | 0.010 | 0.124 | 0.063 | 0.017 | 0.124 | 0.124 | 0.124 | 0.071 | 0.124 | 0.124 | 0.009 | 0.010 | 0.010 |
| Djibouti | 0.005 | 0.065 | 0.065 | 0.065 | 0.065 | 0.065 | 0.065 | 0.136 | 0.071 | 0.002 | 0.006 | 0.006 | 0.002 | 0.006 | 0.131 | 0.131 | 0.010 | 0.071 | 0.006 | 0.005 | 0.011 | 0.002 |
| Dominica | 0.002 | 0.057 | 0.002 | 0.002 | 0.013 | 0.057 | 0.057 | 0.061 | 0.015 | 0.004 | 0.070 | 0.070 | 0.015 | 0.025 | 0.114 | 0.114 | 0.114 | 0.114 | 0.025 | 0.013 | 0.027 | 0.006 |
| Dominican Republic | 0.001 | 0.063 | 0.007 | 0.001 | 0.063 | 0.063 | 0.063 | 0.065 | 0.070 | 0.003 | 0.070 | 0.064 | 0.009 | 0.015 | 0.125 | 0.125 | 0.070 | 0.070 | 0.015 | 0.007 | 0.010 | 0.010 |
| Ecuador | 0.002 | 0.056 | 0.013 | 0.002 | 0.056 | 0.056 | 0.056 | 0.061 | 0.059 | 0.005 | 0.059 | 0.015 | 0.015 | 0.025 | 0.113 | 0.113 | 0.069 | 0.113 | 0.025 | 0.013 | 0.028 | 0.028 |
| Egypt | 0.001 | 0.067 | 0.004 | 0.004 | 0.067 | 0.067 | 0.067 | 0.002 | 0.005 | 0.001 | 0.071 | 0.001 | 0.005 | 0.071 | 0.133 | 0.133 | 0.071 | 0.133 | 0.071 | 0.004 | 0.009 | 0.005 |
| El Salvador | 0.002 | 0.059 | 0.002 | 0.002 | 0.011 | 0.059 | 0.059 | 0.063 | 0.012 | 0.004 | 0.070 | 0.070 | 0.012 | 0.021 | 0.118 | 0.118 | 0.070 | 0.118 | 0.021 | 0.059 | 0.023 | 0.014 |
| Equatorial Guinea | 0.001 | 0.066 | 0.005 | 0.005 | 0.066 | 0.066 | 0.066 | 0.136 | 0.071 | 0.002 | 0.066 | 0.006 | 0.002 | 0.006 | 0.131 | 0.131 | 0.071 | 0.071 | 0.006 | 0.005 | 0.011 | 0.002 |
| Eritrea | 0.004 | 0.066 | 0.066 | 0.066 | 0.066 | 0.066 | 0.066 | 0.071 | 0.133 | 0.001 | 0.005 | 0.067 | 0.001 | 0.005 | 0.071 | 0.071 | 0.071 | 0.071 | 0.009 | 0.004 | 0.009 | 0.002 |
| Estonia | 0.001 | 0.008 | 0.008 | 0.001 | 0.001 | 0.008 | 0.008 | 0.064 | 0.009 | 0.002 | 0.125 | 0.071 | 0.071 | 0.125 | 0.125 | 0.125 | 0.071 | 0.125 | 0.016 | 0.008 | 0.010 | 0.002 |
| eSwatini | 0.000 | 0.070 | 0.002 | 0.002 | 0.070 | 0.070 | 0.070 | 0.209 | 0.071 | 0.000 | 0.002 | 0.002 | 0.000 | 0.002 | 0.139 | 0.139 | 0.071 | 0.071 | 0.003 | 0.002 | 0.003 | 0.001 |
| Ethiopia | 0.001 | 0.067 | 0.067 | 0.067 | 0.067 | 0.067 | 0.067 | 0.135 | 0.071 | 0.004 | 0.004 | 0.004 | 0.001 | 0.004 | 0.134 | 0.134 | 0.007 | 0.071 | 0.007 | 0.004 | 0.008 | 0.002 |
| Federated States of Micronesia | 0.001 | 0.062 | 0.008 | 0.001 | 0.008 | 0.062 | 0.062 | 0.078 | 0.070 | 0.002 | 0.070 | 0.064 | 0.009 | 0.009 | 0.125 | 0.125 | 0.070 | 0.070 | 0.070 | 0.008 | 0.010 | 0.003 |
| Fiji | 0.001 | 0.064 | 0.006 | 0.001 | 0.064 | 0.064 | 0.064 | 0.008 | 0.070 | 0.002 | 0.070 | 0.070 | 0.007 | 0.012 | 0.129 | 0.129 | 0.129 | 0.070 | 0.012 | 0.006 | 0.008 | 0.003 |
| Finland | 0.001 | 0.007 | 0.001 | 0.001 | 0.001 | 0.007 | 0.007 | 0.002 | 0.008 | 0.008 | 0.128 | 0.014 | 0.014 | 0.128 | 0.128 | 0.128 | 0.128 | 0.128 | 0.071 | 0.064 | 0.008 | 0.008 |
| France | 0.001 | 0.010 | 0.001 | 0.001 | 0.001 | 0.010 | 0.010 | 0.012 | 0.011 | 0.011 | 0.122 | 0.070 | 0.070 | 0.122 | 0.122 | 0.122 | 0.019 | 0.122 | 0.070 | 0.061 | 0.012 | 0.003 |
| Gabon | 0.001 | 0.064 | 0.006 | 0.006 | 0.064 | 0.064 | 0.064 | 0.135 | 0.070 | 0.002 | 0.065 | 0.007 | 0.002 | 0.012 | 0.129 | 0.129 | 0.070 | 0.070 | 0.007 | 0.006 | 0.013 | 0.003 |
| Georgia | 0.001 | 0.061 | 0.001 | 0.001 | 0.001 | 0.009 | 0.061 | 0.011 | 0.071 | 0.001 | 0.071 | 0.071 | 0.019 | 0.071 | 0.122 | 0.122 | 0.122 | 0.122 | 0.019 | 0.009 | 0.011 | 0.002 |
| Germany | 0.001 | 0.008 | 0.001 | 0.001 | 0.001 | 0.008 | 0.008 | 0.010 | 0.009 | 0.009 | 0.125 | 0.071 | 0.016 | 0.125 | 0.125 | 0.125 | 0.125 | 0.125 | 0.071 | 0.008 | 0.010 | 0.003 |
| Ghana | 0.001 | 0.065 | 0.006 | 0.065 | 0.065 | 0.065 | 0.065 | 0.130 | 0.071 | 0.002 | 0.071 | 0.007 | 0.002 | 0.007 | 0.129 | 0.129 | 0.012 | 0.071 | 0.012 | 0.006 | 0.013 | 0.003 |
| Greece | 0.001 | 0.008 | 0.001 | 0.001 | 0.001 | 0.008 | 0.008 | 0.009 | 0.008 | 0.008 | 0.126 | 0.016 | 0.016 | 0.126 | 0.126 | 0.126 | 0.126 | 0.126 | 0.071 | 0.063 | 0.009 | 0.002 |
| Greenland | 0.001 | 0.057 | 0.001 | 0.013 | 0.013 | 0.013 | 0.013 | 0.059 | 0.070 | 0.002 | 0.070 | 0.058 | 0.002 | 0.114 | 0.114 | 0.114 | 0.070 | 0.070 | 0.070 | 0.013 | 0.015 | 0.015 |
| Grenada | 0.001 | 0.058 | 0.001 | 0.001 | 0.001 | 0.012 | 0.058 | 0.060 | 0.013 | 0.002 | 0.070 | 0.070 | 0.024 | 0.070 | 0.116 | 0.116 | 0.116 | 0.116 | 0.024 | 0.012 | 0.014 | 0.014 |
| Guam | 0.001 | 0.061 | 0.009 | 0.061 | 0.009 | 0.061 | 0.061 | 0.011 | 0.071 | 0.002 | 0.071 | 0.010 | 0.010 | 0.071 | 0.122 | 0.122 | 0.071 | 0.122 | 0.019 | 0.009 | 0.011 | 0.003 |
| Guatemala | 0.001 | 0.064 | 0.006 | 0.001 | 0.006 | 0.064 | 0.064 | 0.066 | 0.065 | 0.003 | 0.008 | 0.065 | 0.003 | 0.013 | 0.127 | 0.127 | 0.127 | 0.127 | 0.013 | 0.006 | 0.014 | 0.014 |
| Guinea | 0.004 | 0.067 | 0.067 | 0.067 | 0.067 | 0.067 | 0.067 | 0.071 | 0.071 | 0.001 | 0.005 | 0.067 | 0.001 | 0.005 | 0.134 | 0.134 | 0.008 | 0.071 | 0.008 | 0.004 | 0.009 | 0.002 |
| Guinea-Bissau | 0.000 | 0.068 | 0.068 | 0.068 | 0.068 | 0.068 | 0.068 | 0.139 | 0.071 | 0.001 | 0.004 | 0.068 | 0.001 | 0.004 | 0.135 | 0.071 | 0.007 | 0.071 | 0.007 | 0.003 | 0.007 | 0.001 |
| Guyana | 0.001 | 0.063 | 0.007 | 0.001 | 0.001 | 0.063 | 0.063 | 0.077 | 0.070 | 0.003 | 0.070 | 0.064 | 0.009 | 0.015 | 0.125 | 0.125 | 0.125 | 0.070 | 0.009 | 0.007 | 0.010 | 0.010 |
| Haiti | 0.001 | 0.065 | 0.005 | 0.001 | 0.065 | 0.065 | 0.065 | 0.136 | 0.070 | 0.006 | 0.006 | 0.066 | 0.002 | 0.006 | 0.131 | 0.131 | 0.070 | 0.070 | 0.010 | 0.005 | 0.011 | 0.007 |
| Honduras | 0.002 | 0.061 | 0.009 | 0.002 | 0.061 | 0.061 | 0.061 | 0.064 | 0.070 | 0.003 | 0.063 | 0.010 | 0.010 | 0.010 | 0.123 | 0.123 | 0.070 | 0.123 | 0.017 | 0.009 | 0.019 | 0.019 |
| Hungary | 0.001 | 0.007 | 0.001 | 0.001 | 0.001 | 0.007 | 0.007 | 0.008 | 0.008 | 0.001 | 0.127 | 0.014 | 0.014 | 0.127 | 0.127 | 0.127 | 0.127 | 0.127 | 0.127 | 0.007 | 0.008 | 0.008 |
| Iceland | 0.001 | 0.009 | 0.001 | 0.001 | 0.001 | 0.009 | 0.009 | 0.011 | 0.010 | 0.010 | 0.123 | 0.063 | 0.018 | 0.123 | 0.123 | 0.123 | 0.071 | 0.123 | 0.071 | 0.062 | 0.011 | 0.011 |
| India | 0.001 | 0.062 | 0.008 | 0.008 | 0.062 | 0.062 | 0.062 | 0.064 | 0.070 | 0.002 | 0.017 | 0.009 | 0.002 | 0.017 | 0.124 | 0.124 | 0.070 | 0.070 | 0.124 | 0.001 | 0.010 | 0.010 |
| Indonesia | 0.001 | 0.063 | 0.008 | 0.008 | 0.063 | 0.063 | 0.063 | 0.009 | 0.126 | 0.002 | 0.071 | 0.009 | 0.009 | 0.071 | 0.126 | 0.126 | 0.071 | 0.071 | 0.015 | 0.008 | 0.009 | 0.003 |
| Iran | 0.001 | 0.062 | 0.001 | 0.008 | 0.008 | 0.062 | 0.062 | 0.010 | 0.064 | 0.002 | 0.070 | 0.009 | 0.009 | 0.070 | 0.125 | 0.125 | 0.070 | 0.125 | 0.070 | 0.008 | 0.017 | 0.010 |
| Iraq | 0.001 | 0.065 | 0.006 | 0.006 | 0.065 | 0.065 | 0.065 | 0.002 | 0.071 | 0.002 | 0.071 | 0.006 | 0.006 | 0.071 | 0.130 | 0.130 | 0.071 | 0.130 | 0.006 | 0.006 | 0.012 | 0.007 |
| Ireland | 0.001 | 0.008 | 0.001 | 0.001 | 0.001 | 0.008 | 0.008 | 0.010 | 0.009 | 0.009 | 0.124 | 0.071 | 0.071 | 0.124 | 0.124 | 0.124 | 0.071 | 0.124 | 0.071 | 0.008 | 0.010 | 0.002 |
| Israel | 0.001 | 0.059 | 0.012 | 0.001 | 0.001 | 0.059 | 0.012 | 0.014 | 0.013 | 0.013 | 0.117 | 0.013 | 0.023 | 0.117 | 0.117 | 0.117 | 0.117 | 0.117 | 0.023 | 0.012 | 0.014 | 0.004 |
| Italy | 0.001 | 0.008 | 0.001 | 0.001 | 0.001 | 0.008 | 0.008 | 0.009 | 0.008 | 0.008 | 0.126 | 0.071 | 0.016 | 0.126 | 0.126 | 0.126 | 0.126 | 0.126 | 0.071 | 0.008 | 0.009 | 0.002 |

| | Met need for FP with modern contraception | Antenatal, peripartum and postnatal care for newborns | Antenatal, postpartum and postnatal care for mothers | MCV1 coverage | DTP3 coverage | Diarrhoea treatment | LRI treatment | ART coverage | TB treatment | Acute lymphoid leukaemia treatment | Breast cancer treatment | Cervical cancer treatment | Uterine cancer treatment | Colon/rectum cancer treatment | IHD treatment | Stroke treatment | Diabetes treatment | CKD treatment | COPD treatment | Asthma treatment | Epilepsy treatment | Appendicitis treatment |
|---|---|---|---|---|---|---|---|---|---|---|---|---|---|---|---|---|---|---|---|---|---|---|
| Jamaica | 0.001 | 0.060 | 0.001 | 0.001 | 0.001 | 0.010 | 0.060 | 0.071 | 0.011 | 0.003 | 0.070 | 0.070 | 0.019 | 0.070 | 0.121 | 0.121 | 0.121 | 0.121 | 0.019 | 0.010 | 0.013 | 0.013 |
| Japan | 0.001 | 0.006 | 0.001 | 0.001 | 0.001 | 0.001 | 0.006 | 0.002 | 0.012 | 0.007 | 0.130 | 0.071 | 0.071 | 0.130 | 0.130 | 0.130 | 0.071 | 0.130 | 0.071 | 0.006 | 0.007 | 0.007 |
| Jordan | 0.001 | 0.064 | 0.007 | 0.007 | 0.064 | 0.064 | 0.064 | 0.002 | 0.008 | 0.002 | 0.071 | 0.008 | 0.008 | 0.071 | 0.127 | 0.127 | 0.071 | 0.127 | 0.014 | 0.064 | 0.015 | 0.009 |
| Kazakhstan | 0.001 | 0.061 | 0.009 | 0.001 | 0.001 | 0.061 | 0.061 | 0.011 | 0.062 | 0.002 | 0.071 | 0.062 | 0.018 | 0.071 | 0.123 | 0.123 | 0.071 | 0.071 | 0.071 | 0.009 | 0.019 | 0.011 |
| Kenya | 0.000 | 0.067 | 0.067 | 0.004 | 0.067 | 0.067 | 0.067 | 0.138 | 0.135 | 0.001 | 0.004 | 0.004 | 0.001 | 0.004 | 0.135 | 0.135 | 0.007 | 0.071 | 0.007 | 0.004 | 0.008 | 0.001 |
| Kiribati | 0.001 | 0.065 | 0.006 | 0.065 | 0.065 | 0.065 | 0.065 | 0.007 | 0.071 | 0.001 | 0.071 | 0.071 | 0.006 | 0.006 | 0.130 | 0.130 | 0.071 | 0.071 | 0.011 | 0.006 | 0.012 | 0.002 |
| Kuwait | 0.001 | 0.064 | 0.007 | 0.001 | 0.001 | 0.064 | 0.064 | 0.008 | 0.065 | 0.007 | 0.071 | 0.007 | 0.007 | 0.071 | 0.128 | 0.128 | 0.071 | 0.128 | 0.013 | 0.064 | 0.014 | 0.008 |
| Kyrgyzstan | 0.001 | 0.064 | 0.006 | 0.001 | 0.001 | 0.064 | 0.064 | 0.066 | 0.071 | 0.002 | 0.071 | 0.065 | 0.007 | 0.013 | 0.128 | 0.128 | 0.071 | 0.071 | 0.071 | 0.006 | 0.014 | 0.008 |
| Laos | 0.001 | 0.064 | 0.007 | 0.064 | 0.064 | 0.064 | 0.064 | 0.066 | 0.070 | 0.002 | 0.065 | 0.008 | 0.008 | 0.013 | 0.127 | 0.127 | 0.070 | 0.070 | 0.013 | 0.007 | 0.009 | 0.009 |
| Latvia | 0.001 | 0.008 | 0.001 | 0.001 | 0.001 | 0.008 | 0.008 | 0.064 | 0.071 | 0.001 | 0.126 | 0.016 | 0.071 | 0.126 | 0.126 | 0.126 | 0.071 | 0.126 | 0.016 | 0.008 | 0.009 | 0.002 |
| Lebanon | 0.001 | 0.061 | 0.001 | 0.009 | 0.009 | 0.061 | 0.061 | 0.012 | 0.011 | 0.011 | 0.122 | 0.011 | 0.011 | 0.122 | 0.122 | 0.122 | 0.070 | 0.122 | 0.019 | 0.009 | 0.012 | 0.012 |
| Lesotho | 0.000 | 0.069 | 0.003 | 0.003 | 0.069 | 0.069 | 0.069 | 0.206 | 0.137 | 0.001 | 0.005 | 0.005 | 0.001 | 0.003 | 0.071 | 0.137 | 0.071 | 0.071 | 0.005 | 0.000 | 0.003 | 0.001 |
| Liberia | 0.004 | 0.066 | 0.066 | 0.066 | 0.066 | 0.066 | 0.066 | 0.071 | 0.071 | 0.001 | 0.005 | 0.005 | 0.001 | 0.005 | 0.133 | 0.133 | 0.071 | 0.071 | 0.005 | 0.004 | 0.010 | 0.002 |
| Libya | 0.001 | 0.062 | 0.009 | 0.001 | 0.009 | 0.062 | 0.062 | 0.010 | 0.063 | 0.002 | 0.071 | 0.010 | 0.010 | 0.071 | 0.124 | 0.124 | 0.071 | 0.124 | 0.071 | 0.009 | 0.018 | 0.010 |
| Lithuania | 0.001 | 0.008 | 0.001 | 0.001 | 0.001 | 0.008 | 0.008 | 0.064 | 0.071 | 0.001 | 0.125 | 0.071 | 0.017 | 0.125 | 0.125 | 0.125 | 0.071 | 0.125 | 0.017 | 0.008 | 0.010 | 0.002 |
| Luxembourg | 0.001 | 0.009 | 0.001 | 0.001 | 0.001 | 0.009 | 0.009 | 0.011 | 0.010 | 0.010 | 0.123 | 0.010 | 0.071 | 0.123 | 0.123 | 0.123 | 0.123 | 0.123 | 0.071 | 0.009 | 0.011 | 0.011 |
| Madagascar | 0.001 | 0.067 | 0.067 | 0.067 | 0.067 | 0.067 | 0.067 | 0.071 | 0.071 | 0.001 | 0.004 | 0.068 | 0.001 | 0.004 | 0.134 | 0.134 | 0.008 | 0.071 | 0.008 | 0.004 | 0.008 | 0.002 |
| Malawi | 0.000 | 0.068 | 0.003 | 0.068 | 0.068 | 0.068 | 0.068 | 0.139 | 0.137 | 0.001 | 0.003 | 0.069 | 0.001 | 0.001 | 0.071 | 0.137 | 0.005 | 0.071 | 0.005 | 0.003 | 0.006 | 0.001 |
| Malaysia | 0.002 | 0.059 | 0.002 | 0.011 | 0.011 | 0.059 | 0.059 | 0.062 | 0.070 | 0.004 | 0.070 | 0.013 | 0.013 | 0.070 | 0.118 | 0.118 | 0.070 | 0.118 | 0.022 | 0.011 | 0.014 | 0.005 |
| Maldives | 0.001 | 0.061 | 0.010 | 0.010 | 0.010 | 0.061 | 0.061 | 0.003 | 0.070 | 0.002 | 0.070 | 0.011 | 0.011 | 0.070 | 0.121 | 0.121 | 0.070 | 0.121 | 0.070 | 0.010 | 0.020 | 0.003 |
| Mali | 0.003 | 0.068 | 0.068 | 0.068 | 0.068 | 0.068 | 0.068 | 0.137 | 0.071 | 0.001 | 0.003 | 0.003 | 0.001 | 0.003 | 0.136 | 0.136 | 0.006 | 0.071 | 0.006 | 0.003 | 0.006 | 0.001 |
| Malta | 0.001 | 0.063 | 0.001 | 0.001 | 0.001 | 0.008 | 0.008 | 0.009 | 0.009 | 0.009 | 0.126 | 0.016 | 0.016 | 0.126 | 0.126 | 0.126 | 0.126 | 0.126 | 0.071 | 0.008 | 0.009 | 0.002 |
| Marshall Islands | 0.001 | 0.063 | 0.008 | 0.063 | 0.063 | 0.063 | 0.063 | 0.064 | 0.071 | 0.002 | 0.071 | 0.009 | 0.009 | 0.016 | 0.125 | 0.125 | 0.071 | 0.071 | 0.016 | 0.008 | 0.010 | 0.002 |
| Mauritania | 0.005 | 0.065 | 0.065 | 0.065 | 0.065 | 0.065 | 0.065 | 0.007 | 0.071 | 0.002 | 0.006 | 0.066 | 0.002 | 0.006 | 0.131 | 0.131 | 0.010 | 0.071 | 0.010 | 0.065 | 0.011 | 0.003 |
| Mauritius | 0.001 | 0.062 | 0.001 | 0.001 | 0.001 | 0.062 | 0.062 | 0.064 | 0.009 | 0.002 | 0.071 | 0.017 | 0.017 | 0.071 | 0.125 | 0.125 | 0.125 | 0.125 | 0.017 | 0.008 | 0.017 | 0.002 |
| Mexico | 0.002 | 0.060 | 0.010 | 0.002 | 0.002 | 0.060 | 0.060 | 0.063 | 0.012 | 0.012 | 0.070 | 0.012 | 0.012 | 0.070 | 0.119 | 0.119 | 0.119 | 0.119 | 0.020 | 0.010 | 0.022 | 0.005 |
| Moldova | 0.001 | 0.062 | 0.001 | 0.001 | 0.001 | 0.009 | 0.062 | 0.063 | 0.071 | 0.001 | 0.071 | 0.071 | 0.017 | 0.124 | 0.124 | 0.124 | 0.071 | 0.071 | 0.017 | 0.009 | 0.010 | 0.010 |
| Monaco | 0.001 | 0.006 | 0.001 | 0.001 | 0.001 | 0.006 | 0.006 | 0.007 | 0.011 | 0.006 | 0.130 | 0.066 | 0.011 | 0.130 | 0.130 | 0.130 | 0.130 | 0.130 | 0.071 | 0.006 | 0.007 | 0.002 |
| Mongolia | 0.001 | 0.066 | 0.005 | 0.001 | 0.066 | 0.066 | 0.066 | 0.002 | 0.071 | 0.001 | 0.067 | 0.067 | 0.005 | 0.071 | 0.132 | 0.132 | 0.071 | 0.071 | 0.009 | 0.005 | 0.010 | 0.010 |
| Montenegro | 0.001 | 0.007 | 0.001 | 0.001 | 0.007 | 0.007 | 0.007 | 0.008 | 0.008 | 0.001 | 0.128 | 0.071 | 0.014 | 0.128 | 0.128 | 0.128 | 0.128 | 0.128 | 0.014 | 0.064 | 0.008 | 0.002 |
| Morocco | 0.001 | 0.063 | 0.007 | 0.063 | 0.063 | 0.063 | 0.063 | 0.009 | 0.070 | 0.002 | 0.070 | 0.008 | 0.002 | 0.015 | 0.126 | 0.126 | 0.070 | 0.126 | 0.015 | 0.007 | 0.009 | 0.009 |
| Mozambique | 0.000 | 0.068 | 0.068 | 0.068 | 0.068 | 0.068 | 0.068 | 0.139 | 0.137 | 0.001 | 0.003 | 0.069 | 0.001 | 0.003 | 0.071 | 0.137 | 0.005 | 0.005 | 0.003 | 0.003 | 0.005 | 0.001 |
| Myanmar | 0.001 | 0.061 | 0.009 | 0.061 | 0.061 | 0.061 | 0.061 | 0.071 | 0.070 | 0.002 | 0.010 | 0.010 | 0.010 | 0.018 | 0.122 | 0.122 | 0.070 | 0.070 | 0.070 | 0.009 | 0.011 | 0.003 |
| Namibia | 0.000 | 0.069 | 0.002 | 0.000 | 0.069 | 0.069 | 0.069 | 0.207 | 0.071 | 0.001 | 0.071 | 0.003 | 0.001 | 0.003 | 0.138 | 0.138 | 0.004 | 0.071 | 0.004 | 0.002 | 0.003 | 0.001 |
| Nauru | 0.001 | 0.064 | 0.006 | 0.006 | 0.064 | 0.064 | 0.064 | 0.066 | 0.065 | 0.001 | 0.071 | 0.065 | 0.007 | 0.007 | 0.129 | 0.071 | 0.071 | 0.071 | 0.071 | 0.006 | 0.013 | 0.008 |
| Nepal | 0.001 | 0.060 | 0.010 | 0.060 | 0.060 | 0.060 | 0.060 | 0.063 | 0.070 | 0.003 | 0.011 | 0.011 | 0.003 | 0.011 | 0.120 | 0.120 | 0.020 | 0.070 | 0.120 | 0.001 | 0.021 | 0.021 |
| Netherlands | 0.001 | 0.008 | 0.001 | 0.001 | 0.001 | 0.008 | 0.008 | 0.009 | 0.009 | 0.009 | 0.126 | 0.071 | 0.016 | 0.126 | 0.126 | 0.126 | 0.071 | 0.126 | 0.126 | 0.008 | 0.009 | 0.002 |
| New Zealand | 0.001 | 0.063 | 0.001 | 0.001 | 0.001 | 0.008 | 0.008 | 0.009 | 0.009 | 0.009 | 0.126 | 0.016 | 0.016 | 0.126 | 0.126 | 0.126 | 0.071 | 0.126 | 0.126 | 0.008 | 0.009 | 0.002 |
| Nicaragua | 0.002 | 0.061 | 0.008 | 0.002 | 0.008 | 0.061 | 0.061 | 0.065 | 0.063 | 0.010 | 0.070 | 0.063 | 0.004 | 0.017 | 0.122 | 0.122 | 0.070 | 0.122 | 0.017 | 0.008 | 0.012 | 0.019 |
| Niger | 0.002 | 0.069 | 0.069 | 0.069 | 0.069 | 0.069 | 0.069 | 0.071 | 0.071 | 0.001 | 0.002 | 0.070 | 0.001 | 0.001 | 0.139 | 0.139 | 0.003 | 0.071 | 0.003 | 0.002 | 0.004 | 0.001 |
| Nigeria | 0.001 | 0.068 | 0.068 | 0.068 | 0.068 | 0.068 | 0.068 | 0.074 | 0.071 | 0.001 | 0.068 | 0.004 | 0.001 | 0.004 | 0.135 | 0.135 | 0.007 | 0.071 | 0.004 | 0.003 | 0.007 | 0.002 |
| Niue | 0.001 | 0.059 | 0.001 | 0.011 | 0.011 | 0.059 | 0.059 | 0.062 | 0.070 | 0.002 | 0.070 | 0.022 | 0.012 | 0.022 | 0.119 | 0.119 | 0.119 | 0.119 | 0.022 | 0.011 | 0.013 | 0.003 |
| North Korea | 0.001 | 0.062 | 0.001 | 0.009 | 0.009 | 0.062 | 0.062 | 0.011 | 0.071 | 0.002 | 0.071 | 0.018 | 0.010 | 0.071 | 0.123 | 0.123 | 0.071 | 0.071 | 0.123 | 0.009 | 0.011 | 0.003 |
| North Macedonia | 0.001 | 0.062 | 0.001 | 0.009 | 0.009 | 0.009 | 0.062 | 0.002 | 0.010 | 0.001 | 0.071 | 0.018 | 0.071 | 0.123 | 0.123 | 0.123 | 0.123 | 0.123 | 0.018 | 0.009 | 0.011 | 0.002 |
| Northern Mariana Islands | 0.001 | 0.009 | 0.001 | 0.009 | 0.009 | 0.061 | 0.061 | 0.011 | 0.062 | 0.002 | 0.070 | 0.070 | 0.070 | 0.070 | 0.122 | 0.122 | 0.070 | 0.122 | 0.019 | 0.009 | 0.011 | 0.003 |
| Norway | 0.001 | 0.008 | 0.001 | 0.001 | 0.001 | 0.008 | 0.008 | 0.010 | 0.009 | 0.009 | 0.125 | 0.063 | 0.016 | 0.125 | 0.125 | 0.125 | 0.071 | 0.125 | 0.071 | 0.062 | 0.010 | 0.010 |
| Oman | 0.001 | 0.063 | 0.007 | 0.001 | 0.063 | 0.063 | 0.063 | 0.009 | 0.064 | 0.008 | 0.071 | 0.008 | 0.001 | 0.071 | 0.127 | 0.127 | 0.071 | 0.071 | 0.015 | 0.063 | 0.015 | 0.009 |
| Pakistan | 0.001 | 0.066 | 0.066 | 0.066 | 0.066 | 0.066 | 0.066 | 0.006 | 0.071 | 0.005 | 0.071 | 0.005 | 0.005 | 0.005 | 0.132 | 0.132 | 0.071 | 0.071 | 0.009 | 0.001 | 0.010 | 0.002 |
| Palau | 0.001 | 0.008 | 0.008 | 0.008 | 0.008 | 0.008 | 0.063 | 0.064 | 0.071 | 0.001 | 0.071 | 0.071 | 0.001 | 0.071 | 0.125 | 0.125 | 0.071 | 0.125 | 0.071 | 0.008 | 0.009 | 0.002 |
| Palestine | 0.001 | 0.062 | 0.008 | 0.008 | 0.062 | 0.062 | 0.062 | 0.003 | 0.009 | 0.002 | 0.070 | 0.009 | 0.009 | 0.070 | 0.124 | 0.124 | 0.070 | 0.124 | 0.017 | 0.062 | 0.018 | 0.010 |
| Panama | 0.002 | 0.060 | 0.010 | 0.002 | 0.002 | 0.060 | 0.060 | 0.064 | 0.070 | 0.004 | 0.070 | 0.062 | 0.011 | 0.019 | 0.120 | 0.120 | 0.070 | 0.120 | 0.019 | 0.010 | 0.013 | 0.013 |
| Papua New Guinea | 0.001 | 0.066 | 0.005 | 0.066 | 0.066 | 0.066 | 0.066 | 0.075 | 0.071 | 0.001 | 0.071 | 0.005 | 0.001 | 0.005 | 0.133 | 0.071 | 0.071 | 0.071 | 0.071 | 0.005 | 0.010 | 0.002 |
| Paraguay | 0.002 | 0.058 | 0.012 | 0.002 | 0.012 | 0.058 | 0.058 | 0.061 | 0.059 | 0.004 | 0.070 | 0.059 | 0.014 | 0.024 | 0.115 | 0.115 | 0.070 | 0.070 | 0.024 | 0.058 | 0.016 | 0.016 |
| Peru | 0.002 | 0.057 | 0.012 | 0.002 | 0.057 | 0.057 | 0.057 | 0.062 | 0.069 | 0.005 | 0.059 | 0.069 | 0.015 | 0.069 | 0.114 | 0.069 | 0.024 | 0.114 | 0.015 | 0.012 | 0.017 | 0.027 |
| Philippines | 0.001 | 0.063 | 0.008 | 0.063 | 0.063 | 0.063 | 0.063 | 0.010 | 0.125 | 0.002 | 0.071 | 0.009 | 0.009 | 0.016 | 0.125 | 0.125 | 0.071 | 0.071 | 0.016 | 0.008 | 0.010 | 0.003 |
| Poland | 0.001 | 0.009 | 0.001 | 0.001 | 0.001 | 0.009 | 0.009 | 0.011 | 0.019 | 0.001 | 0.123 | 0.019 | 0.071 | 0.123 | 0.123 | 0.123 | 0.123 | 0.123 | 0.019 | 0.061 | 0.011 | 0.002 |
| Portugal | 0.001 | 0.009 | 0.001 | 0.001 | 0.001 | 0.009 | 0.009 | 0.063 | 0.018 | 0.001 | 0.123 | 0.018 | 0.018 | 0.123 | 0.123 | 0.123 | 0.123 | 0.071 | 0.071 | 0.062 | 0.010 | 0.002 |
| Puerto Rico | 0.001 | 0.009 | 0.001 | 0.001 | 0.001 | 0.009 | 0.009 | 0.063 | 0.010 | 0.002 | 0.122 | 0.071 | 0.019 | 0.122 | 0.122 | 0.122 | 0.122 | 0.122 | 0.019 | 0.009 | 0.011 | 0.011 |
| Qatar | 0.001 | 0.064 | 0.006 | 0.006 | 0.006 | 0.064 | 0.064 | 0.008 | 0.065 | 0.001 | 0.071 | 0.007 | 0.007 | 0.071 | 0.129 | 0.071 | 0.129 | 0.071 | 0.065 | 0.006 | 0.071 | 0.008 |
| Romania | 0.001 | 0.010 | 0.001 | 0.001 | 0.001 | 0.010 | 0.061 | 0.011 | 0.071 | 0.001 | 0.071 | 0.071 | 0.019 | 0.123 | 0.123 | 0.123 | 0.071 | 0.123 | 0.019 | 0.061 | 0.011 | 0.002 |
| Russia | 0.001 | 0.008 | 0.001 | 0.001 | 0.001 | 0.008 | 0.063 | 0.064 | 0.071 | 0.001 | 0.071 | 0.064 | 0.071 | 0.126 | 0.126 | 0.126 | 0.015 | 0.126 | 0.015 | 0.008 | 0.009 | 0.009 |
| Rwanda | 0.001 | 0.066 | 0.005 | 0.066 | 0.066 | 0.066 | 0.066 | 0.137 | 0.071 | 0.001 | 0.005 | 0.067 | 0.001 | 0.005 | 0.132 | 0.132 | 0.009 | 0.071 | 0.009 | 0.005 | 0.010 | 0.002 |
| Saint Kitts and Nevis | 0.001 | 0.059 | 0.011 | 0.001 | 0.001 | 0.059 | 0.059 | 0.071 | 0.012 | 0.003 | 0.070 | 0.070 | 0.012 | 0.070 | 0.118 | 0.118 | 0.070 | 0.118 | 0.022 | 0.011 | 0.014 | 0.014 |
| Saint Lucia | 0.001 | 0.058 | 0.012 | 0.001 | 0.001 | 0.058 | 0.058 | 0.061 | 0.013 | 0.002 | 0.070 | 0.070 | 0.013 | 0.024 | 0.117 | 0.117 | 0.117 | 0.117 | 0.024 | 0.012 | 0.014 | 0.014 |
| Saint Vincent and the Grenadines | 0.001 | 0.060 | 0.010 | 0.001 | 0.001 | 0.060 | 0.060 | 0.063 | 0.012 | 0.003 | 0.070 | 0.070 | 0.020 | 0.020 | 0.120 | 0.120 | 0.120 | 0.120 | 0.020 | 0.010 | 0.013 | 0.013 |
| Samoa | 0.001 | 0.061 | 0.001 | 0.061 | 0.009 | 0.061 | 0.061 | 0.063 | 0.070 | 0.002 | 0.070 | 0.062 | 0.010 | 0.018 | 0.122 | 0.122 | 0.070 | 0.070 | 0.018 | 0.009 | 0.020 | 0.003 |
| San Marino | 0.001 | 0.008 | 0.001 | 0.001 | 0.008 | 0.008 | 0.008 | 0.010 | 0.009 | 0.016 | 0.125 | 0.064 | 0.009 | 0.125 | 0.125 | 0.125 | 0.070 | 0.125 | 0.070 | 0.063 | 0.010 | 0.003 |
| Saudi Arabia | 0.001 | 0.064 | 0.007 | 0.001 | 0.007 | 0.064 | 0.007 | 0.008 | 0.071 | 0.002 | 0.065 | 0.008 | 0.008 | 0.071 | 0.128 | 0.128 | 0.071 | 0.128 | 0.071 | 0.007 | 0.071 | 0.008 |
| Senegal | 0.005 | 0.066 | 0.066 | 0.066 | 0.066 | 0.066 | 0.066 | 0.071 | 0.071 | 0.002 | 0.005 | 0.005 | 0.002 | 0.005 | 0.132 | 0.132 | 0.071 | 0.071 | 0.009 | 0.005 | 0.010 | 0.002 |
| Serbia | 0.001 | 0.007 | 0.001 | 0.001 | 0.007 | 0.007 | 0.007 | 0.009 | 0.008 | 0.001 | 0.127 | 0.071 | 0.015 | 0.127 | 0.127 | 0.127 | 0.127 | 0.127 | 0.071 | 0.007 | 0.009 | 0.002 |
| Seychelles | 0.002 | 0.058 | 0.002 | 0.012 | 0.012 | 0.012 | 0.058 | 0.061 | 0.070 | 0.003 | 0.070 | 0.070 | 0.014 | 0.070 | 0.115 | 0.115 | 0.070 | 0.115 | 0.025 | 0.002 | 0.016 | 0.005 |
| Sierra Leone | 0.004 | 0.067 | 0.067 | 0.067 | 0.067 | 0.067 | 0.067 | 0.134 | 0.071 | 0.001 | 0.005 | 0.005 | 0.001 | 0.005 | 0.133 | 0.133 | 0.008 | 0.071 | 0.008 | 0.004 | 0.009 | 0.002 |
| Singapore | 0.001 | 0.007 | 0.001 | 0.001 | 0.001 | 0.001 | 0.063 | 0.009 | 0.071 | 0.008 | 0.127 | 0.071 | 0.008 | 0.127 | 0.127 | 0.127 | 0.071 | 0.127 | 0.014 | 0.007 | 0.009 | 0.009 |
| Slovakia | 0.001 | 0.008 | 0.001 | 0.001 | 0.008 | 0.008 | 0.063 | 0.002 | 0.008 | 0.001 | 0.126 | 0.071 | 0.071 | 0.126 | 0.126 | 0.126 | 0.071 | 0.126 | 0.015 | 0.008 | 0.009 | 0.009 |
| Slovenia | 0.001 | 0.007 | 0.001 | 0.001 | 0.001 | 0.007 | 0.007 | 0.008 | 0.008 | 0.008 | 0.127 | 0.071 | 0.014 | 0.127 | 0.127 | 0.127 | 0.127 | 0.127 | 0.014 | 0.064 | 0.008 | 0.002 |
| Solomon Islands | 0.001 | 0.065 | 0.006 | 0.065 | 0.065 | 0.065 | 0.065 | 0.007 | 0.071 | 0.001 | 0.071 | 0.065 | 0.007 | 0.007 | 0.130 | 0.130 | 0.071 | 0.071 | 0.012 | 0.006 | 0.013 | 0.002 |
| Somalia | 0.002 | 0.069 | 0.069 | 0.069 | 0.069 | 0.069 | 0.069 | 0.071 | 0.137 | 0.001 | 0.003 | 0.069 | 0.001 | 0.003 | 0.071 | 0.137 | 0.005 | 0.071 | 0.005 | 0.002 | 0.005 | 0.001 |
| South Africa | 0.000 | 0.068 | 0.003 | 0.003 | 0.003 | 0.068 | 0.068 | 0.205 | 0.071 | 0.001 | 0.003 | 0.069 | 0.001 | 0.006 | 0.136 | 0.136 | 0.071 | 0.071 | 0.006 | 0.003 | 0.004 | 0.001 |
| South Korea | 0.001 | 0.008 | 0.001 | 0.001 | 0.008 | 0.001 | 0.008 | 0.002 | 0.071 | 0.009 | 0.071 | 0.071 | 0.009 | 0.125 | 0.125 | 0.125 | 0.125 | 0.125 | 0.016 | 0.063 | 0.010 | 0.010 |
| South Sudan | 0.001 | 0.068 | 0.068 | 0.068 | 0.068 | 0.068 | 0.068 | 0.136 | 0.136 | 0.001 | 0.004 | 0.004 | 0.001 | 0.004 | 0.071 | 0.136 | 0.006 | 0.071 | 0.006 | 0.003 | 0.007 | 0.002 |
| Spain | 0.001 | 0.008 | 0.001 | 0.001 | 0.001 | 0.008 | 0.008 | 0.064 | 0.009 | 0.009 | 0.124 | 0.071 | 0.017 | 0.124 | 0.124 | 0.124 | 0.124 | 0.071 | 0.071 | 0.008 | 0.010 | 0.002 |
| Sri Lanka | 0.002 | 0.060 | 0.002 | 0.010 | 0.010 | 0.060 | 0.060 | 0.013 | 0.070 | 0.003 | 0.070 | 0.012 | 0.012 | 0.070 | 0.119 | 0.119 | 0.119 | 0.119 | 0.021 | 0.010 | 0.022 | 0.005 |
| Sudan | 0.001 | 0.067 | 0.067 | 0.067 | 0.067 | 0.067 | 0.067 | 0.071 | 0.067 | 0.005 | 0.005 | 0.005 | 0.001 | 0.005 | 0.133 | 0.133 | 0.071 | 0.071 | 0.008 | 0.004 | 0.009 | 0.005 |
| Suriname | 0.001 | 0.061 | 0.009 | 0.001 | 0.009 | 0.061 | 0.061 | 0.071 | 0.010 | 0.003 | 0.070 | 0.070 | 0.003 | 0.070 | 0.122 | 0.122 | 0.070 | 0.122 | 0.018 | 0.009 | 0.012 | 0.012 |
| Sweden | 0.001 | 0.008 | 0.001 | 0.001 | 0.001 | 0.008 | 0.008 | 0.009 | 0.009 | 0.009 | 0.126 | 0.009 | 0.016 | 0.126 | 0.126 | 0.126 | 0.126 | 0.126 | 0.071 | 0.063 | 0.009 | 0.009 |
| Switzerland | 0.001 | 0.009 | 0.001 | 0.001 | 0.001 | 0.009 | 0.009 | 0.010 | 0.010 | 0.010 | 0.124 | 0.018 | 0.018 | 0.124 | 0.124 | 0.124 | 0.124 | 0.124 | 0.071 | 0.062 | 0.010 | 0.002 |
| Syria | 0.001 | 0.065 | 0.006 | 0.001 | 0.065 | 0.065 | 0.065 | 0.002 | 0.006 | 0.006 | 0.071 | 0.006 | 0.006 | 0.071 | 0.130 | 0.130 | 0.071 | 0.130 | 0.071 | 0.006 | 0.012 | 0.007 |
| São Tomé and PrÍncipe | 0.001 | 0.062 | 0.009 | 0.062 | 0.062 | 0.062 | 0.062 | 0.011 | 0.070 | 0.002 | 0.063 | 0.063 | 0.002 | 0.017 | 0.123 | 0.123 | 0.017 | 0.070 | 0.070 | 0.009 | 0.019 | 0.003 |
| Taiwan (province of China) | 0.001 | 0.007 | 0.001 | 0.001 | 0.001 | 0.007 | 0.007 | 0.008 | 0.071 | 0.001 | 0.071 | 0.071 | 0.071 | 0.128 | 0.128 | 0.128 | 0.128 | 0.128 | 0.014 | 0.007 | 0.008 | 0.002 |
| Tajikistan | 0.001 | 0.066 | 0.005 | 0.001 | 0.066 | 0.066 | 0.066 | 0.067 | 0.071 | 0.001 | 0.067 | 0.005 | 0.005 | 0.010 | 0.132 | 0.132 | 0.071 | 0.071 | 0.010 | 0.066 | 0.010 | 0.006 |

| | Met need for FP with modern contraception | Antenatal, peripartum and postnatal care for newborns | Antenatal, postpartum and postnatal care for mothers | MCV1 coverage | DTP3 coverage | Diarrhoea treatment | LRI treatment | ART coverage | TB treatment | Acute lymphoid leukaemia treatment | Breast cancer treatment | Cervical cancer treatment | Uterine cancer treatment | Colon/rectum cancer treatment | IHD treatment | Stroke treatment | Diabetes treatment | CKD treatment | COPD treatment | Asthma treatment | Epilepsy treatment | Appendicitis treatment |
|---|---|---|---|---|---|---|---|---|---|---|---|---|---|---|---|---|---|---|---|---|---|---|
| Tanzania | 0.001 | 0.067 | 0.067 | 0.067 | 0.067 | 0.067 | 0.067 | 0.138 | 0.071 | 0.001 | 0.004 | 0.004 | 0.001 | 0.004 | 0.134 | 0.134 | 0.007 | 0.071 | 0.004 | 0.004 | 0.008 | 0.002 |
| Thailand | 0.002 | 0.010 | 0.002 | 0.010 | 0.010 | 0.010 | 0.060 | 0.071 | 0.119 | 0.003 | 0.070 | 0.061 | 0.012 | 0.070 | 0.119 | 0.119 | 0.070 | 0.119 | 0.020 | 0.002 | 0.013 | 0.005 |
| The Bahamas | 0.001 | 0.062 | 0.009 | 0.001 | 0.001 | 0.062 | 0.062 | 0.079 | 0.010 | 0.002 | 0.070 | 0.063 | 0.010 | 0.070 | 0.123 | 0.123 | 0.070 | 0.123 | 0.017 | 0.009 | 0.011 | 0.011 |
| The Gambia | 0.005 | 0.066 | 0.066 | 0.066 | 0.066 | 0.066 | 0.066 | 0.071 | 0.132 | 0.001 | 0.005 | 0.005 | 0.001 | 0.005 | 0.132 | 0.132 | 0.009 | 0.071 | 0.009 | 0.005 | 0.010 | 0.002 |
| Timor-Leste | 0.001 | 0.064 | 0.006 | 0.006 | 0.064 | 0.064 | 0.064 | 0.076 | 0.128 | 0.002 | 0.008 | 0.008 | 0.002 | 0.013 | 0.128 | 0.128 | 0.070 | 0.128 | 0.013 | 0.006 | 0.009 | 0.004 |
| Togo | 0.001 | 0.067 | 0.067 | 0.004 | 0.067 | 0.067 | 0.067 | 0.138 | 0.071 | 0.001 | 0.005 | 0.067 | 0.001 | 0.005 | 0.133 | 0.133 | 0.008 | 0.071 | 0.008 | 0.004 | 0.009 | 0.002 |
| Tokelau | 0.001 | 0.058 | 0.012 | 0.058 | 0.012 | 0.058 | 0.058 | 0.060 | 0.070 | 0.002 | 0.070 | 0.013 | 0.013 | 0.025 | 0.116 | 0.116 | 0.116 | 0.070 | 0.025 | 0.012 | 0.015 | 0.004 |
| Tonga | 0.001 | 0.060 | 0.010 | 0.010 | 0.060 | 0.060 | 0.060 | 0.012 | 0.070 | 0.003 | 0.070 | 0.019 | 0.011 | 0.011 | 0.121 | 0.121 | 0.121 | 0.121 | 0.019 | 0.010 | 0.012 | 0.004 |
| Trinidad and Tobago | 0.001 | 0.062 | 0.001 | 0.001 | 0.001 | 0.062 | 0.062 | 0.064 | 0.010 | 0.002 | 0.070 | 0.017 | 0.017 | 0.070 | 0.123 | 0.123 | 0.123 | 0.123 | 0.017 | 0.009 | 0.011 | 0.011 |
| Tunisia | 0.001 | 0.061 | 0.009 | 0.009 | 0.009 | 0.061 | 0.061 | 0.011 | 0.010 | 0.002 | 0.070 | 0.010 | 0.010 | 0.070 | 0.123 | 0.123 | 0.123 | 0.123 | 0.070 | 0.009 | 0.011 | 0.011 |
| Turkey | 0.001 | 0.059 | 0.001 | 0.011 | 0.011 | 0.059 | 0.059 | 0.004 | 0.012 | 0.003 | 0.070 | 0.012 | 0.012 | 0.070 | 0.119 | 0.119 | 0.070 | 0.119 | 0.119 | 0.011 | 0.023 | 0.013 |
| Turkmenistan | 0.001 | 0.066 | 0.004 | 0.001 | 0.001 | 0.066 | 0.066 | 0.068 | 0.071 | 0.001 | 0.071 | 0.067 | 0.005 | 0.071 | 0.133 | 0.133 | 0.071 | 0.071 | 0.009 | 0.004 | 0.010 | 0.006 |
| Tuvalu | 0.001 | 0.060 | 0.010 | 0.010 | 0.010 | 0.060 | 0.060 | 0.062 | 0.070 | 0.002 | 0.070 | 0.012 | 0.012 | 0.021 | 0.120 | 0.120 | 0.120 | 0.070 | 0.070 | 0.010 | 0.013 | 0.003 |
| UK | 0.001 | 0.009 | 0.001 | 0.001 | 0.001 | 0.009 | 0.009 | 0.011 | 0.010 | 0.010 | 0.123 | 0.010 | 0.018 | 0.123 | 0.123 | 0.123 | 0.123 | 0.123 | 0.123 | 0.009 | 0.011 | 0.011 |
| USA | 0.001 | 0.010 | 0.001 | 0.001 | 0.001 | 0.010 | 0.010 | 0.062 | 0.010 | 0.001 | 0.122 | 0.020 | 0.020 | 0.122 | 0.122 | 0.122 | 0.071 | 0.122 | 0.122 | 0.010 | 0.011 | 0.011 |
| Uganda | 0.001 | 0.068 | 0.068 | 0.068 | 0.068 | 0.068 | 0.068 | 0.139 | 0.071 | 0.001 | 0.004 | 0.068 | 0.001 | 0.004 | 0.071 | 0.135 | 0.006 | 0.071 | 0.004 | 0.003 | 0.007 | 0.002 |
| Ukraine | 0.001 | 0.064 | 0.001 | 0.001 | 0.001 | 0.007 | 0.064 | 0.065 | 0.071 | 0.001 | 0.071 | 0.071 | 0.013 | 0.128 | 0.128 | 0.128 | 0.071 | 0.071 | 0.013 | 0.007 | 0.008 | 0.008 |
| United Arab Emirates | 0.000 | 0.065 | 0.006 | 0.000 | 0.006 | 0.065 | 0.006 | 0.066 | 0.066 | 0.001 | 0.071 | 0.006 | 0.006 | 0.071 | 0.131 | 0.131 | 0.071 | 0.071 | 0.071 | 0.006 | 0.071 | 0.006 |
| Uruguay | 0.001 | 0.057 | 0.001 | 0.001 | 0.001 | 0.013 | 0.057 | 0.059 | 0.015 | 0.003 | 0.114 | 0.070 | 0.027 | 0.114 | 0.114 | 0.114 | 0.027 | 0.070 | 0.070 | 0.013 | 0.016 | 0.016 |
| Uzbekistan | 0.001 | 0.065 | 0.005 | 0.001 | 0.001 | 0.065 | 0.065 | 0.067 | 0.071 | 0.001 | 0.071 | 0.066 | 0.006 | 0.071 | 0.131 | 0.131 | 0.071 | 0.071 | 0.011 | 0.005 | 0.011 | 0.007 |
| Vanuatu | 0.001 | 0.064 | 0.006 | 0.006 | 0.064 | 0.064 | 0.064 | 0.066 | 0.071 | 0.002 | 0.071 | 0.007 | 0.007 | 0.007 | 0.128 | 0.128 | 0.071 | 0.071 | 0.071 | 0.006 | 0.014 | 0.002 |
| Venezuela | 0.002 | 0.060 | 0.010 | 0.002 | 0.002 | 0.060 | 0.060 | 0.063 | 0.012 | 0.003 | 0.070 | 0.070 | 0.012 | 0.070 | 0.120 | 0.120 | 0.070 | 0.120 | 0.020 | 0.010 | 0.013 | 0.013 |
| Vietnam | 0.001 | 0.059 | 0.001 | 0.011 | 0.011 | 0.059 | 0.059 | 0.062 | 0.119 | 0.003 | 0.070 | 0.021 | 0.003 | 0.070 | 0.119 | 0.119 | 0.070 | 0.070 | 0.021 | 0.011 | 0.013 | 0.013 |
| Virgin Islands | 0.001 | 0.061 | 0.001 | 0.001 | 0.009 | 0.009 | 0.009 | 0.064 | 0.010 | 0.002 | 0.122 | 0.018 | 0.010 | 0.122 | 0.122 | 0.122 | 0.122 | 0.122 | 0.018 | 0.009 | 0.011 | 0.011 |
| Yemen | 0.001 | 0.068 | 0.068 | 0.068 | 0.068 | 0.068 | 0.068 | 0.004 | 0.071 | 0.001 | 0.068 | 0.004 | 0.001 | 0.004 | 0.135 | 0.135 | 0.071 | 0.071 | 0.007 | 0.003 | 0.007 | 0.007 |
| Zambia | 0.000 | 0.069 | 0.003 | 0.069 | 0.069 | 0.069 | 0.069 | 0.140 | 0.137 | 0.001 | 0.003 | 0.069 | 0.001 | 0.003 | 0.071 | 0.137 | 0.005 | 0.071 | 0.003 | 0.003 | 0.005 | 0.001 |
| Zimbabwe | 0.000 | 0.069 | 0.002 | 0.069 | 0.069 | 0.069 | 0.069 | 0.207 | 0.138 | 0.001 | 0.002 | 0.002 | 0.001 | 0.002 | 0.138 | 0.071 | 0.004 | 0.071 | 0.004 | 0.002 | 0.005 | 0.001 |

**Supplementary table 2. UHC effective coverage index values for 1990, 2010, and 2019, and annualised rates of change for 1990-2019, 1990-2010, and 2010-2019.** All estimates are accompanied by 95% uncertainty intervals in parentheses. Annualised rate of change is reported in terms of percentages. UHC=universal health coverage.

| | UHC effective coverage index | | | Annualised rate of change (%) | | |
|---|---|---|---|---|---|---|
| | **1990** | **2010** | **2019** | **1990-2019** | **1990-2010** | **2010-2019** |
| **Global** | **45.8 (44.2 to 47.5)** | **55.7 (54.4 to 57.0)** | **60.3 (58.7 to 61.9)** | **0.9 (0.8 to 1.1)** | **1.0 (0.8 to 1.1)** | **0.9 (0.6 to 1.2)** |
| **Central Europe, eastern Europe, and central Asia** | **51.3 (49.8 to 52.7)** | **57.0 (55.7 to 58.2)** | **64.0 (61.5 to 66.6)** | **0.8 (0.6 to 0.9)** | **0.5 (0.4 to 0.6)** | **1.3 (0.8 to 1.8)** |
| **Central Asia** | **39.3 (37.3 to 41.3)** | **41.9 (40.1 to 43.7)** | **49.0 (47.3 to 50.8)** | **0.8 (0.5 to 1.0)** | **0.3 (0.1 to 0.5)** | **1.7 (1.1 to 2.3)** |
| Armenia | 46.9 (44.8 to 49.1) | 55.7 (54.0 to 57.2) | 62.4 (59.5 to 65.6) | 1.0 (0.8 to 1.2) | 0.8 (0.7 to 1.0) | 1.3 (0.8 to 1.8) |
| Azerbaijan | 32.0 (29.2 to 34.6) | 40.3 (38.3 to 42.5) | 48.2 (44.9 to 51.5) | 1.4 (1.0 to 1.8) | 1.2 (0.8 to 1.6) | 2.0 (1.0 to 2.8) |
| Georgia | 43.6 (41.8 to 45.9) | 53.2 (50.6 to 55.9) | 55.9 (52.8 to 59.6) | 0.8 (0.6 to 1.1) | 1.0 (0.7 to 1.3) | 0.5 (-0.2 to 1.4) |
| Kazakhstan | 44.8 (42.4 to 47.1) | 50.0 (47.6 to 52.0) | 59.3 (56.2 to 62.1) | 1.0 (0.7 to 1.2) | 0.6 (0.3 to 0.8) | 1.9 (1.3 to 2.5) |
| Kyrgyzstan | 35.0 (32.3 to 38.0) | 42.8 (40.6 to 45.0) | 52.9 (49.9 to 56.1) | 1.4 (1.1 to 1.7) | 1.0 (0.7 to 1.3) | 2.4 (1.7 to 3.0) |
| Mongolia | 27.4 (22.6 to 31.5) | 42.3 (38.6 to 45.9) | 47.9 (44.2 to 51.7) | 1.9 (1.3 to 2.7) | 2.2 (1.3 to 3.2) | 1.4 (0.3 to 2.5) |
| Tajikistan | 41.6 (38.9 to 44.5) | 40.4 (37.4 to 43.8) | 47.9 (44.7 to 51.0) | 0.5 (0.1 to 0.8) | -0.2 (-0.6 to 0.3) | 1.9 (0.7 to 3.0) |
| Turkmenistan | 31.8 (29.7 to 34.4) | 39.0 (35.0 to 42.0) | 44.0 (39.9 to 47.9) | 1.1 (0.6 to 1.6) | 1.0 (0.5 to 1.4) | 1.3 (-0.1 to 2.9) |
| Uzbekistan | 38.1 (35.4 to 40.4) | 35.7 (32.8 to 38.4) | 42.2 (39.2 to 45.1) | 0.4 (0.0 to 0.7) | -0.3 (-0.7 to 0.1) | 1.9 (0.7 to 3.1) |
| **Central Europe** | **52.2 (50.7 to 53.6)** | **66.6 (65.3 to 67.8)** | **71.9 (68.1 to 75.3)** | **1.1 (0.9 to 1.3)** | **1.2 (1.1 to 1.3)** | **0.9 (0.2 to 1.4)** |
| Albania | 54.5 (52.6 to 56.2) | 68.4 (65.8 to 71.1) | 69.7 (64.5 to 74.5) | 0.8 (0.5 to 1.1) | 1.1 (0.9 to 1.4) | 0.2 (-0.6 to 1.0) |
| Bosnia and Herzegovina | 54.2 (52.1 to 56.1) | 61.6 (59.7 to 63.3) | 64.3 (59.3 to 68.7) | 0.6 (0.3 to 0.9) | 0.6 (0.5 to 0.8) | 0.5 (-0.4 to 1.3) |
| Bulgaria | 52.4 (50.7 to 54.3) | 56.3 (54.4 to 58.0) | 62.6 (58.0 to 67.1) | 0.6 (0.3 to 0.9) | 0.4 (0.2 to 0.5) | 1.2 (0.3 to 2.0) |
| Croatia | 61.0 (59.3 to 62.7) | 73.2 (71.6 to 74.6) | 79.0 (74.0 to 83.3) | 0.9 (0.7 to 1.1) | 0.9 (0.8 to 1.0) | 0.8 (0.1 to 1.5) |
| Czech Republic | 55.9 (54.4 to 57.4) | 77.7 (76.3 to 79.0) | 82.1 (77.6 to 85.9) | 1.3 (1.1 to 1.5) | 1.6 (1.5 to 1.8) | 0.6 (-0.0 to 1.2) |
| Hungary | 53.8 (52.0 to 55.4) | 67.1 (65.6 to 68.6) | 72.0 (67.5 to 76.3) | 1.0 (0.8 to 1.2) | 1.1 (1.0 to 1.2) | 0.8 (0.1 to 1.4) |
| Montenegro | 63.3 (61.4 to 65.2) | 63.5 (61.9 to 65.1) | 66.0 (62.5 to 69.4) | 0.1 (-0.1 to 0.3) | 0.0 (-0.1 to 0.2) | 0.4 (-0.2 to 1.0) |
| North Macedonia | 49.6 (47.3 to 51.7) | 55.7 (53.9 to 57.4) | 60.8 (55.9 to 65.4) | 0.7 (0.4 to 1.0) | 0.6 (0.4 to 0.8) | 1.0 (0.0 to 1.8) |
| Poland | 49.8 (48.2 to 51.5) | 68.3 (66.8 to 69.6) | 72.7 (64.3 to 80.5) | 1.3 (0.9 to 1.7) | 1.6 (1.5 to 1.7) | 0.7 (-0.7 to 1.8) |
| Romania | 50.1 (48.1 to 52.0) | 62.6 (60.8 to 64.0) | 69.6 (66.3 to 72.7) | 1.1 (0.9 to 1.4) | 1.1 (1.0 to 1.2) | 1.2 (0.5 to 1.8) |
| Serbia | 49.2 (47.1 to 51.0) | 57.0 (55.3 to 58.6) | 63.3 (58.6 to 67.9) | 0.9 (0.6 to 1.2) | 0.7 (0.6 to 0.9) | 1.2 (0.3 to 2.0) |
| Slovakia | 56.2 (54.6 to 57.8) | 72.3 (71.0 to 73.8) | 78.1 (73.2 to 82.5) | 1.1 (0.9 to 1.3) | 1.3 (1.2 to 1.4) | 0.8 (0.1 to 1.4) |
| Slovenia | 67.1 (62.3 to 71.4) | 84.9 (83.4 to 86.3) | 90.0 (85.5 to 93.0) | 1.0 (0.7 to 1.3) | 1.2 (0.8 to 1.6) | 0.6 (0.1 to 1.1) |
| **Eastern Europe** | **54.4 (52.8 to 55.9)** | **57.5 (56.2 to 58.9)** | **66.5 (61.8 to 70.6)** | **0.7 (0.4 to 0.9)** | **0.3 (0.1 to 0.4)** | **1.6 (0.8 to 2.3)** |
| Belarus | 57.1 (55.5 to 59.2) | 58.5 (57.2 to 59.9) | 70.5 (66.7 to 73.8) | 0.7 (0.5 to 0.9) | 0.1 (-0.0 to 0.2) | 2.1 (1.3 to 2.7) |
| Estonia | 60.1 (58.5 to 61.7) | 74.9 (72.2 to 76.6) | 82.0 (77.6 to 86.3) | 1.1 (0.8 to 1.3) | 1.1 (0.9 to 1.2) | 1.0 (0.4 to 1.6) |
| Latvia | 55.4 (53.9 to 56.9) | 62.0 (60.4 to 63.6) | 69.8 (66.8 to 72.6) | 0.8 (0.6 to 1.0) | 0.6 (0.4 to 0.7) | 1.3 (0.7 to 1.9) |
| Lithuania | 60.6 (59.0 to 62.2) | 65.9 (64.3 to 67.5) | 70.4 (66.7 to 73.7) | 0.5 (0.3 to 0.7) | 0.4 (0.3 to 0.5) | 0.7 (0.0 to 1.4) |
| Moldova | 48.5 (46.8 to 50.2) | 48.6 (47.1 to 50.2) | 62.2 (59.4 to 64.9) | 0.9 (0.7 to 1.0) | 0.0 (-0.2 to 0.2) | 2.7 (2.3 to 3.2) |
| Russia | 54.2 (52.6 to 55.9) | 57.6 (56.2 to 59.1) | 69.1 (63.1 to 74.2) | 0.8 (0.5 to 1.1) | 0.3 (0.2 to 0.4) | 2.0 (1.0 to 2.8) |
| Ukraine | 54.0 (52.5 to 56.0) | 56.4 (55.1 to 57.7) | 56.8 (48.4 to 64.2) | 0.2 (-0.4 to 0.6) | 0.2 (0.1 to 0.3) | 0.1 (-1.7 to 1.4) |
| **High income** | **70.1 (69.0 to 71.1)** | **84.1 (83.1 to 84.9)** | **86.1 (84.4 to 87.3)** | **0.7 (0.7 to 0.7)** | **0.9 (0.9 to 0.9)** | **0.3 (0.1 to 0.4)** |
| **Australasia** | **71.5 (70.2 to 72.8)** | **87.3 (85.9 to 88.5)** | **88.5 (86.5 to 89.7)** | **0.7 (0.7 to 0.8)** | **1.0 (0.9 to 1.1)** | **0.1 (0.0 to 0.3)** |
| Australia | 72.2 (70.9 to 73.5) | 88.3 (86.9 to 89.5) | 89.5 (87.3 to 90.8) | 0.7 (0.7 to 0.8) | 1.0 (0.9 to 1.1) | 0.2 (-0.0 to 0.3) |
| New Zealand | 68.1 (66.6 to 69.5) | 82.3 (81.0 to 83.6) | 83.0 (80.8 to 84.9) | 0.7 (0.6 to 0.8) | 0.9 (0.8 to 1.1) | 0.1 (-0.2 to 0.3) |
| **High-income Asia Pacific** | **75.2 (73.8 to 76.4)** | **91.1 (90.0 to 92.0)** | **94.1 (92.8 to 95.1)** | **0.8 (0.7 to 0.8)** | **1.0 (0.9 to 1.0)** | **0.4 (0.3 to 0.4)** |

| | UHC effective coverage index | | | Annualised rate of change (%) | | |
|---|---|---|---|---|---|---|
| | **1990** | **2010** | **2019** | **1990-2019** | **1990-2010** | **2010-2019** |
| Brunei | 52.3 (49.5 to 55.2) | 63.1 (60.8 to 65.4) | 65.6 (62.6 to 67.9) | 0.8 (0.5 to 1.0) | 0.9 (0.6 to 1.3) | 0.4 (-0.1 to 0.9) |
| Japan | 80.9 (79.5 to 82.1) | 93.6 (92.5 to 94.7) | 96.4 (95.0 to 97.4) | 0.6 (0.5 to 0.7) | 0.7 (0.7 to 0.8) | 0.3 (0.2 to 0.4) |
| South Korea | 59.7 (57.8 to 61.4) | 84.8 (83.1 to 86.0) | 89.2 (87.3 to 90.5) | 1.4 (1.3 to 1.5) | 1.7 (1.6 to 1.9) | 0.6 (0.4 to 0.7) |
| Singapore | 66.5 (64.7 to 68.2) | 89.5 (88.1 to 90.7) | 92.6 (90.1 to 93.9) | 1.1 (1.0 to 1.2) | 1.5 (1.4 to 1.6) | 0.4 (0.1 to 0.6) |
| **High-income North America** | **74.0 (72.9 to 75.1)** | **81.9 (80.8 to 83.0)** | **83.0 (81.2 to 84.4)** | **0.4 (0.3 to 0.4)** | **0.5 (0.5 to 0.6)** | **0.2 (-0.0 to 0.3)** |
| Canada | 79.8 (78.4 to 81.2) | 88.1 (86.9 to 89.2) | 90.4 (88.4 to 91.7) | 0.4 (0.4 to 0.5) | 0.5 (0.4 to 0.6) | 0.3 (0.1 to 0.4) |
| Greenland | 54.1 (51.9 to 56.4) | 63.5 (61.2 to 66.2) | 68.8 (64.6 to 72.9) | 0.8 (0.6 to 1.1) | 0.8 (0.6 to 1.0) | 0.9 (0.2 to 1.5) |
| USA | 73.4 (72.3 to 74.5) | 81.2 (80.0 to 82.4) | 82.2 (80.4 to 83.6) | 0.4 (0.3 to 0.4) | 0.5 (0.5 to 0.6) | 0.1 (-0.1 to 0.2) |
| **Southern Latin America** | **49.5 (48.0 to 51.1)** | **61.7 (60.1 to 63.5)** | **65.4 (59.9 to 69.3)** | **1.0 (0.7 to 1.1)** | **1.1 (1.0 to 1.2)** | **0.6 (-0.2 to 1.2)** |
| Argentina | 46.6 (44.8 to 48.3) | 58.9 (57.2 to 60.8) | 61.4 (55.6 to 65.7) | 0.9 (0.6 to 1.2) | 1.2 (1.0 to 1.3) | 0.4 (-0.5 to 1.1) |
| Chile | 55.9 (54.1 to 57.9) | 68.2 (66.2 to 69.9) | 74.6 (69.7 to 77.8) | 1.0 (0.8 to 1.1) | 1.0 (0.8 to 1.1) | 1.0 (0.4 to 1.4) |
| Uruguay | 53.4 (51.6 to 55.2) | 64.2 (62.5 to 65.9) | 68.8 (62.8 to 73.1) | 0.9 (0.6 to 1.1) | 0.9 (0.7 to 1.1) | 0.8 (-0.2 to 1.4) |
| **Western Europe** | **67.5 (66.3 to 68.6)** | **85.8 (84.8 to 86.6)** | **88.3 (86.0 to 89.9)** | **0.9 (0.9 to 1.0)** | **1.2 (1.1 to 1.3)** | **0.3 (0.1 to 0.5)** |
| Andorra | 80.9 (75.6 to 84.3) | 90.8 (87.2 to 93.3) | 91.9 (87.6 to 94.5) | 0.4 (0.2 to 0.7) | 0.6 (0.3 to 0.9) | 0.1 (-0.3 to 0.5) |
| Austria | 71.2 (69.6 to 72.6) | 82.3 (81.1 to 83.5) | 86.5 (83.5 to 88.6) | 0.7 (0.6 to 0.8) | 0.7 (0.6 to 0.8) | 0.5 (0.2 to 0.8) |
| Belgium | 67.6 (66.0 to 69.0) | 83.4 (82.1 to 84.8) | 87.5 (83.9 to 90.0) | 0.9 (0.8 to 1.0) | 1.1 (1.0 to 1.1) | 0.5 (0.1 to 0.8) |
| Cyprus | 56.9 (54.8 to 58.9) | 74.8 (73.0 to 76.5) | 79.6 (77.6 to 81.7) | 1.2 (1.0 to 1.3) | 1.4 (1.2 to 1.5) | 0.7 (0.4 to 1.0) |
| Denmark | 65.3 (63.4 to 67.1) | 80.8 (79.3 to 82.3) | 84.3 (80.6 to 86.7) | 0.9 (0.7 to 1.0) | 1.1 (1.0 to 1.2) | 0.5 (0.0 to 0.8) |
| Finland | 71.4 (69.8 to 73.0) | 88.5 (87.2 to 89.8) | 91.4 (89.0 to 92.9) | 0.8 (0.8 to 0.9) | 1.1 (1.0 to 1.2) | 0.4 (0.1 to 0.5) |
| France | 66.2 (64.5 to 67.8) | 88.0 (86.8 to 89.0) | 91.0 (86.6 to 93.6) | 1.1 (0.9 to 1.2) | 1.4 (1.3 to 1.5) | 0.4 (-0.2 to 0.7) |
| Germany | 66.5 (65.1 to 67.9) | 83.5 (82.1 to 84.7) | 86.5 (82.1 to 89.1) | 0.9 (0.7 to 1.0) | 1.1 (1.0 to 1.2) | 0.4 (-0.1 to 0.7) |
| Greece | 66.1 (64.4 to 67.8) | 79.9 (78.3 to 81.6) | 80.3 (76.3 to 82.9) | 0.7 (0.5 to 0.8) | 1.0 (0.9 to 1.0) | 0.0 (-0.4 to 0.4) |
| Iceland | 78.2 (76.3 to 80.2) | 93.8 (92.5 to 95.2) | 95.3 (93.6 to 96.8) | 0.7 (0.6 to 0.8) | 0.9 (0.8 to 1.1) | 0.2 (-0.0 to 0.4) |
| Ireland | 63.2 (61.5 to 65.0) | 86.3 (84.7 to 87.6) | 90.6 (87.3 to 92.3) | 1.2 (1.1 to 1.3) | 1.6 (1.4 to 1.7) | 0.5 (0.1 to 0.8) |
| Israel | 59.5 (57.8 to 61.1) | 78.6 (77.1 to 80.0) | 81.6 (77.8 to 84.1) | 1.1 (0.9 to 1.2) | 1.4 (1.3 to 1.5) | 0.4 (-0.1 to 0.7) |
| Italy | 70.4 (69.0 to 71.6) | 86.8 (85.6 to 88.2) | 89.0 (86.2 to 90.7) | 0.8 (0.7 to 0.9) | 1.0 (1.0 to 1.1) | 0.3 (-0.0 to 0.4) |
| Luxembourg | 66.2 (64.3 to 68.1) | 87.5 (85.6 to 89.3) | 91.5 (90.3 to 92.5) | 1.1 (1.0 to 1.2) | 1.4 (1.2 to 1.6) | 0.5 (0.3 to 0.7) |
| Malta | 60.0 (57.7 to 62.2) | 77.5 (75.9 to 79.0) | 82.9 (80.8 to 84.6) | 1.1 (1.0 to 1.3) | 1.3 (1.1 to 1.5) | 0.8 (0.5 to 1.0) |
| Monaco | 78.2 (74.7 to 82.1) | 88.7 (85.9 to 91.6) | 91.2 (88.5 to 94.8) | 0.5 (0.3 to 0.7) | 0.6 (0.4 to 0.9) | 0.3 (-0.0 to 0.7) |
| Netherlands | 69.8 (68.2 to 71.3) | 87.6 (86.1 to 89.1) | 89.9 (86.0 to 91.7) | 0.9 (0.7 to 0.9) | 1.1 (1.0 to 1.2) | 0.3 (-0.1 to 0.5) |
| Norway | 72.0 (70.4 to 73.5) | 90.2 (88.7 to 91.4) | 94.4 (91.3 to 96.1) | 0.9 (0.8 to 1.0) | 1.1 (1.0 to 1.2) | 0.5 (0.2 to 0.7) |
| Portugal | 60.8 (59.1 to 62.4) | 79.2 (77.5 to 80.7) | 83.8 (80.3 to 85.8) | 1.1 (1.0 to 1.2) | 1.3 (1.2 to 1.4) | 0.6 (0.2 to 0.9) |
| San Marino | 83.1 (80.1 to 85.7) | 91.9 (85.8 to 96.1) | 93.4 (84.8 to 97.3) | 0.4 (0.1 to 0.6) | 0.5 (0.1 to 0.8) | 0.2 (-0.5 to 0.8) |
| Spain | 65.0 (63.6 to 66.4) | 88.4 (87.1 to 89.7) | 90.3 (87.1 to 91.6) | 1.1 (1.0 to 1.2) | 1.5 (1.4 to 1.6) | 0.2 (-0.1 to 0.4) |
| Sweden | 78.8 (77.5 to 80.0) | 88.3 (87.1 to 89.4) | 90.4 (88.3 to 92.2) | 0.5 (0.4 to 0.5) | 0.6 (0.5 to 0.6) | 0.3 (0.0 to 0.5) |
| Switzerland | 77.2 (75.7 to 78.6) | 90.7 (89.4 to 91.9) | 93.7 (91.0 to 95.0) | 0.7 (0.6 to 0.7) | 0.8 (0.7 to 0.9) | 0.4 (0.1 to 0.5) |
| UK | 67.2 (65.9 to 68.4) | 86.0 (84.9 to 87.1) | 88.0 (85.2 to 89.9) | 0.9 (0.8 to 1.0) | 1.2 (1.2 to 1.3) | 0.3 (-0.1 to 0.5) |
| **Latin America and Caribbean** | **45.3 (43.5 to 47.2)** | **58.6 (57.4 to 59.9)** | **63.8 (61.5 to 65.9)** | **1.2 (1.0 to 1.3)** | **1.3 (1.1 to 1.4)** | **0.9 (0.6 to 1.3)** |
| **Andean Latin America** | **48.1 (45.0 to 51.4)** | **59.2 (57.1 to 61.1)** | **68.3 (64.4 to 71.5)** | **1.2 (0.9 to 1.5)** | **1.0 (0.7 to 1.4)** | **1.6 (0.9 to 2.2)** |
| Bolivia | 29.0 (23.8 to 34.3) | 44.8 (40.9 to 49.4) | 52.5 (47.2 to 58.0) | 2.0 (1.4 to 2.8) | 2.2 (1.3 to 3.1) | 1.7 (0.6 to 2.7) |
| Ecuador | 51.5 (47.5 to 54.6) | 57.2 (54.8 to 59.5) | 64.5 (60.4 to 68.4) | 0.8 (0.4 to 1.1) | 0.5 (0.2 to 0.9) | 1.3 (0.5 to 2.1) |

| | UHC effective coverage index | | | Annualised rate of change (%) | | |
|---|---|---|---|---|---|---|
| | 1990 | 2010 | 2019 | 1990-2019 | 1990-2010 | 2010-2019 |
| Peru | 52.3 (48.1 to 56.4) | 65.2 (62.2 to 68.1) | 76.0 (70.4 to 79.9) | 1.3 (0.9 to 1.7) | 1.1 (0.7 to 1.5) | 1.7 (0.8 to 2.5) |
| **Caribbean** | **41.8 (40.2 to 43.4)** | **51.7 (50.3 to 53.2)** | **55.1 (52.3 to 57.8)** | **1.0 (0.7 to 1.2)** | **1.1 (0.8 to 1.3)** | **0.7 (0.0 to 1.3)** |
| Antigua and Barbuda | 47.1 (45.4 to 48.9) | 59.9 (58.1 to 61.6) | 59.7 (56.5 to 62.7) | 0.8 (0.6 to 1.0) | 1.2 (1.0 to 1.4) | -0.0 (-0.7 to 0.5) |
| The Bahamas | 45.5 (43.3 to 47.9) | 59.8 (57.7 to 61.8) | 60.6 (56.9 to 64.3) | 1.0 (0.7 to 1.3) | 1.4 (1.1 to 1.6) | 0.1 (-0.6 to 0.8) |
| Barbados | 46.6 (44.8 to 48.4) | 60.8 (59.2 to 62.5) | 61.3 (57.5 to 65.0) | 0.9 (0.7 to 1.2) | 1.3 (1.2 to 1.5) | 0.1 (-0.6 to 0.7) |
| Belize | 39.5 (36.5 to 42.2) | 52.1 (49.9 to 54.4) | 54.3 (51.2 to 57.2) | 1.1 (0.8 to 1.4) | 1.4 (1.1 to 1.7) | 0.4 (-0.3 to 1.1) |
| Bermuda | 55.9 (54.3 to 57.4) | 73.8 (71.8 to 75.4) | 77.7 (73.3 to 80.8) | 1.1 (0.9 to 1.3) | 1.4 (1.2 to 1.6) | 0.6 (0.0 to 1.0) |
| Cuba | 59.4 (57.8 to 60.8) | 70.9 (69.4 to 72.4) | 72.5 (69.0 to 76.4) | 0.7 (0.5 to 0.9) | 0.9 (0.8 to 1.0) | 0.3 (-0.4 to 0.9) |
| Dominica | 43.9 (41.7 to 46.4) | 50.9 (48.0 to 53.4) | 51.9 (47.2 to 56.0) | 0.6 (0.2 to 0.9) | 0.7 (0.3 to 1.1) | 0.2 (-0.7 to 1.0) |
| Dominican Republic | 37.6 (34.2 to 40.9) | 48.4 (44.8 to 52.1) | 52.7 (46.6 to 58.0) | 1.2 (0.7 to 1.6) | 1.3 (0.6 to 1.9) | 0.9 (-0.7 to 2.3) |
| Grenada | 33.9 (31.6 to 36.0) | 48.4 (46.5 to 50.2) | 50.5 (48.3 to 52.8) | 1.4 (1.1 to 1.6) | 1.8 (1.5 to 2.1) | 0.5 (0.0 to 1.0) |
| Guyana | 23.2 (20.1 to 26.5) | 33.1 (30.3 to 36.0) | 40.7 (34.9 to 46.3) | 1.9 (1.3 to 2.6) | 1.8 (1.0 to 2.6) | 2.3 (0.6 to 3.7) |
| Haiti | 11.8 (9.0 to 15.3) | 25.0 (21.0 to 29.0) | 35.8 (30.5 to 41.2) | 3.8 (2.8 to 4.9) | 3.8 (2.4 to 5.2) | 4.0 (2.5 to 5.5) |
| Jamaica | 44.7 (42.8 to 46.5) | 57.7 (55.9 to 59.3) | 56.9 (51.9 to 61.5) | 0.8 (0.5 to 1.1) | 1.3 (1.1 to 1.5) | -0.1 (-1.1 to 0.7) |
| Puerto Rico | 53.4 (51.4 to 55.2) | 71.7 (70.0 to 73.2) | 75.8 (70.4 to 79.9) | 1.2 (0.9 to 1.4) | 1.5 (1.3 to 1.6) | 0.6 (-0.2 to 1.3) |
| Saint Kitts and Nevis | 35.7 (33.7 to 37.4) | 55.4 (53.5 to 57.3) | 53.0 (49.4 to 56.0) | 1.4 (1.1 to 1.6) | 2.2 (1.9 to 2.5) | -0.5 (-1.3 to 0.1) |
| Saint Lucia | 39.9 (37.7 to 42.2) | 58.0 (56.3 to 59.8) | 59.2 (55.4 to 62.6) | 1.4 (1.1 to 1.6) | 1.9 (1.6 to 2.1) | 0.2 (-0.5 to 0.9) |
| Saint Vincent and the Grenadines | 37.9 (35.8 to 39.8) | 49.7 (47.6 to 51.9) | 49.6 (46.4 to 52.6) | 0.9 (0.7 to 1.2) | 1.4 (1.1 to 1.6) | -0.1 (-0.8 to 0.7) |
| Suriname | 36.4 (33.8 to 39.4) | 48.9 (47.0 to 50.6) | 50.2 (46.0 to 54.0) | 1.1 (0.7 to 1.5) | 1.5 (1.1 to 1.8) | 0.3 (-0.7 to 1.1) |
| Trinidad and Tobago | 37.5 (35.4 to 39.7) | 51.9 (50.1 to 53.6) | 55.6 (49.3 to 61.4) | 1.4 (0.9 to 1.7) | 1.6 (1.4 to 1.8) | 0.8 (-0.6 to 1.9) |
| Virgin Islands | 47.6 (44.4 to 50.9) | 52.6 (50.6 to 54.7) | 53.7 (50.6 to 56.9) | 0.4 (0.1 to 0.7) | 0.5 (0.1 to 0.9) | 0.2 (-0.3 to 0.8) |
| **Central Latin America** | **48.7 (46.9 to 50.5)** | **59.2 (57.7 to 60.7)** | **63.3 (59.8 to 66.9)** | **0.9 (0.7 to 1.1)** | **1.0 (0.9 to 1.1)** | **0.7 (0.1 to 1.4)** |
| Colombia | 55.7 (53.8 to 57.4) | 66.4 (64.8 to 67.9) | 74.5 (69.2 to 79.0) | 1.0 (0.7 to 1.2) | 0.9 (0.7 to 1.0) | 1.3 (0.5 to 2.0) |
| Costa Rica | 65.8 (63.8 to 67.6) | 74.0 (72.0 to 75.7) | 79.1 (74.2 to 83.1) | 0.6 (0.4 to 0.8) | 0.6 (0.4 to 0.7) | 0.8 (-0.0 to 1.5) |
| El Salvador | 41.6 (39.1 to 44.2) | 56.7 (54.6 to 58.4) | 61.7 (56.3 to 66.6) | 1.4 (1.0 to 1.7) | 1.5 (1.3 to 1.8) | 0.9 (-0.1 to 1.9) |
| Guatemala | 39.5 (36.7 to 42.8) | 47.2 (43.3 to 51.0) | 52.2 (46.1 to 57.8) | 1.0 (0.4 to 1.4) | 0.9 (0.4 to 1.4) | 1.1 (-0.6 to 2.6) |
| Honduras | 46.9 (43.5 to 50.2) | 52.7 (48.8 to 57.2) | 54.5 (49.2 to 58.4) | 0.5 (0.1 to 0.8) | 0.6 (0.1 to 1.1) | 0.3 (-0.8 to 1.3) |
| Mexico | 46.2 (44.3 to 48.1) | 57.8 (55.9 to 59.6) | 61.4 (56.4 to 66.8) | 1.0 (0.7 to 1.3) | 1.1 (1.0 to 1.3) | 0.7 (-0.2 to 1.5) |
| Nicaragua | 47.4 (44.7 to 49.8) | 54.1 (52.3 to 56.1) | 57.1 (54.0 to 60.8) | 0.6 (0.4 to 0.9) | 0.7 (0.4 to 0.9) | 0.6 (-0.2 to 1.4) |
| Panama | 53.9 (52.2 to 55.9) | 63.9 (62.1 to 65.7) | 71.3 (65.4 to 76.3) | 1.0 (0.6 to 1.2) | 0.8 (0.7 to 1.0) | 1.2 (0.3 to 2.1) |
| Venezuela | 51.2 (47.3 to 54.0) | 60.3 (58.4 to 62.3) | 61.1 (55.1 to 66.3) | 0.6 (0.2 to 1.0) | 0.8 (0.6 to 1.2) | 0.1 (-1.0 to 1.1) |
| **Tropical Latin America** | **42.0 (39.8 to 44.2)** | **59.1 (57.6 to 60.8)** | **64.8 (63.0 to 66.7)** | **1.5 (1.3 to 1.7)** | **1.7 (1.5 to 2.0)** | **1.0 (0.8 to 1.3)** |
| Brazil | 41.6 (39.5 to 43.9) | 59.1 (57.6 to 60.8) | 64.8 (63.0 to 66.7) | 1.5 (1.4 to 1.7) | 1.7 (1.5 to 2.0) | 1.0 (0.8 to 1.3) |
| Paraguay | 53.3 (50.6 to 55.9) | 59.5 (57.3 to 61.8) | 63.4 (57.9 to 68.2) | 0.6 (0.2 to 0.9) | 0.6 (0.3 to 0.8) | 0.7 (-0.3 to 1.6) |
| **North Africa and Middle East** | **44.4 (43.0 to 45.8)** | **55.6 (54.5 to 56.7)** | **60.5 (58.3 to 62.6)** | **1.1 (0.9 to 1.2)** | **1.1 (1.0 to 1.3)** | **0.9 (0.6 to 1.3)** |
| **North Africa and Middle East** | **44.4 (43.0 to 45.8)** | **55.6 (54.5 to 56.7)** | **60.5 (58.3 to 62.6)** | **1.1 (0.9 to 1.2)** | **1.1 (1.0 to 1.3)** | **0.9 (0.6 to 1.3)** |
| Afghanistan | 21.9 (17.1 to 27.2) | 31.0 (25.5 to 36.4) | 39.2 (33.6 to 45.1) | 2.0 (1.3 to 2.9) | 1.7 (0.6 to 3.0) | 2.6 (1.2 to 4.0) |
| Algeria | 48.7 (45.3 to 51.9) | 62.5 (59.5 to 65.5) | 64.8 (61.4 to 68.3) | 1.0 (0.7 to 1.3) | 1.3 (0.9 to 1.7) | 0.4 (-0.3 to 1.2) |
| Bahrain | 54.8 (52.2 to 57.4) | 66.3 (64.1 to 68.6) | 70.6 (66.4 to 74.5) | 0.9 (0.6 to 1.1) | 1.0 (0.7 to 1.2) | 0.7 (0.0 to 1.4) |
| Egypt | 38.2 (35.1 to 40.9) | 49.9 (47.4 to 52.8) | 54.7 (49.0 to 60.6) | 1.2 (0.8 to 1.7) | 1.3 (1.1 to 1.7) | 1.0 (-0.3 to 2.2) |
| Iran | 52.6 (50.1 to 54.8) | 62.1 (60.3 to 63.8) | 69.6 (67.4 to 71.4) | 1.0 (0.8 to 1.1) | 0.8 (0.7 to 1.0) | 1.3 (1.0 to 1.5) |

| | UHC effective coverage index | | | Annualised rate of change (%) | | |
|---|---|---|---|---|---|---|
| | 1990 | 2010 | 2019 | 1990-2019 | 1990-2010 | 2010-2019 |
| Iraq | 45.4 (42.5 to 48.2) | 50.5 (46.6 to 55.0) | 57.7 (53.5 to 62.4) | 0.8 (0.5 to 1.2) | 0.6 (0.1 to 1.0) | 1.5 (0.7 to 2.2) |
| Jordan | 53.0 (49.8 to 56.4) | 68.0 (65.4 to 70.8) | 70.0 (66.9 to 72.8) | 1.0 (0.7 to 1.2) | 1.2 (0.9 to 1.6) | 0.3 (-0.3 to 0.9) |
| Kuwait | 65.4 (61.7 to 67.9) | 75.1 (73.4 to 76.7) | 81.9 (78.6 to 84.9) | 0.8 (0.6 to 1.0) | 0.7 (0.5 to 1.0) | 1.0 (0.4 to 1.4) |
| Lebanon | 54.3 (51.4 to 56.7) | 69.0 (67.0 to 71.3) | 74.5 (72.5 to 76.6) | 1.1 (0.9 to 1.3) | 1.2 (1.0 to 1.5) | 0.8 (0.5 to 1.2) |
| Libya | 57.7 (54.2 to 61.6) | 66.6 (64.8 to 68.4) | 66.4 (62.3 to 70.6) | 0.5 (0.1 to 0.8) | 0.7 (0.4 to 1.0) | -0.0 (-0.8 to 0.7) |
| Morocco | 41.0 (38.0 to 44.3) | 53.2 (49.5 to 57.6) | 57.8 (54.6 to 62.5) | 1.2 (0.8 to 1.6) | 1.3 (0.8 to 1.9) | 0.9 (0.1 to 1.7) |
| Oman | 53.5 (49.7 to 57.6) | 61.6 (59.9 to 63.2) | 71.2 (69.0 to 73.3) | 1.0 (0.7 to 1.3) | 0.7 (0.3 to 1.1) | 1.6 (1.3 to 2.0) |
| Palestine | 47.2 (43.9 to 50.6) | 56.6 (54.7 to 58.4) | 61.3 (58.3 to 64.1) | 0.9 (0.6 to 1.2) | 0.9 (0.5 to 1.4) | 0.9 (0.3 to 1.5) |
| Qatar | 60.0 (55.4 to 63.7) | 76.4 (72.2 to 79.3) | 80.7 (75.9 to 83.3) | 1.0 (0.7 to 1.3) | 1.2 (0.8 to 1.6) | 0.6 (-0.0 to 1.3) |
| Saudi Arabia | 50.1 (45.3 to 55.6) | 58.4 (55.9 to 61.2) | 64.2 (59.6 to 68.9) | 0.8 (0.4 to 1.3) | 0.8 (0.2 to 1.3) | 1.0 (0.1 to 1.9) |
| Sudan | 27.3 (22.4 to 33.2) | 44.3 (39.5 to 49.6) | 51.9 (47.1 to 56.6) | 2.2 (1.5 to 2.9) | 2.4 (1.4 to 3.5) | 1.7 (0.8 to 2.7) |
| Syria | 45.3 (42.0 to 49.3) | 56.4 (52.9 to 59.8) | 57.6 (52.6 to 62.5) | 0.8 (0.3 to 1.3) | 1.1 (0.5 to 1.6) | 0.2 (-0.9 to 1.3) |
| Tunisia | 55.9 (53.0 to 58.9) | 63.5 (58.6 to 68.9) | 68.2 (61.7 to 73.8) | 0.7 (0.3 to 1.0) | 0.6 (0.1 to 1.2) | 0.8 (-0.2 to 1.6) |
| Turkey | 47.8 (44.9 to 50.7) | 64.1 (61.6 to 66.8) | 69.2 (64.6 to 73.9) | 1.3 (1.0 to 1.6) | 1.5 (1.2 to 1.8) | 0.8 (0.0 to 1.7) |
| United Arab Emirates | 55.1 (50.5 to 59.7) | 63.4 (59.4 to 67.1) | 63.2 (56.8 to 69.9) | 0.5 (0.0 to 0.9) | 0.7 (0.2 to 1.2) | -0.0 (-1.1 to 1.2) |
| Yemen | 30.1 (24.1 to 37.7) | 47.7 (42.4 to 52.6) | 49.1 (43.8 to 53.9) | 1.7 (0.9 to 2.5) | 2.3 (1.3 to 3.4) | 0.3 (-0.7 to 1.3) |
| **South Asia** | **28.4 (25.2 to 31.8)** | **42.1 (39.6 to 44.6)** | **46.5 (42.8 to 50.1)** | **1.7 (1.3 to 2.1)** | **2.0 (1.5 to 2.5)** | **1.1 (0.2 to 2.0)** |
| **South Asia** | **28.4 (25.2 to 31.8)** | **42.1 (39.6 to 44.6)** | **46.5 (42.8 to 50.1)** | **1.7 (1.3 to 2.1)** | **2.0 (1.5 to 2.5)** | **1.1 (0.2 to 2.0)** |
| Bangladesh | 31.7 (28.2 to 35.5) | 50.3 (47.3 to 52.8) | 53.7 (49.6 to 58.3) | 1.8 (1.3 to 2.3) | 2.3 (1.7 to 2.9) | 0.8 (-0.1 to 1.6) |
| Bhutan | 42.0 (35.0 to 52.0) | 51.8 (47.7 to 56.5) | 51.4 (45.3 to 57.3) | 0.7 (-0.2 to 1.4) | 1.1 (-0.1 to 2.0) | -0.1 (-1.3 to 0.9) |
| India | 27.0 (23.1 to 30.7) | 42.1 (39.3 to 45.0) | 46.8 (42.4 to 51.5) | 1.9 (1.4 to 2.5) | 2.2 (1.7 to 2.9) | 1.2 (0.1 to 2.3) |
| Nepal | 29.3 (25.9 to 33.0) | 44.8 (41.4 to 49.4) | 47.2 (43.1 to 51.7) | 1.7 (1.1 to 2.2) | 2.1 (1.4 to 2.9) | 0.6 (-0.4 to 1.5) |
| Pakistan | 36.3 (31.8 to 41.1) | 35.1 (30.5 to 40.0) | 39.3 (33.1 to 45.1) | 0.3 (-0.4 to 0.8) | -0.2 (-1.0 to 0.6) | 1.2 (-0.8 to 3.3) |
| **Southeast Asia, east Asia, and Oceania** | **48.5 (46.0 to 51.1)** | **59.4 (56.9 to 61.6)** | **65.0 (61.0 to 68.5)** | **1.0 (0.8 to 1.3)** | **1.0 (0.7 to 1.3)** | **1.0 (0.2 to 1.7)** |
| **East Asia** | **49.9 (46.9 to 53.2)** | **63.1 (60.0 to 65.9)** | **69.7 (64.1 to 74.6)** | **1.1 (0.8 to 1.5)** | **1.2 (0.8 to 1.5)** | **1.1 (0.1 to 2.0)** |
| China | 49.7 (46.6 to 53.2) | 63.1 (59.9 to 66.0) | 69.9 (64.1 to 74.9) | 1.2 (0.8 to 1.5) | 1.2 (0.8 to 1.6) | 1.1 (0.1 to 2.1) |
| North Korea | 46.5 (42.9 to 50.5) | 49.7 (47.3 to 53.0) | 52.9 (49.5 to 56.1) | 0.4 (0.1 to 0.8) | 0.3 (-0.1 to 0.8) | 0.7 (-0.0 to 1.3) |
| Taiwan (province of China) | 62.7 (60.8 to 64.6) | 77.4 (75.7 to 78.9) | 79.3 (74.7 to 82.7) | 0.8 (0.6 to 1.0) | 1.0 (0.9 to 1.2) | 0.3 (-0.4 to 0.8) |
| **Oceania** | **37.4 (34.5 to 40.3)** | **35.3 (31.3 to 39.3)** | **39.0 (32.9 to 45.5)** | **0.2 (-0.4 to 0.7)** | **-0.3 (-0.8 to 0.2)** | **1.2 (-0.0 to 2.3)** |
| American Samoa | 53.9 (51.3 to 56.0) | 54.1 (51.5 to 56.0) | 53.3 (49.7 to 56.3) | -0.0 (-0.3 to 0.2) | 0.0 (-0.2 to 0.3) | -0.2 (-0.8 to 0.5) |
| Cook Islands | 52.9 (50.2 to 55.3) | 64.5 (62.3 to 66.7) | 62.3 (58.2 to 66.5) | 0.6 (0.3 to 0.9) | 1.0 (0.7 to 1.3) | -0.4 (-1.1 to 0.3) |
| Fiji | 44.3 (41.2 to 47.6) | 42.4 (40.7 to 44.3) | 45.2 (40.4 to 49.9) | 0.1 (-0.4 to 0.5) | -0.2 (-0.6 to 0.2) | 0.7 (-0.5 to 1.8) |
| Guam | 63.0 (60.9 to 64.9) | 67.1 (65.2 to 68.9) | 63.8 (60.6 to 66.8) | 0.0 (-0.2 to 0.2) | 0.3 (0.1 to 0.5) | -0.6 (-1.2 to -0.0) |
| Kiribati | 25.8 (23.4 to 28.8) | 32.0 (29.6 to 35.2) | 35.7 (32.1 to 39.8) | 1.1 (0.5 to 1.7) | 1.1 (0.4 to 1.7) | 1.2 (0.1 to 2.3) |
| Marshall Islands | 38.7 (36.4 to 41.4) | 42.1 (38.9 to 45.9) | 43.9 (39.1 to 49.5) | 0.4 (-0.1 to 0.9) | 0.4 (-0.1 to 0.9) | 0.5 (-0.8 to 1.7) |
| Federated States of Micronesia | 33.9 (30.2 to 38.3) | 37.7 (34.9 to 41.8) | 34.3 (31.1 to 39.8) | 0.0 (-0.5 to 0.7) | 0.6 (-0.2 to 1.3) | -1.1 (-2.1 to 0.0) |
| Nauru | 39.5 (37.0 to 42.6) | 38.4 (35.7 to 40.9) | 41.9 (39.0 to 46.1) | 0.2 (-0.1 to 0.5) | -0.2 (-0.6 to 0.2) | 1.0 (0.2 to 2.0) |
| Niue | 47.8 (44.5 to 50.6) | 48.4 (45.3 to 51.6) | 48.9 (44.8 to 53.3) | 0.1 (-0.3 to 0.5) | 0.1 (-0.3 to 0.4) | 0.1 (-0.6 to 0.9) |
| Northern Mariana Islands | 62.6 (60.2 to 64.5) | 63.6 (61.6 to 65.9) | 60.4 (58.1 to 63.0) | -0.1 (-0.3 to 0.1) | 0.1 (-0.1 to 0.3) | -0.6 (-1.1 to -0.1) |
| Palau | 46.1 (41.4 to 49.8) | 49.4 (46.2 to 52.9) | 44.8 (41.1 to 49.6) | -0.1 (-0.5 to 0.4) | 0.3 (-0.2 to 1.0) | -1.0 (-2.0 to -0.0) |
| Papua New Guinea | 35.5 (31.6 to 39.7) | 33.0 (27.8 to 38.1) | 37.6 (30.2 to 45.3) | 0.2 (-0.5 to 0.9) | -0.4 (-1.1 to 0.3) | 1.5 (-0.0 to 3.0) |

| | UHC effective coverage index | | | Annualised rate of change (%) | | |
|---|---|---|---|---|---|---|
| | 1990 | 2010 | 2019 | 1990-2019 | 1990-2010 | 2010-2019 |
| Samoa | 39.5 (35.1 to 43.4) | 43.5 (39.3 to 47.2) | 49.8 (44.4 to 55.0) | 0.8 (0.3 to 1.3) | 0.5 (-0.2 to 1.2) | 1.5 (0.4 to 2.6) |
| Solomon Islands | 25.3 (22.4 to 28.8) | 33.1 (30.8 to 35.6) | 39.3 (37.0 to 41.8) | 1.5 (1.0 to 2.0) | 1.3 (0.6 to 2.0) | 1.9 (1.1 to 2.7) |
| Tokelau | 43.2 (38.4 to 47.4) | 52.6 (47.9 to 56.5) | 52.7 (47.4 to 57.7) | 0.7 (0.2 to 1.2) | 1.0 (0.4 to 1.6) | 0.0 (-0.8 to 0.9) |
| Tonga | 53.9 (50.9 to 57.5) | 51.7 (47.9 to 55.4) | 52.6 (47.6 to 56.8) | -0.1 (-0.5 to 0.3) | -0.2 (-0.7 to 0.2) | 0.1 (-0.7 to 1.0) |
| Tuvalu | 29.2 (25.9 to 33.1) | 38.8 (35.2 to 42.9) | 39.6 (34.1 to 45.0) | 1.1 (0.2 to 1.7) | 1.4 (0.5 to 2.2) | 0.2 (-1.1 to 1.4) |
| Vanuatu | 33.2 (28.3 to 39.0) | 33.6 (29.4 to 37.9) | 34.1 (29.2 to 39.7) | 0.1 (-0.7 to 0.8) | 0.1 (-0.9 to 1.0) | 0.1 (-1.4 to 1.7) |
| **Southeast Asia** | **44.8 (42.8 to 46.7)** | **51.2 (49.4 to 53.1)** | **55.1 (52.0 to 58.0)** | **0.7 (0.5 to 0.9)** | **0.7 (0.4 to 0.9)** | **0.8 (0.1 to 1.5)** |
| Cambodia | 30.1 (26.5 to 33.6) | 50.0 (46.9 to 53.2) | 56.9 (53.7 to 61.1) | 2.2 (1.7 to 2.7) | 2.5 (1.9 to 3.3) | 1.5 (0.7 to 2.3) |
| Indonesia | 38.6 (35.4 to 41.9) | 47.3 (43.9 to 51.0) | 48.6 (42.2 to 54.9) | 0.8 (0.3 to 1.3) | 1.0 (0.5 to 1.5) | 0.3 (-1.4 to 1.8) |
| Laos | 15.4 (11.4 to 19.4) | 34.3 (29.2 to 40.3) | 43.8 (38.3 to 49.2) | 3.6 (2.7 to 4.7) | 4.0 (2.6 to 5.6) | 2.7 (1.1 to 4.4) |
| Malaysia | 51.3 (49.4 to 53.0) | 62.2 (60.3 to 63.8) | 66.6 (62.3 to 70.7) | 0.9 (0.6 to 1.2) | 1.0 (0.8 to 1.1) | 0.8 (-0.0 to 1.5) |
| Maldives | 36.8 (33.7 to 40.2) | 63.3 (61.4 to 65.1) | 66.9 (64.0 to 69.9) | 2.1 (1.7 to 2.4) | 2.7 (2.3 to 3.1) | 0.6 (-0.0 to 1.2) |
| Mauritius | 43.8 (41.5 to 46.0) | 49.0 (47.0 to 51.0) | 55.9 (51.3 to 60.0) | 0.8 (0.5 to 1.2) | 0.6 (0.4 to 0.8) | 1.4 (0.5 to 2.3) |
| Myanmar | 28.0 (23.6 to 33.3) | 39.1 (35.5 to 42.5) | 46.9 (42.9 to 50.9) | 1.8 (1.1 to 2.5) | 1.6 (0.7 to 2.7) | 2.1 (0.9 to 3.2) |
| Philippines | 59.8 (56.2 to 62.9) | 49.2 (45.9 to 52.3) | 54.8 (48.5 to 60.5) | -0.3 (-0.7 to 0.1) | -1.0 (-1.3 to -0.6) | 1.2 (-0.2 to 2.4) |
| Seychelles | 53.9 (52.0 to 55.8) | 60.2 (58.5 to 61.8) | 61.5 (59.5 to 63.5) | 0.5 (0.3 to 0.6) | 0.5 (0.4 to 0.7) | 0.2 (-0.2 to 0.7) |
| Sri Lanka | 53.1 (50.4 to 56.0) | 57.1 (55.0 to 59.0) | 65.6 (59.7 to 71.2) | 0.7 (0.3 to 1.1) | 0.4 (0.1 to 0.6) | 1.5 (0.4 to 2.5) |
| Thailand | 57.6 (55.2 to 59.9) | 65.5 (62.8 to 68.0) | 71.6 (66.2 to 76.6) | 0.8 (0.4 to 1.0) | 0.6 (0.4 to 0.9) | 1.0 (0.0 to 1.9) |
| Timor-Leste | 30.4 (26.3 to 36.2) | 45.2 (40.6 to 53.1) | 45.8 (41.1 to 51.6) | 1.4 (0.6 to 2.1) | 2.0 (1.0 to 3.0) | 0.2 (-1.4 to 1.4) |
| Viet Nam | 47.3 (43.2 to 51.6) | 56.7 (54.8 to 58.5) | 59.7 (56.7 to 63.0) | 0.8 (0.4 to 1.2) | 0.9 (0.5 to 1.4) | 0.6 (0.0 to 1.2) |
| **Sub-Saharan Africa** | **27.1 (24.7 to 29.4)** | **35.4 (33.4 to 37.8)** | **45.0 (42.3 to 47.8)** | **1.8 (1.4 to 2.1)** | **1.3 (1.0 to 1.7)** | **2.6 (1.9 to 3.3)** |
| **Central sub-Saharan Africa** | **22.1 (18.5 to 26.1)** | **30.1 (26.3 to 34.8)** | **43.0 (37.9 to 48.1)** | **2.3 (1.6 to 3.0)** | **1.6 (0.6 to 2.5)** | **3.9 (2.5 to 5.3)** |
| Angola | 18.5 (14.2 to 23.9) | 31.5 (27.4 to 35.4) | 39.1 (34.1 to 44.1) | 2.6 (1.5 to 3.6) | 2.7 (1.2 to 4.2) | 2.4 (0.8 to 3.9) |
| Central African Republic | 17.6 (14.4 to 21.7) | 16.7 (12.5 to 22.3) | 22.2 (16.3 to 29.3) | 0.8 (-0.6 to 2.1) | -0.3 (-2.1 to 1.5) | 3.0 (-0.0 to 6.3) |
| Congo (Brazzaville) | 25.2 (21.4 to 29.7) | 36.4 (32.2 to 41.0) | 43.9 (38.6 to 49.3) | 1.9 (1.2 to 2.6) | 1.8 (0.9 to 2.8) | 2.1 (0.7 to 3.4) |
| DR Congo | 22.8 (18.3 to 27.8) | 29.7 (24.9 to 35.7) | 45.1 (39.5 to 51.1) | 2.3 (1.6 to 3.2) | 1.3 (0.1 to 2.5) | 4.6 (2.9 to 6.3) |
| Equatorial Guinea | 16.7 (12.1 to 21.9) | 42.6 (35.7 to 49.4) | 50.1 (42.3 to 56.3) | 3.8 (2.6 to 5.0) | 4.7 (3.1 to 6.4) | 1.8 (0.4 to 3.3) |
| Gabon | 35.8 (31.6 to 40.0) | 41.8 (38.4 to 45.9) | 52.9 (48.4 to 58.1) | 1.3 (0.9 to 1.9) | 0.8 (0.1 to 1.6) | 2.7 (1.5 to 3.7) |
| **Eastern sub-Saharan Africa** | **25.6 (23.4 to 27.9)** | **37.9 (35.9 to 40.3)** | **48.1 (45.5 to 51.2)** | **2.2 (1.9 to 2.5)** | **2.0 (1.6 to 2.3)** | **2.7 (2.1 to 3.1)** |
| Burundi | 27.0 (21.8 to 32.8) | 38.0 (32.9 to 43.4) | 50.0 (43.5 to 55.8) | 2.1 (1.3 to 3.0) | 1.7 (0.6 to 2.9) | 3.0 (1.8 to 4.2) |
| Comoros | 34.2 (27.3 to 54.0) | 41.8 (36.8 to 46.6) | 48.2 (42.7 to 53.8) | 1.2 (-0.4 to 2.0) | 1.0 (-1.3 to 2.1) | 1.6 (0.3 to 2.8) |
| Djibouti | 39.1 (32.8 to 44.6) | 40.6 (34.5 to 47.1) | 45.4 (38.7 to 52.0) | 0.5 (-0.2 to 1.2) | 0.2 (-0.7 to 1.2) | 1.2 (-0.3 to 2.7) |
| Eritrea | 17.7 (12.2 to 23.5) | 35.0 (30.3 to 40.2) | 42.3 (35.9 to 48.2) | 3.0 (1.9 to 4.3) | 3.4 (1.9 to 5.3) | 2.1 (0.7 to 3.3) |
| Ethiopia | 10.0 (7.3 to 13.4) | 35.8 (32.3 to 39.5) | 46.5 (42.3 to 51.0) | 5.3 (4.3 to 6.5) | 6.3 (5.0 to 8.0) | 2.9 (2.0 to 3.9) |
| Kenya | 42.8 (38.9 to 47.7) | 42.0 (38.5 to 45.8) | 51.7 (46.7 to 56.2) | 0.6 (0.3 to 0.9) | -0.1 (-0.5 to 0.3) | 2.3 (1.5 to 3.1) |
| Madagascar | 30.6 (26.7 to 34.4) | 36.4 (32.3 to 40.5) | 39.7 (34.2 to 45.4) | 0.9 (0.3 to 1.5) | 0.9 (-0.0 to 1.7) | 1.0 (-0.4 to 2.3) |
| Malawi | 31.8 (28.1 to 35.4) | 41.8 (38.6 to 45.3) | 55.5 (51.9 to 59.1) | 1.9 (1.5 to 2.4) | 1.4 (0.8 to 2.0) | 3.1 (2.2 to 4.0) |
| Mozambique | 23.9 (19.2 to 29.8) | 31.5 (27.9 to 35.5) | 44.0 (39.4 to 48.7) | 2.1 (1.3 to 3.0) | 1.4 (0.3 to 2.5) | 3.7 (2.3 to 5.1) |
| Rwanda | 23.4 (19.6 to 27.8) | 45.4 (42.0 to 49.1) | 59.3 (54.5 to 64.4) | 3.2 (2.5 to 3.8) | 3.3 (2.5 to 4.1) | 3.0 (2.2 to 3.8) |
| Somalia | 15.2 (9.9 to 20.9) | 18.8 (13.0 to 25.5) | 24.1 (17.1 to 31.1) | 1.6 (0.0 to 3.0) | 1.1 (-0.9 to 3.0) | 2.7 (-0.5 to 5.8) |
| South Sudan | 31.8 (24.8 to 37.9) | 34.5 (28.3 to 40.9) | 41.6 (34.5 to 49.6) | 0.9 (0.1 to 1.8) | 0.4 (-0.6 to 1.5) | 2.1 (0.3 to 3.9) |

| | UHC effective coverage index | | | Annualised rate of change (%) | | |
|---|---|---|---|---|---|---|
| | 1990 | 2010 | 2019 | 1990-2019 | 1990-2010 | 2010-2019 |
| Uganda | 29.1<br>(25.6 to 32.9) | 39.9<br>(36.2 to 44.4) | 52.7<br>(49.0 to 56.9) | 2.0<br>(1.6 to 2.5) | 1.6<br>(0.9 to 2.3) | 3.1<br>(2.0 to 4.2) |
| Tanzania | 35.5<br>(31.4 to 40.1) | 45.1<br>(41.4 to 49.3) | 55.2<br>(51.1 to 59.3) | 1.5<br>(1.1 to 2.0) | 1.2<br>(0.6 to 1.9) | 2.2<br>(1.3 to 3.2) |
| Zambia | 29.8<br>(26.0 to 33.6) | 38.2<br>(35.1 to 41.1) | 52.7<br>(49.0 to 56.6) | 2.0<br>(1.4 to 2.6) | 1.2<br>(0.5 to 2.0) | 3.6<br>(2.5 to 4.5) |
| **Southern sub-Saharan Africa** | **47.3<br>(44.3 to 49.9)** | **39.6<br>(37.6 to 41.7)** | **58.1<br>(55.5 to 60.6)** | **0.7<br>(0.5 to 1.0)** | **-0.9<br>(-1.2 to -0.5)** | **4.3<br>(3.7 to 4.8)** |
| Botswana | 44.6<br>(40.2 to 48.8) | 46.4<br>(42.1 to 51.5) | 57.5<br>(52.5 to 62.4) | 0.9<br>(0.5 to 1.3) | 0.2<br>(-0.3 to 0.8) | 2.4<br>(1.3 to 3.3) |
| eSwatini | 44.8<br>(40.7 to 49.0) | 35.0<br>(30.8 to 39.5) | 53.3<br>(48.4 to 58.6) | 0.6<br>(0.1 to 1.1) | -1.2<br>(-2.1 to -0.4) | 4.7<br>(3.4 to 6.1) |
| Lesotho | 39.4<br>(35.4 to 43.4) | 31.8<br>(28.3 to 35.6) | 38.6<br>(33.9 to 44.3) | -0.1<br>(-0.6 to 0.5) | -1.1<br>(-1.8 to -0.3) | 2.1<br>(1.0 to 3.3) |
| Namibia | 43.8<br>(39.9 to 48.8) | 49.2<br>(45.6 to 53.0) | 62.1<br>(57.4 to 66.8) | 1.2<br>(0.7 to 1.6) | 0.6<br>(-0.0 to 1.1) | 2.6<br>(1.8 to 3.4) |
| South Africa | 48.8<br>(45.2 to 51.9) | 40.7<br>(38.1 to 43.6) | 59.7<br>(56.7 to 62.8) | 0.7<br>(0.5 to 1.0) | -0.9<br>(-1.3 to -0.5) | 4.3<br>(3.6 to 4.9) |
| Zimbabwe | 44.5<br>(40.7 to 47.5) | 34.2<br>(31.7 to 37.4) | 54.5<br>(50.3 to 58.5) | 0.7<br>(0.3 to 1.1) | -1.3<br>(-1.7 to -0.8) | 5.1<br>(4.1 to 6.1) |
| **Western sub-Saharan Africa** | **24.4<br>(20.9 to 27.9)** | **33.6<br>(30.4 to 37.4)** | **40.2<br>(36.3 to 44.5)** | **1.7<br>(1.2 to 2.4)** | **1.6<br>(0.9 to 2.3)** | **2.0<br>(0.6 to 3.2)** |
| Benin | 27.5<br>(23.1 to 31.5) | 35.6<br>(30.8 to 40.4) | 44.7<br>(38.2 to 51.0) | 1.7<br>(0.9 to 2.4) | 1.3<br>(0.3 to 2.3) | 2.5<br>(1.1 to 3.9) |
| Burkina Faso | 22.8<br>(18.8 to 27.3) | 37.6<br>(34.0 to 42.9) | 41.7<br>(37.8 to 46.6) | 2.1<br>(1.4 to 2.8) | 2.5<br>(1.7 to 3.5) | 1.1<br>(-0.2 to 2.4) |
| Cape Verde | 49.8<br>(47.4 to 52.3) | 61.2<br>(59.2 to 63.1) | 62.3<br>(58.6 to 65.1) | 0.8<br>(0.5 to 1.0) | 1.0<br>(0.8 to 1.3) | 0.2<br>(-0.5 to 0.8) |
| Cameroon | 29.1<br>(24.8 to 34.2) | 30.9<br>(26.7 to 35.6) | 42.3<br>(36.6 to 48.5) | 1.3<br>(0.6 to 2.0) | 0.3<br>(-0.7 to 1.3) | 3.5<br>(1.8 to 5.0) |
| Chad | 21.0<br>(16.7 to 25.2) | 26.0<br>(22.4 to 30.1) | 31.4<br>(26.4 to 36.7) | 1.4<br>(0.5 to 2.4) | 1.1<br>(-0.1 to 2.4) | 2.1<br>(-0.0 to 4.0) |
| Côte d'Ivoire | 26.4<br>(21.9 to 31.6) | 33.7<br>(28.8 to 38.8) | 42.7<br>(37.8 to 49.3) | 1.7<br>(1.0 to 2.4) | 1.2<br>(0.3 to 2.2) | 2.7<br>(1.2 to 4.1) |
| The Gambia | 47.6<br>(41.1 to 54.2) | 45.8<br>(42.3 to 49.9) | 48.1<br>(43.5 to 53.5) | 0.0<br>(-0.6 to 0.7) | -0.2<br>(-0.9 to 0.6) | 0.5<br>(-0.6 to 1.6) |
| Ghana | 29.5<br>(24.6 to 34.7) | 41.5<br>(38.1 to 45.3) | 49.2<br>(45.5 to 53.1) | 1.8<br>(1.1 to 2.5) | 1.7<br>(0.8 to 2.7) | 1.8<br>(0.9 to 2.8) |
| Guinea | 20.8<br>(16.3 to 25.2) | 31.4<br>(26.5 to 36.1) | 32.4<br>(26.1 to 38.3) | 1.5<br>(0.5 to 2.5) | 2.1<br>(0.9 to 3.3) | 0.4<br>(-1.8 to 2.3) |
| Guinea-Bissau | 18.7<br>(14.6 to 23.3) | 23.8<br>(20.3 to 28.2) | 35.8<br>(30.4 to 41.5) | 2.2<br>(1.3 to 3.4) | 1.2<br>(-0.1 to 2.7) | 4.5<br>(2.4 to 6.2) |
| Liberia | 19.2<br>(14.9 to 23.3) | 36.8<br>(32.8 to 41.2) | 47.6<br>(41.9 to 53.7) | 3.1<br>(2.3 to 4.1) | 3.3<br>(2.3 to 4.6) | 2.9<br>(1.6 to 4.1) |
| Mali | 20.8<br>(16.7 to 25.6) | 35.5<br>(31.0 to 39.8) | 40.8<br>(34.5 to 46.6) | 2.3<br>(1.4 to 3.2) | 2.6<br>(1.5 to 3.8) | 1.6<br>(-0.1 to 3.0) |
| Mauritania | 27.2<br>(22.1 to 32.4) | 46.3<br>(42.2 to 50.1) | 53.1<br>(47.5 to 59.6) | 2.3<br>(1.6 to 3.2) | 2.7<br>(1.8 to 3.7) | 1.5<br>(0.3 to 2.8) |
| Niger | 18.0<br>(13.5 to 22.6) | 31.6<br>(26.2 to 37.4) | 35.1<br>(28.4 to 41.5) | 2.3<br>(1.2 to 3.4) | 2.9<br>(1.5 to 4.2) | 1.2<br>(-1.0 to 3.0) |
| Nigeria | 22.6<br>(16.9 to 28.3) | 31.5<br>(26.3 to 37.2) | 38.4<br>(31.8 to 44.7) | 1.8<br>(0.9 to 2.9) | 1.7<br>(0.3 to 3.1) | 2.2<br>(-0.4 to 4.5) |
| São Tomé and PrÍncipe | 41.6<br>(38.0 to 48.0) | 51.0<br>(48.3 to 54.4) | 54.7<br>(50.7 to 59.1) | 0.9<br>(0.4 to 1.3) | 1.0<br>(0.3 to 1.6) | 0.8<br>(0.0 to 1.5) |
| Senegal | 31.9<br>(27.6 to 38.7) | 45.0<br>(41.8 to 49.1) | 49.4<br>(44.9 to 54.9) | 1.5<br>(0.7 to 2.2) | 1.7<br>(0.8 to 2.4) | 1.0<br>(-0.1 to 2.2) |
| Sierra Leone | 28.2<br>(23.3 to 34.2) | 36.8<br>(31.5 to 42.3) | 42.1<br>(35.8 to 48.7) | 1.4<br>(0.6 to 2.2) | 1.3<br>(0.2 to 2.3) | 1.5<br>(-0.2 to 3.1) |
| Togo | 32.6<br>(28.4 to 36.7) | 33.6<br>(29.7 to 37.7) | 42.8<br>(37.9 to 48.0) | 0.9<br>(0.3 to 1.5) | 0.2<br>(-0.6 to 1.0) | 2.7<br>(1.3 to 4.0) |
